# A comprehensive metabolomic data set of date palm fruit

**DOI:** 10.1016/j.dib.2018.04.012

**Published:** 2018-04-10

**Authors:** Nisha Stephan, Anna Halama, Sweety Mathew, Shahina Hayat, Aditya Bhagwat, Lisa Sara Mathew, Ilham Diboun, Joel Malek, Karsten Suhre

**Affiliations:** a Department of Physiology and Biophysics, Weill Cornell Medicine-Qatar, Education City, P.O. Box 24144, Doha, Qatar; b Genomics Core, Weill Cornell Medicine-Qatar, Education City, P.O. Box 24144 , Doha, Qatar

## Abstract

This article provides detailed information on the phenotypes and the metabolic profiles of 196 date fruits from 123 unique date fruit varieties. These date fruits are extensively diverse in their country of origin, variety and post harvesting conditions. We used a non-targeted mass-spectrometry based metabolomics approach to metabolically characterize date fruits, and measured 427 metabolites from a wide range of metabolic pathways.

The metabolomics data for all the date fruit samples are available at the NIH Common Fund's Data Repository and Coordinating Center (supported by NIH grant, U01-DK097430) website, http://www.metabolomicsworkbench.org), under Metabolomics Workbench StudyID: ST000867. The data are directly accessible at http://www.metabolomicsworkbench.org/data/DRCCMetadata.php?Mode=Study&StudyID=ST000867&StudyType=MS&ResultType=1.

**Specifications Table**

**Table:** 

Subject area	*Plant Biology*
More specific subject area	*Plant physiology, Metabolomics*
Type of data	*Tables, Figures, images*
How data was acquired	*non-targeted mass-spectrometry based metabolomics*
Data format	*Raw data, image files*
Experimental factors	*Samples were frozen at* − 80 °C *without any treatment*
Experimental features	*Samples were preprocessed and the metabolite measurement was done on* Gas Chromatography Mass Spectrometry (GC–MS) and the Orbitrap Elite Accurate Liquid Chromatography Mass Spectrometry (LC–MS/MS) platforms
Data source location	Samples were collected from 14 different countries, including Qatar, UAE, Iran, Saudi Arabia, Egypt, Pakistan, Libya, Tunisia, USA, Morocco, Jordan, Sudan, Oman and Spain and metabolomics analysis was done by Metabolon Inc.
Data accessibility	*The data is deposited to data repository, Metabolomics Workbench under StudyID: ST000867. Also available with this article.*

**Value of the data**

• This data presents the metabolic variation in date fruits and can be used to identify healthiest varieties.
• Since the data includes fresh and processed samples, the impact of date fruit processing can be investigated.
• Fruits from all collected varieties are available at our Bio-bank for future collaborative research.

## 1 Data

Here we describe a freely available non-targeted metabolomics data set together with phenotypic information of 196 date fruit samples. To provide wide geographical coverage, we collected dates from 14 different countries including Qatar, United Arab Emirates (UAE), Iran, Saudi Arabia, Egypt, Pakistan, Libya, Tunisia, United States of America (USA), Morocco, Jordan, Sudan, Oman and Spain. Since developmental stage can impact the metabolic profile, we included date fruits at different ripening stages from ten different varieties. Additionally, biological replicates from forty varieties were included.

## 2 Experimental design, materials and methods

### 2.1 Sample collection

The samples were collected in two separate batches. A first collection was performed in 2012 followed by a second collection in 2013. The first batch contained only mature dates, whereas in the second batch included both mature dates and dates at different ripening stages. Dates were mostly acquired from commercial sources such as shops, markets and date festivals. Depending on availability, up to ten individual dates from same date variety were collected.

### 2.2 Experimental design

Each individual date fruit was assigned a unique identifier (“Sample name” in the database). Similarly, another unique identifier was assigned to each date fruit variety under “Subject name” in the database. Different “Subject name” were assigned to date fruits of the same variety collected from different countries. In the database, we used “Subject name” to represent the date fruit variety and “Sample name” to represent individual date fruits to comply with data repository standards. The value “replicate” in the column “Sample replicate” indicates that the date fruit is a biological replicate of the existing date fruit variety and the variety can be identified from the “Subject name”. For example, “Sample name” 41.1 and 41.3 indicates date fruits from the Sufri variety depicted by “41” (present under “Subject name”), and “.1”, and “.3” in the “Sample name” reflect replicate one and three, respectively. An additional column “Ripening stage” is included to state the ripening stage of the date fruit. For mature fruits, this column will have value “0” while values “− 1”, “− 2”, “− 3” etc. represent the pre-ripening stages (with “− 3” being the least ripen). Hence date fruit varieties with different pre-ripening stages will have non-zero values for “Ripening stage” and the variety can be identified from the “Subject name”. For example, “Sample name” 90A, 90B and 90C indicates date fruits from Mejhool variety depicted by “90” (present under “Subject name”), and “0”, “− 1” and “− 2” in the “Ripening stage” indicate different ripening stages.

### 2.3 Phenotypic characterization

**Fig. 1:**
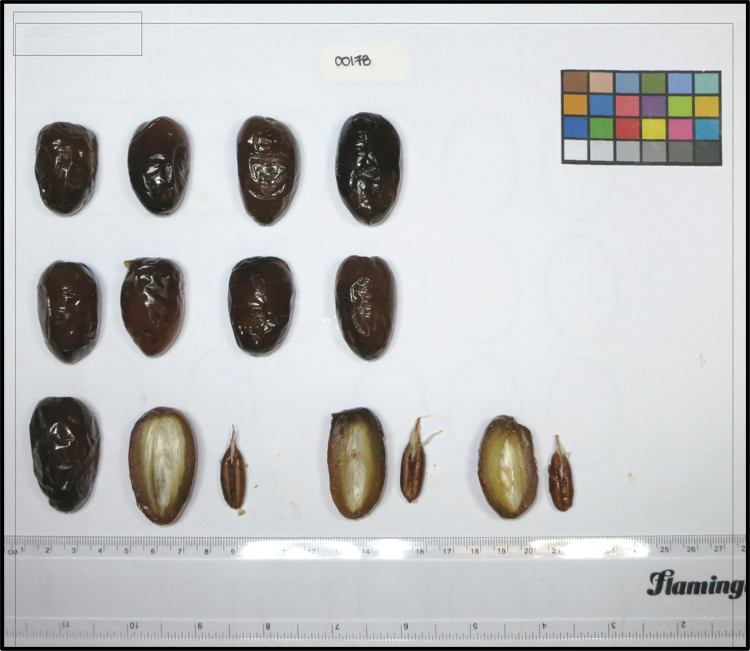
Sample photograph of one mature date fruit variety (Subject name 178).

Each date fruit was phenotypically classified by length, width, seed length and weight, and a photograph of each date fruit was recorded. Description of the phenotypes can be found in Table 1 and the phenotype data for all samples are provided as Additional file 1.xls. The metabolic profiling was performed in two batches, identified in the database as “Batch One” and “Batch Two”. Biological replicates of ten dates from Batch One were included in Batch Two to connect the semi-quantitative data between the two measurements. Each fruit was weighed and the weight was recorded for each date sample. Among the replicates, two fruits were halved to reveal the longitudinal and cross sectional appearance of the fruit pericarp and seed. A photograph was taken under artificial light using a Canon Power Shot S100 USA camera at a resolution of 4288 × 2848 pixels. A 20 cm ruler and an international Color Checker Color-Rendition Chart (Color Checker Classic, X-Rite, USA) were placed alongside the fruits on a white cardboard background. The camera was attached to a pre-set tripod so the background would be identical for all images. A sample photo is presented in Fig. 1 and photographs of all samples are provided along with the submission. The seed length was measured from the fruits that were previously halved to show the pericarp and seed.

**Table 1:** The description of samples phenotypes.

Header	Description
mb_sample_id	Sample Id given by Metabolomics Workbench
Subject name	Each distinct phenotypic class of dates is considered as unique variety here and also the same fruit variety collected from different countries has assigned a different Subject name.
Sample name	Unique name for each fruit sample
Genotyping sample id	Unique identifier used Internally
Date variety	Identified date fruit variety name and if not identified then 'Unnamed' as date variety
Average length(cm)	Average length of particular date fruit variety in centimetres
SD length(cm)	Standard deviation of length measurement in centimetres
No of samples for length	Number of date fruit samples used to calculate Average length of date variety
Average width(cm)	Average width of particular date fruit variety in centimetres
SD width(cm)	Standard deviation of width measurement in centimetres
No of samples for width	Number of date fruit samples used to calculate Average width of date variety
Average seed length(cm)	Average seed length of particular date fruit variety in centimetres
SD seed length(cm)	Standard deviation of seed length measurement in centimetres
No of samples for seed length	Number of date fruit samples used to calculate Average seed length of date variety
Average weight	Average weight for samples of particular date fruit variety
No of samples for weight	Number of date fruit samples used to calculate Average weight of date variety
Sample replicate	Indicates whether the sample is a replicate of existing fruit variety
Data set	Indicates the batch in which the samples are measured (Batch 1 or Batch 2)
Country of origin	Country of produce
Collection information	Some background information about the collection of samples, mainly the collection occasion.
Ripening stage	Indicates the ripening state of the date fruit sample. For mature fruits, this column will have value “0” and values “− 1”, “− 2”,”− 3” etc. represents the pre-ripening stages with “− 3” being the least ripened one.
Picture file	Name of the picture file for date fruit variety
Biosample availability	Indicates whether bio sample is available for that date fruit variety in internal dates biobank.

### 2.4 Sample pre-processing

The samples from “Batch One” were processed as previously described [1]. Briefly, 50 mg of the date fruit peel and flesh were flash frozen in liquid nitrogen and samples were homogenized twice for one minute at maximum speed using a mixer mill (Retsch, Germany). For metabolite extraction, a mixed solvent of methanol: methyl-tert-butyl-ether: water (1:3:1) was added to each homogenate and samples were shaken for 30 min at 40 °C and then incubated for 10 min in an ice cooled ultra-sonication bath. Upon incubation, 650 µL of UPLC-grade methanol: water (3:1) was added to the samples, followed by vortexing and centrifugation for 5 min at 40 °C in a table-top centrifuge (Eppendorf, Germany). The procedure described above led to a phase separation, providing the upper organic phase containing hydrophobic metabolites (lipids) and a lower aqueous phase containing a polar and semi-polar metabolite. The phases were separated, dried in a speedvac concentrator (Centrivac, Heraeus, Germany) and stored at − 80 °C for further analyses.

Batch Two was processed using a protocol by Metabolon Inc. [2]. Briefly, the samples were weighed and frozen at − 800 °C prior to extraction. To each frozen sample beads and water (8 µL per mg of sample) were added and the samples were homogenized in GenoGrinder (Glen Mills GenoGrinder 2000, Germany) at 1000 strokes per minute for 5 min. From each sample 30 µL was taken and the aliquots from all samples were pooled together to create sample matrix. The blanks were prepared by adding 700 µL of water to 3 cryovials, and were processed same as the samples.

### 2.5 Metabolite measurements

All samples were analysed at Metabolon Inc. as previously described [2]. Briefly, 100 µL of aliquot from each sample was transferred to the plates. Additional samples were processed in parallel to the samples for technical validation and quality control (QC) purposes. In total six blanks, six sample matrix and one human plasma sample were placed on each plate (100 µL of sample per well). To each sample, 450 µL of extraction solvent (MeOH containing 10 µL/ml chlorophenylalanine, 2.5 µL/ml 2-fluorophenylglycine, 25 µg/ml d-6 cholesterol and 25 µL/ml tridecanoic acid) was added. The samples were then shaken on the GenoGrinder (GenoGrinder, Spex, USA) at 675 strokes per minute for 2 min and centrifuged at 2000 rpm for 5 min on a Beckman centrifuge (Beckman GS-6R Centrifuge, USA) at 4 °C.

The sample extracts were divided into two equal aliquots for metabolite measurement on the Gas Chromatography Mass Spectrometry (GC–MS) and the Orbitrap Elite Accurate Liquid Chromatography Mass Spectrometry (LC–MS/MS) platforms. For GC–MS measurements, 250 µL of sample was transferred to auto sampler vial inserts, and for LC–MS/MS measurements samples were distributed among three PCR plates (110 µL aliquots/well), designated LC positive, LC negative and replicate set. The Hamilton robot (Hamilton Star, Germany) was used for sample transfer. All samples were dried for 120 min by using a Zymark Turbovap 96 (Zymark Turbovap, USA), followed by overnight incubation in a dry box to ensure optimal dryness of the sample.

The sample extracts assigned for the GC–MS measurements underwent re-drying for an additional 24 h under vacuum desiccation followed by derivatization with bistrimethyl-silyl-trifluoroacetamide (BSTFA) under dried nitrogen conditions. The column deployed for GC–MS analysis was 5% phenyl and the temperature ramp range was from 40 to 300 °C in a time span of 16 min. The GC–MS measurements were performed on a Thermo FinniganTM TRACETM DSQTM (ThermoFinnigan, USA) fast-scanning single–quadrupole mass spectrophotometer using electron impact ionization source.

The dried sample extracts assigned for LC–MS/MS analysis were reconstituted either in acidic or basic solvents, containing at least 11 injections of standards with fixed concentration. The acidic samples were analysed in acidic positive ion optimized conditions, and the basic samples were analysed using basic negative ion optimized conditions. The aliquots were injected independently and processed on separate, dedicated columns. The gradient elution was optimized for each sample type – for acidic samples water and methanol containing 0.1% formic acid were used and for basic samples water and methanol containing 6.5 mM ammonium bicarbonate were used [2]. Analysis was performed on coupled Waters ACUITY ultra-performance liquid chromatography (Waters Corporation, USA) with ThermoFischer Scientific Orbitrap Elite high-resolution accurate mass spectrometer (Thermo Fischer Scientific Inc., USA). The mass spectrometer unit was equipped with a heated electrospray ionization (HESI) source and an Orbitrap mass analyser. The mass spectra analysis alternated between MS and data dependent MS scans using dynamic exclusion.

### 2.6 Signal processing and metabolite calling

**Fig. 2:**
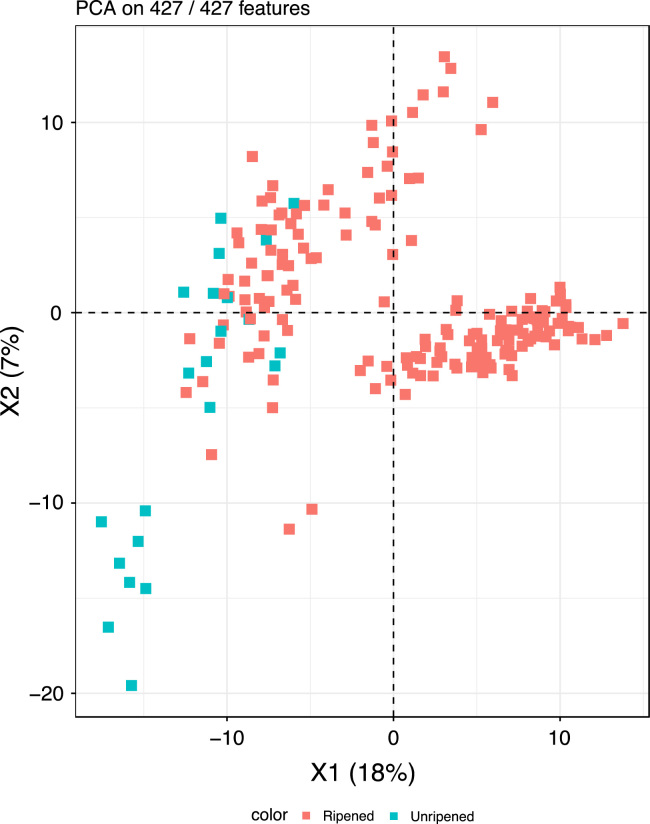
PCA sample plot showing ripened and Un-ripened date fruit metabolites.

**Fig. 3:**
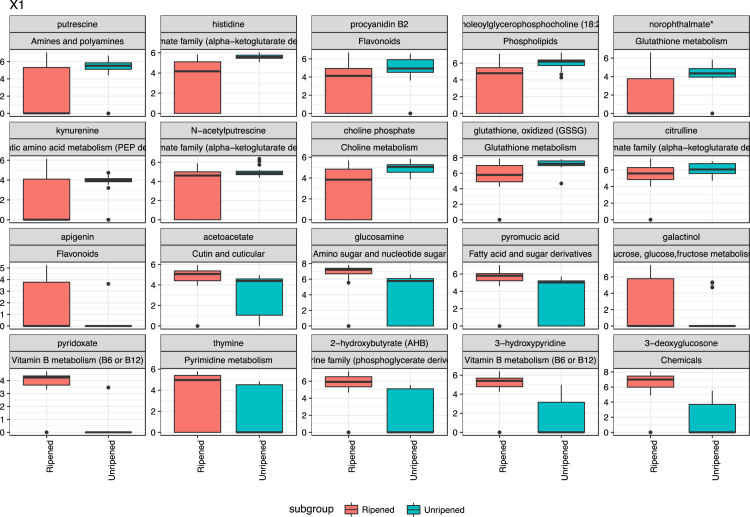
Box plot showing top 20 PC1 metabolites.

**Fig. 4:**
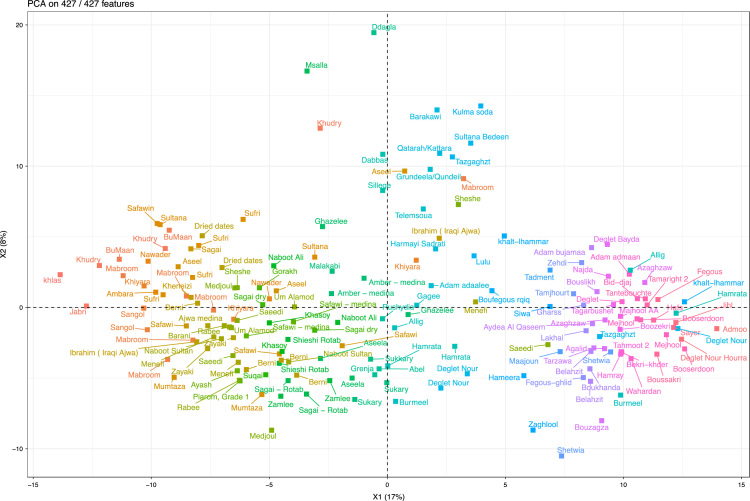
PCA sample plot after filtering out Un-ripened date fruits. PC1 score was mapped to colours so that the date fruits with similar metabolite profiles will have similar colours.

**Fig. 5:**
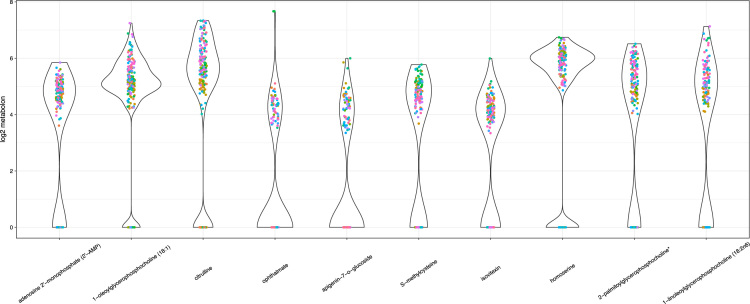
Violin plot showing distribution of log2 metabolite values for top 10 PC1 metabolites after removing Un-ripened date fruits.

The raw data files obtained from the GC–MS and LC–MS/MS platforms were extracted and processed by deploying an in-house developed pipeline with software and hardware for data extraction; peak and compound identification; and processing tools for quality control, and data interpretation and visualization. The component identification strategy is based on comparing and matching the obtained data to the Metabolon reference library, which contains information on retention index, retention time, chromatographic data, mass to charge ratio (m/z) and MS/MS spectral data of chemical standards [3]. An overview of metabolites identified can be found in Table 2 and the metabolomics data for all samples are provided as Additional file 2.xls. Fig. 2, Fig. 3, Fig. 4, Fig. 5 show various data exploration plots.

**Table 2:** An overview of metabolites identified in this study.

Metabolite super pathway	Number of metabolites in each category
Amino acids	93
Peptides	92
Lipids	85
Carbohydrates	55
Secondary metabolism	37
Nucleotides	37
Cofactors, Prosthetic Groups, Electron Carriers	22
Hormone metabolism	2
Xenobiotics	4

Source codes for the data exploration can be found on the bitbucket repository https://bitbucket.org/shh2026/datepalm.2018.

### 2.7 Data validation and quality control

Instrument variability was determined by calculating the median relative standard deviation (RSD) for the internal standards that were added to each sample before injection into the mass spectrometers. Overall process variability was determined by calculating the median RSD for all endogenous metabolites (i.e. non-instrument standards). A master pool was prepared by pooling all the individual dates samples and the samples were aliquoted to formulate 18 client matrix samples, which are technical replicates of the same master pool. One client matrix sample was measured after every sixth individual date sample. The measurements for client matrix samples are provided as Additional file 3.xls.
