# Supplementary material for: A comprehensive metabolomic data set of date palm fruit
Source: Data Brief. 2018 Apr 10;18:1313–21. doi: 10.1016/j.dib.2018.04.012 (PMC5997577; doi:10.1016/j.dib.2018.04.012)
Supplement: Supplementary file 1 — Transparency document [file mmc1.docx]

### Conflict of interest statement

Manuscript title : A Comprehensive Metabolomic Data Set of Date Palm Fruit

The authors whose names are listed immediately below certify that they have NO involvement in any organization or entity with any financial interest or non-financial interest in the materials discussed in this manuscript.

Author names:

Nisha Stephan

Anna Halama

Sweety Mathew

Shahina Hayat

Aditya Bhagwat

Lisa Sara Mathew

Ilham Diboun

Joel Malek

Karsten Suhre
