# Supplementary figures and images for: A comprehensive metabolomic data set of date palm fruit (part 2 of 2)
Source: Data Brief. 2018 Apr 10;18:1313–21. doi: 10.1016/j.dib.2018.04.012 (PMC5997577; doi:10.1016/j.dib.2018.04.012)

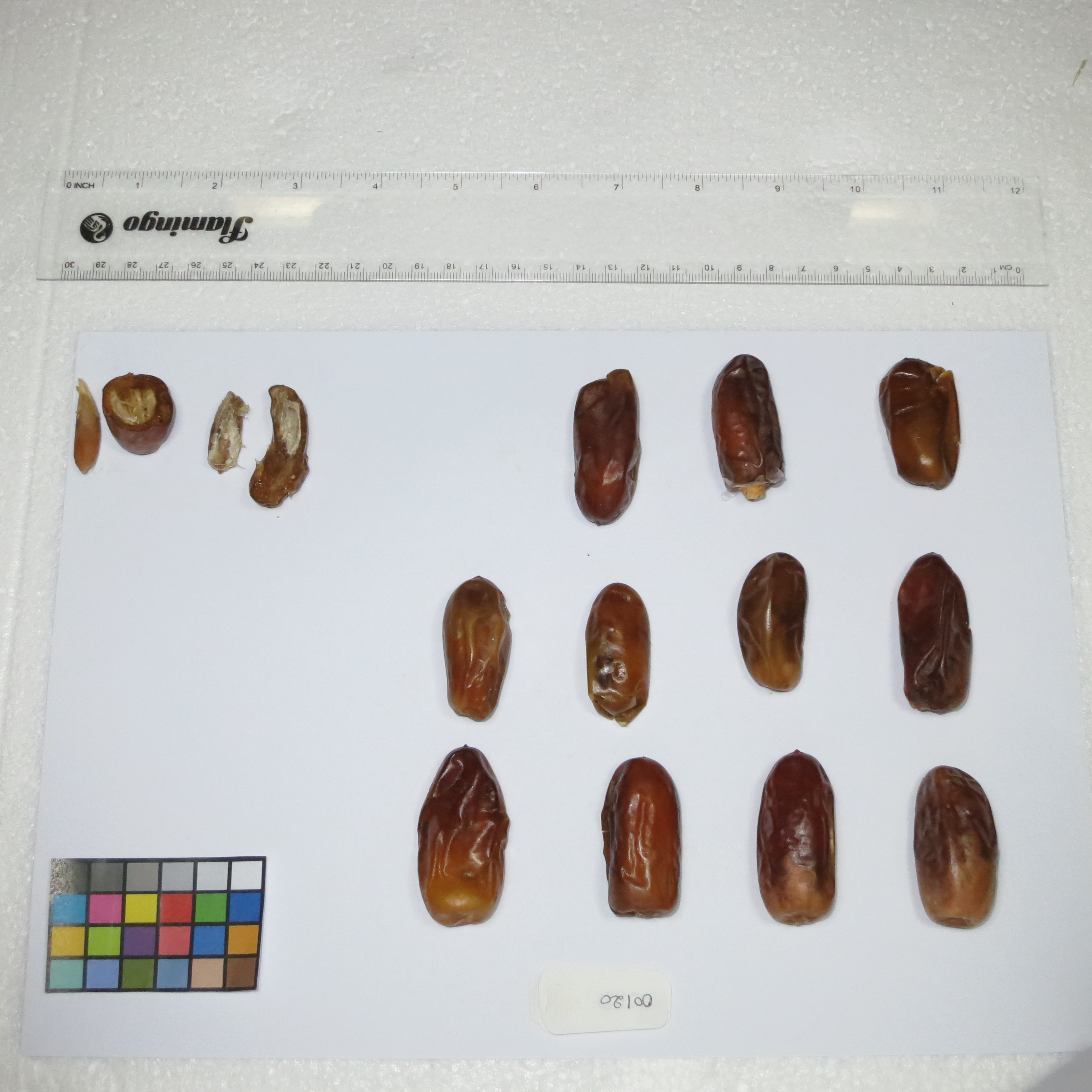

Supplement: Supplementary file 5 — Supplementary material [file mmc5.zip › dates images/00120.JPG]

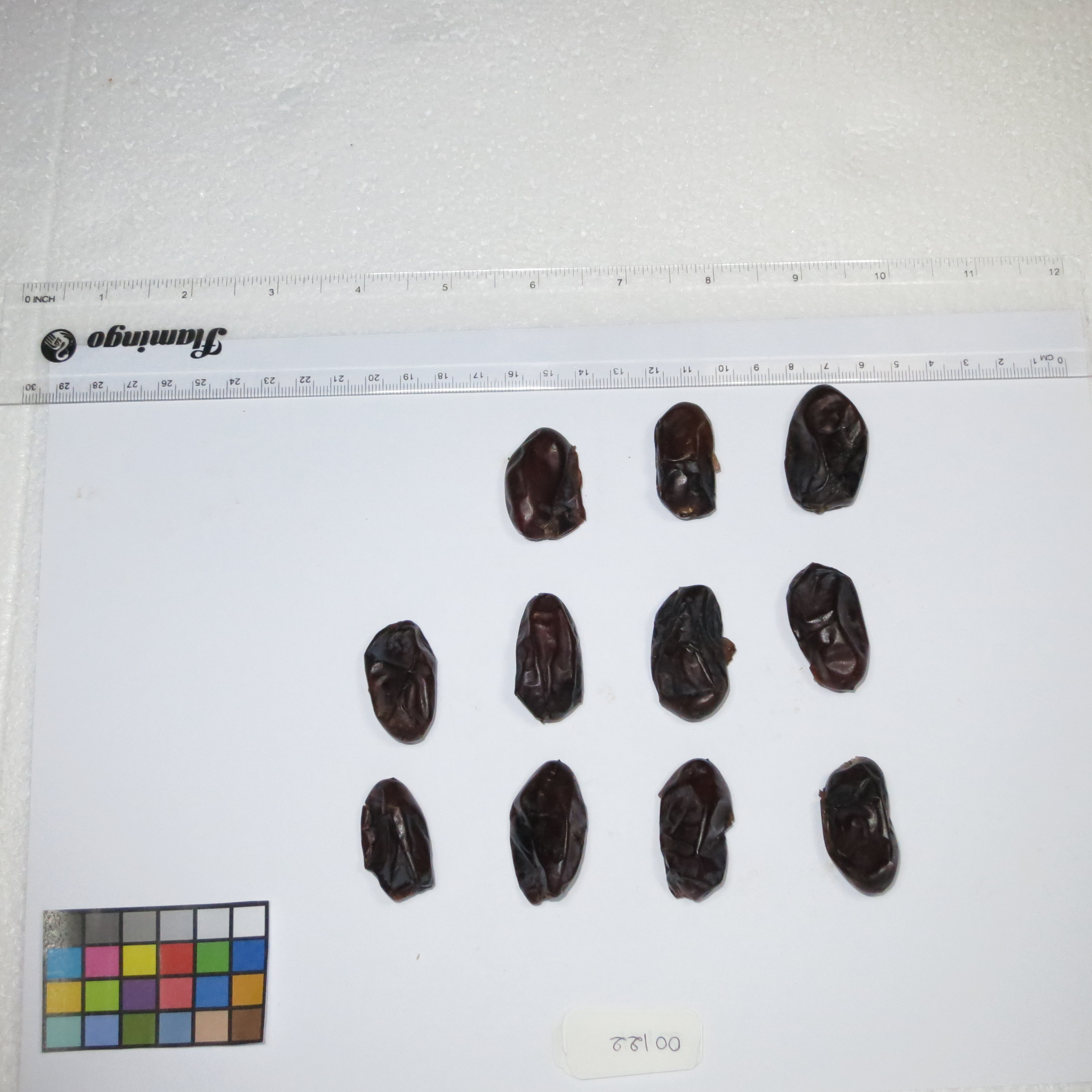

Supplement: Supplementary file 5 — Supplementary material [file mmc5.zip › dates images/00122.JPG]

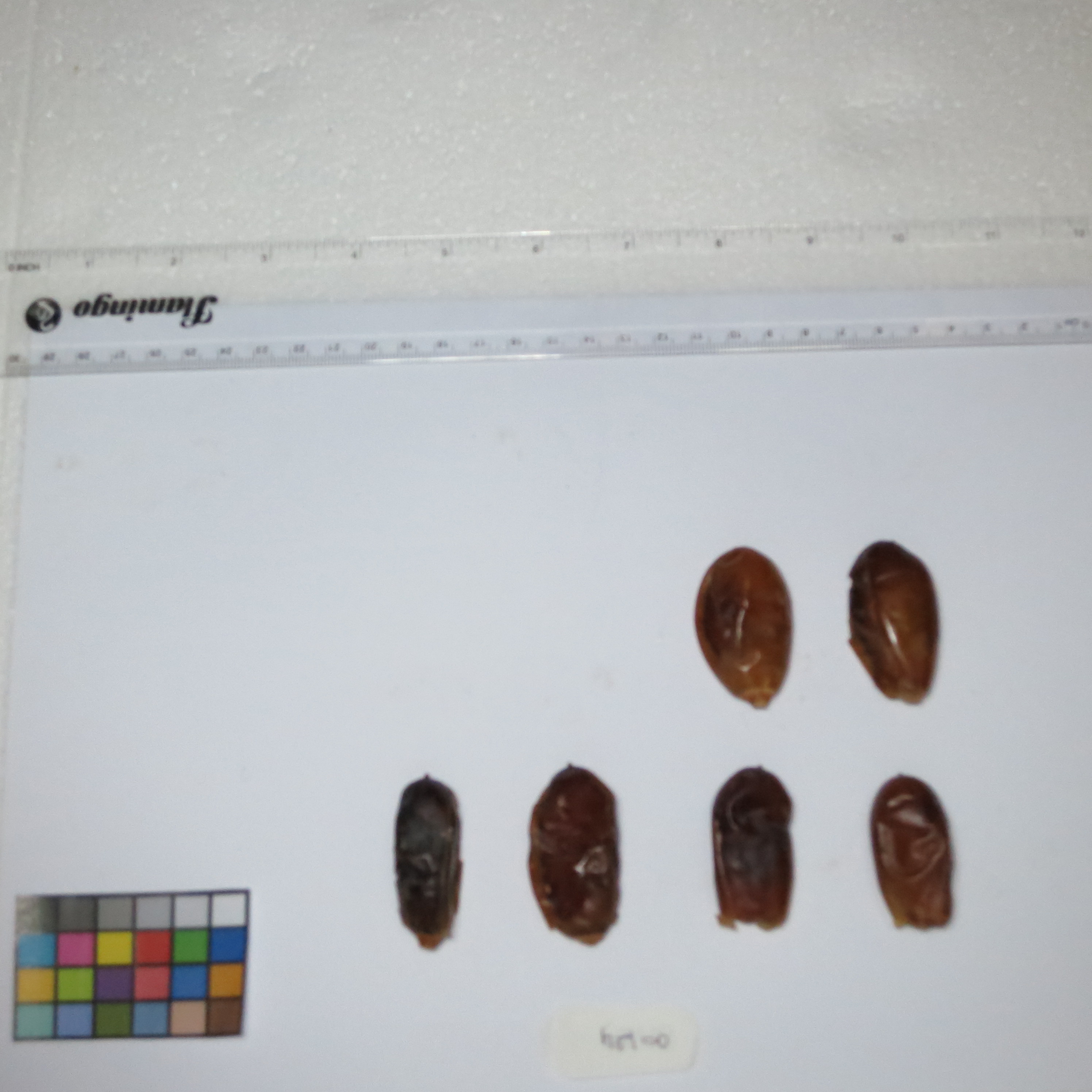

Supplement: Supplementary file 5 — Supplementary material [file mmc5.zip › dates images/00124.JPG]

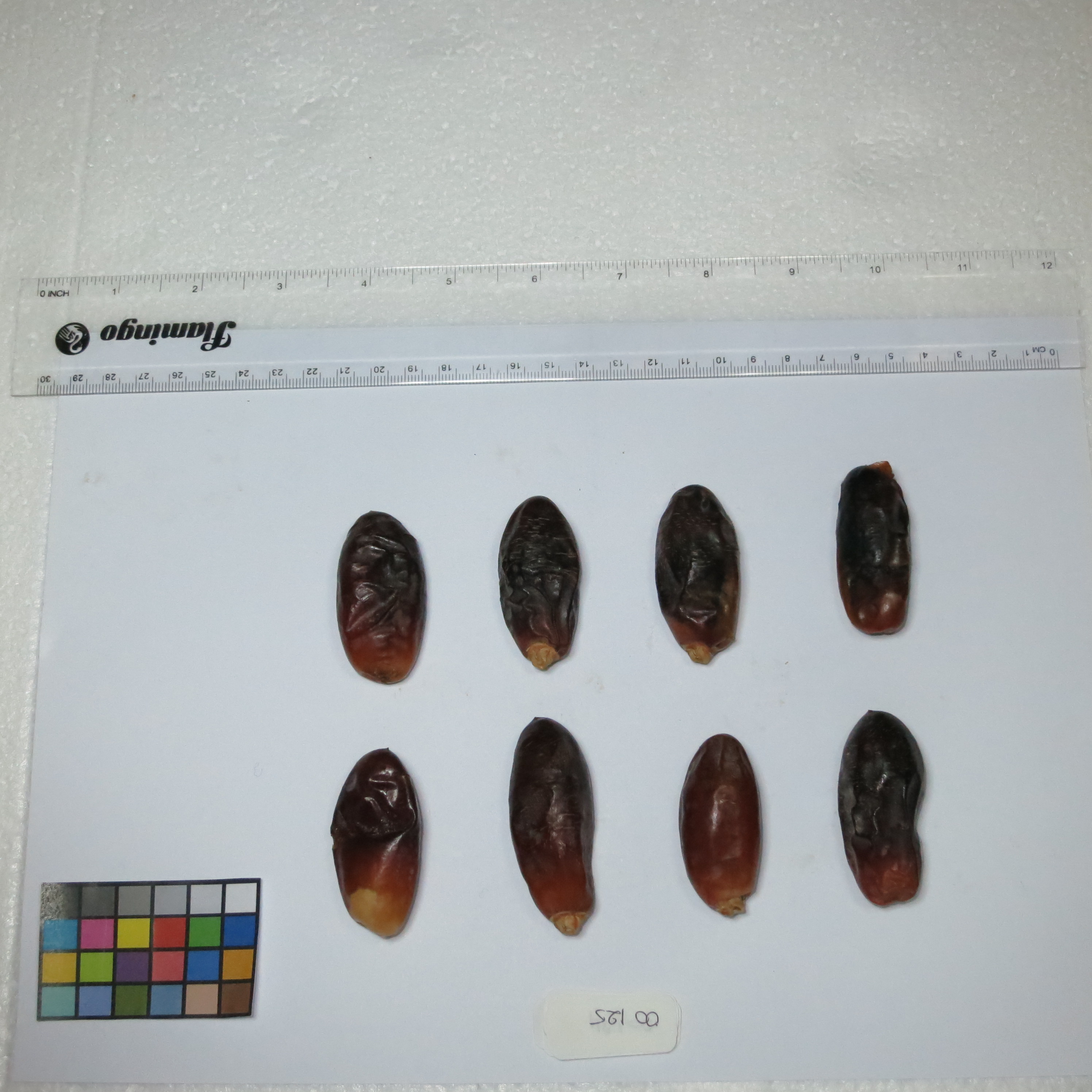

Supplement: Supplementary file 5 — Supplementary material [file mmc5.zip › dates images/00125.JPG]

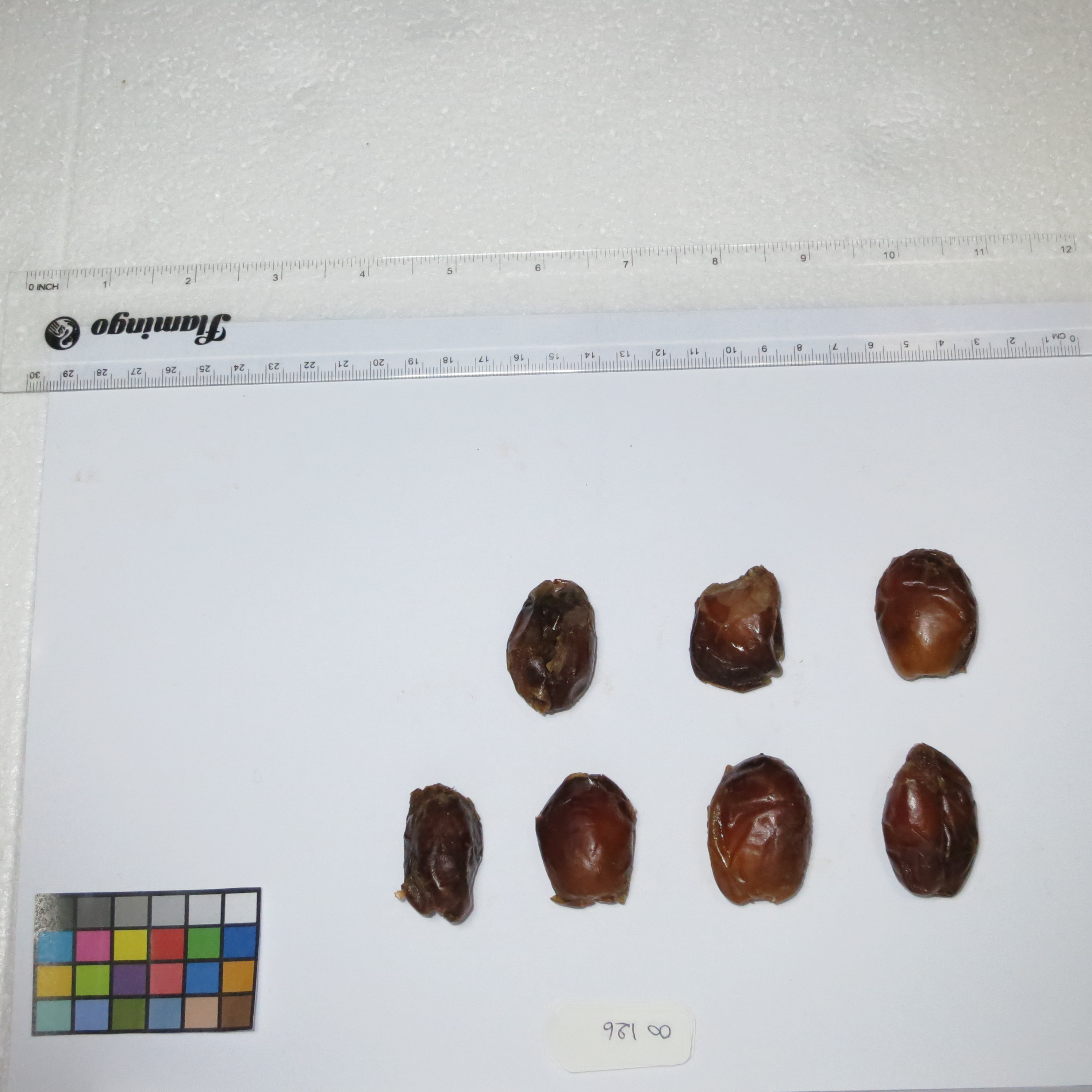

Supplement: Supplementary file 5 — Supplementary material [file mmc5.zip › dates images/00126.JPG]

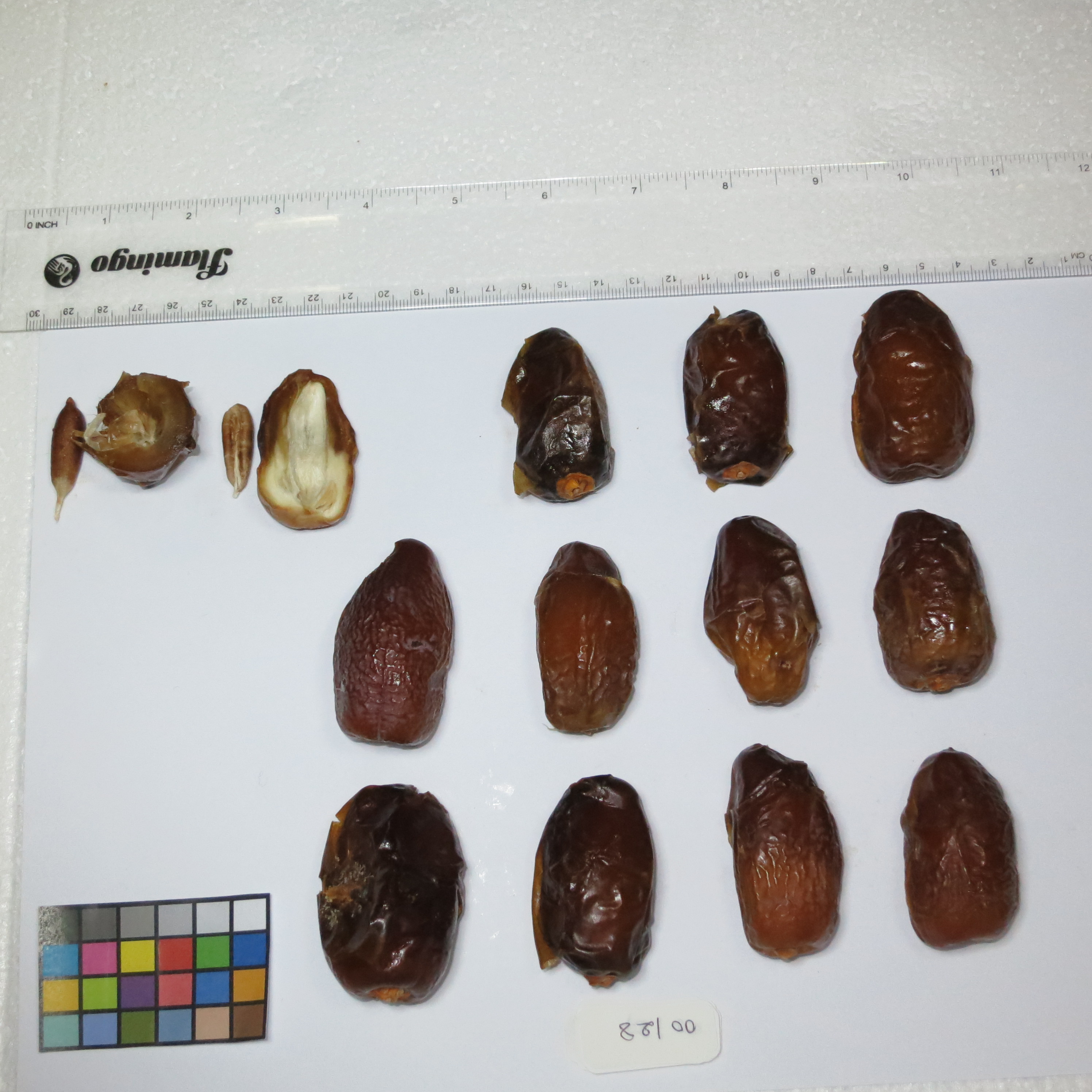

Supplement: Supplementary file 5 — Supplementary material [file mmc5.zip › dates images/00128.JPG]

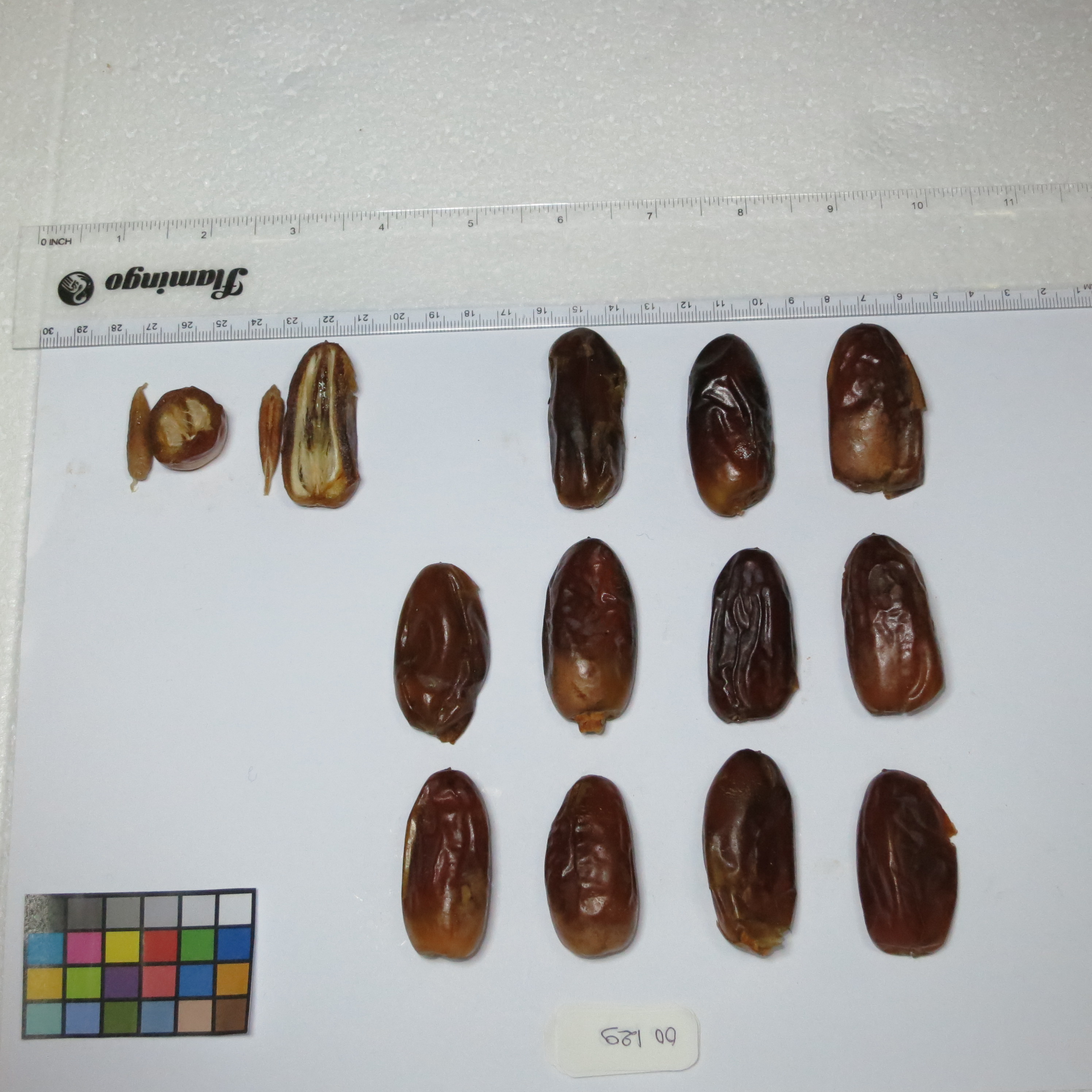

Supplement: Supplementary file 5 — Supplementary material [file mmc5.zip › dates images/00129.JPG]

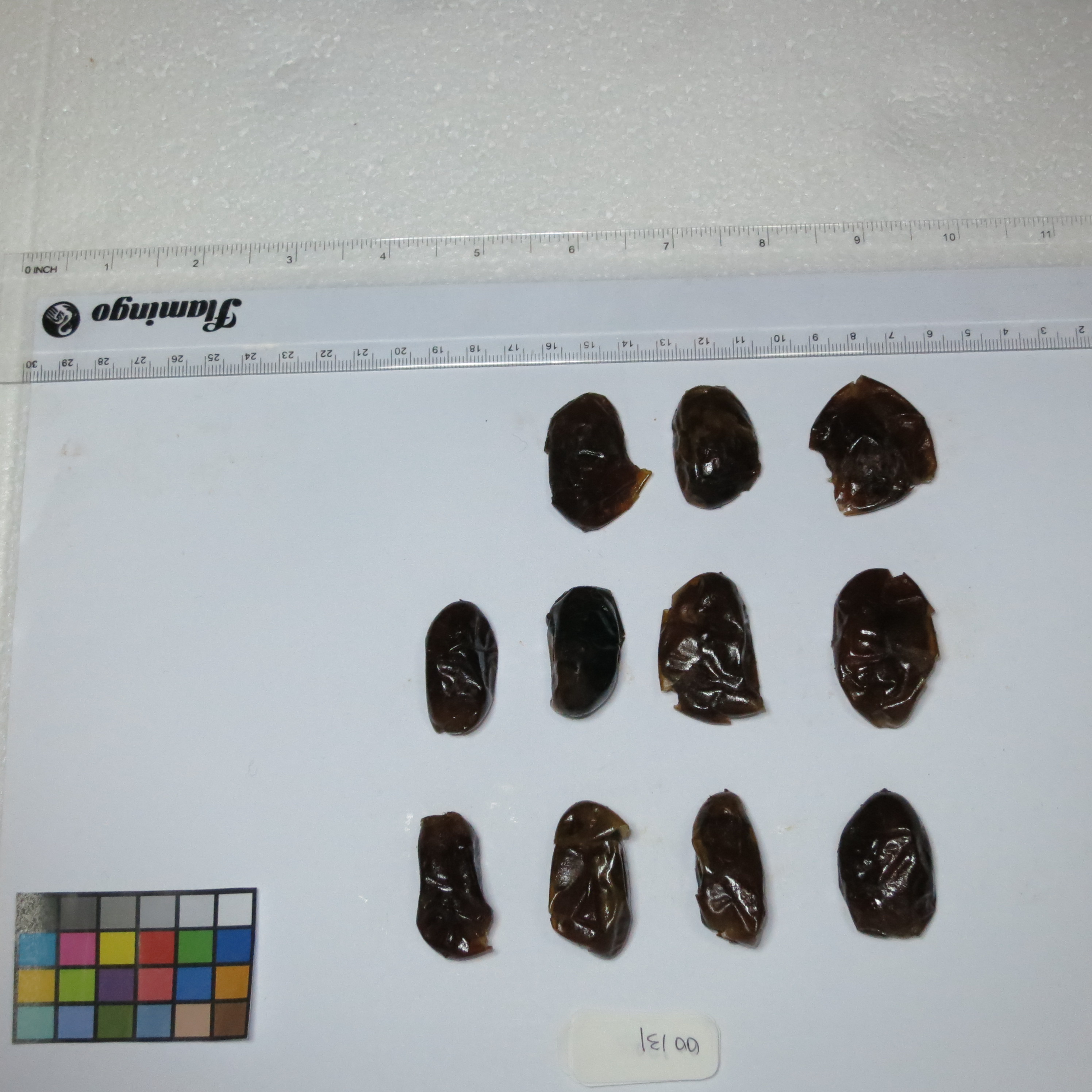

Supplement: Supplementary file 5 — Supplementary material [file mmc5.zip › dates images/00131.JPG]

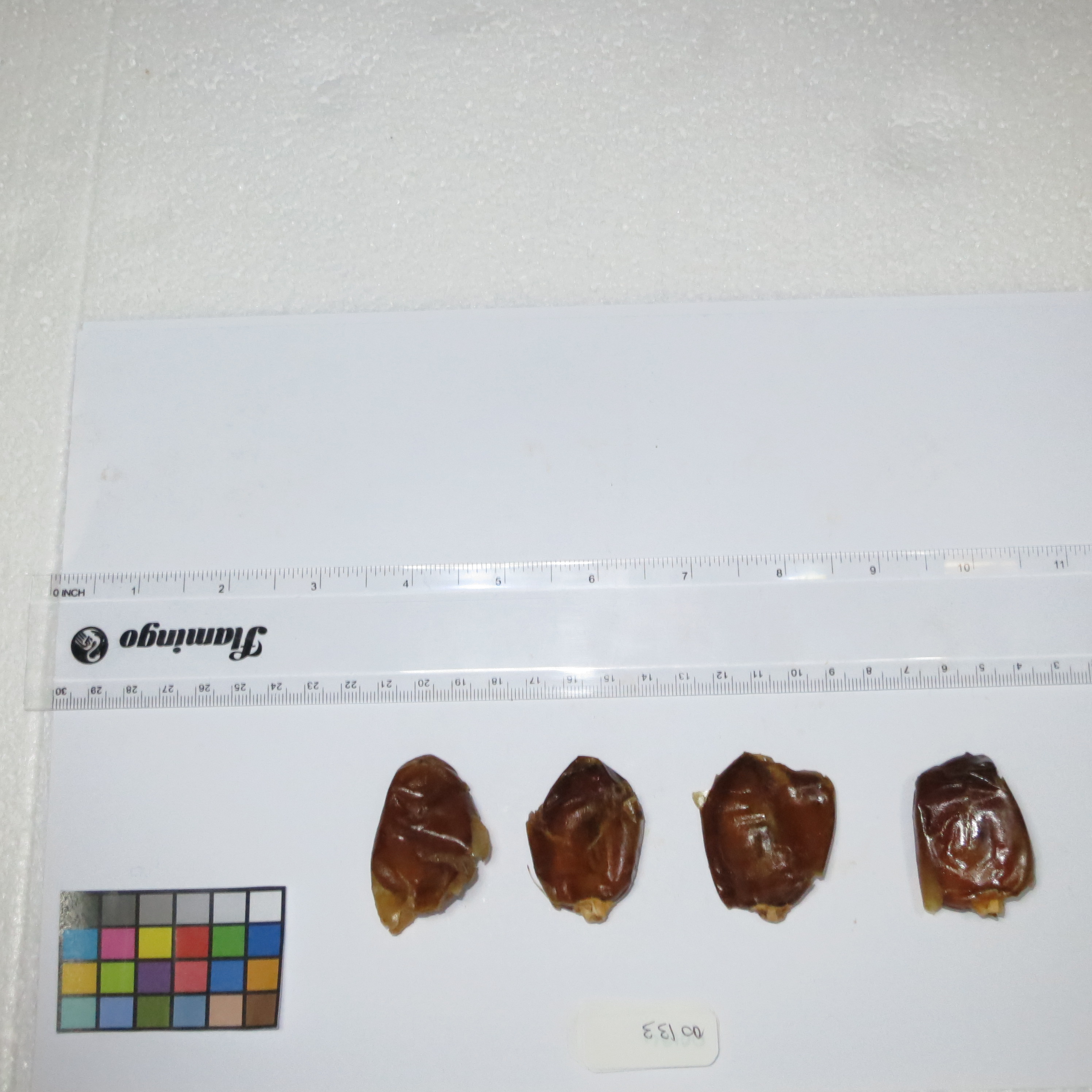

Supplement: Supplementary file 5 — Supplementary material [file mmc5.zip › dates images/00133.JPG]

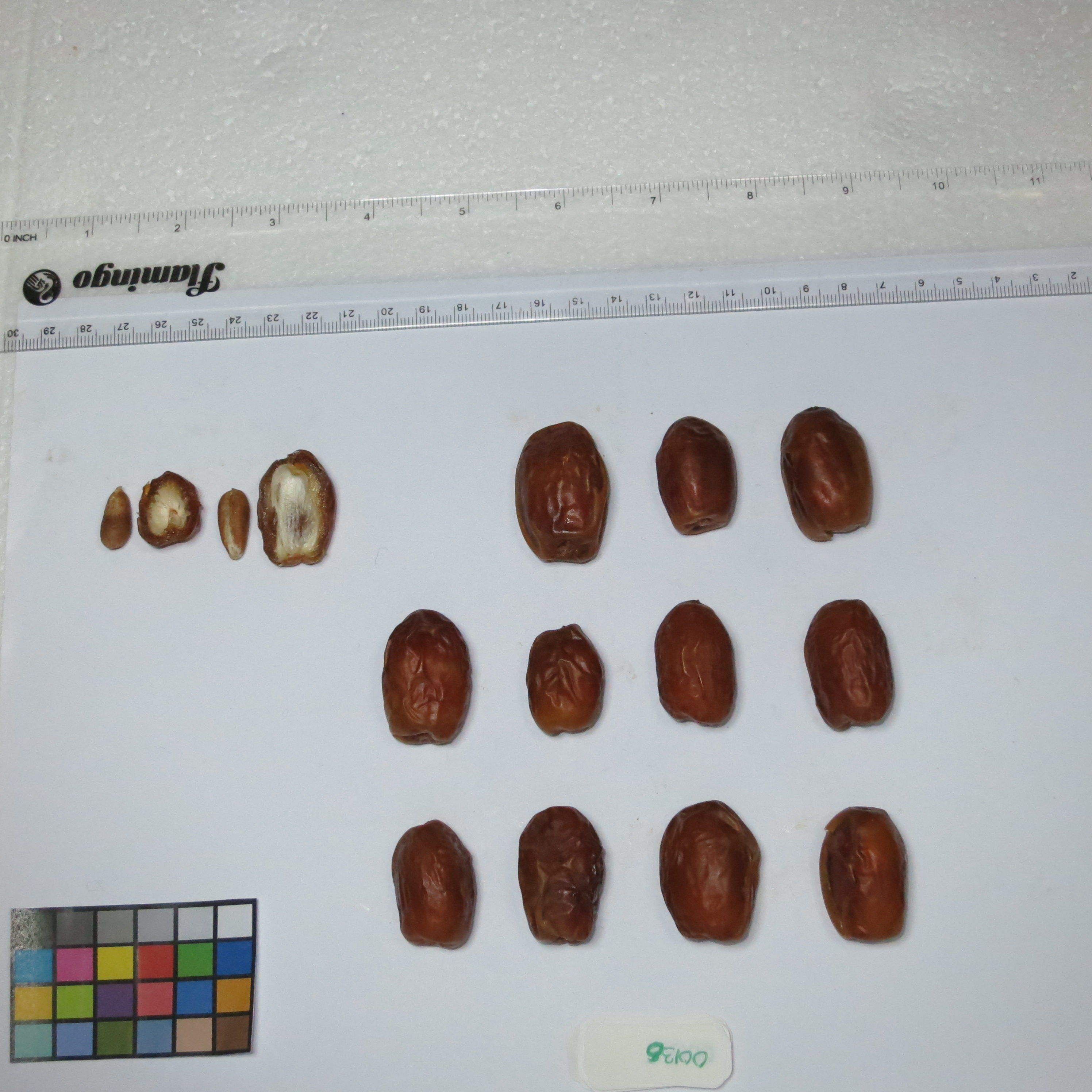

Supplement: Supplementary file 5 — Supplementary material [file mmc5.zip › dates images/00136.JPG]

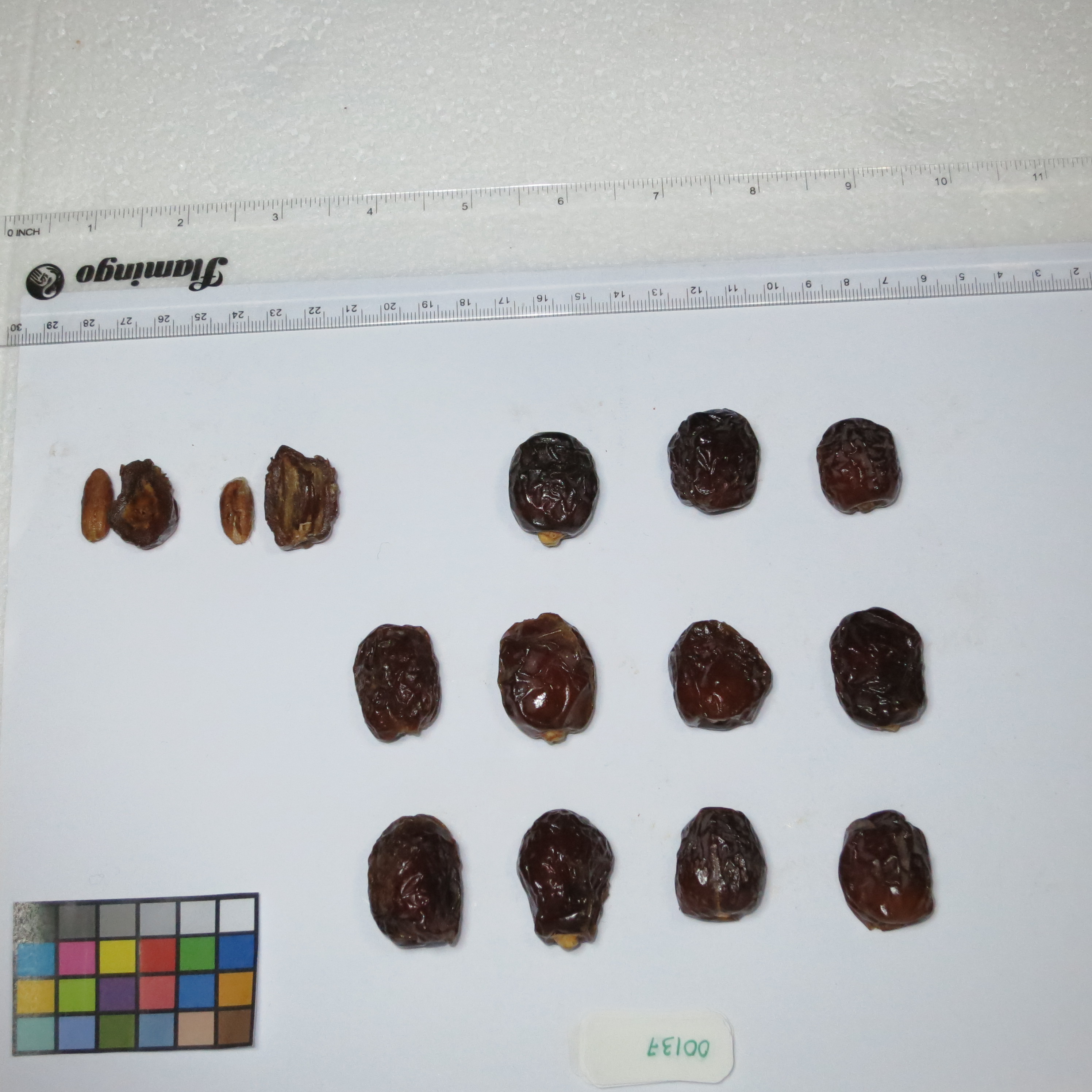

Supplement: Supplementary file 5 — Supplementary material [file mmc5.zip › dates images/00137.JPG]

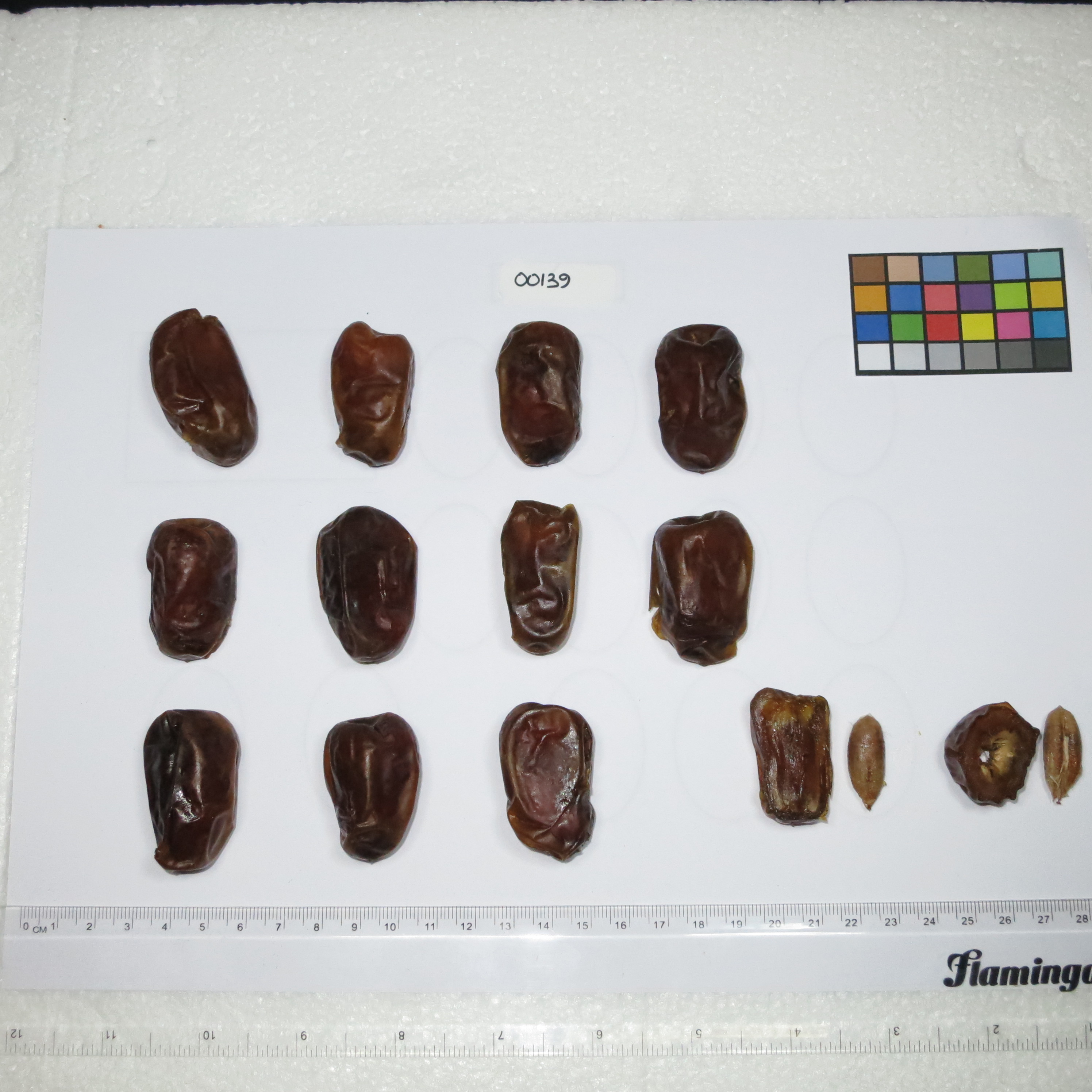

Supplement: Supplementary file 5 — Supplementary material [file mmc5.zip › dates images/00139.JPG]

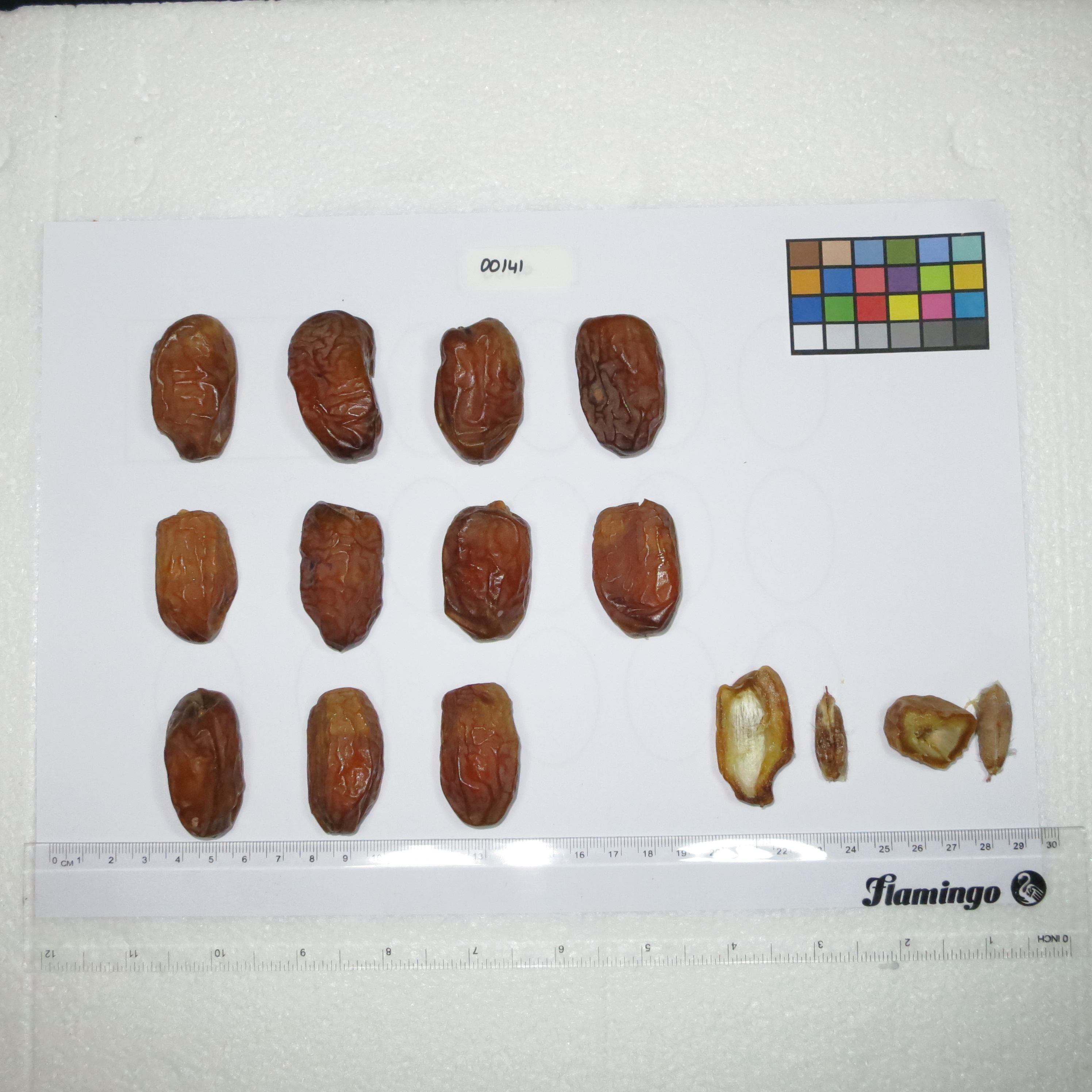

Supplement: Supplementary file 5 — Supplementary material [file mmc5.zip › dates images/00141.JPG]

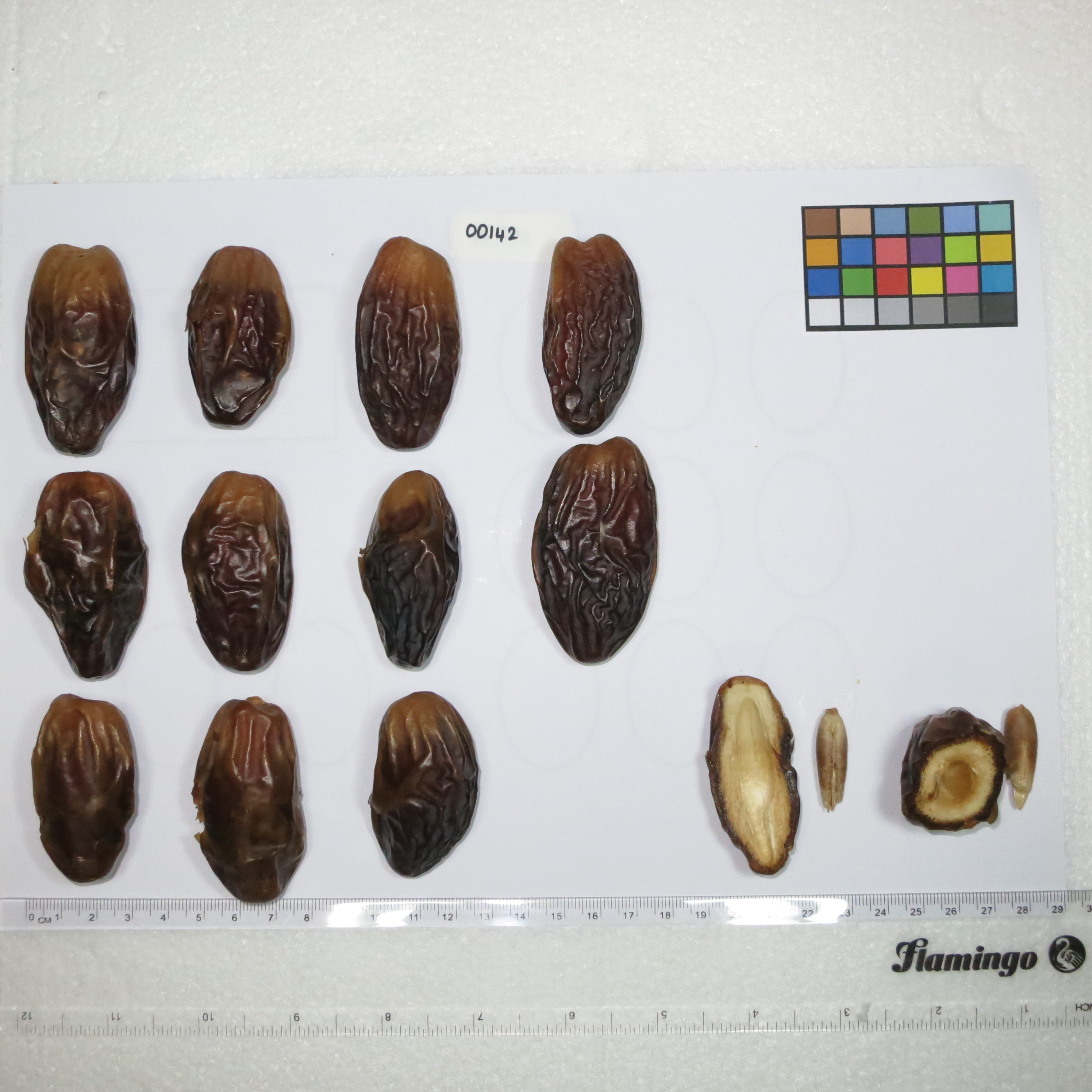

Supplement: Supplementary file 5 — Supplementary material [file mmc5.zip › dates images/00142.JPG]

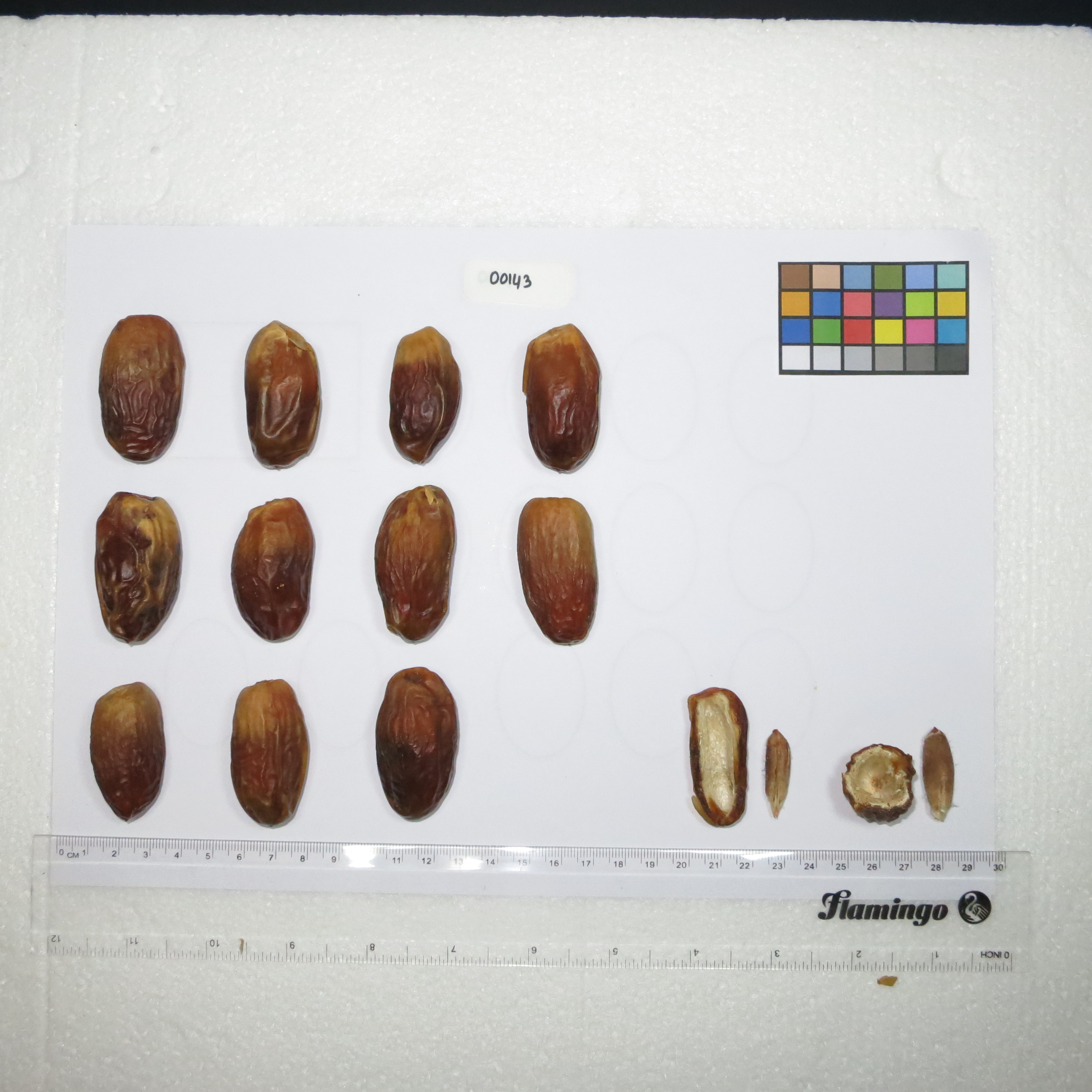

Supplement: Supplementary file 5 — Supplementary material [file mmc5.zip › dates images/00143.JPG]

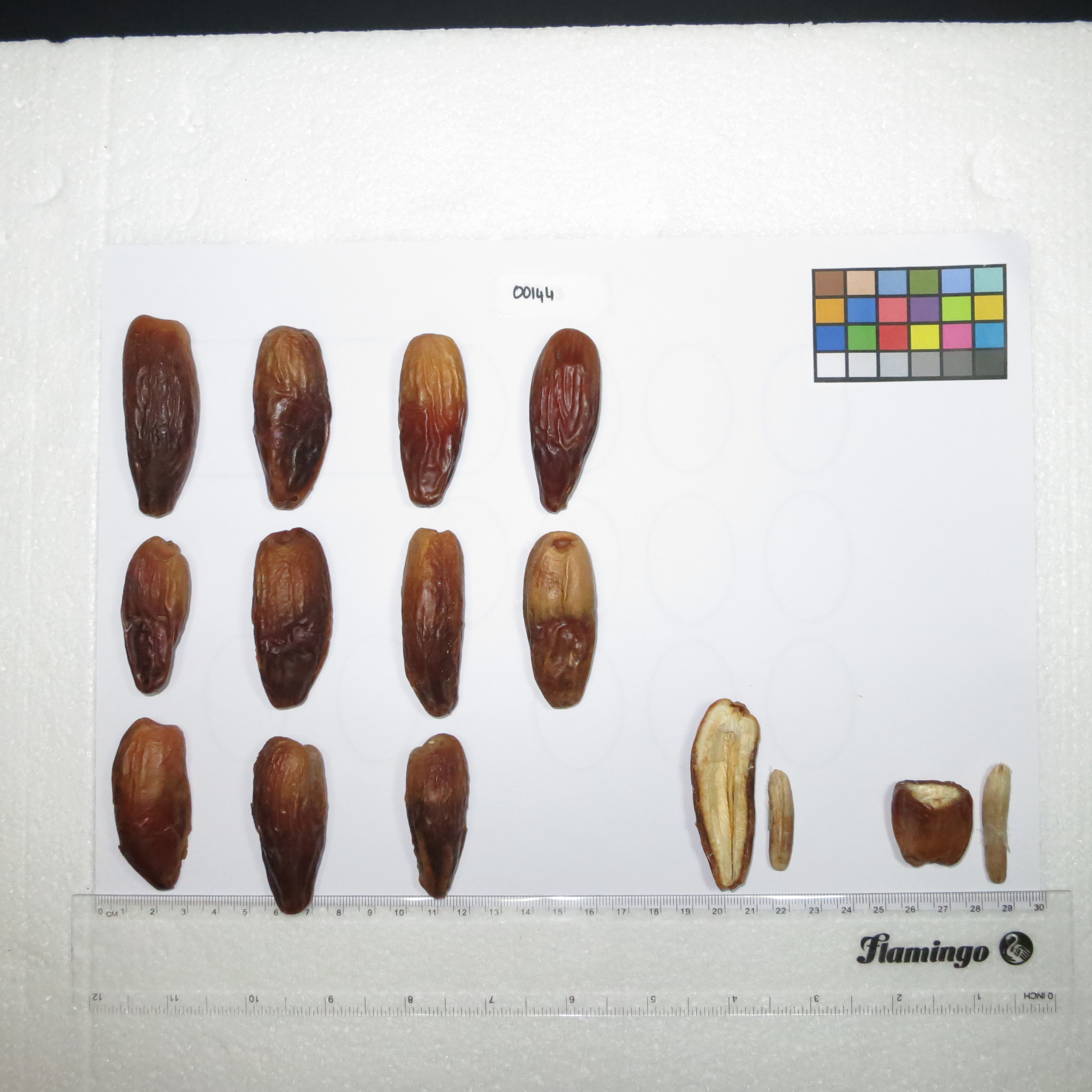

Supplement: Supplementary file 5 — Supplementary material [file mmc5.zip › dates images/00144.JPG]

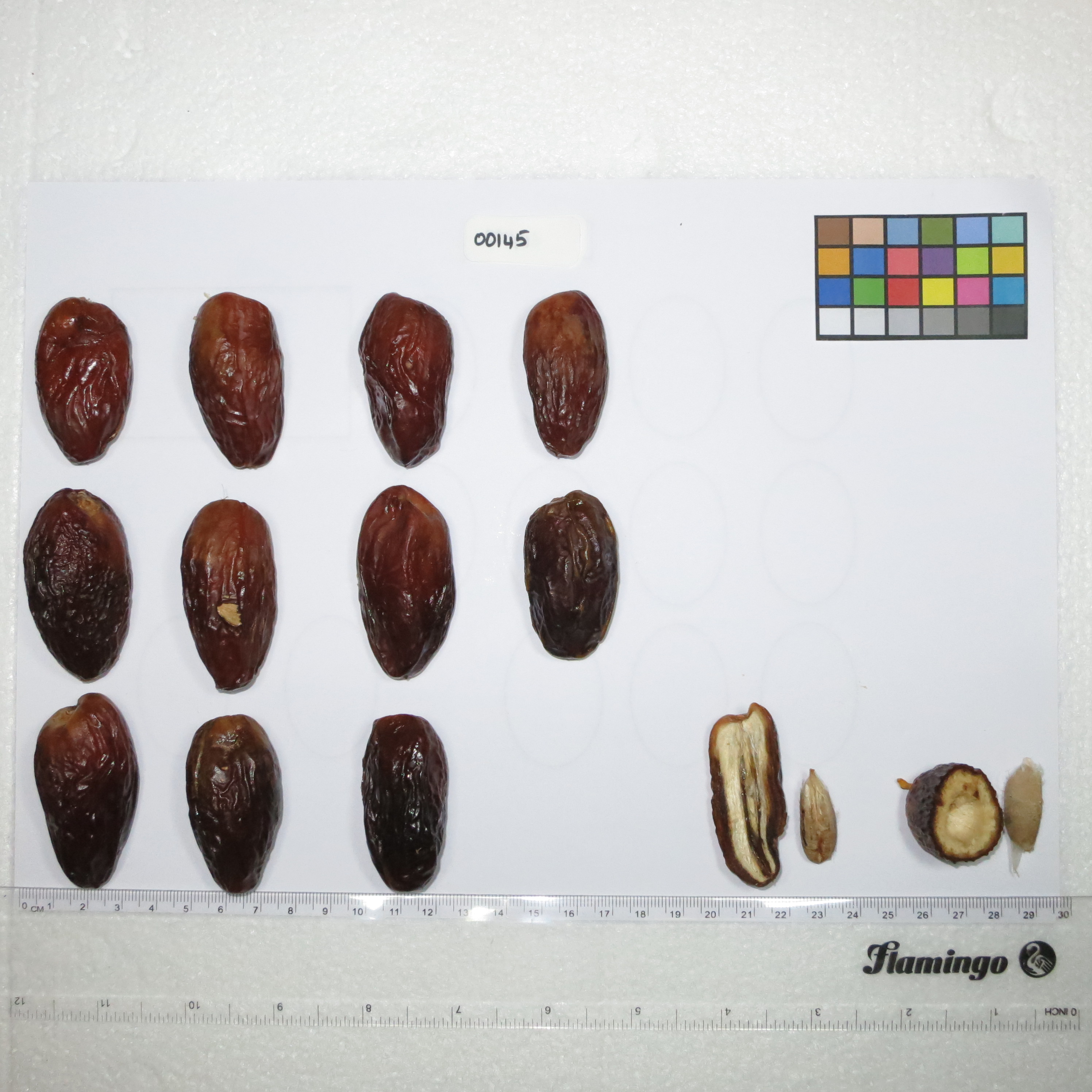

Supplement: Supplementary file 5 — Supplementary material [file mmc5.zip › dates images/00145.JPG]

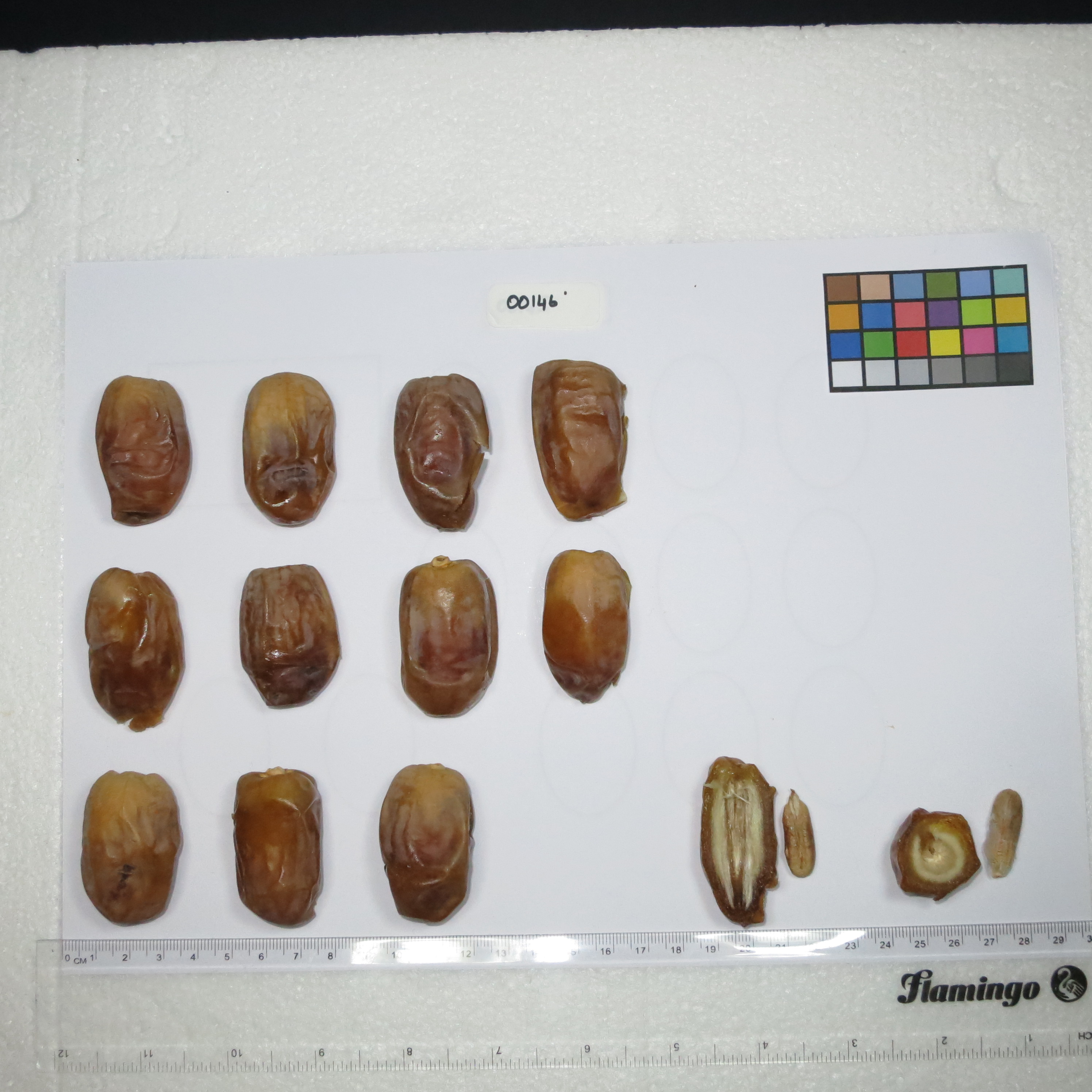

Supplement: Supplementary file 5 — Supplementary material [file mmc5.zip › dates images/00146.JPG]

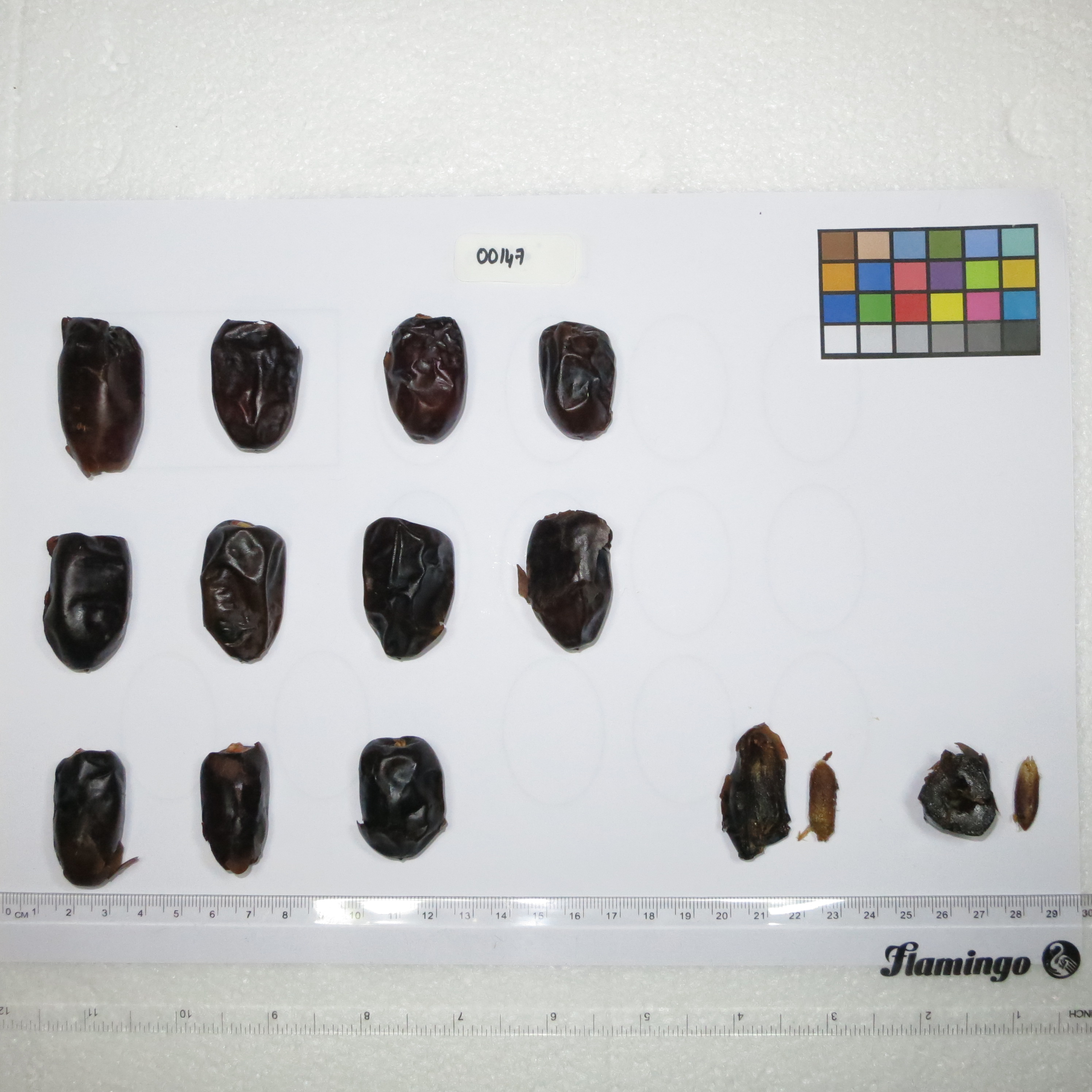

Supplement: Supplementary file 5 — Supplementary material [file mmc5.zip › dates images/00147.JPG]

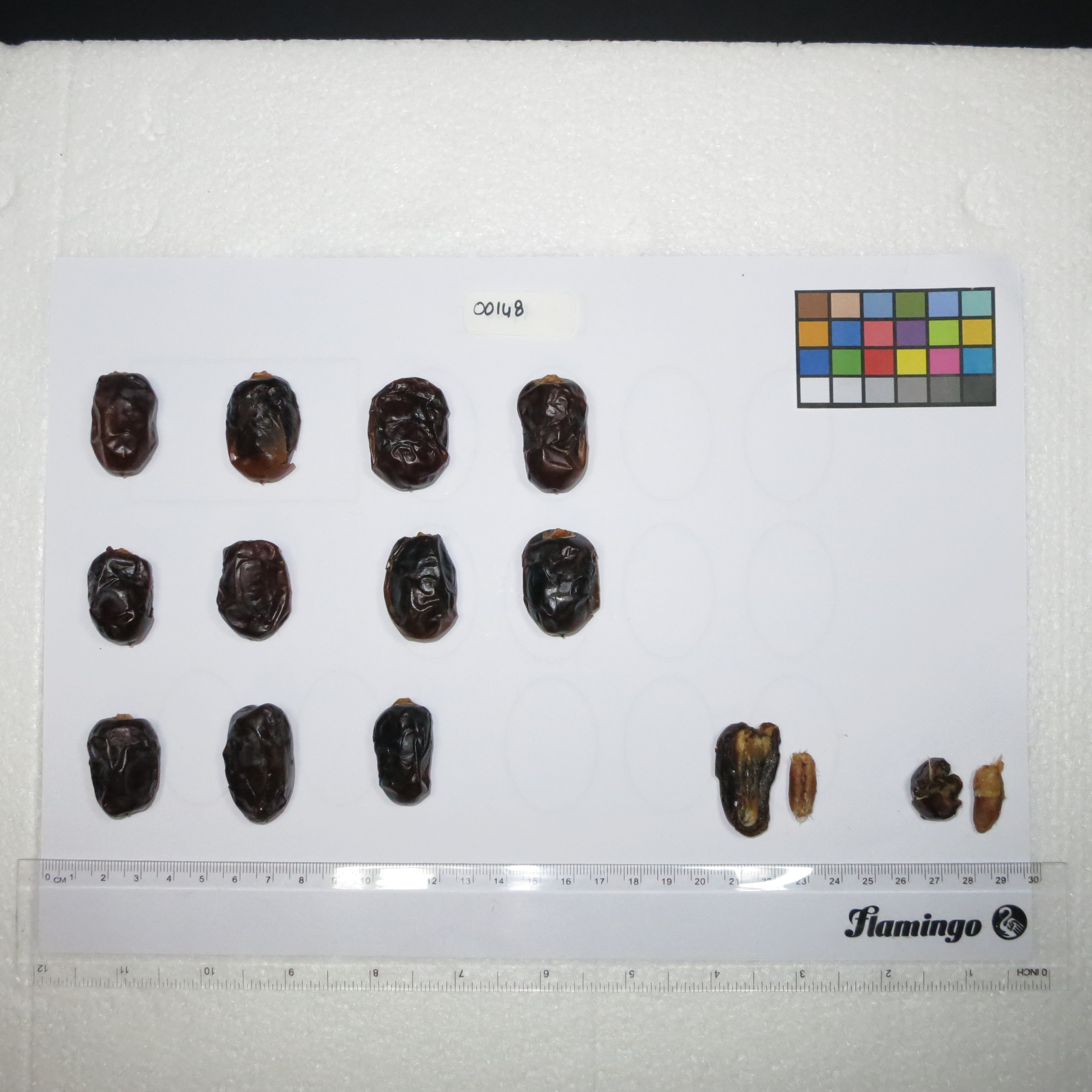

Supplement: Supplementary file 5 — Supplementary material [file mmc5.zip › dates images/00148.JPG]

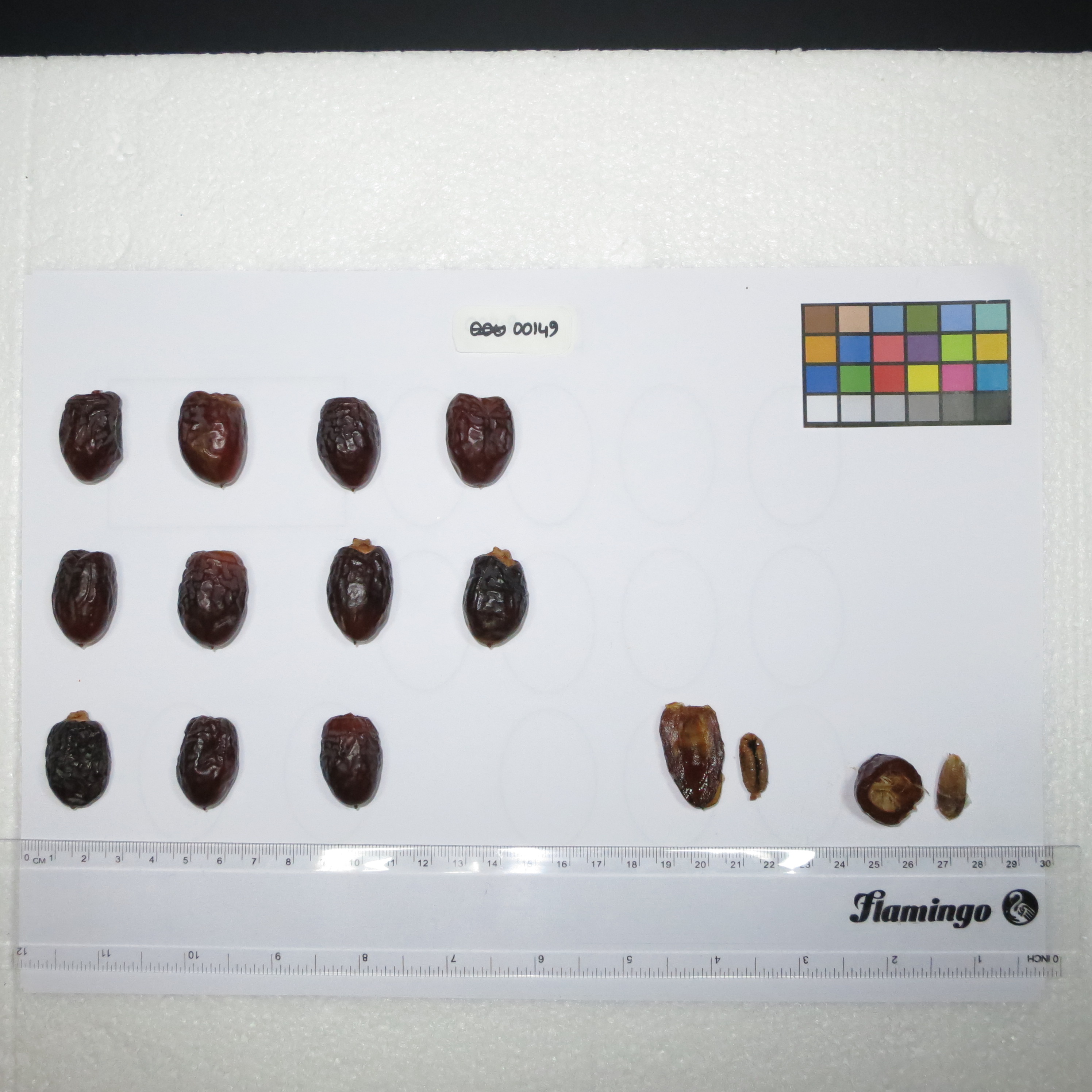

Supplement: Supplementary file 5 — Supplementary material [file mmc5.zip › dates images/00149.JPG]

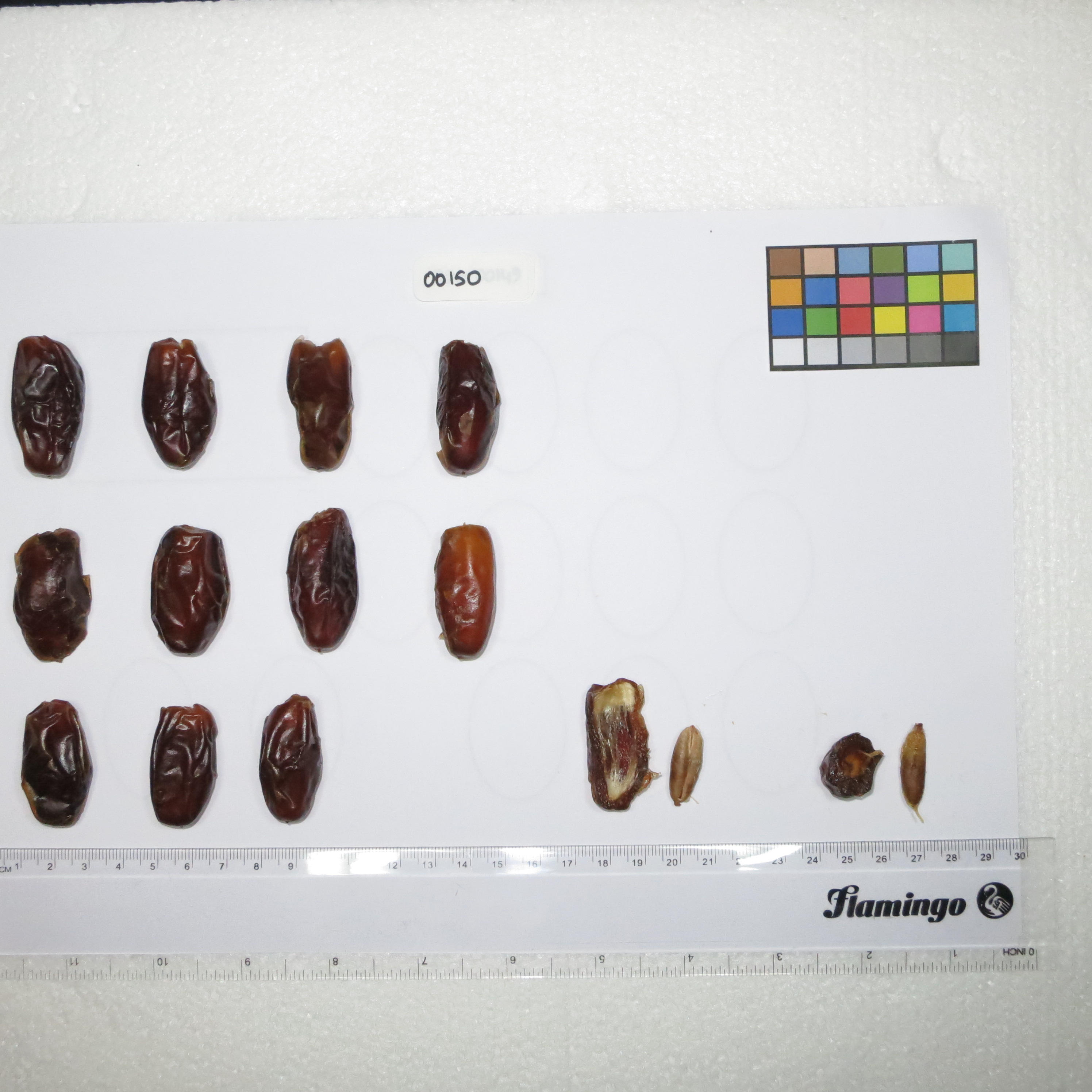

Supplement: Supplementary file 5 — Supplementary material [file mmc5.zip › dates images/00150.JPG]

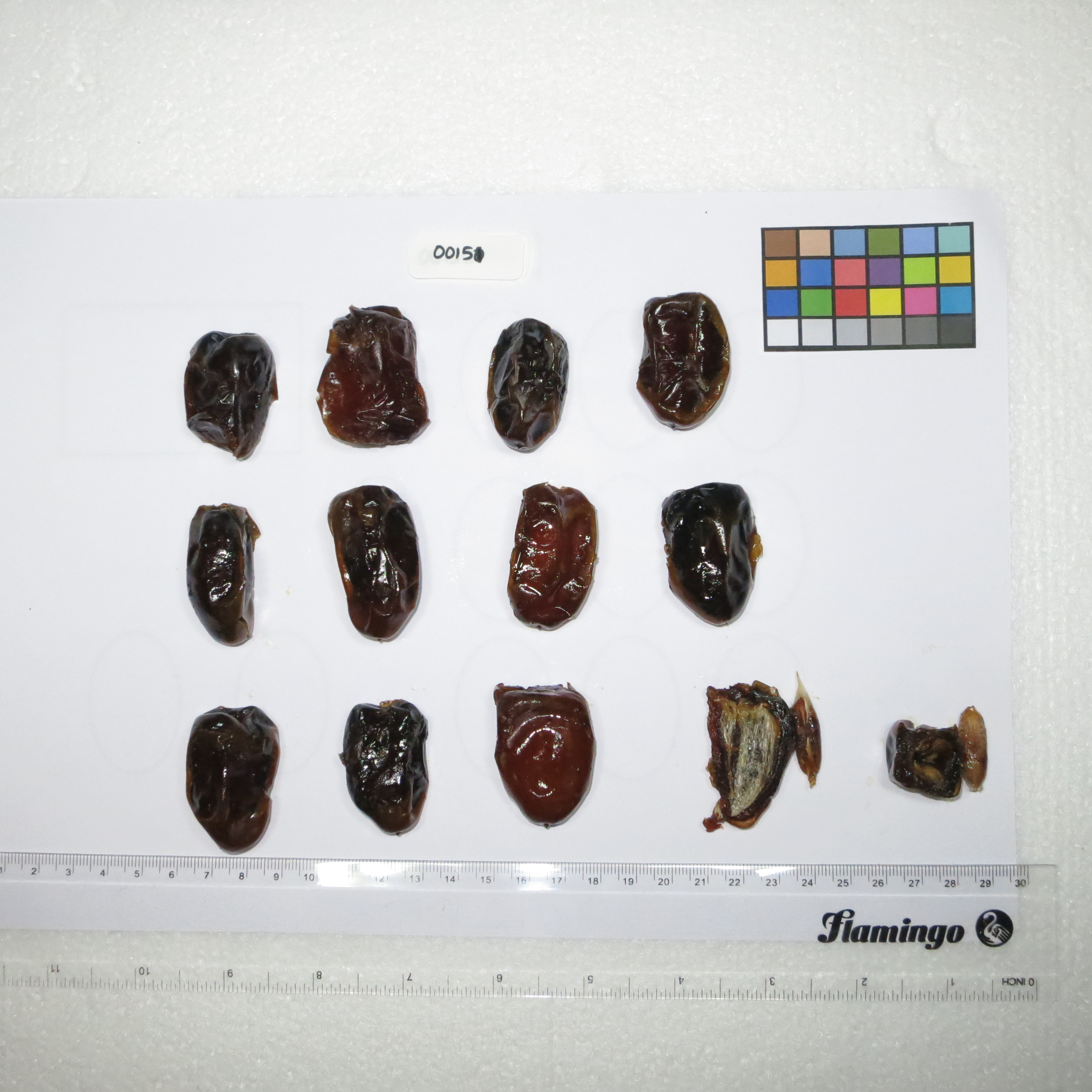

Supplement: Supplementary file 5 — Supplementary material [file mmc5.zip › dates images/00151.JPG]
