# Supplementary figures and images for: A comprehensive metabolomic data set of date palm fruit (part 1 of 2)
Source: Data Brief. 2018 Apr 10;18:1313–21. doi: 10.1016/j.dib.2018.04.012 (PMC5997577; doi:10.1016/j.dib.2018.04.012)

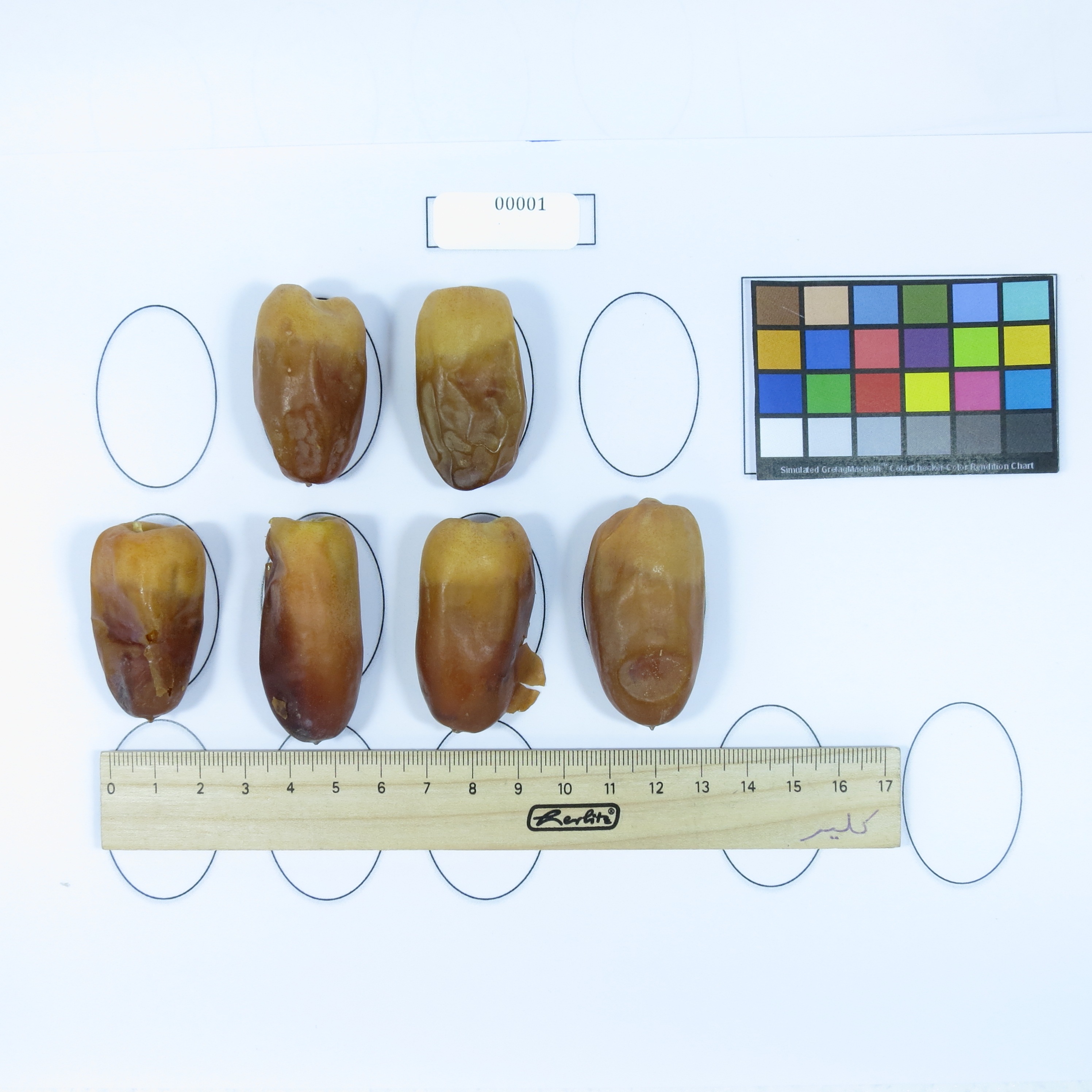

Supplement: Supplementary file 5 — Supplementary material [file mmc5.zip › dates images/00001.JPG]

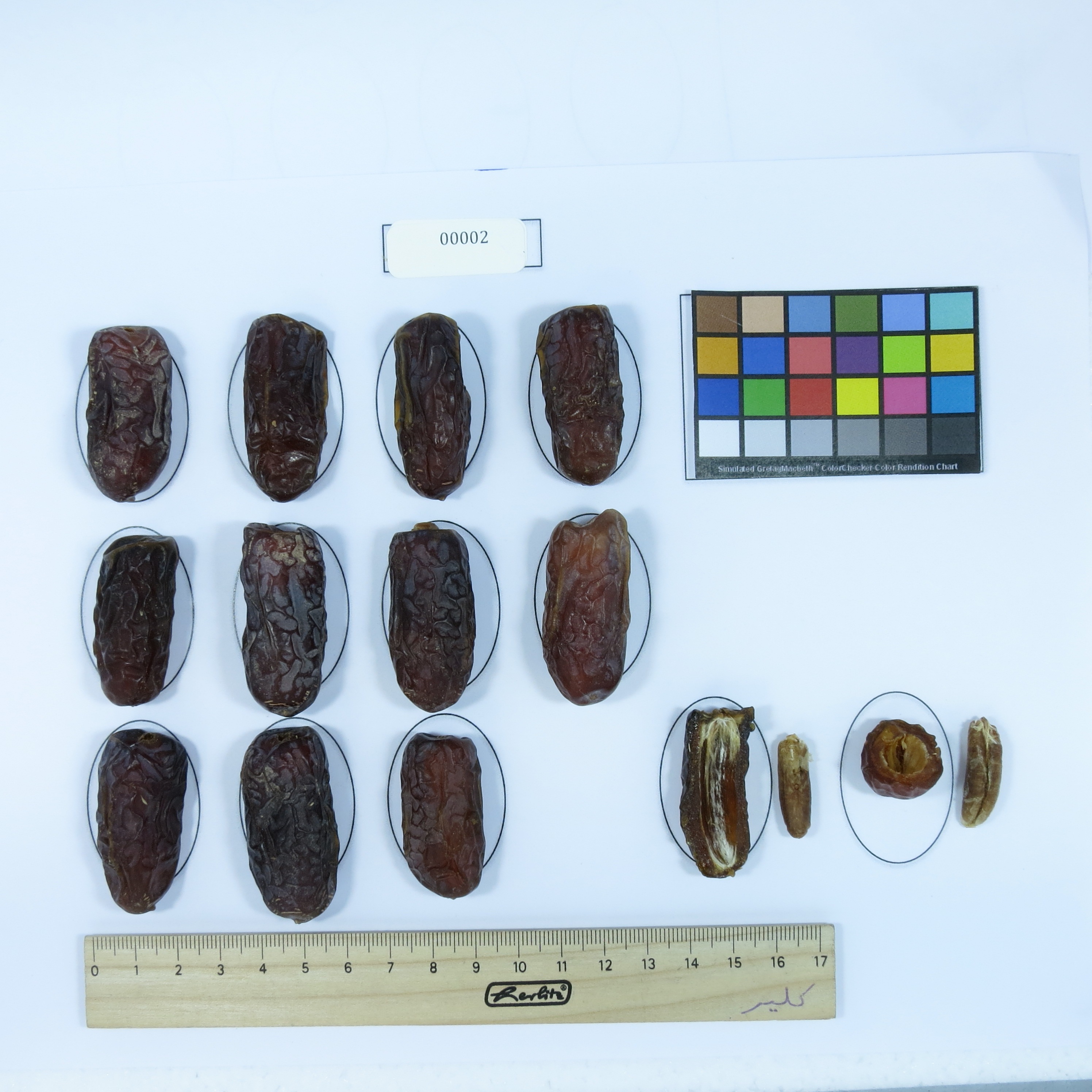

Supplement: Supplementary file 5 — Supplementary material [file mmc5.zip › dates images/00002.JPG]

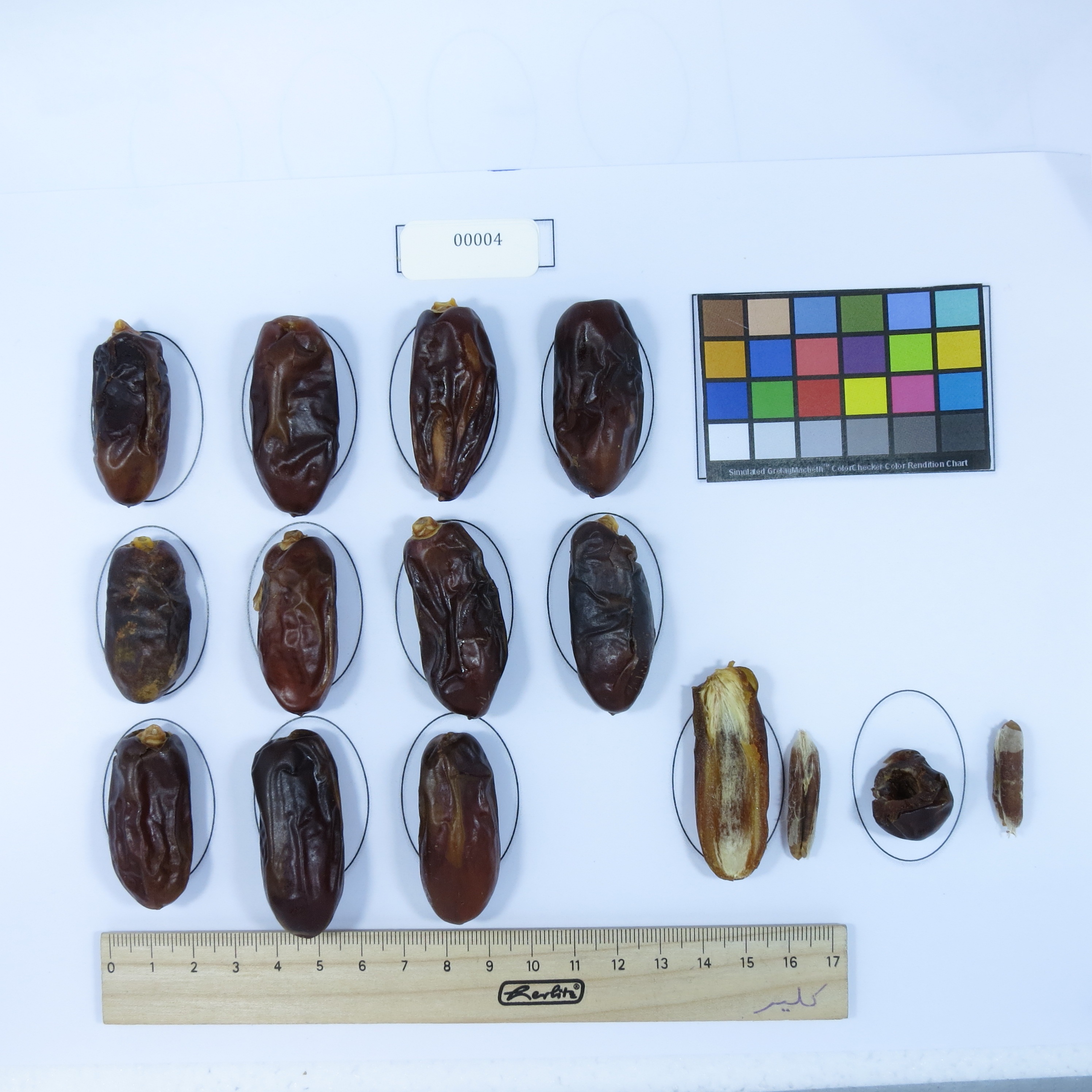

Supplement: Supplementary file 5 — Supplementary material [file mmc5.zip › dates images/00004.JPG]

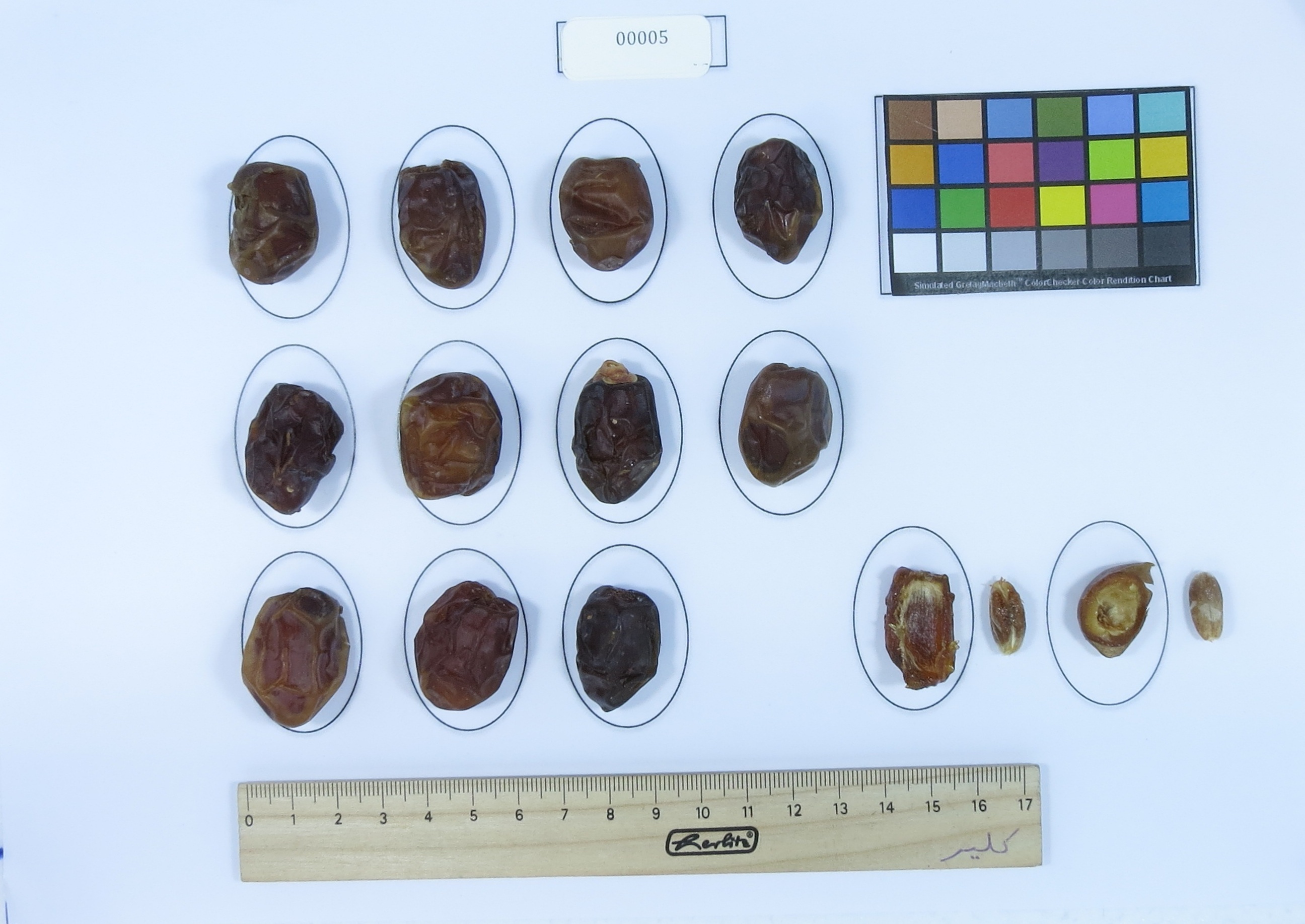

Supplement: Supplementary file 5 — Supplementary material [file mmc5.zip › dates images/00005.JPG]

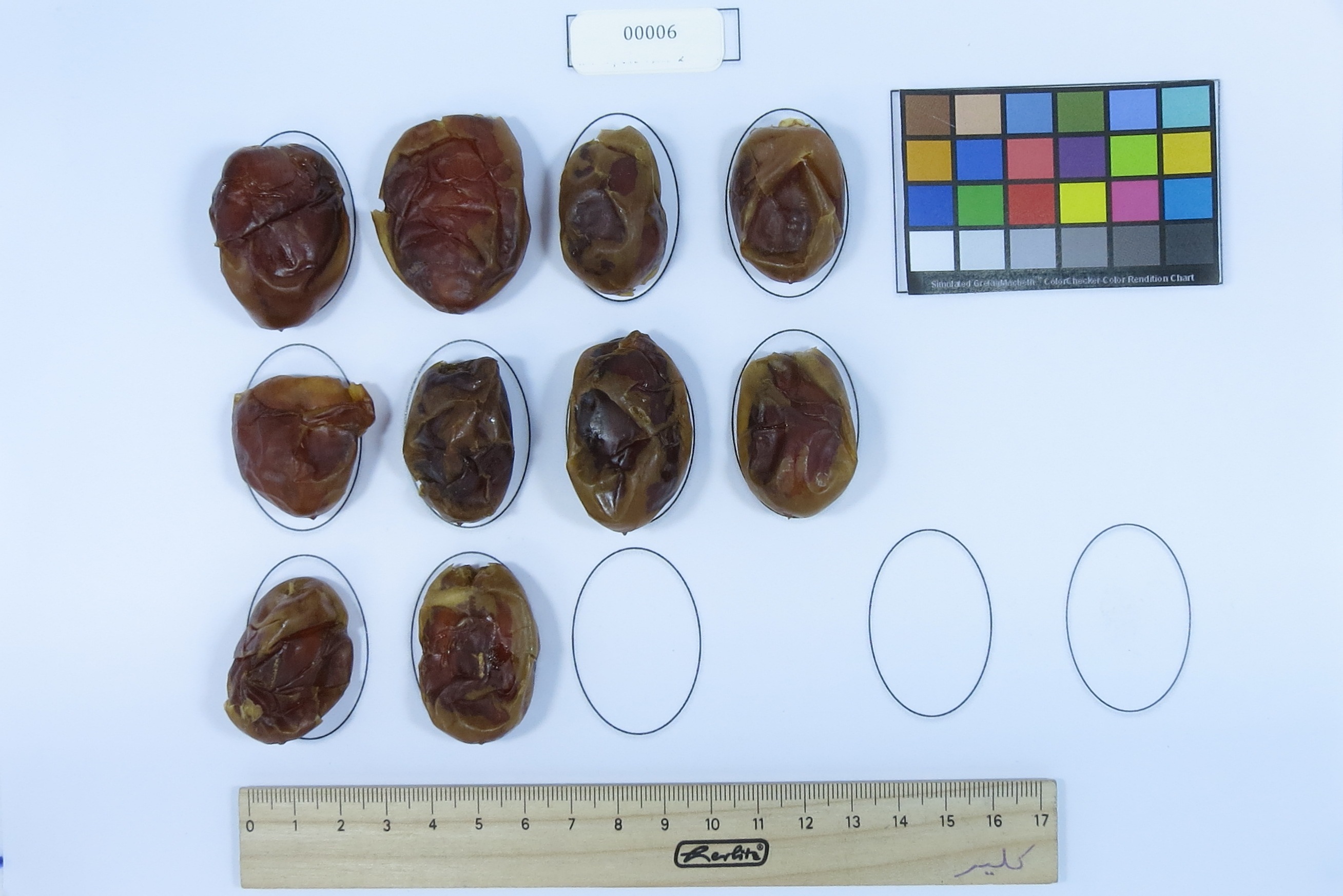

Supplement: Supplementary file 5 — Supplementary material [file mmc5.zip › dates images/00006.JPG]

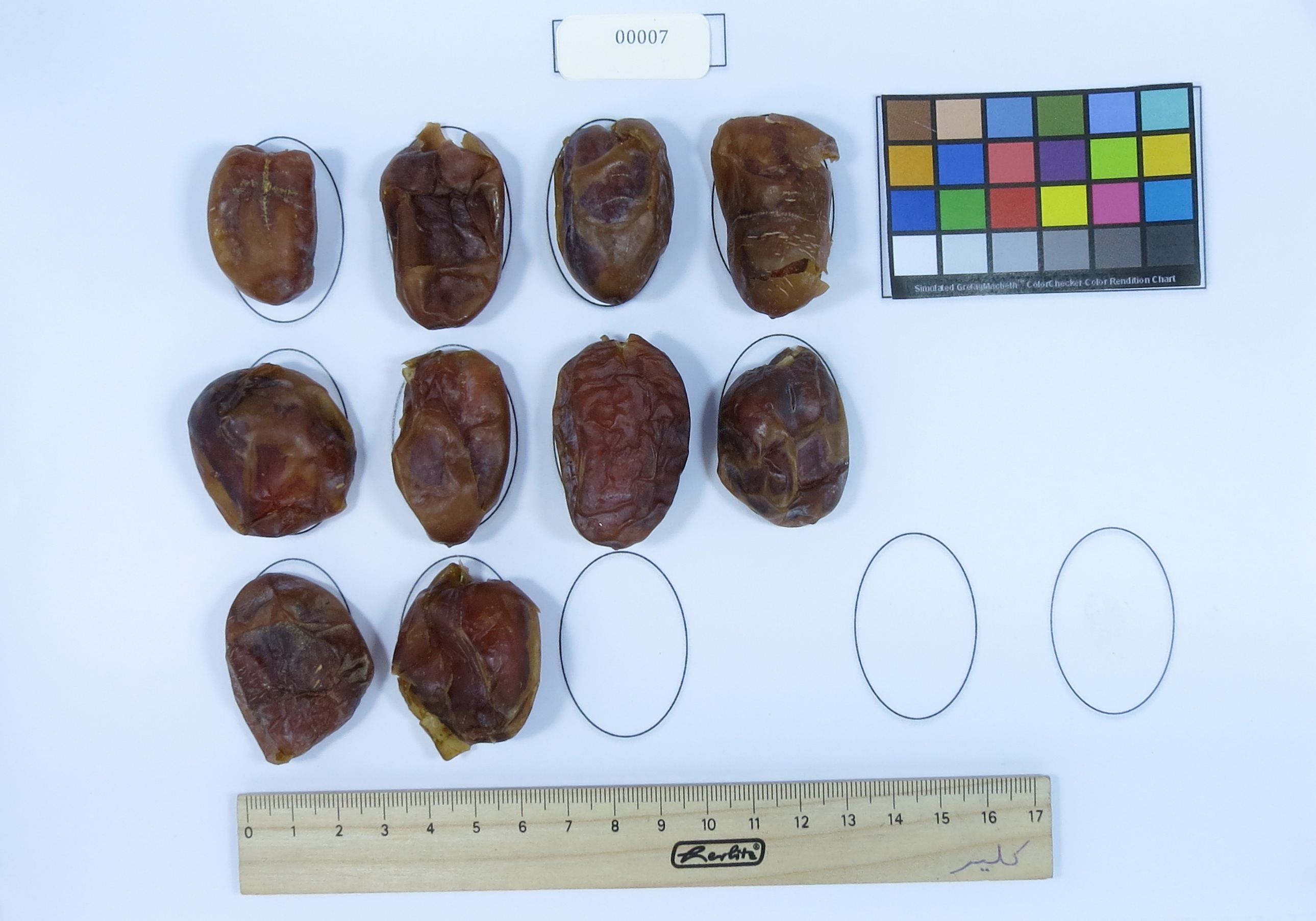

Supplement: Supplementary file 5 — Supplementary material [file mmc5.zip › dates images/00007.JPG]

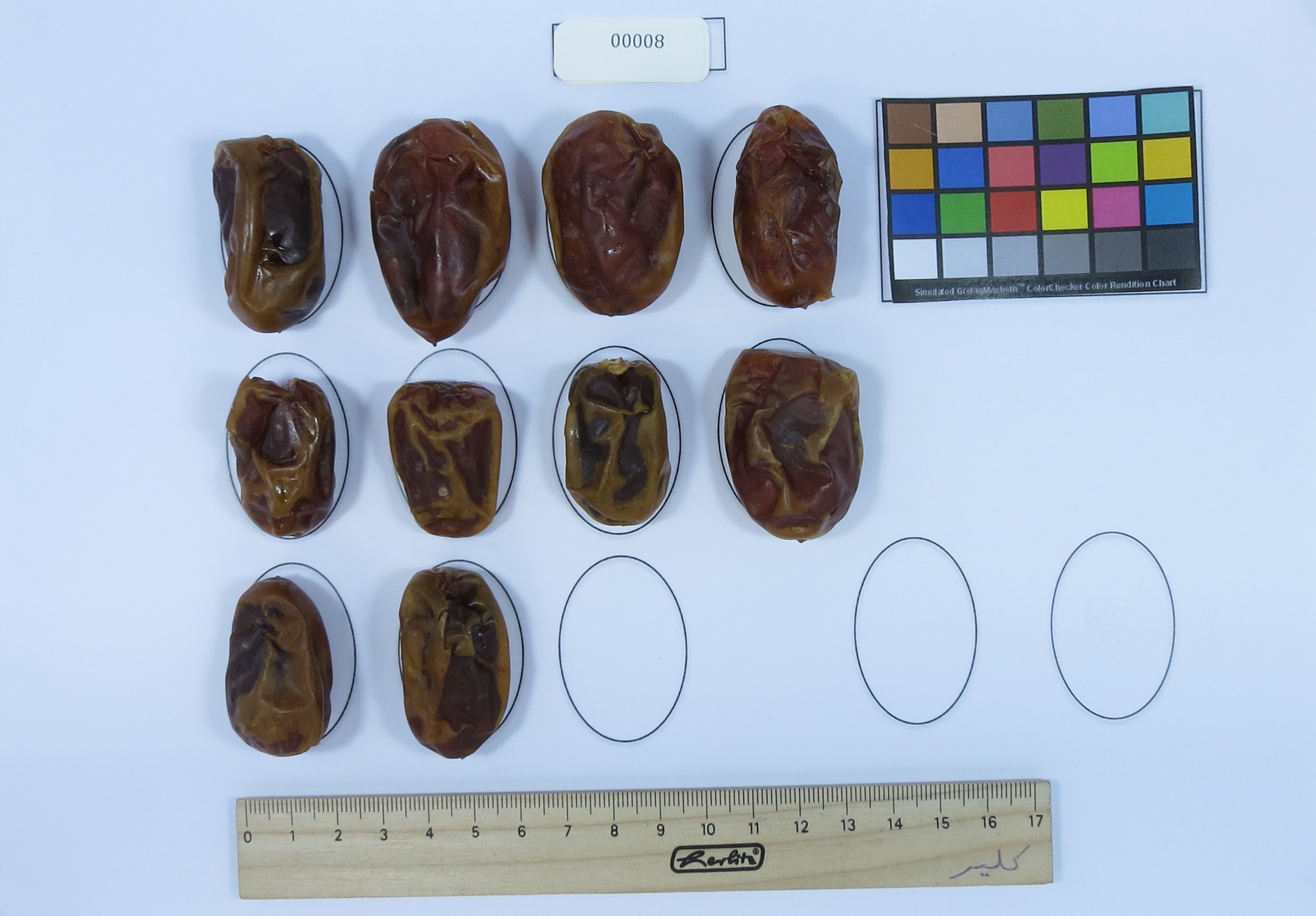

Supplement: Supplementary file 5 — Supplementary material [file mmc5.zip › dates images/00008.JPG]

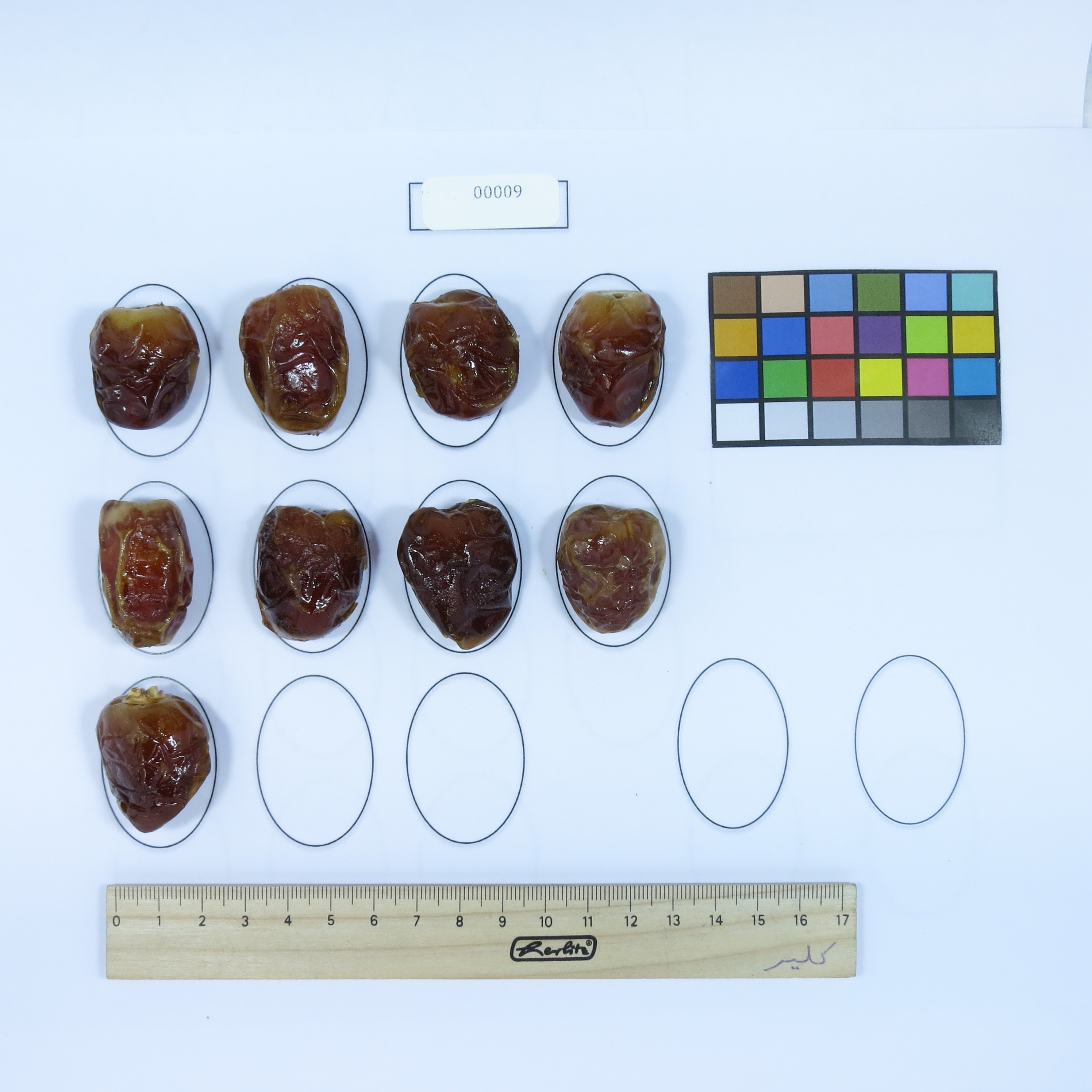

Supplement: Supplementary file 5 — Supplementary material [file mmc5.zip › dates images/00009.JPG]

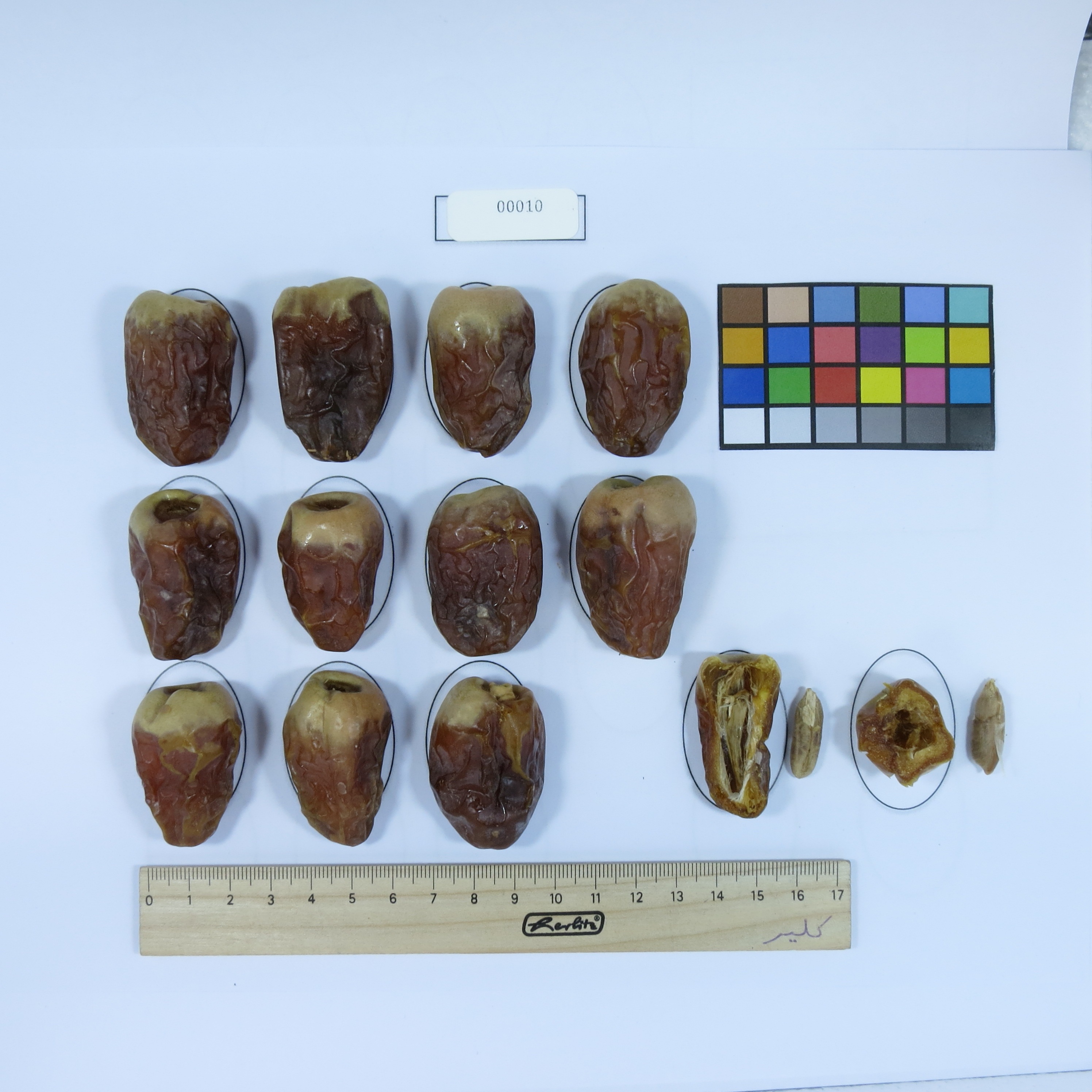

Supplement: Supplementary file 5 — Supplementary material [file mmc5.zip › dates images/00010.JPG]

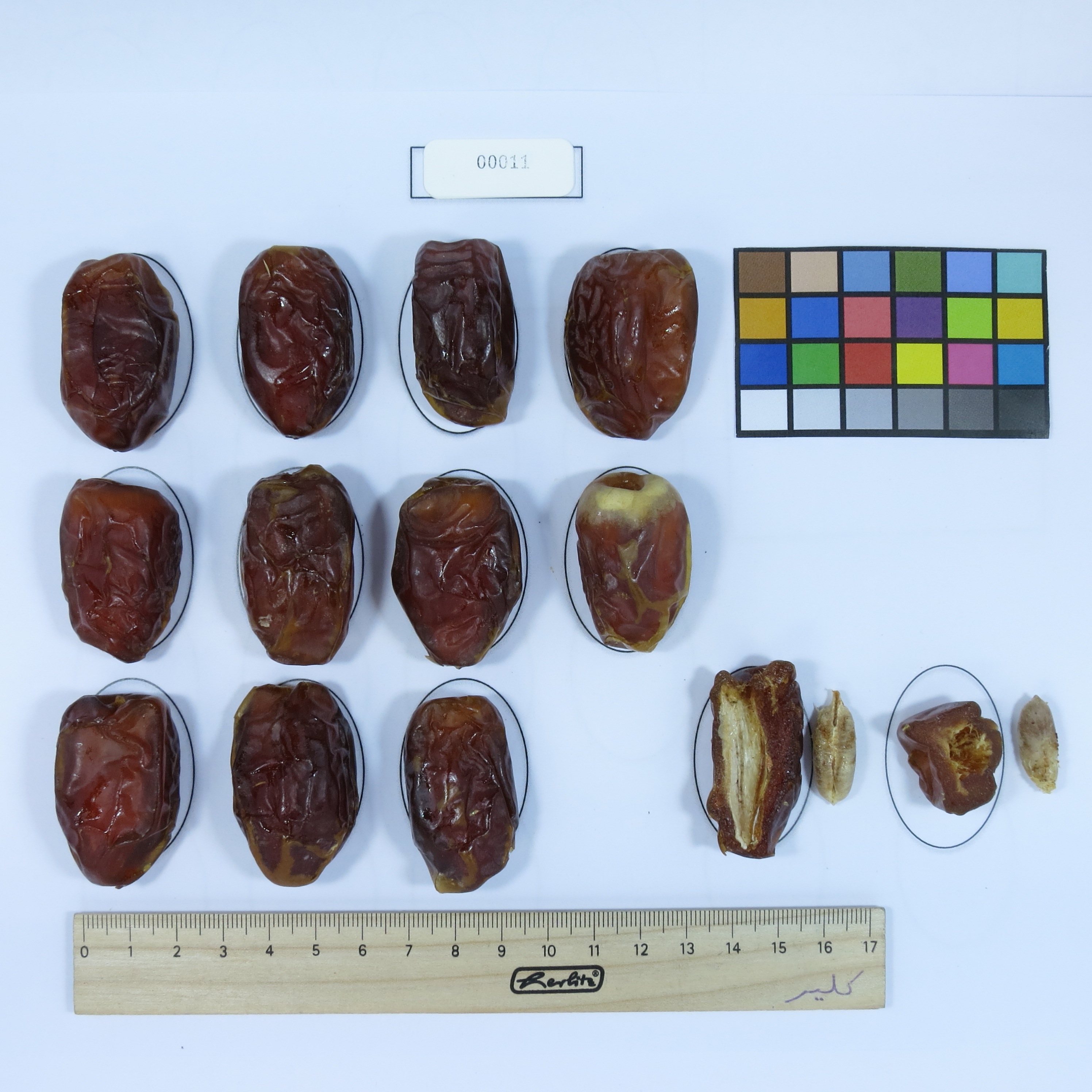

Supplement: Supplementary file 5 — Supplementary material [file mmc5.zip › dates images/00011.JPG]

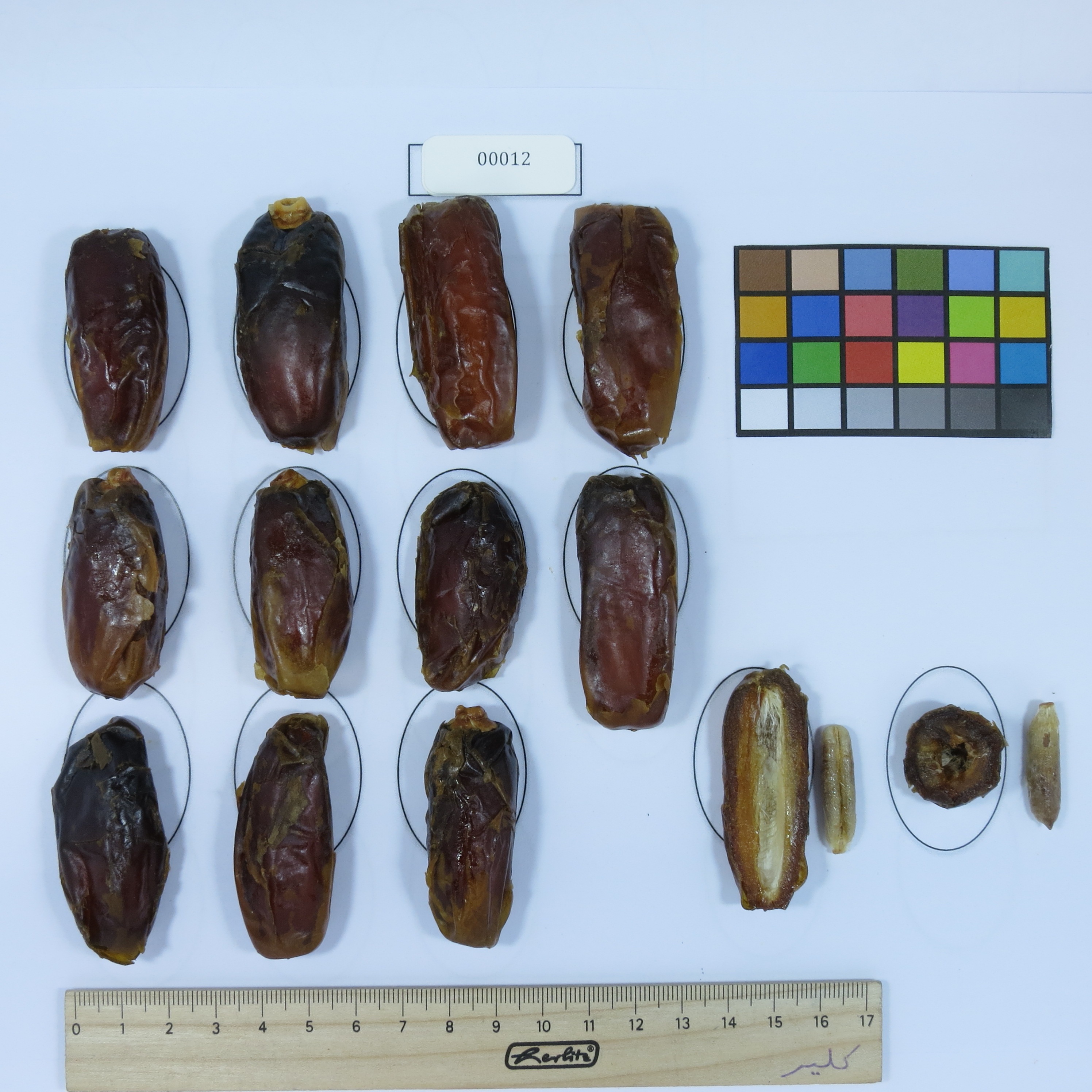

Supplement: Supplementary file 5 — Supplementary material [file mmc5.zip › dates images/00012.JPG]

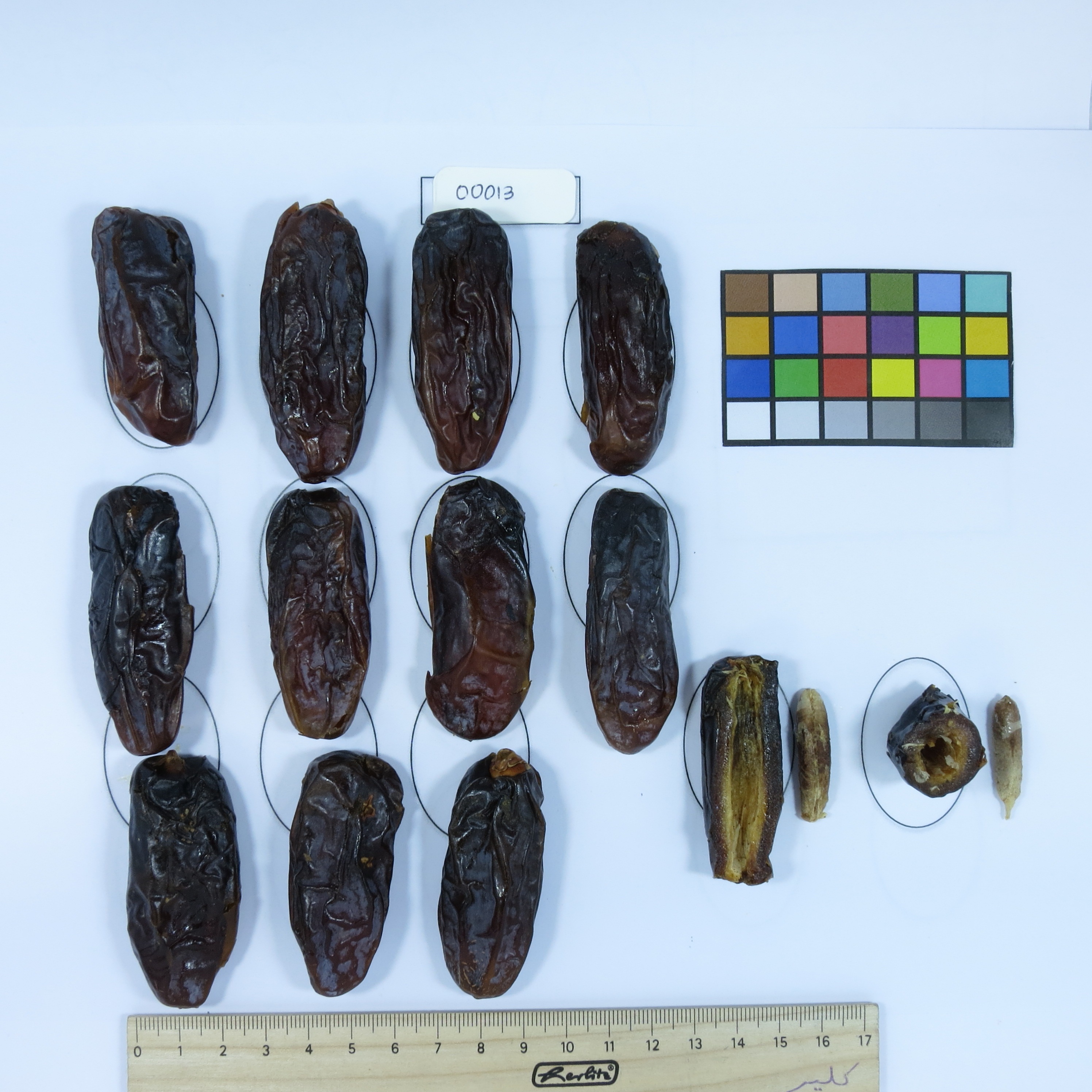

Supplement: Supplementary file 5 — Supplementary material [file mmc5.zip › dates images/00013.JPG]

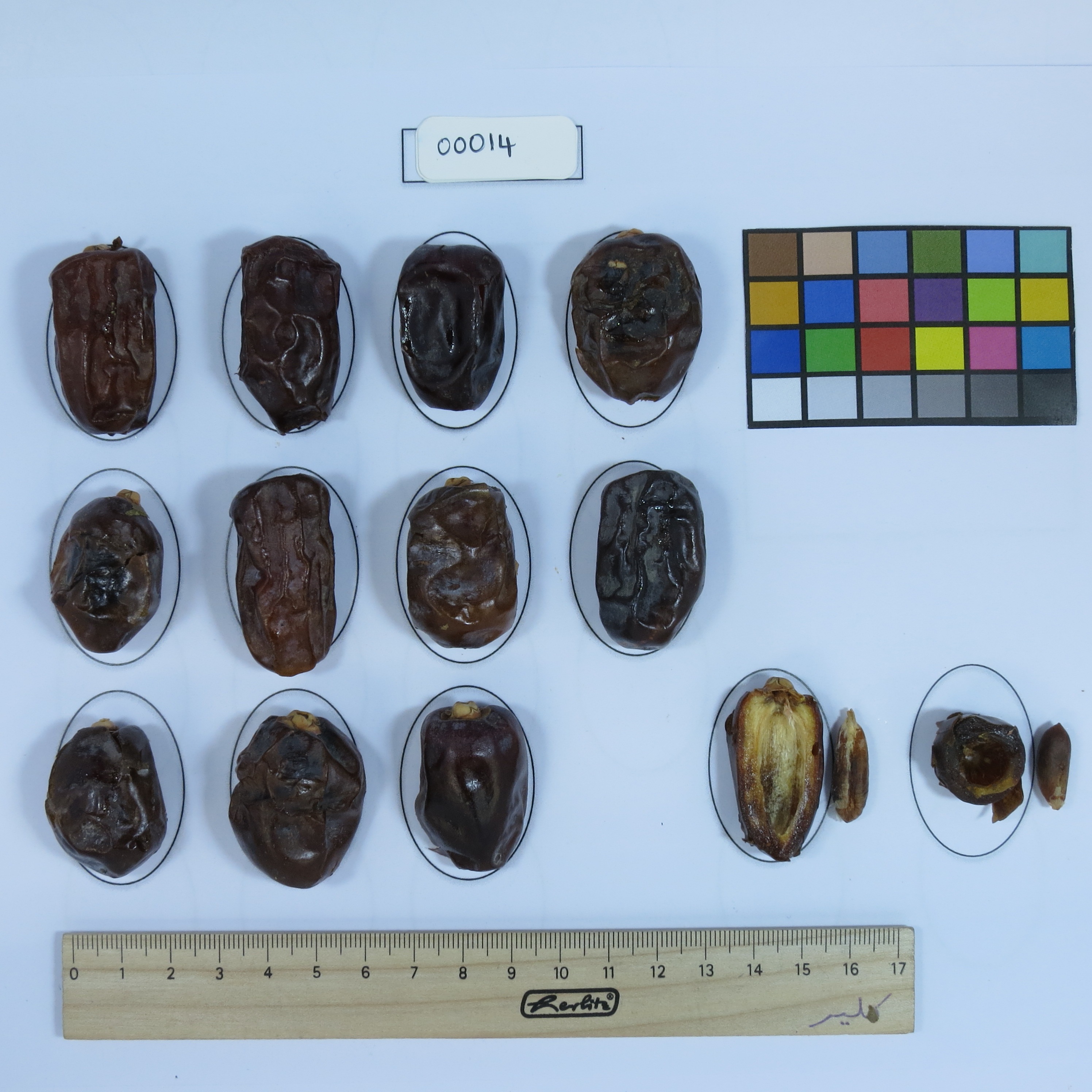

Supplement: Supplementary file 5 — Supplementary material [file mmc5.zip › dates images/00014.JPG]

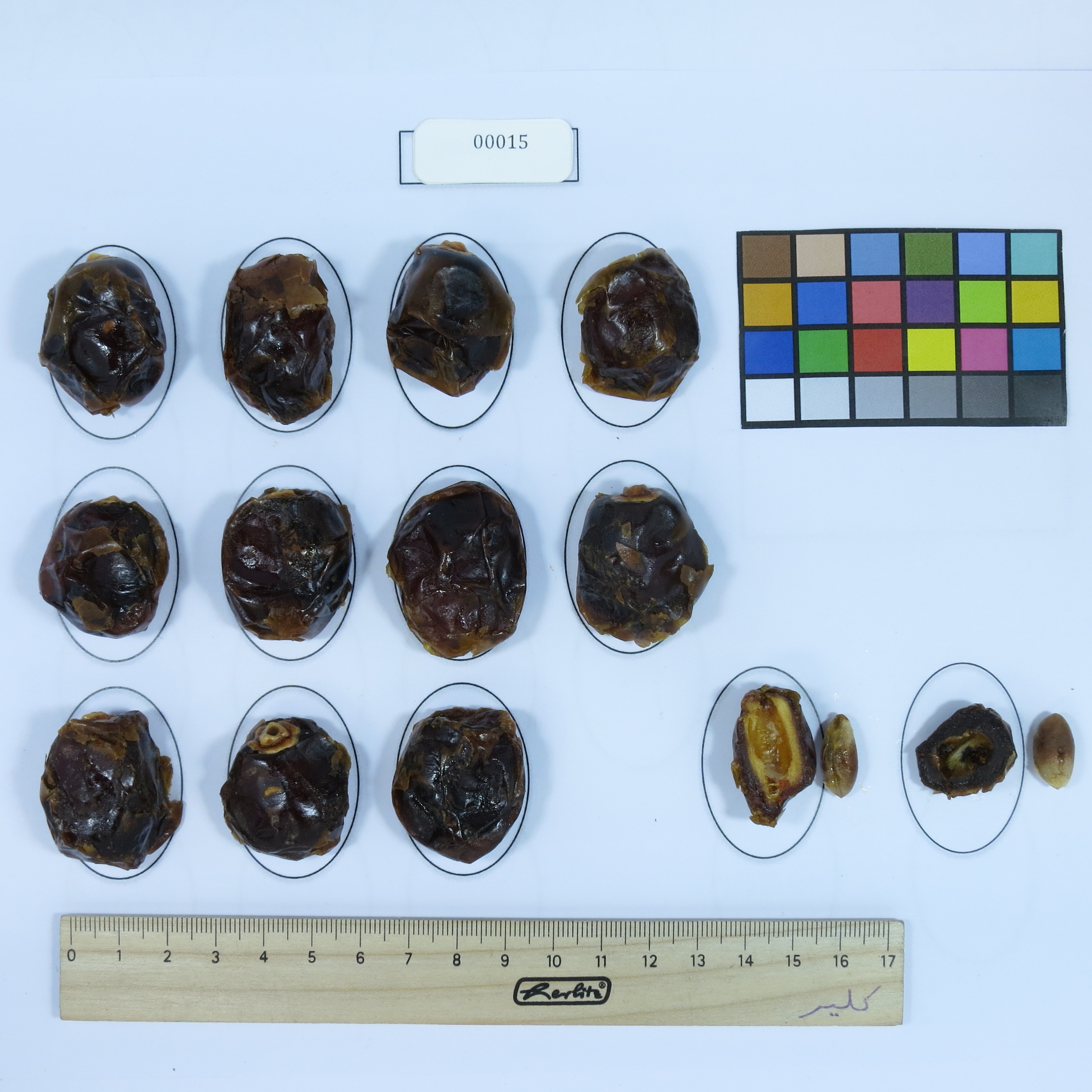

Supplement: Supplementary file 5 — Supplementary material [file mmc5.zip › dates images/00015.JPG]

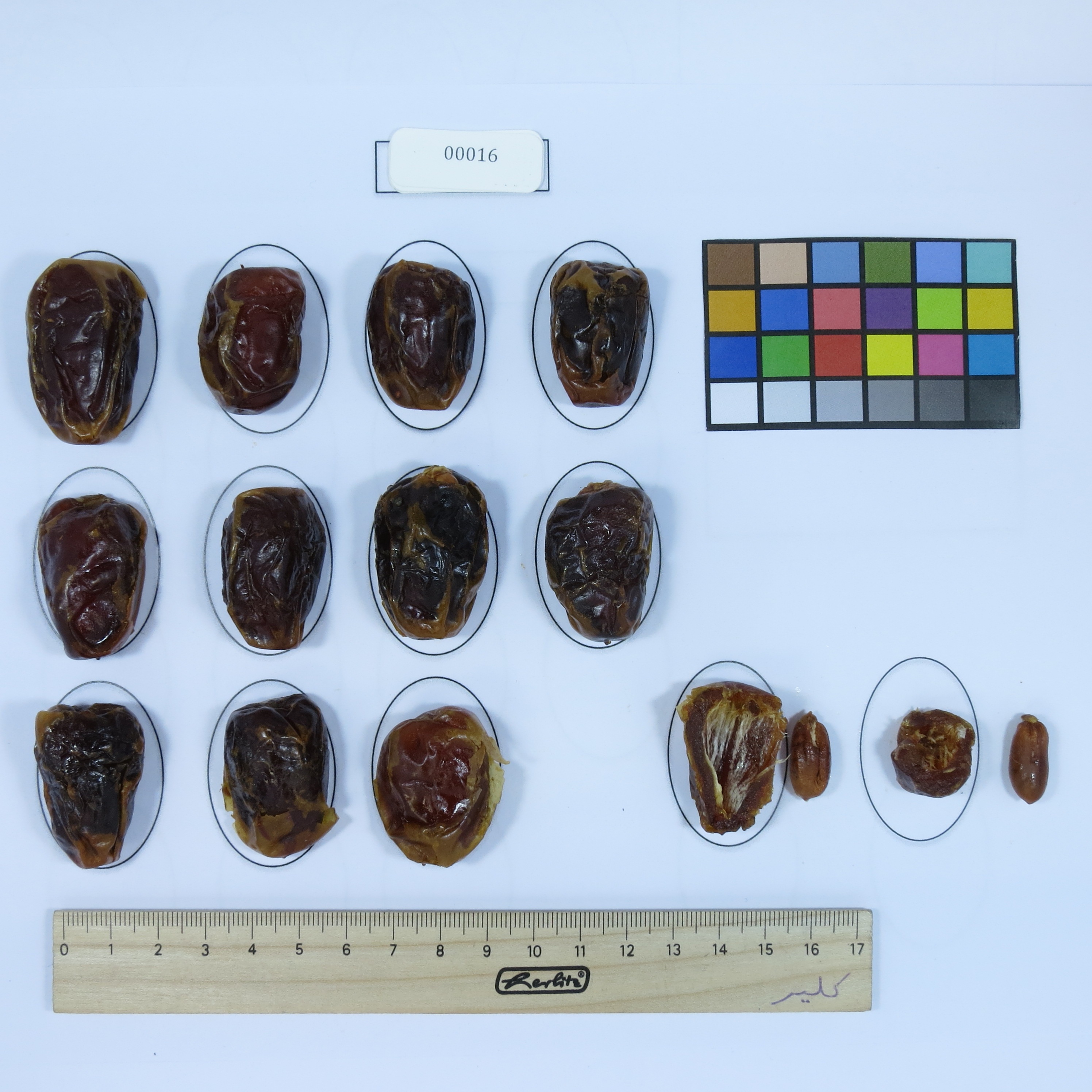

Supplement: Supplementary file 5 — Supplementary material [file mmc5.zip › dates images/00016.JPG]

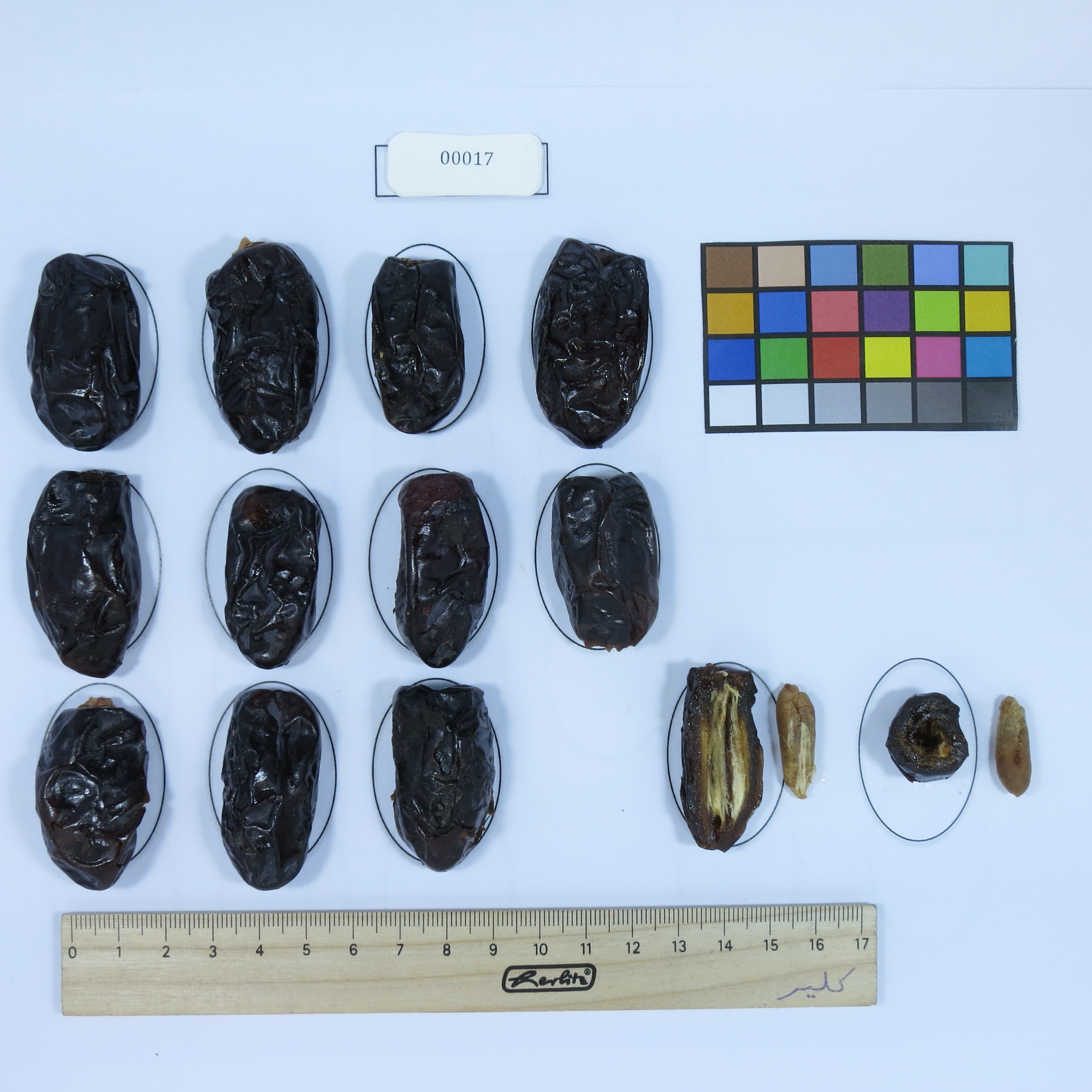

Supplement: Supplementary file 5 — Supplementary material [file mmc5.zip › dates images/00017.JPG]

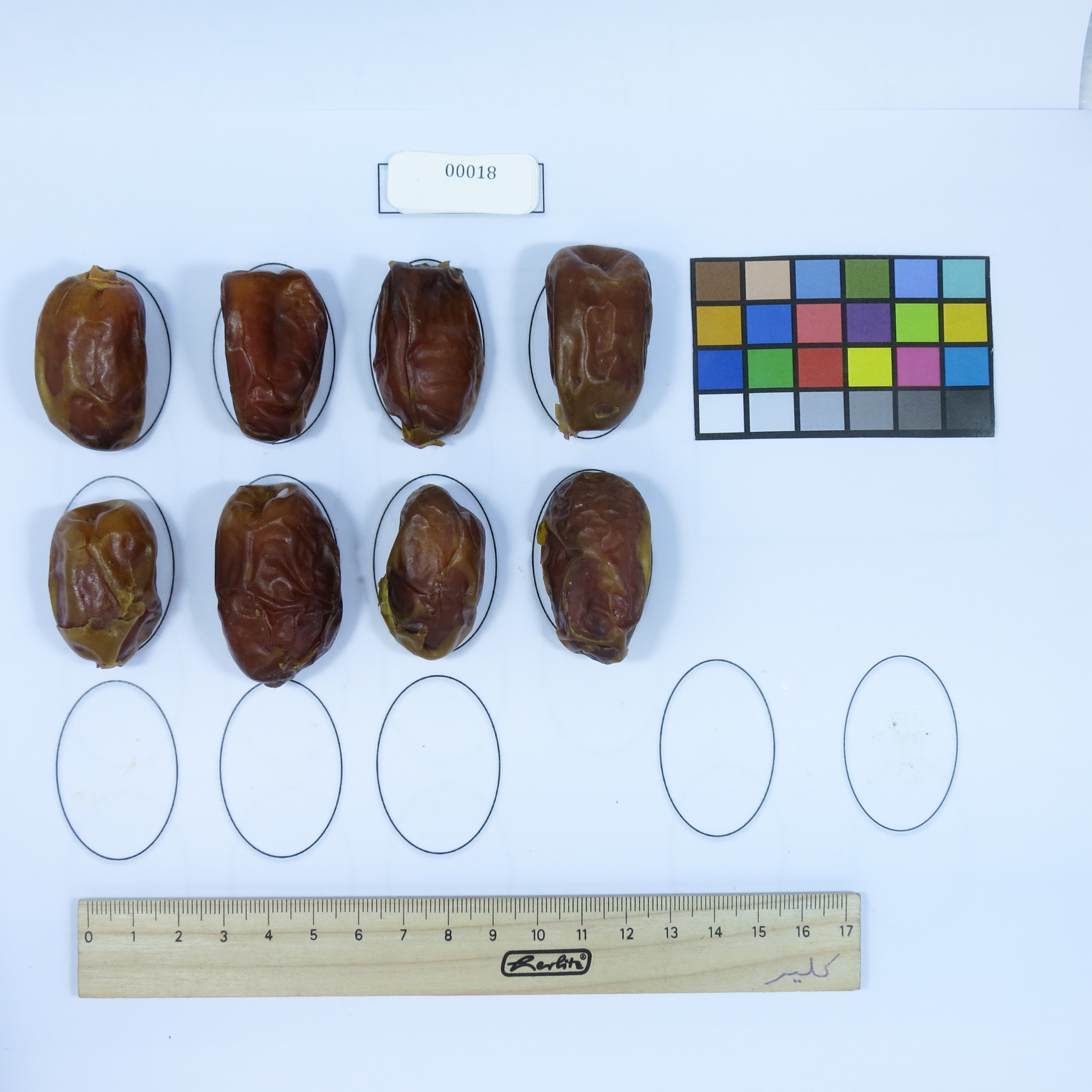

Supplement: Supplementary file 5 — Supplementary material [file mmc5.zip › dates images/00018.JPG]

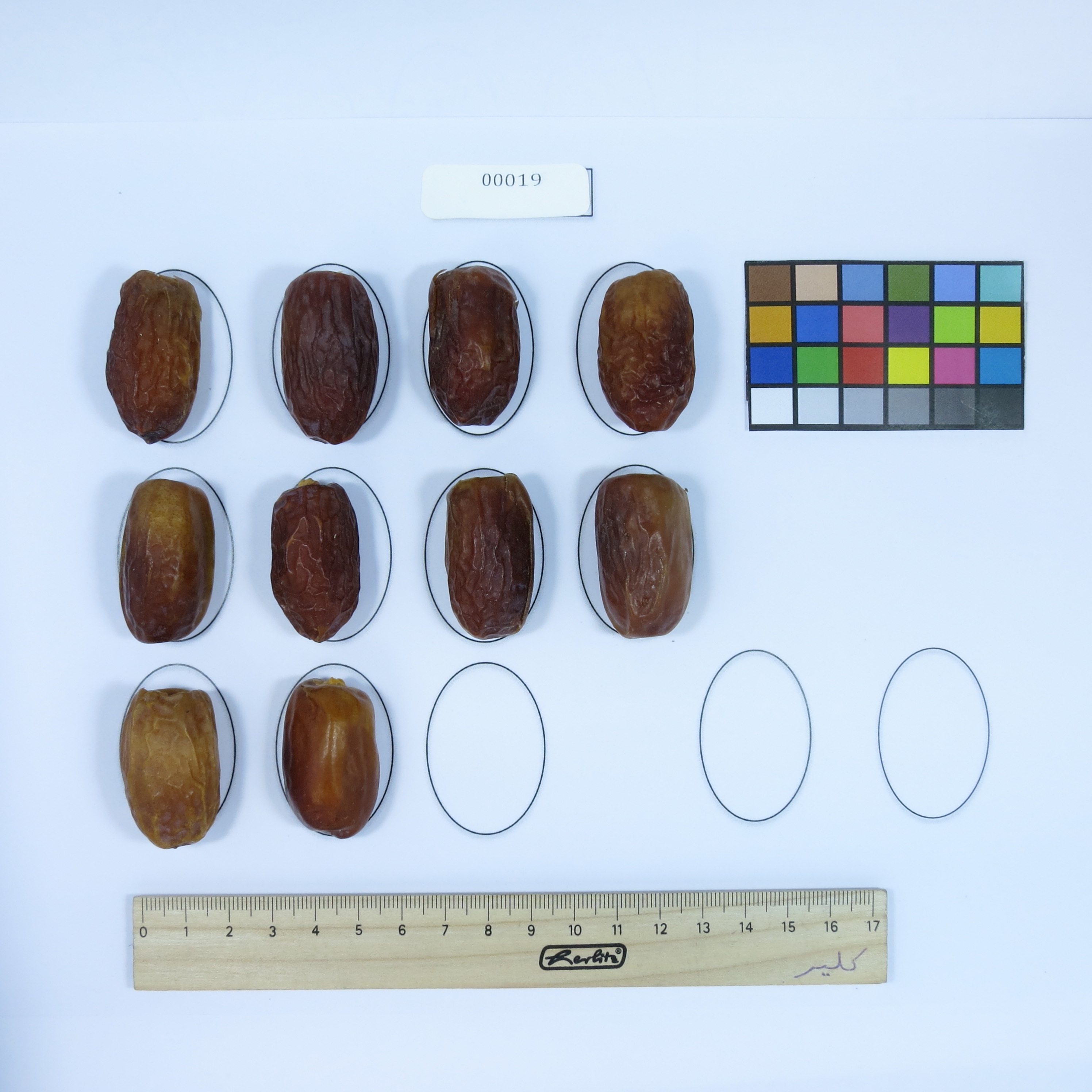

Supplement: Supplementary file 5 — Supplementary material [file mmc5.zip › dates images/00019.JPG]

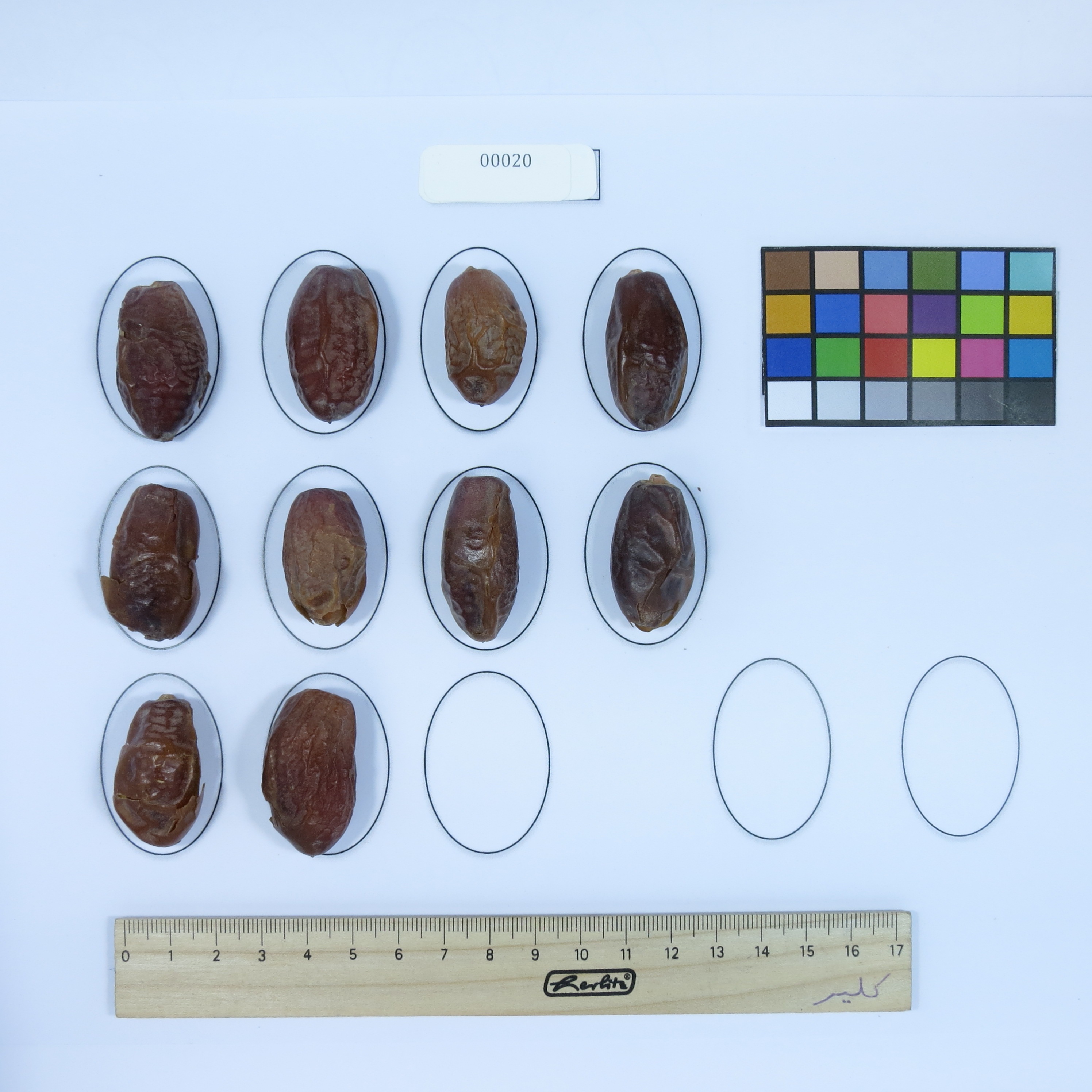

Supplement: Supplementary file 5 — Supplementary material [file mmc5.zip › dates images/00020.JPG]

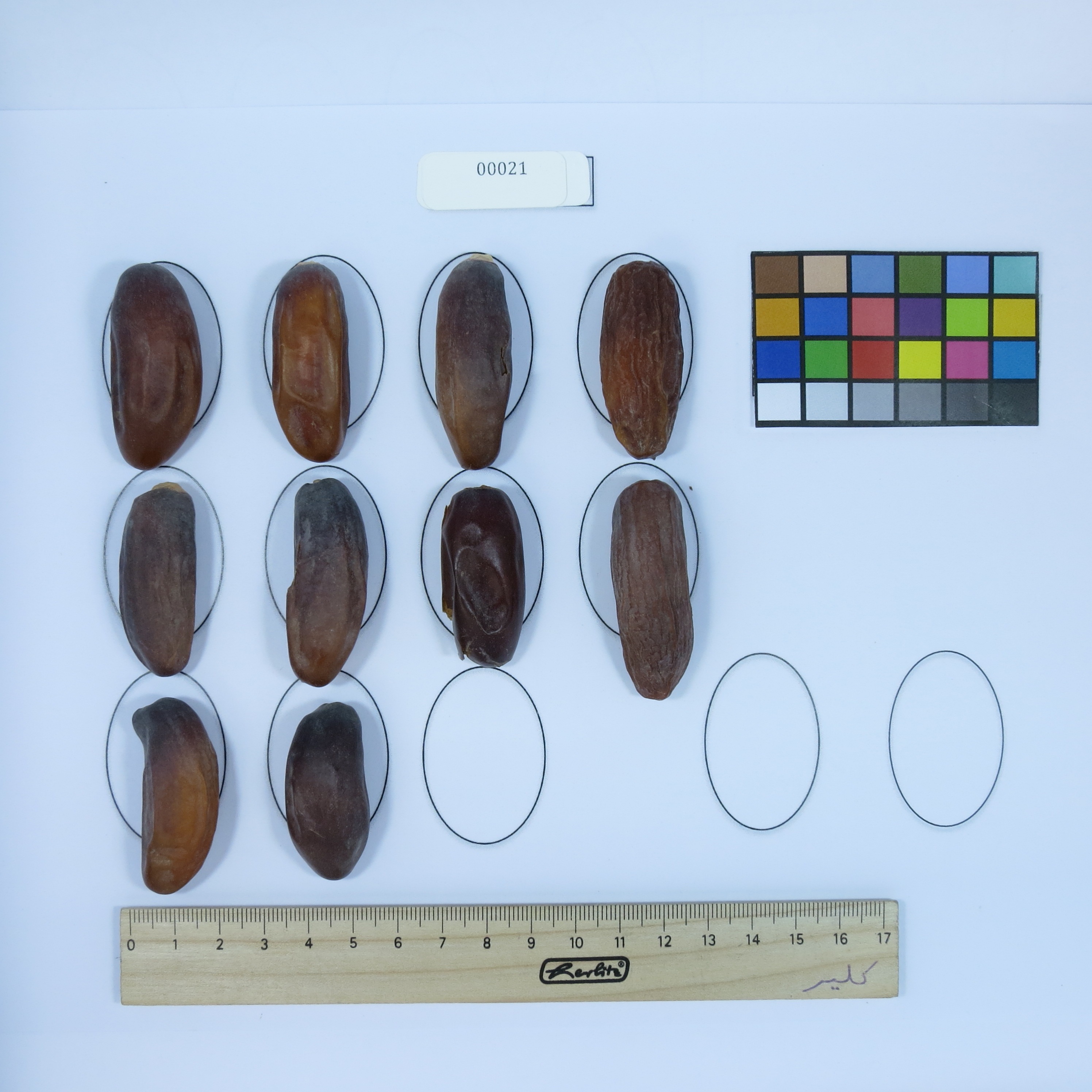

Supplement: Supplementary file 5 — Supplementary material [file mmc5.zip › dates images/00021.JPG]

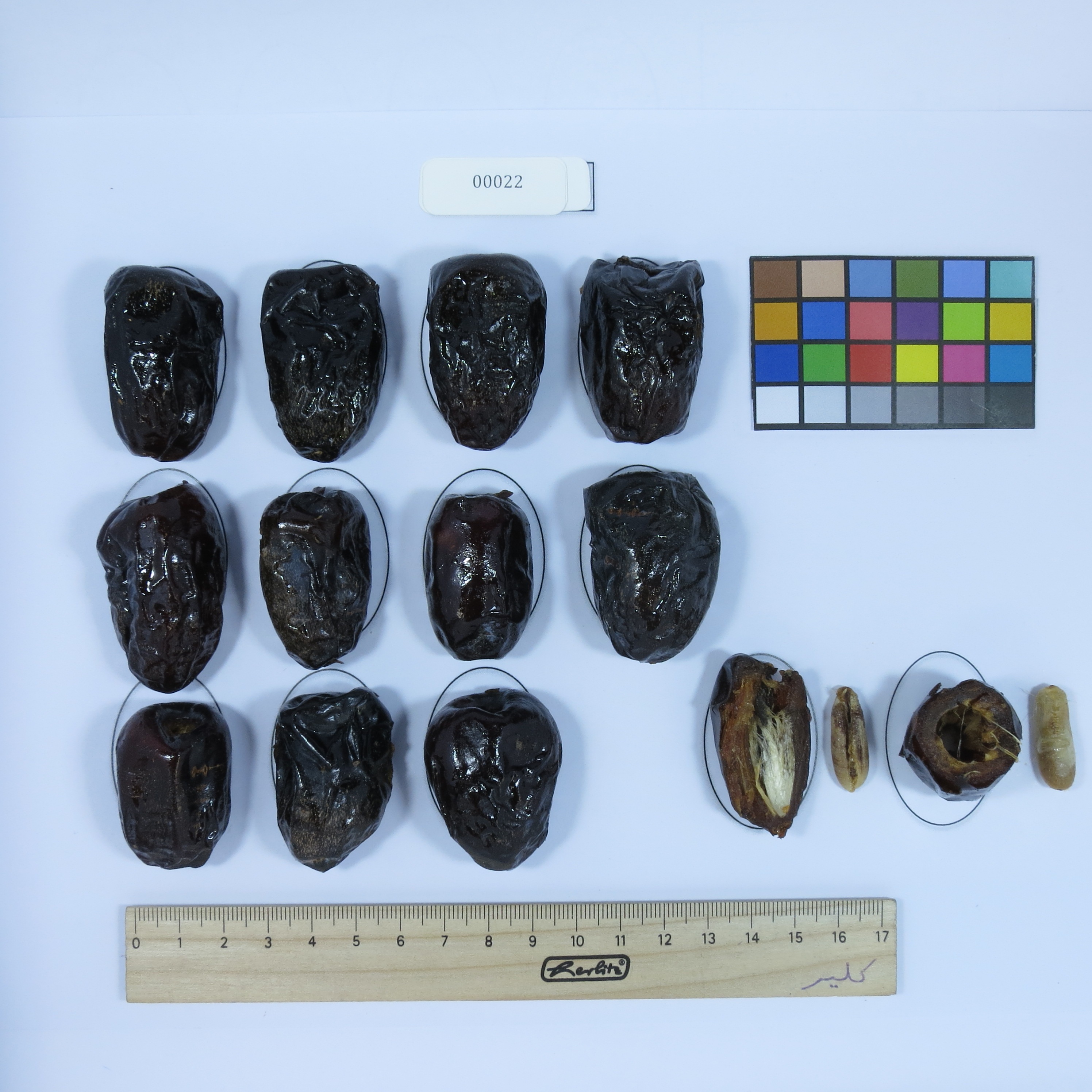

Supplement: Supplementary file 5 — Supplementary material [file mmc5.zip › dates images/00022.JPG]

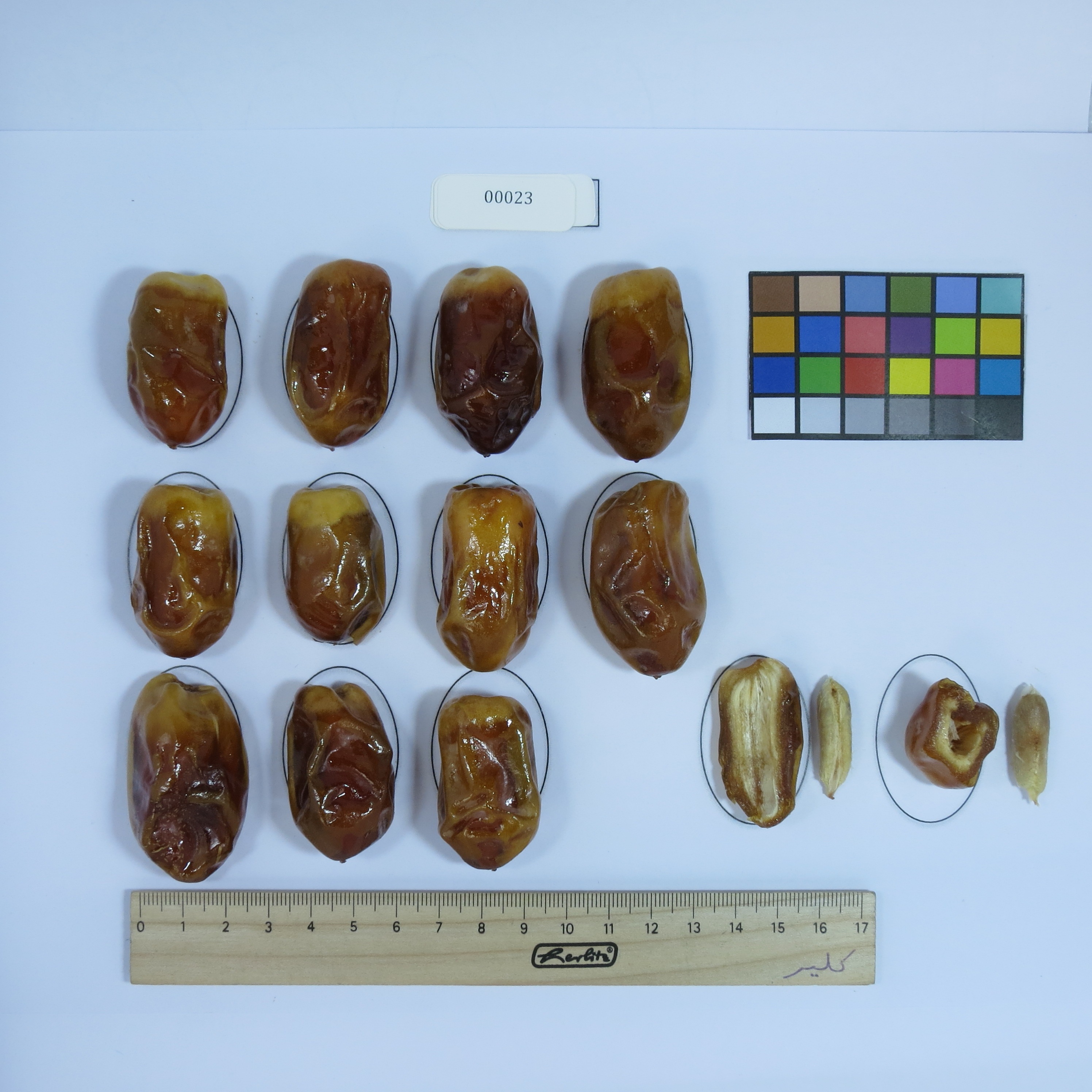

Supplement: Supplementary file 5 — Supplementary material [file mmc5.zip › dates images/00023.JPG]

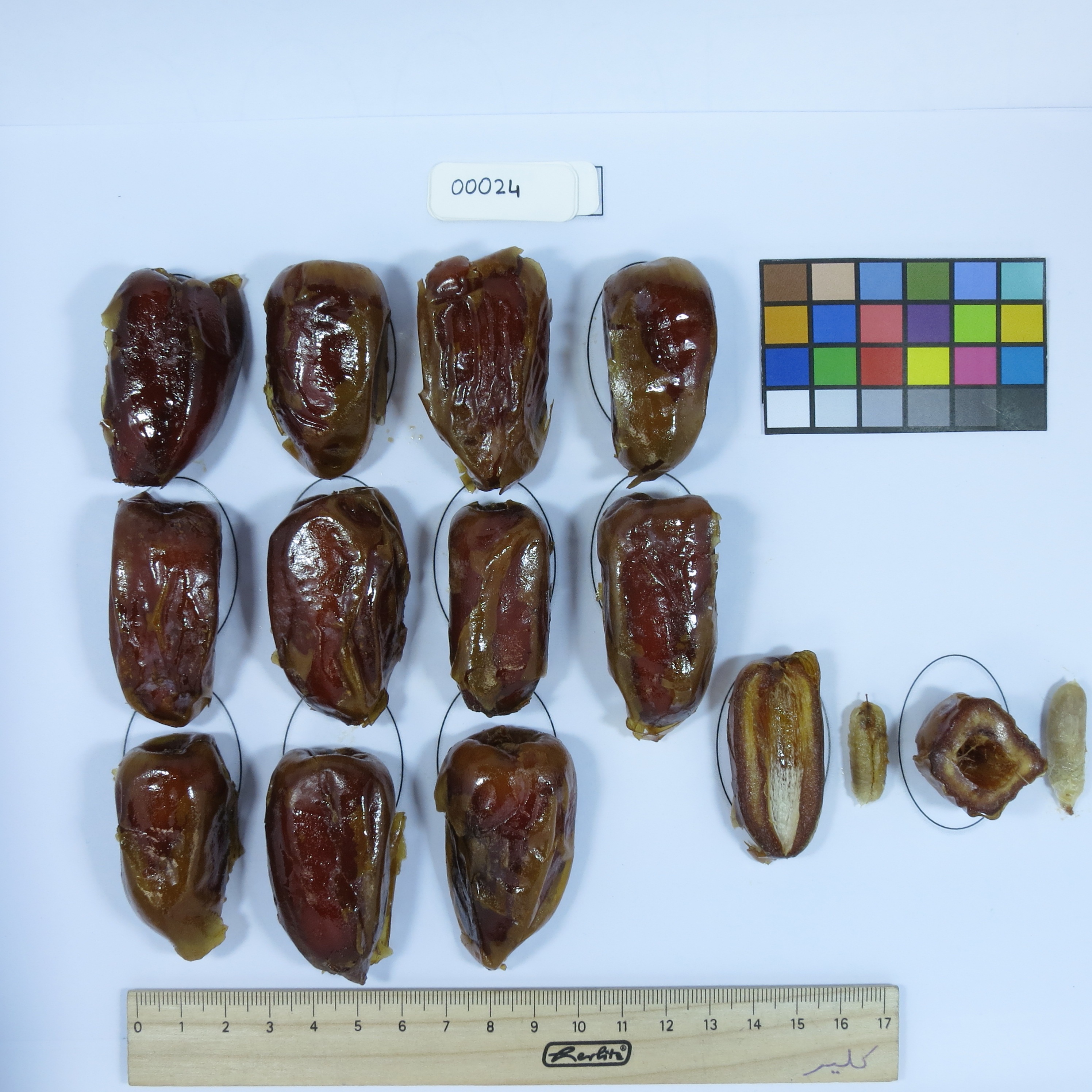

Supplement: Supplementary file 5 — Supplementary material [file mmc5.zip › dates images/00024.JPG]

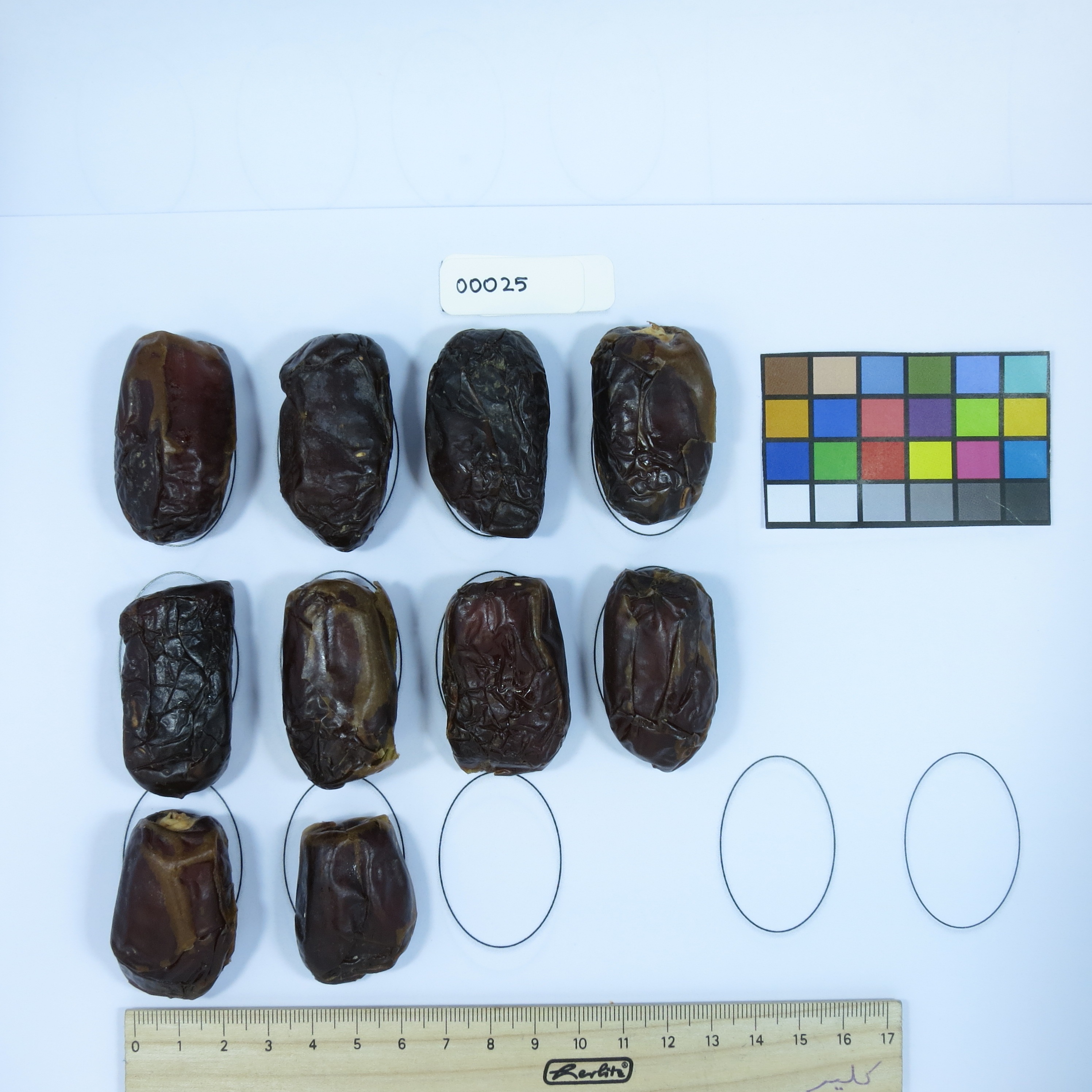

Supplement: Supplementary file 5 — Supplementary material [file mmc5.zip › dates images/00025.JPG]

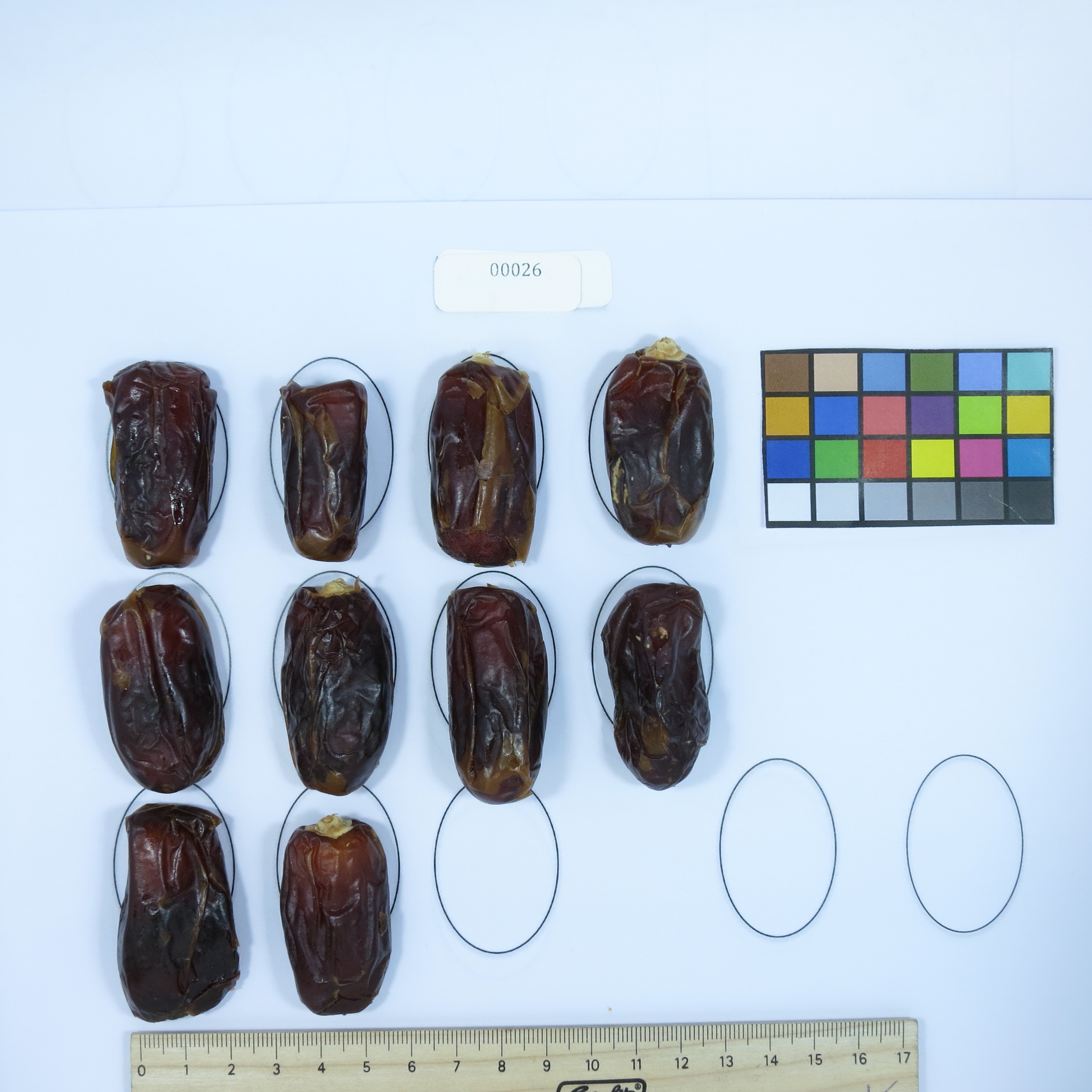

Supplement: Supplementary file 5 — Supplementary material [file mmc5.zip › dates images/00026.JPG]

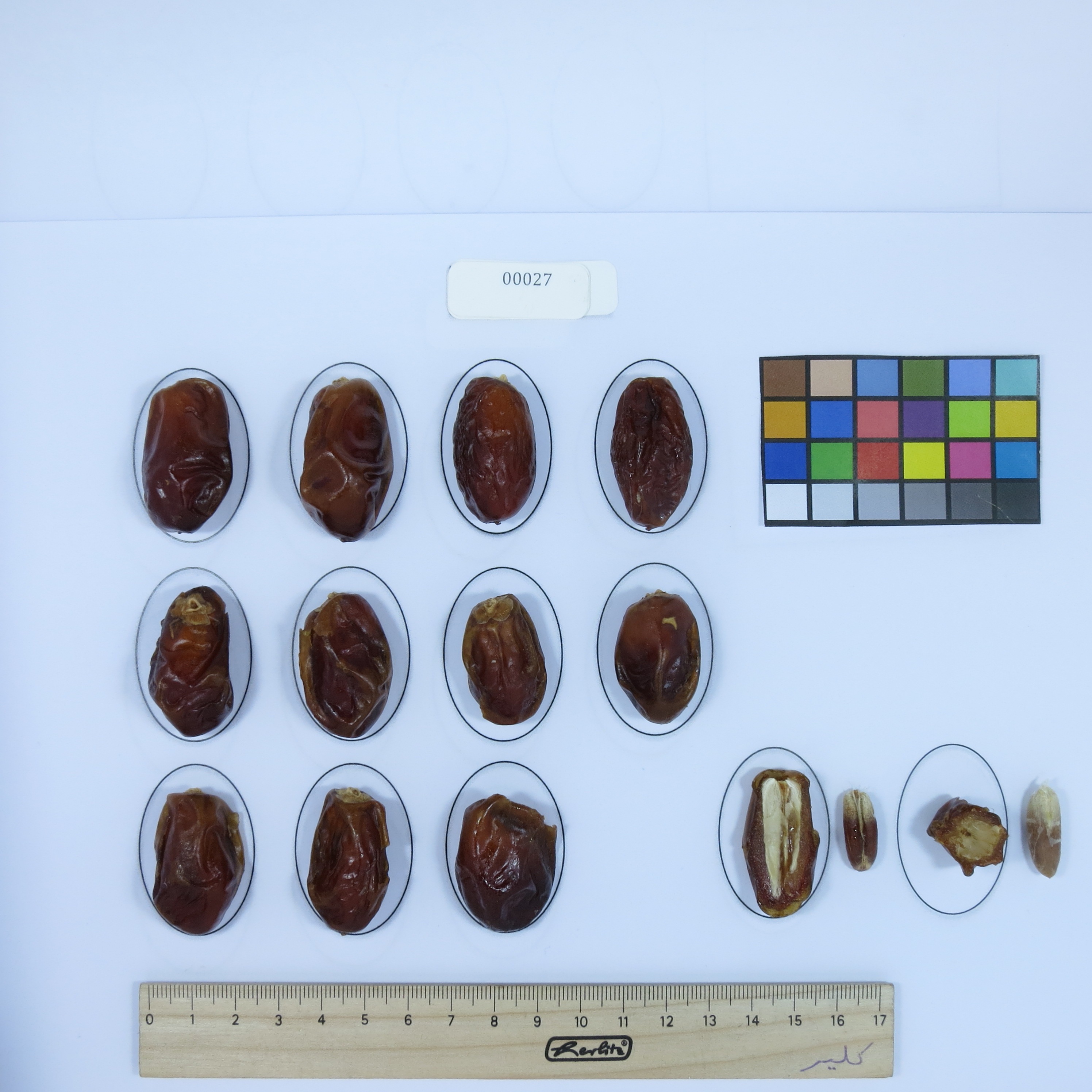

Supplement: Supplementary file 5 — Supplementary material [file mmc5.zip › dates images/00027.JPG]

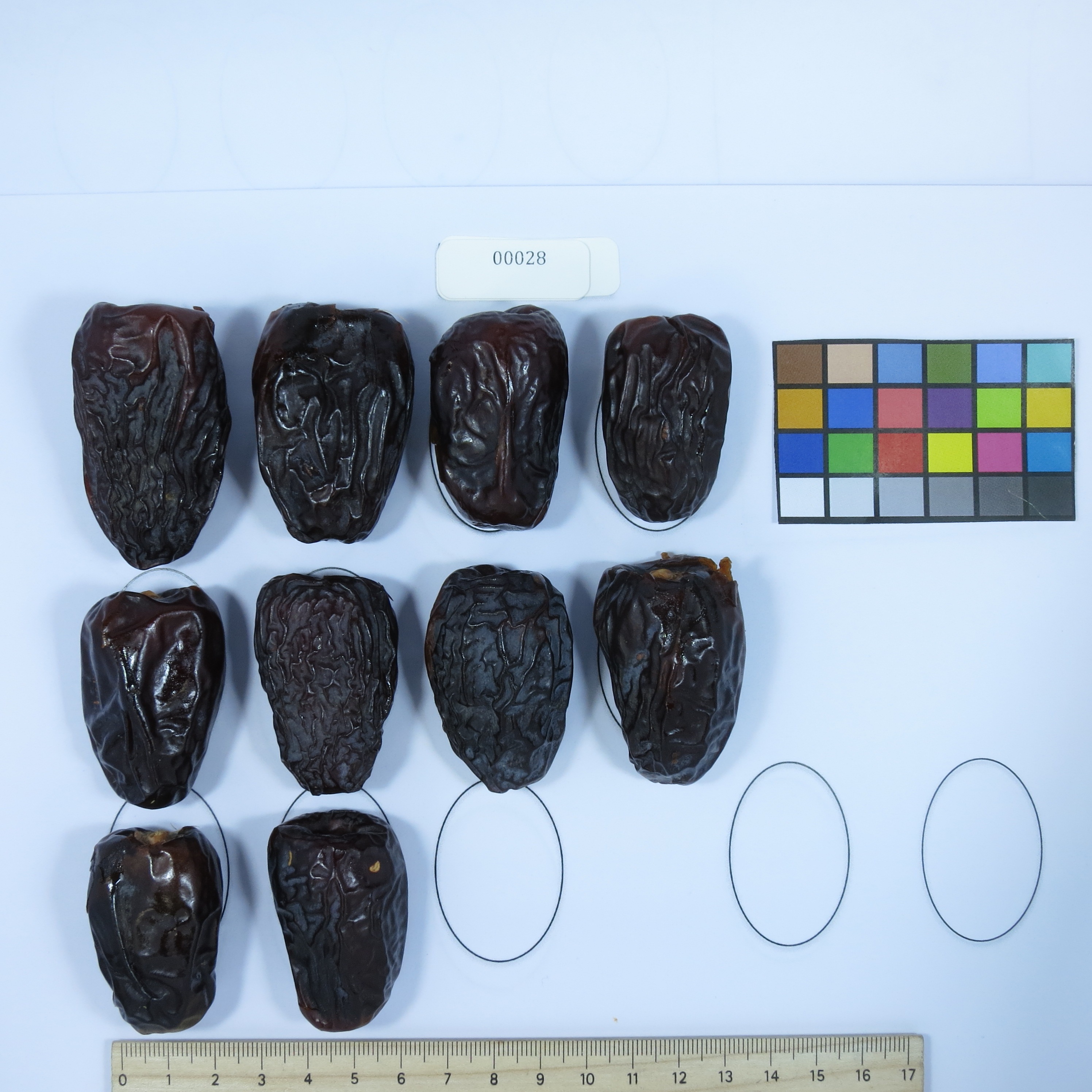

Supplement: Supplementary file 5 — Supplementary material [file mmc5.zip › dates images/00028.JPG]

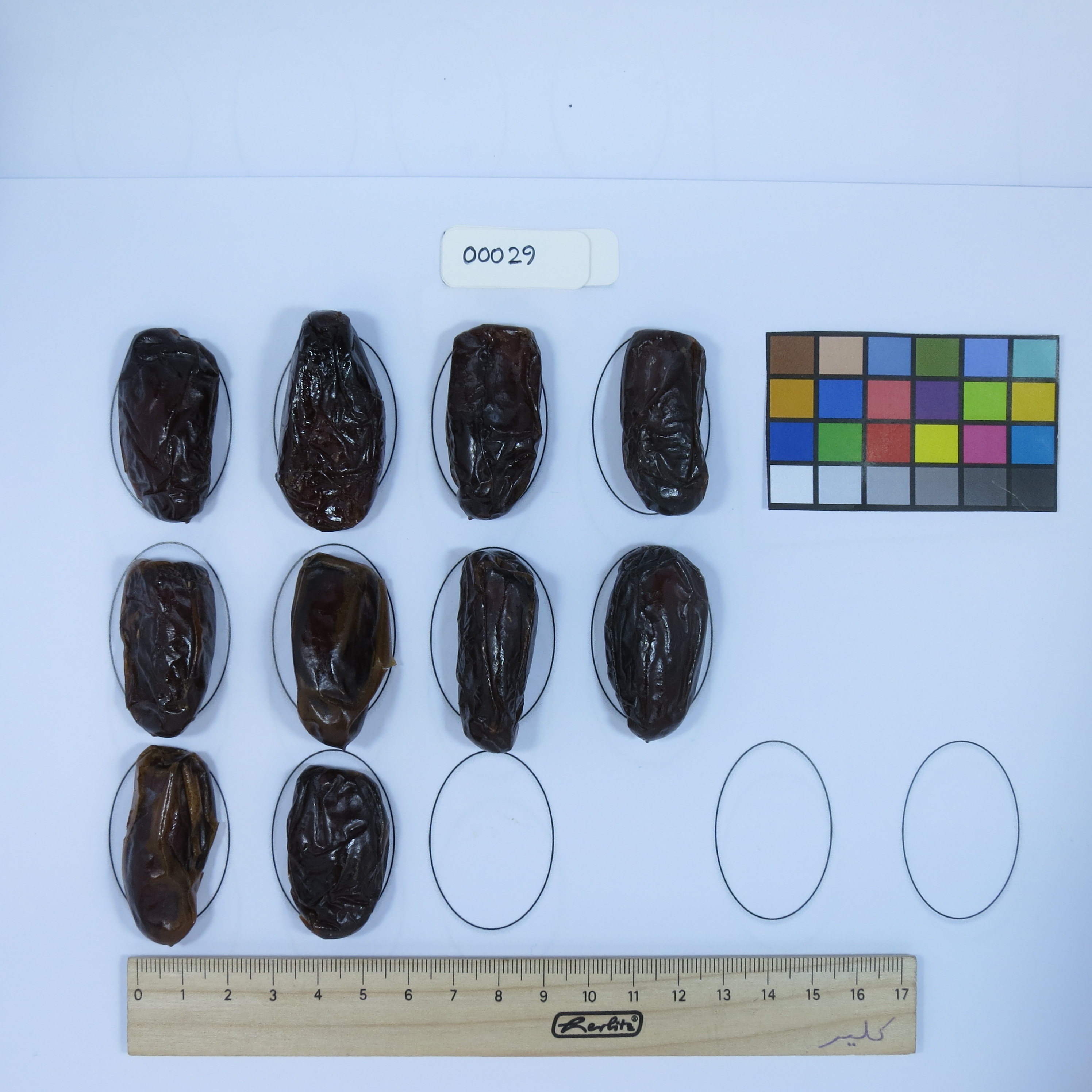

Supplement: Supplementary file 5 — Supplementary material [file mmc5.zip › dates images/00029.JPG]

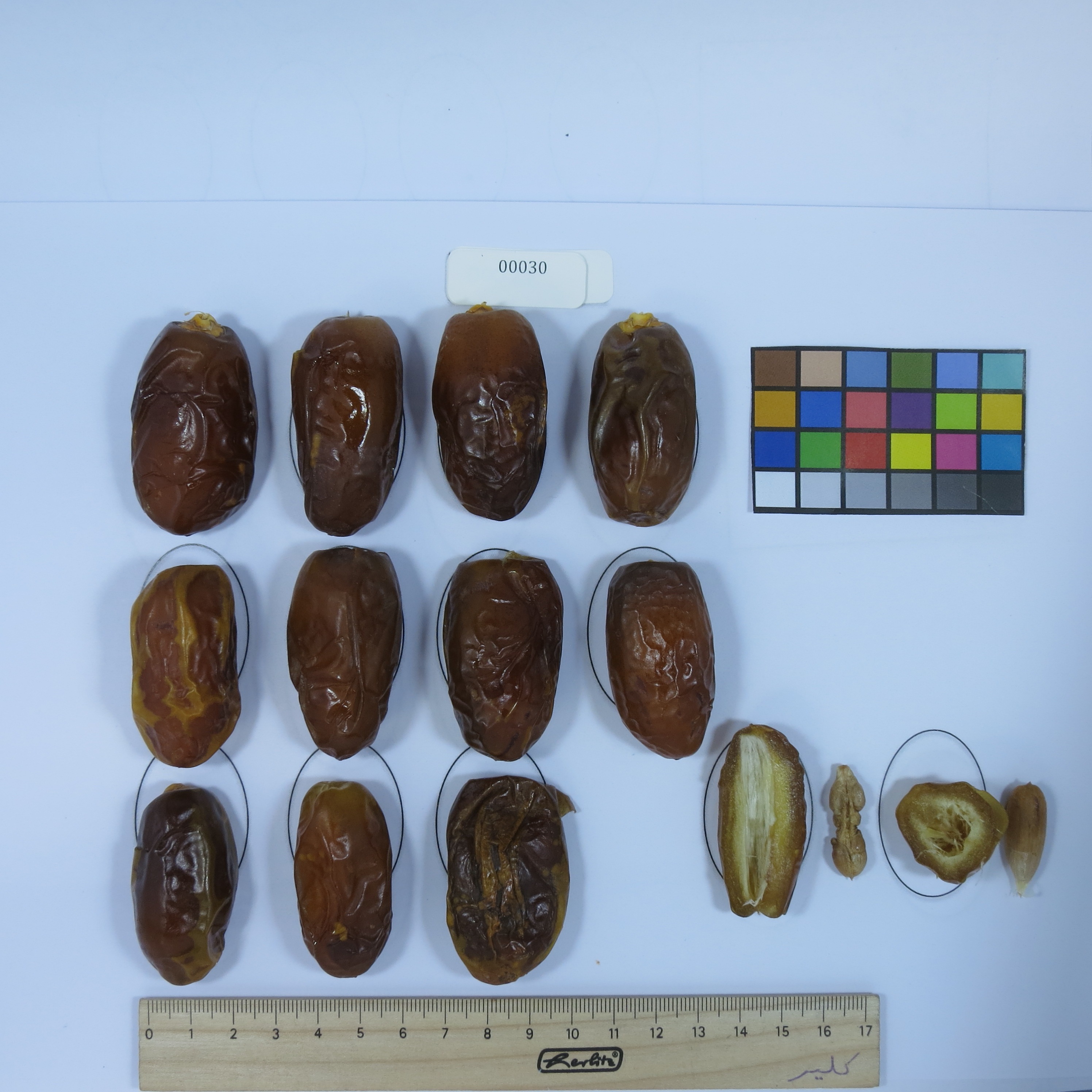

Supplement: Supplementary file 5 — Supplementary material [file mmc5.zip › dates images/00030.JPG]

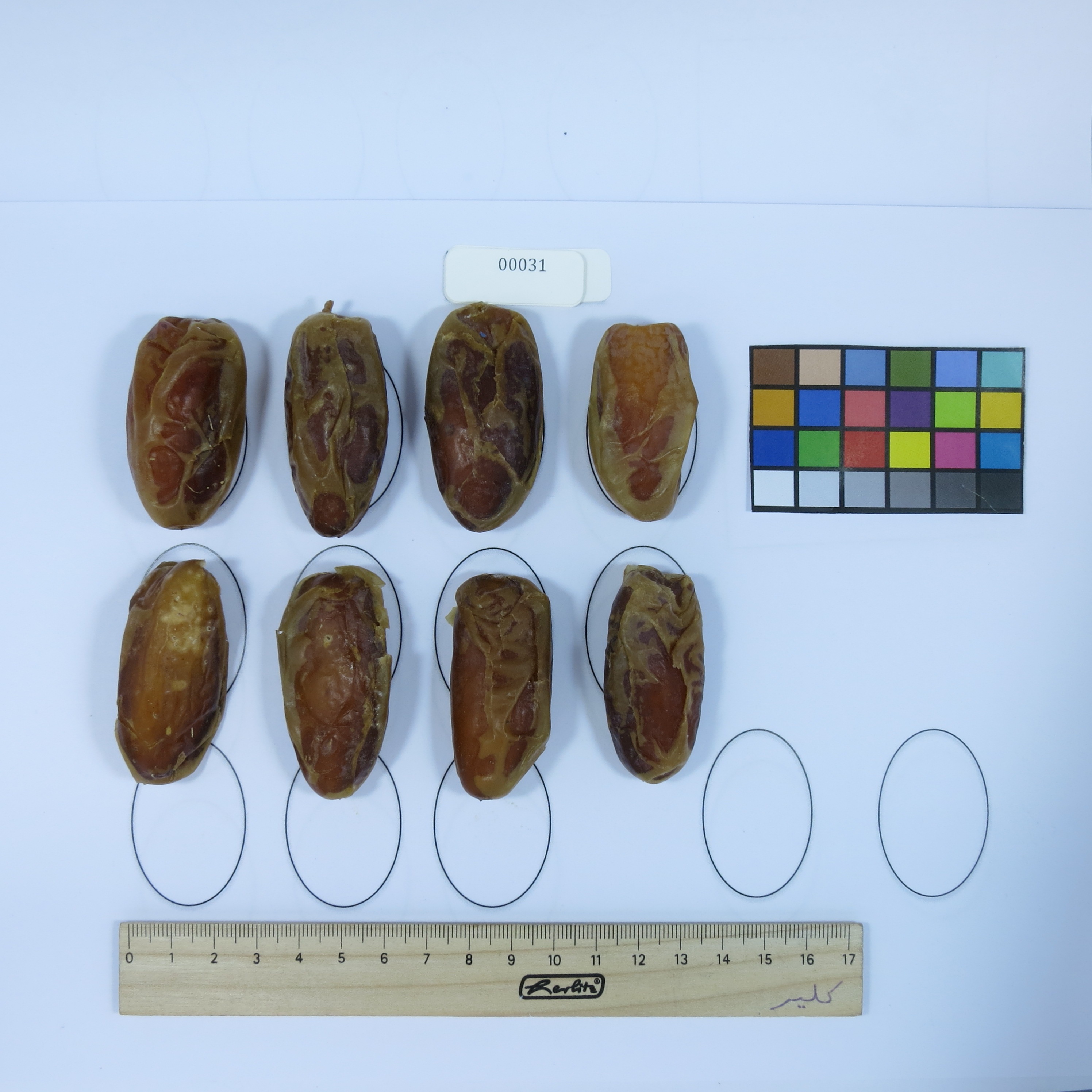

Supplement: Supplementary file 5 — Supplementary material [file mmc5.zip › dates images/00031.JPG]

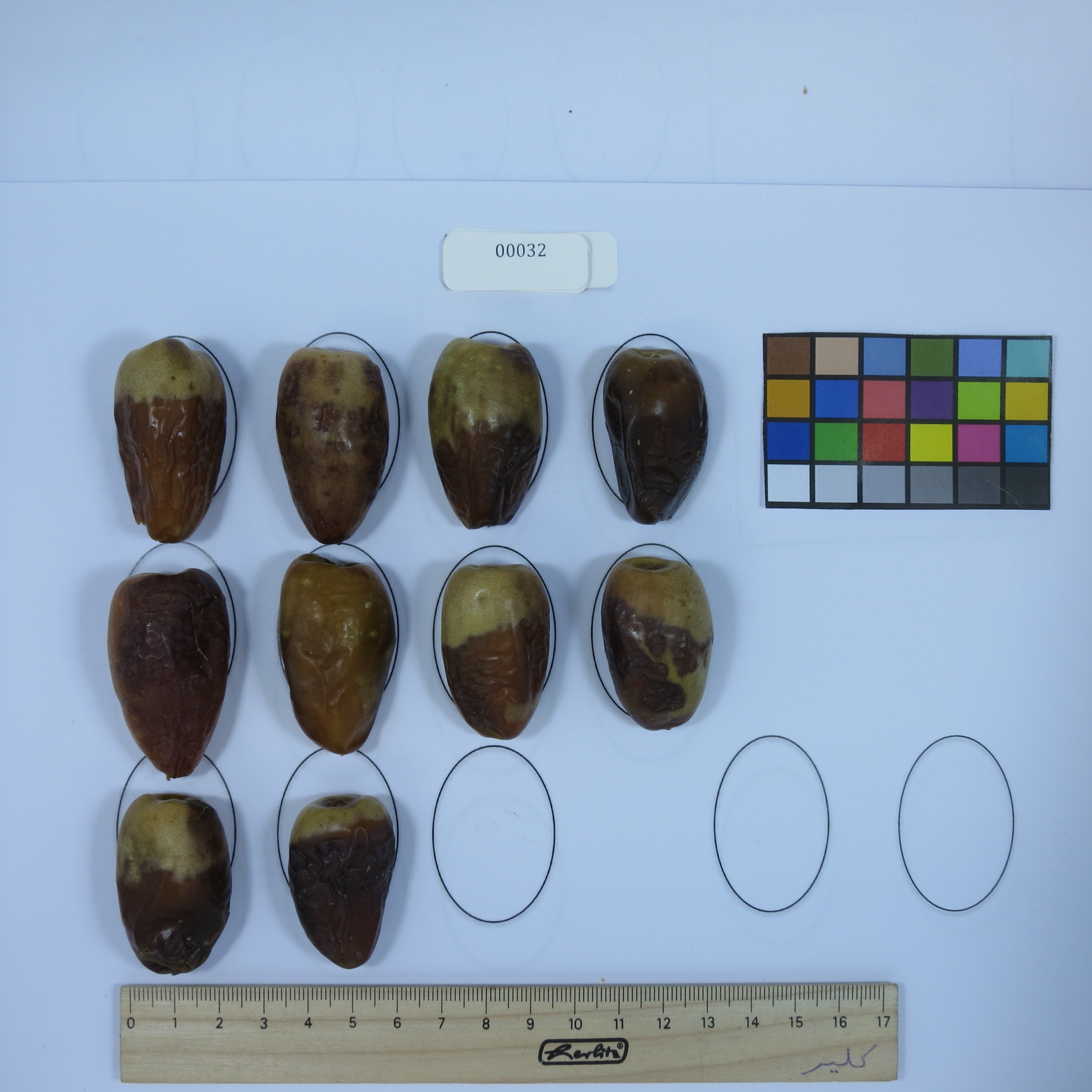

Supplement: Supplementary file 5 — Supplementary material [file mmc5.zip › dates images/00032.JPG]

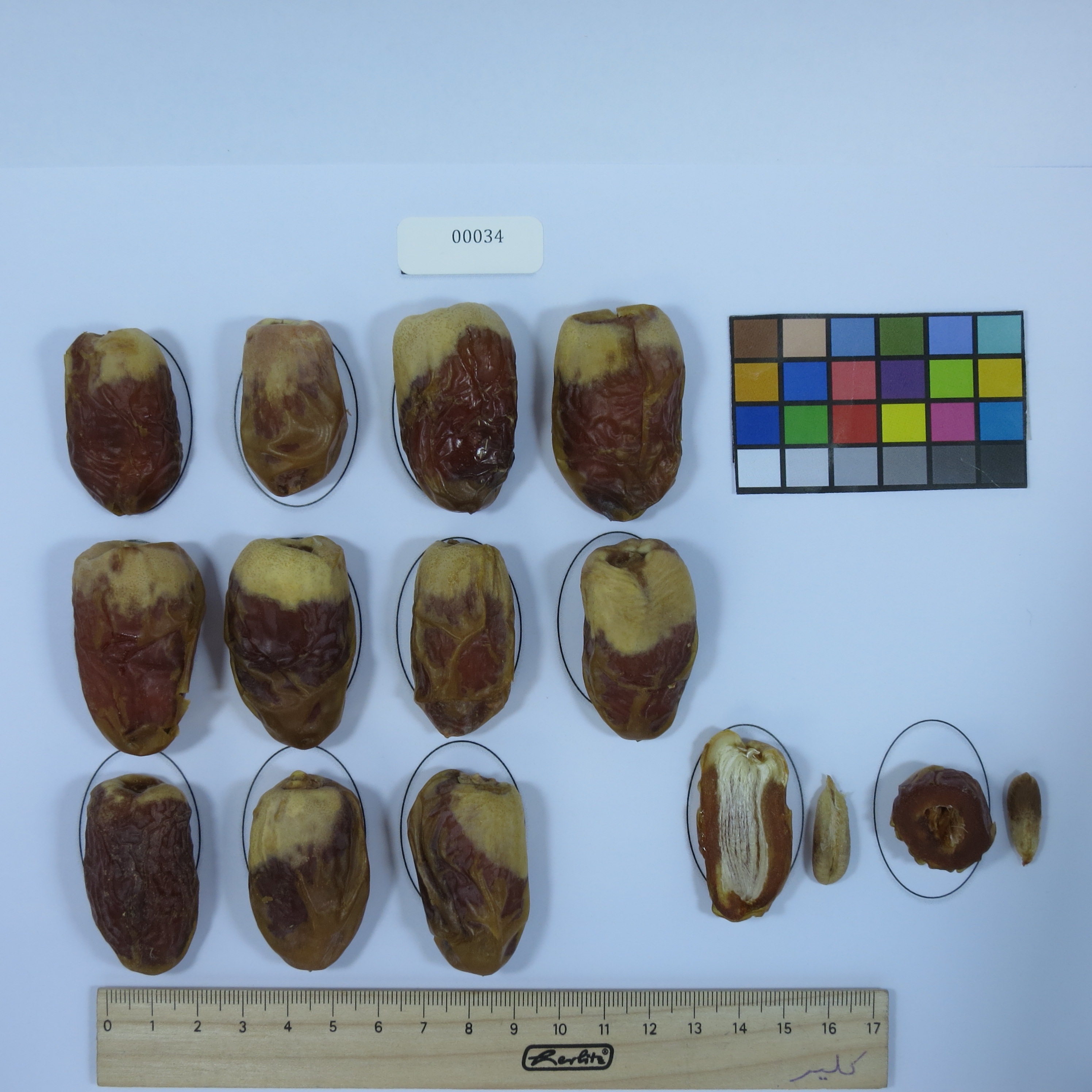

Supplement: Supplementary file 5 — Supplementary material [file mmc5.zip › dates images/00034.JPG]

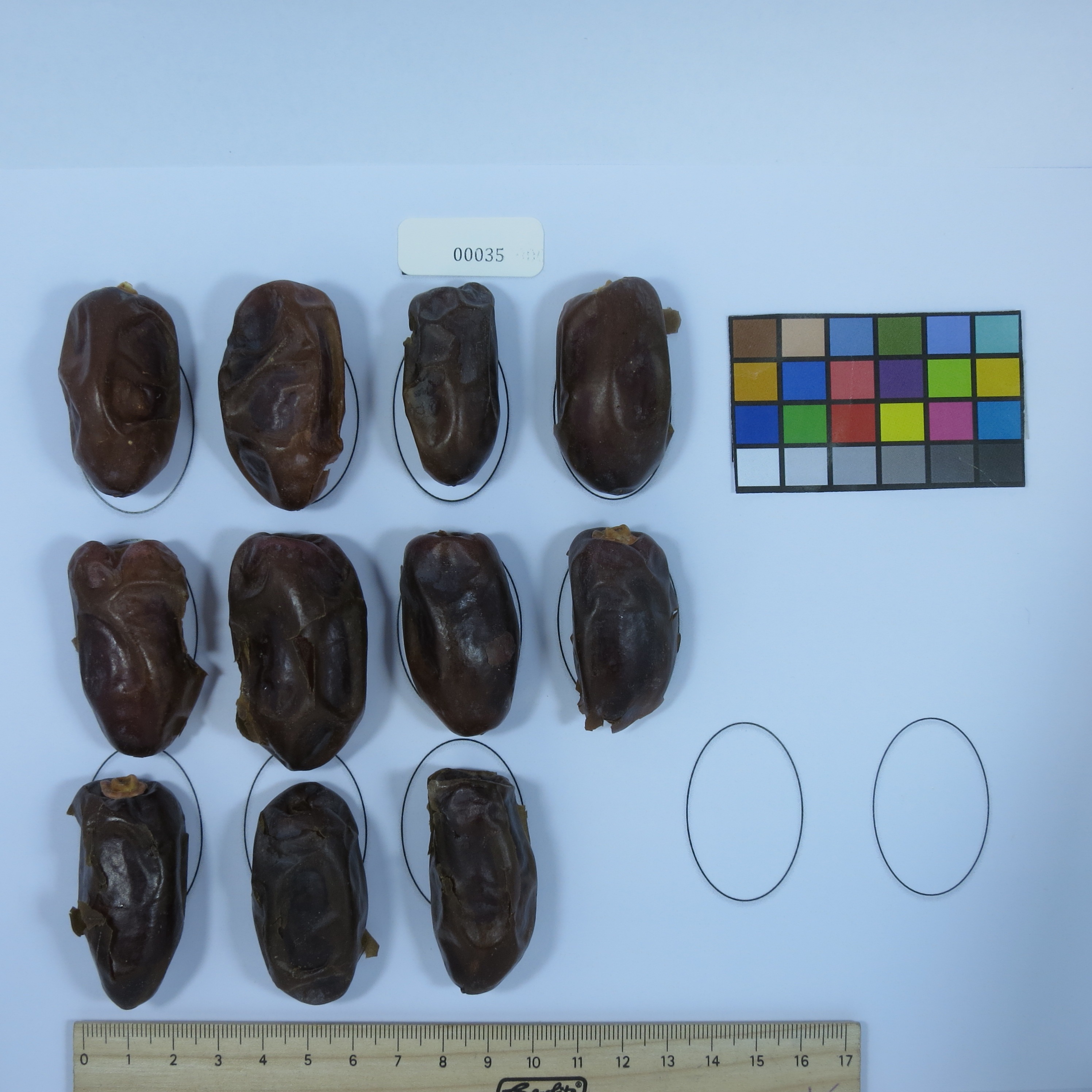

Supplement: Supplementary file 5 — Supplementary material [file mmc5.zip › dates images/00035.JPG]

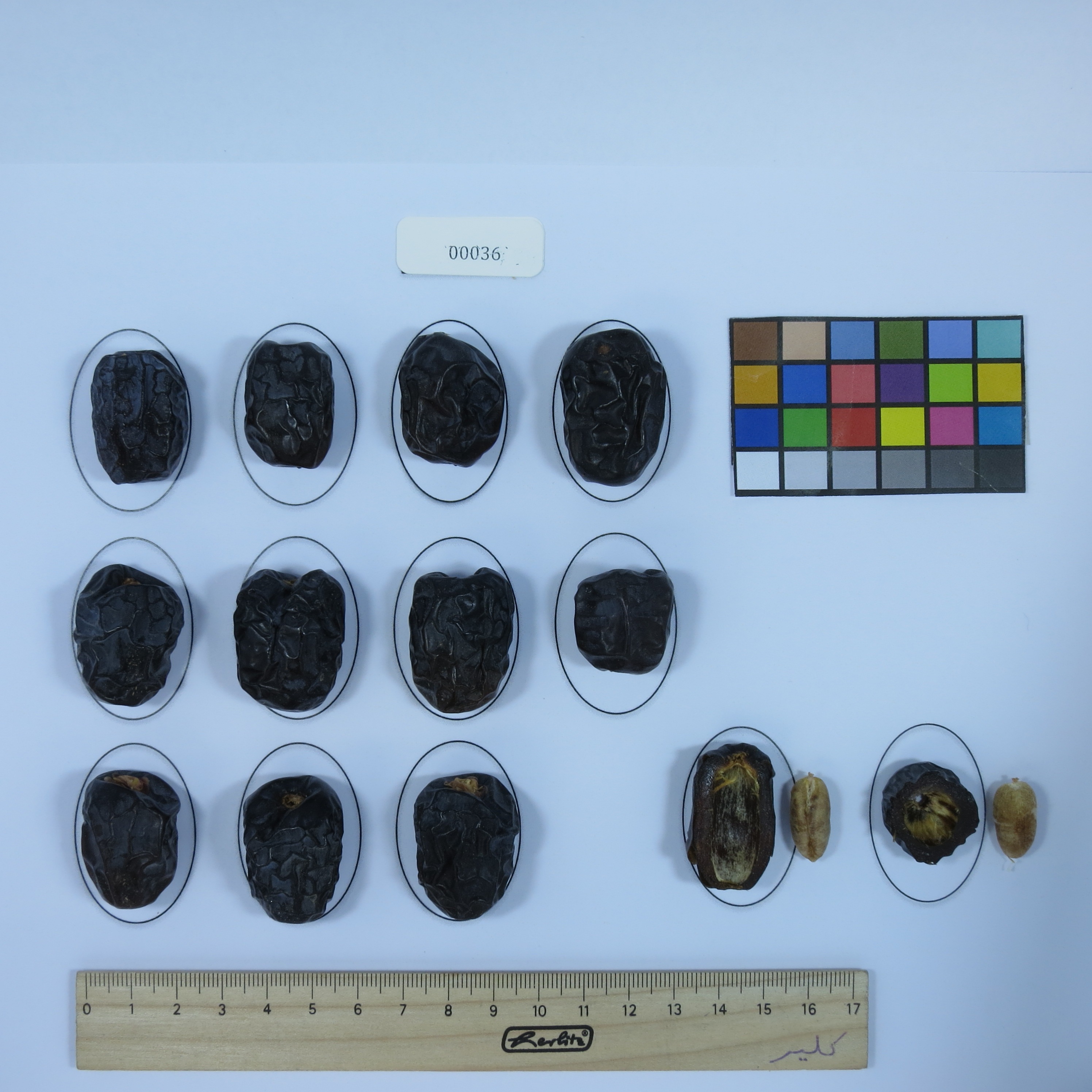

Supplement: Supplementary file 5 — Supplementary material [file mmc5.zip › dates images/00036.JPG]

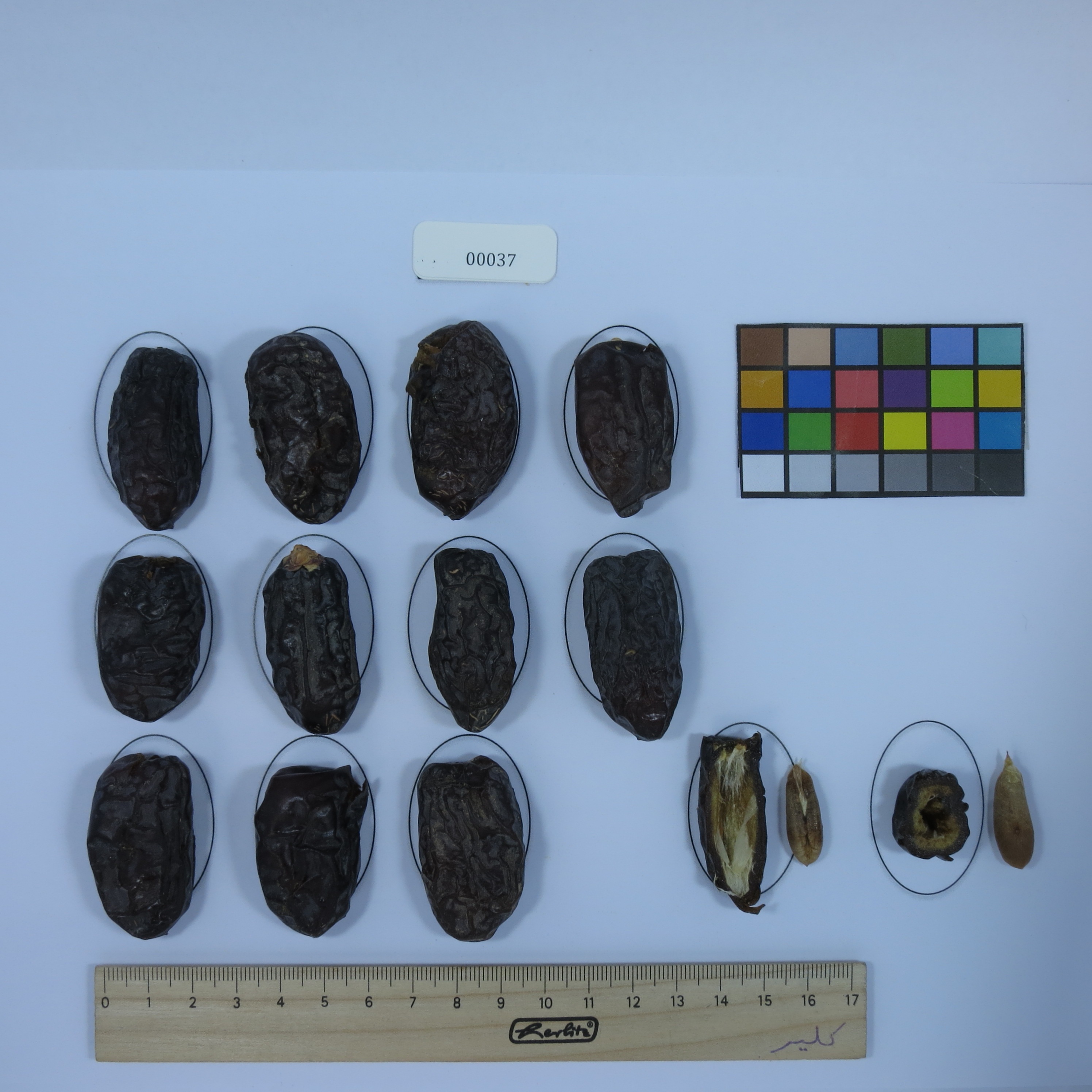

Supplement: Supplementary file 5 — Supplementary material [file mmc5.zip › dates images/00037.JPG]

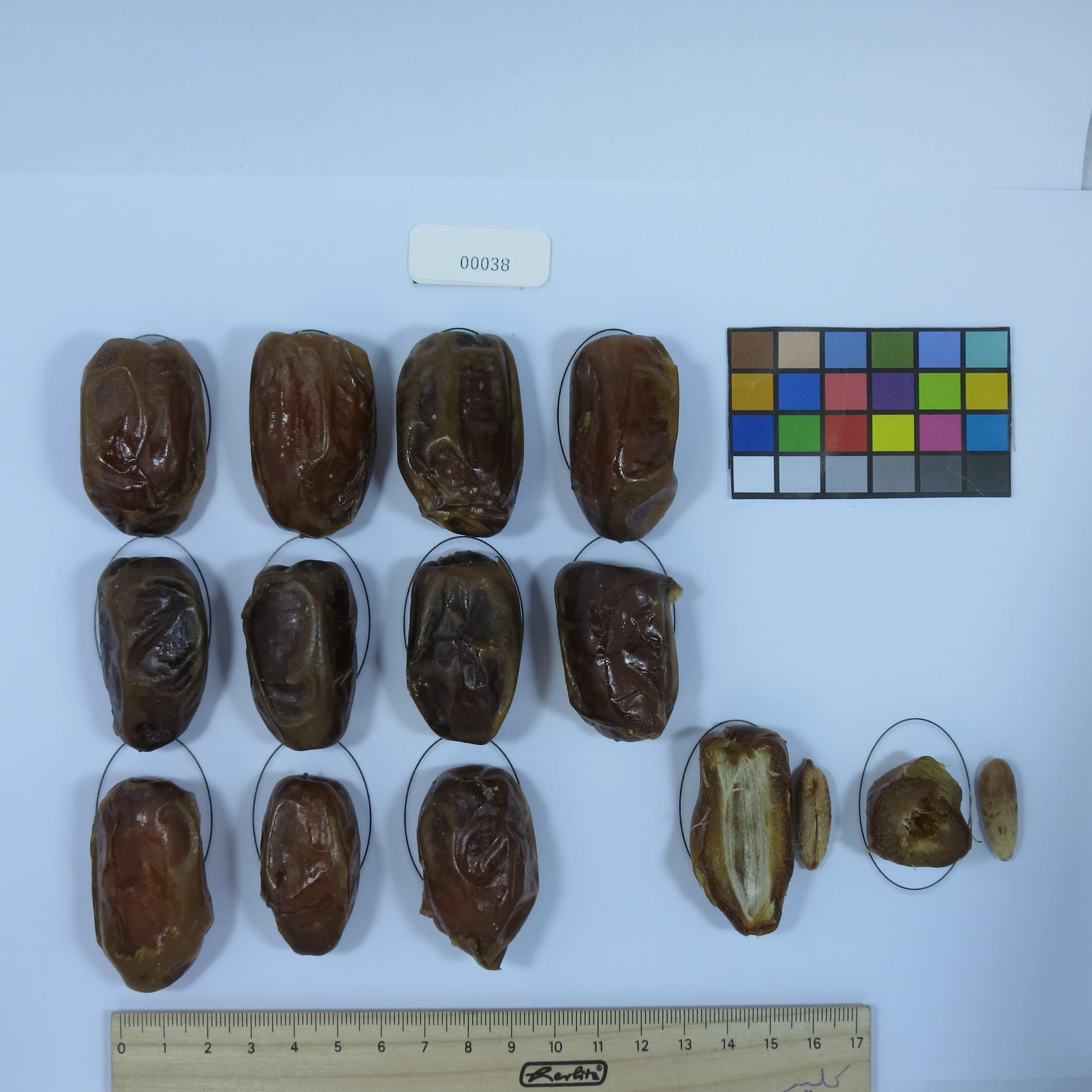

Supplement: Supplementary file 5 — Supplementary material [file mmc5.zip › dates images/00038.JPG]

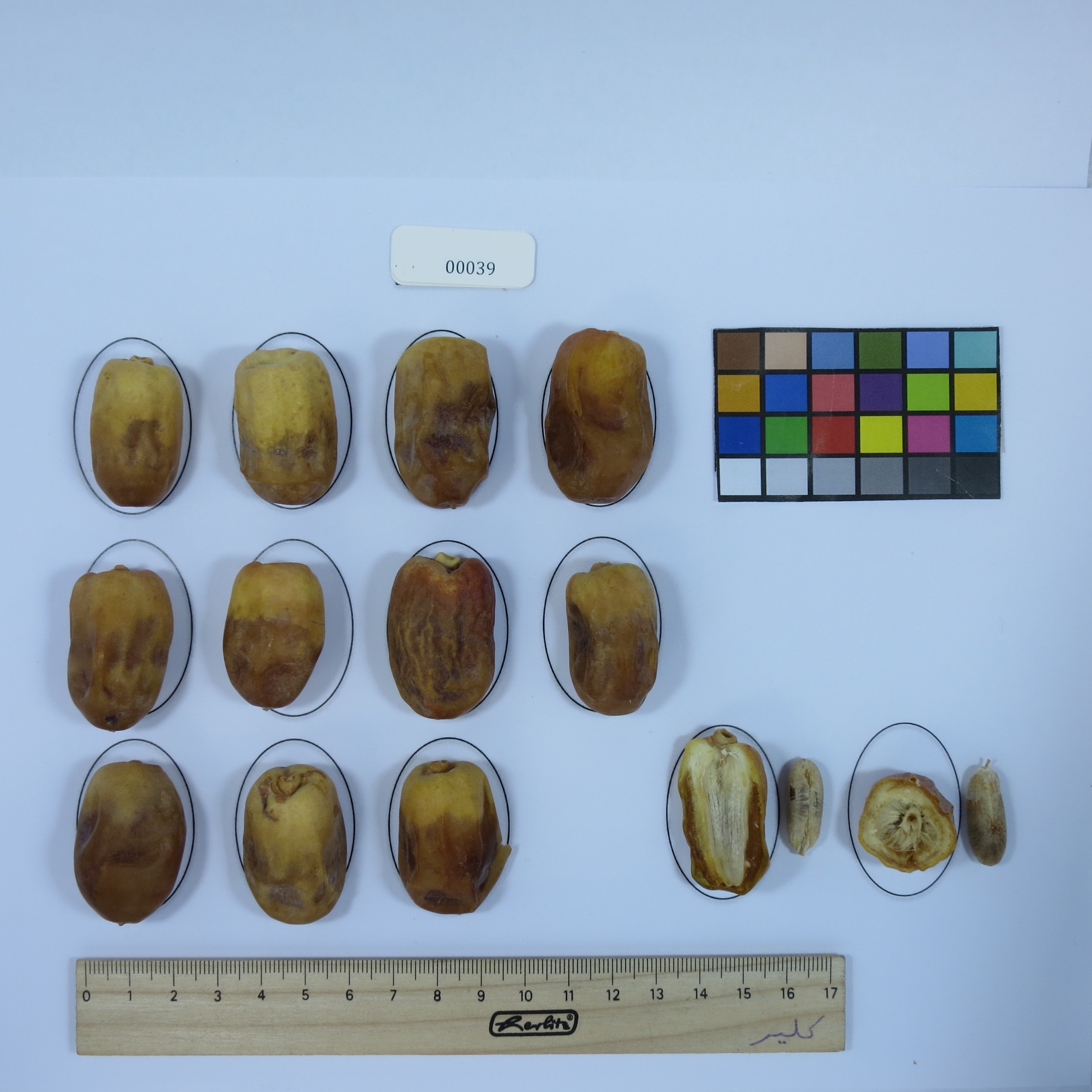

Supplement: Supplementary file 5 — Supplementary material [file mmc5.zip › dates images/00039.JPG]

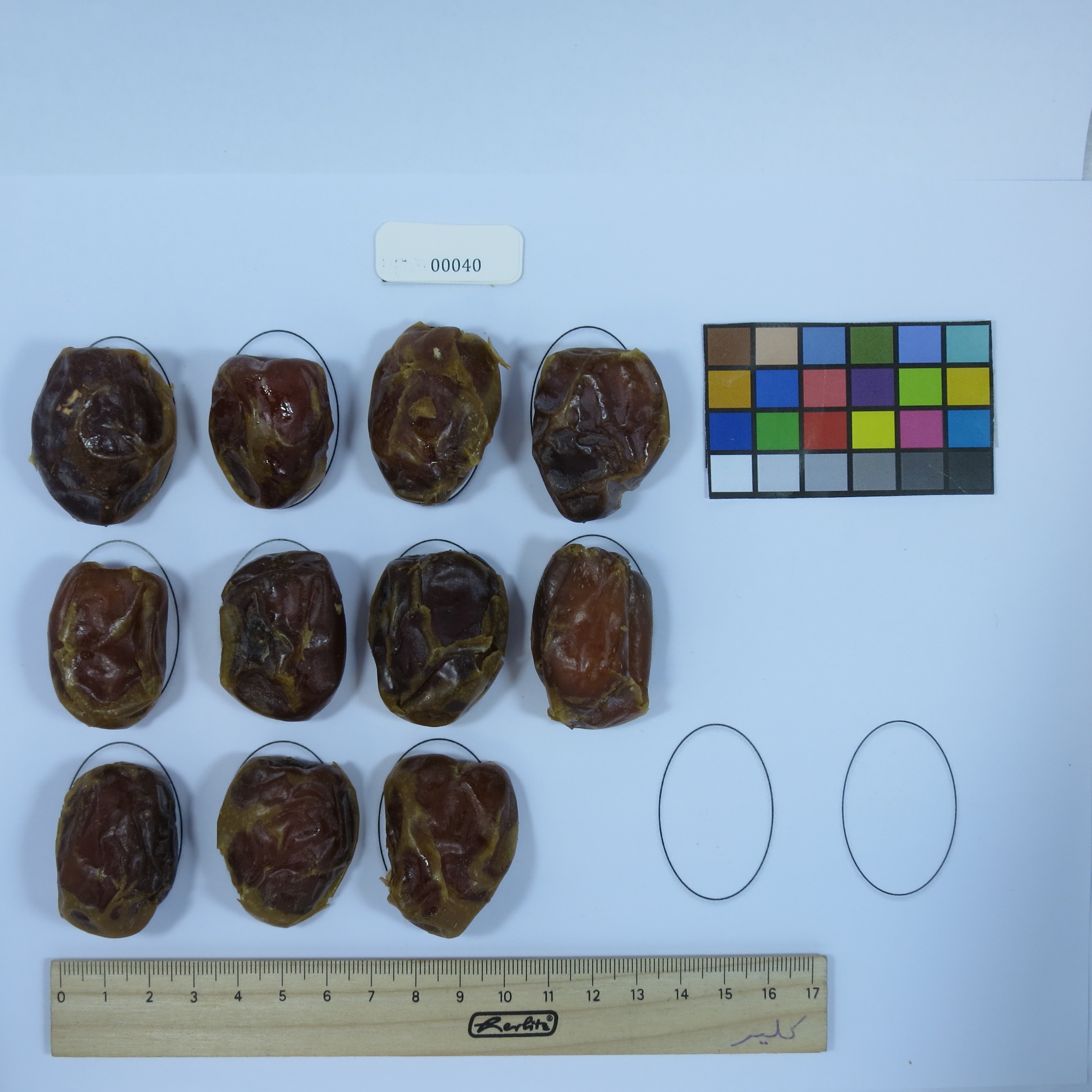

Supplement: Supplementary file 5 — Supplementary material [file mmc5.zip › dates images/00040.JPG]

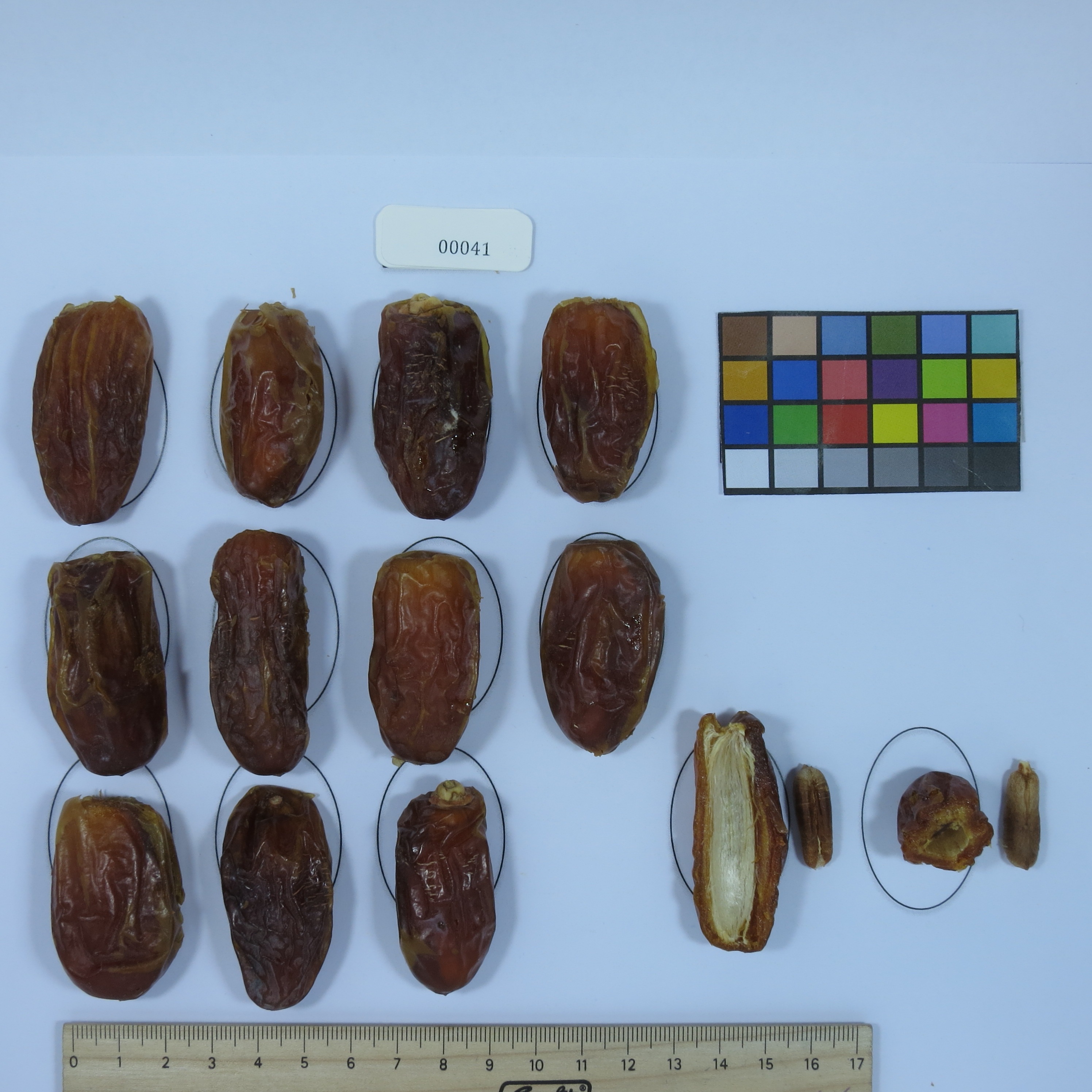

Supplement: Supplementary file 5 — Supplementary material [file mmc5.zip › dates images/00041.JPG]

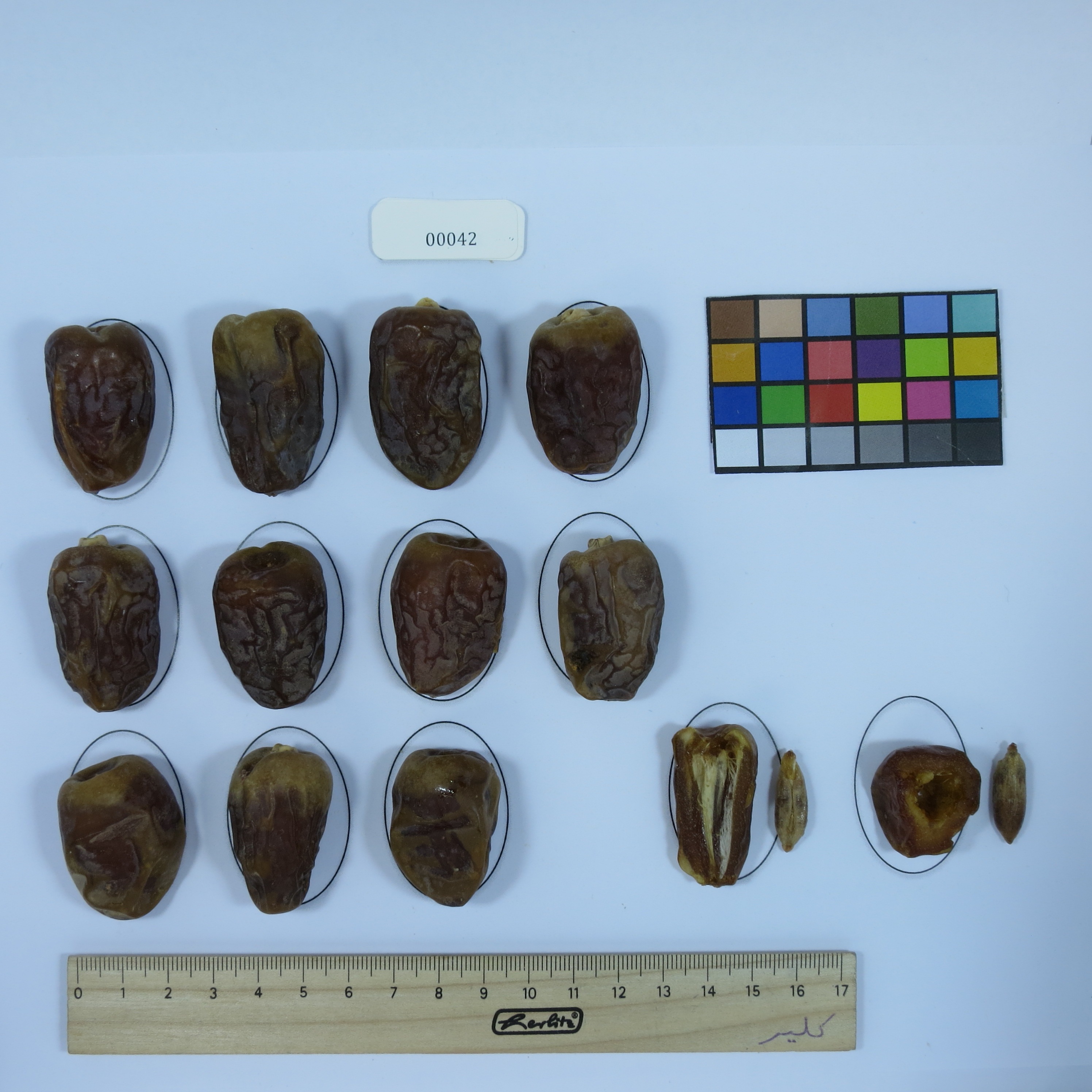

Supplement: Supplementary file 5 — Supplementary material [file mmc5.zip › dates images/00042.JPG]

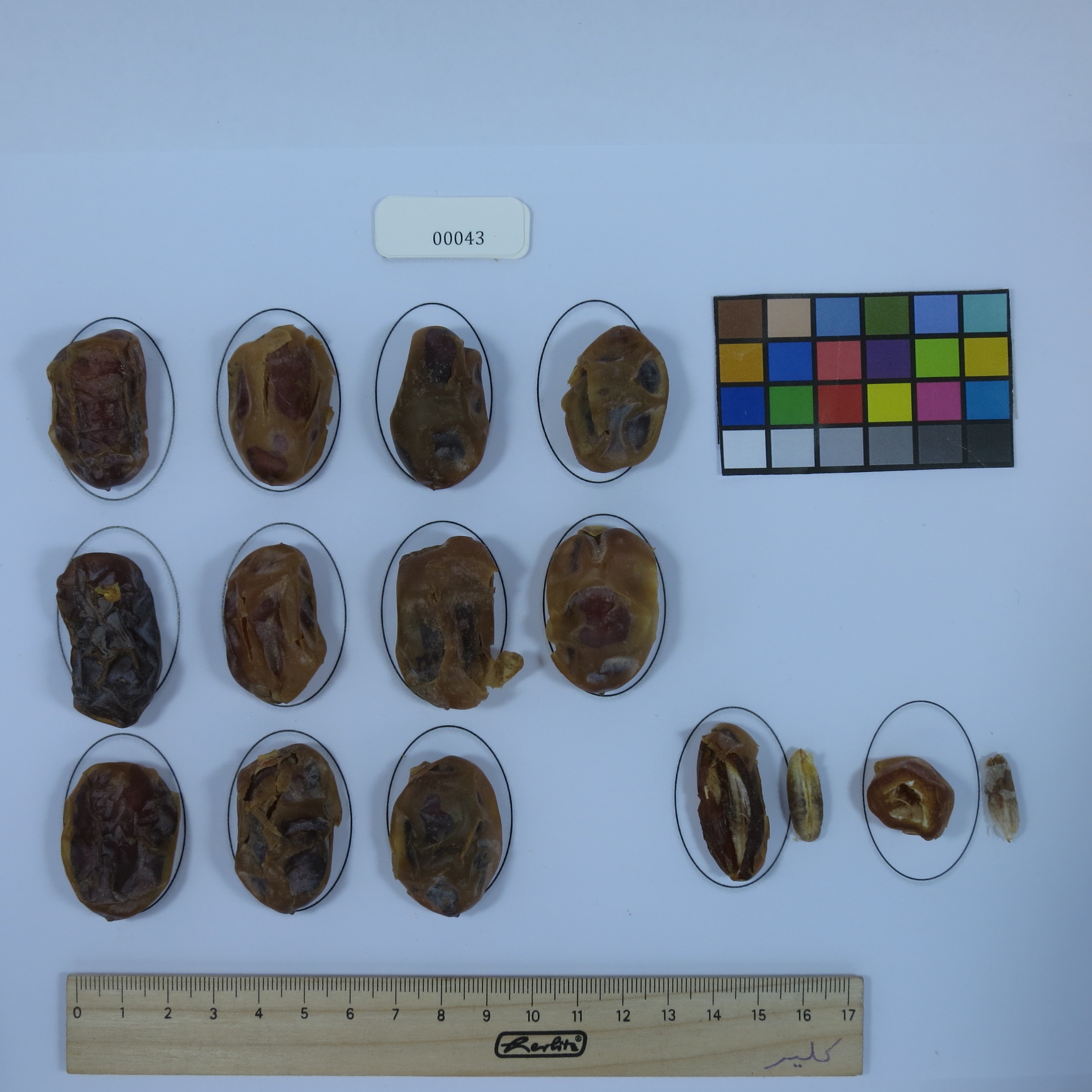

Supplement: Supplementary file 5 — Supplementary material [file mmc5.zip › dates images/00043.JPG]

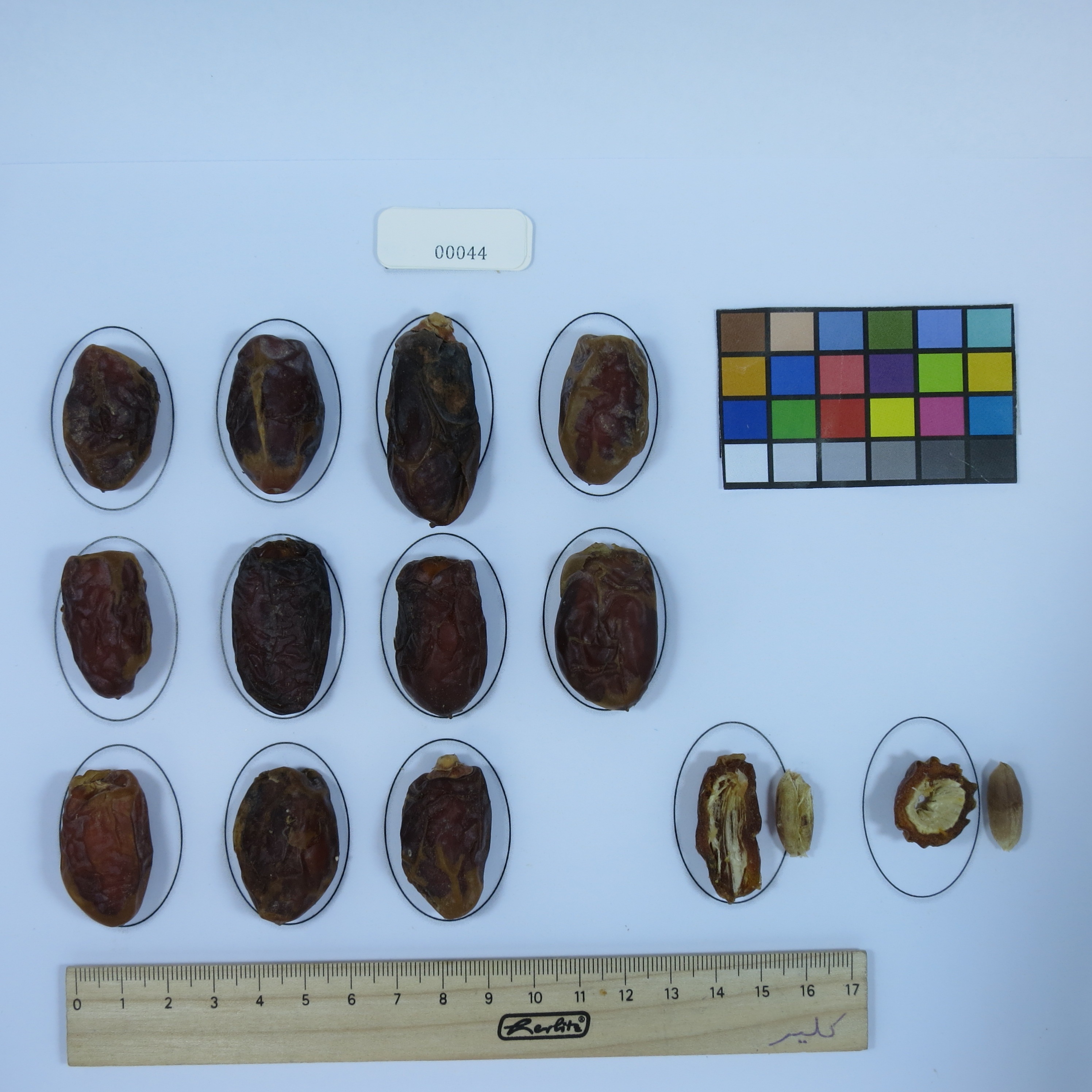

Supplement: Supplementary file 5 — Supplementary material [file mmc5.zip › dates images/00044.JPG]

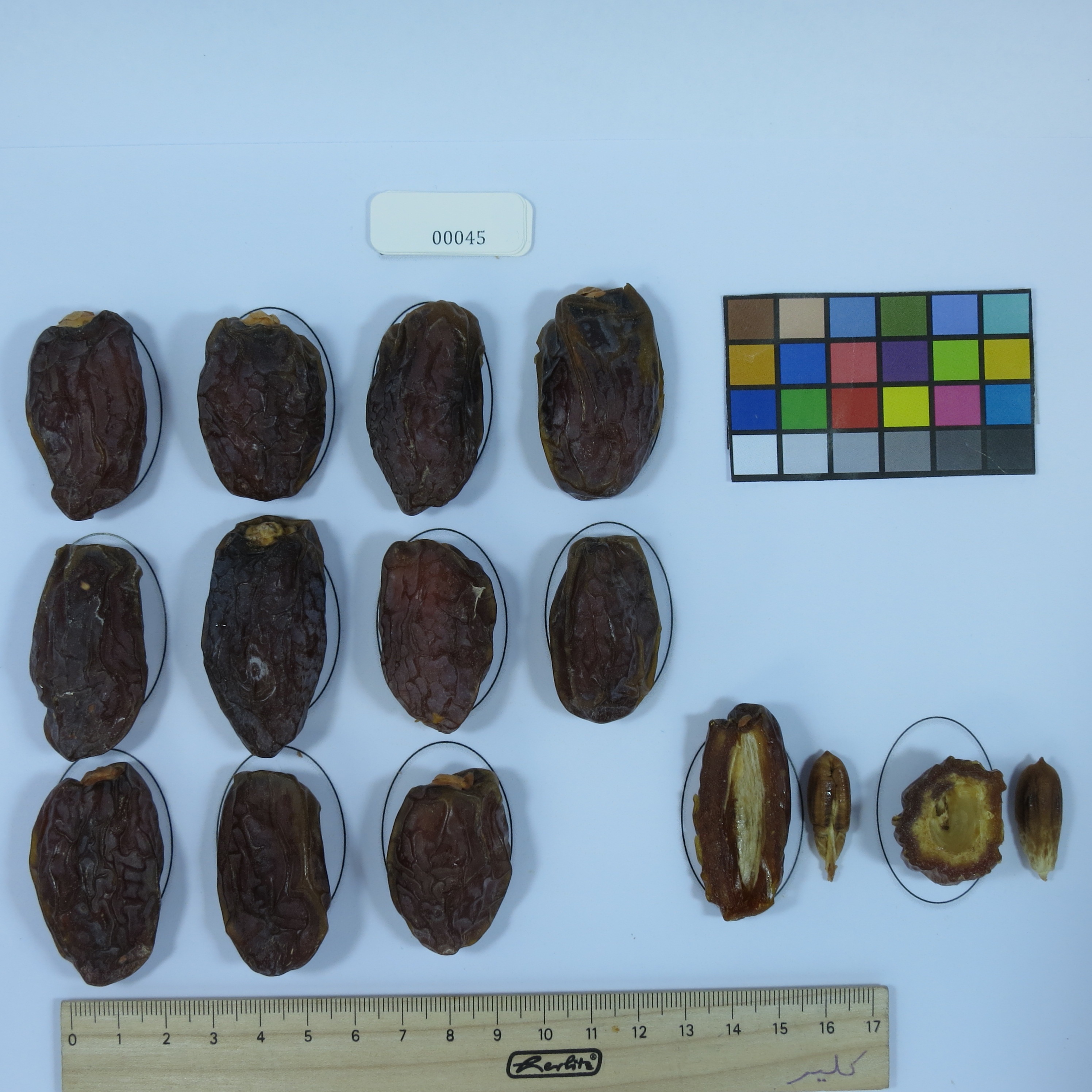

Supplement: Supplementary file 5 — Supplementary material [file mmc5.zip › dates images/00045.JPG]

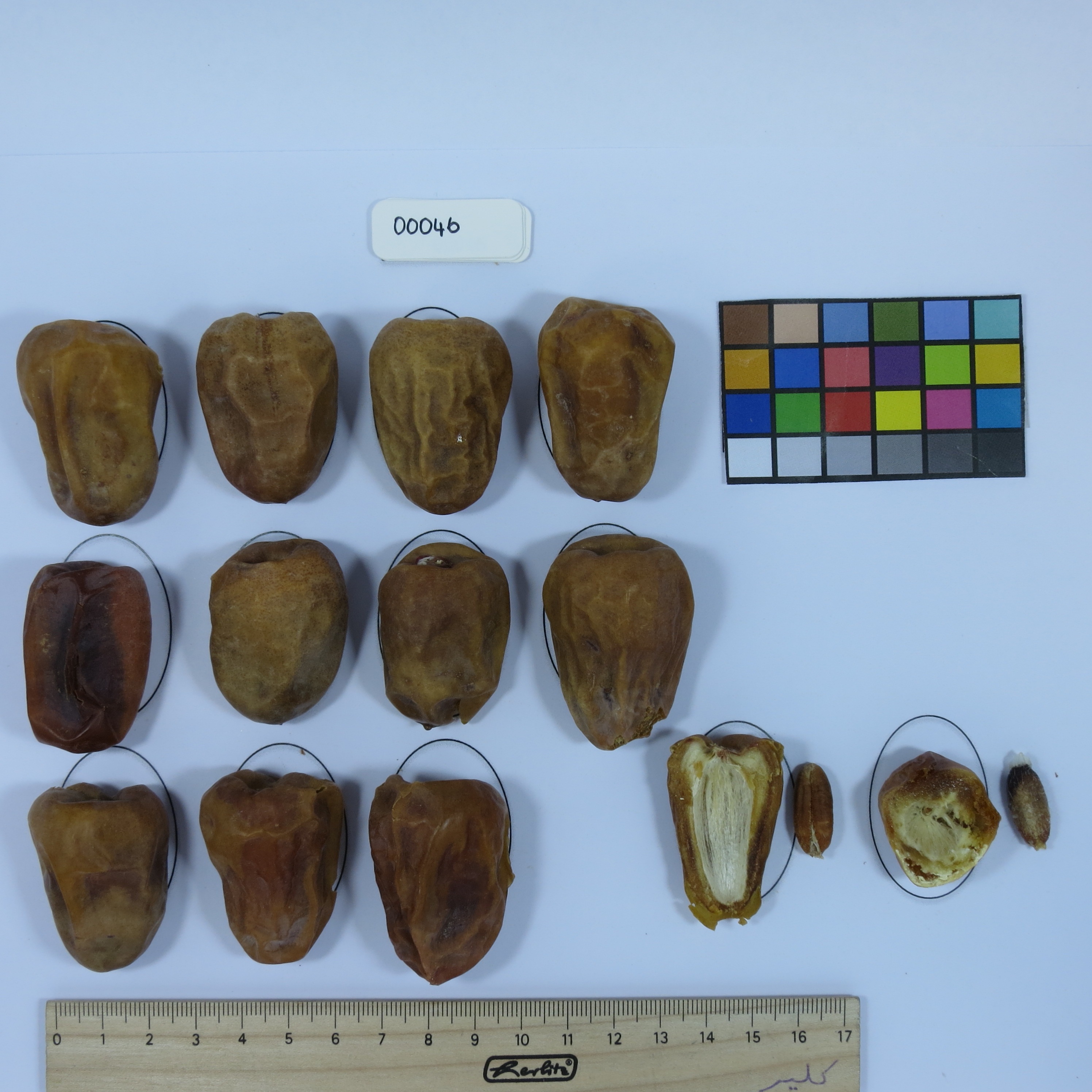

Supplement: Supplementary file 5 — Supplementary material [file mmc5.zip › dates images/00046.JPG]

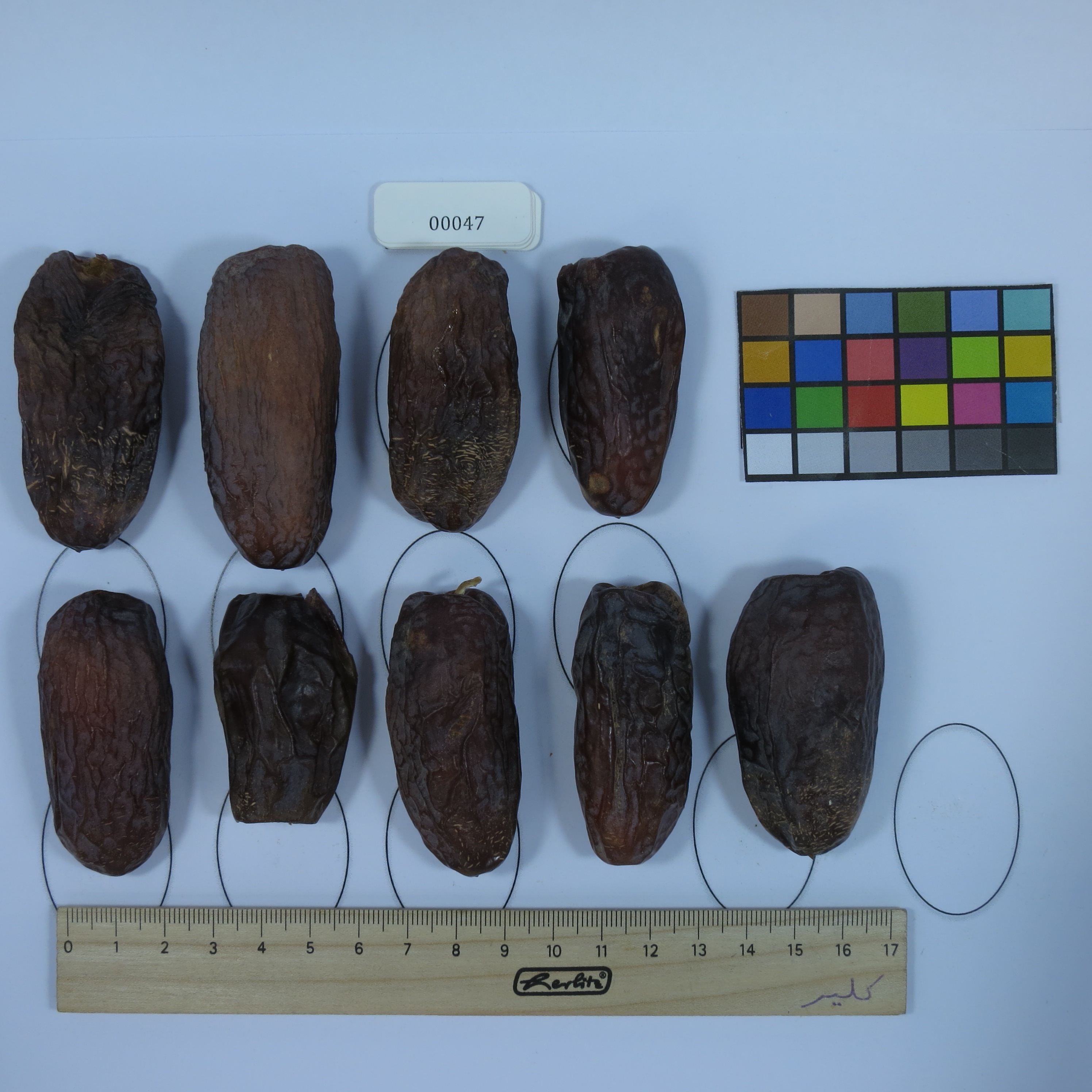

Supplement: Supplementary file 5 — Supplementary material [file mmc5.zip › dates images/00047.JPG]

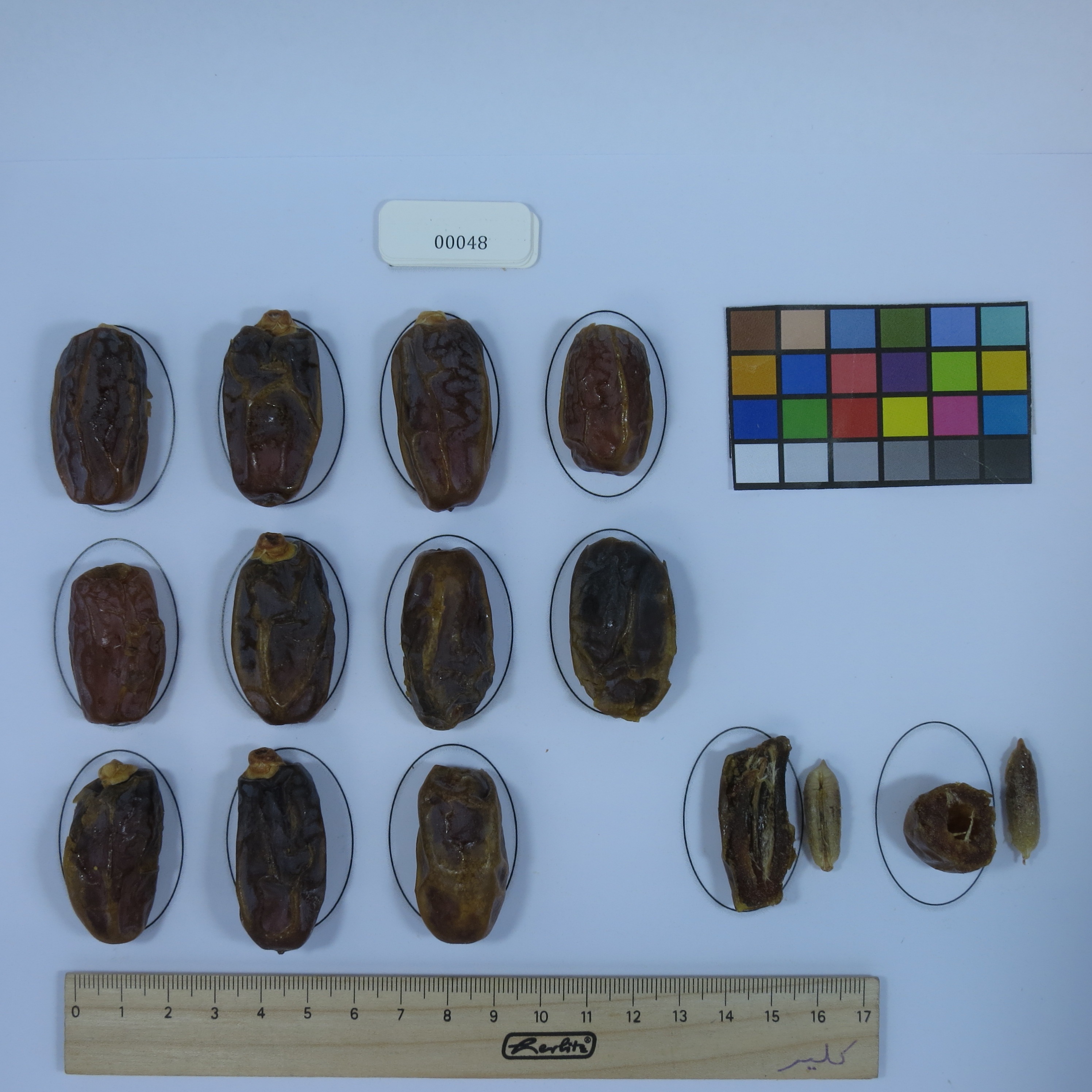

Supplement: Supplementary file 5 — Supplementary material [file mmc5.zip › dates images/00048.JPG]

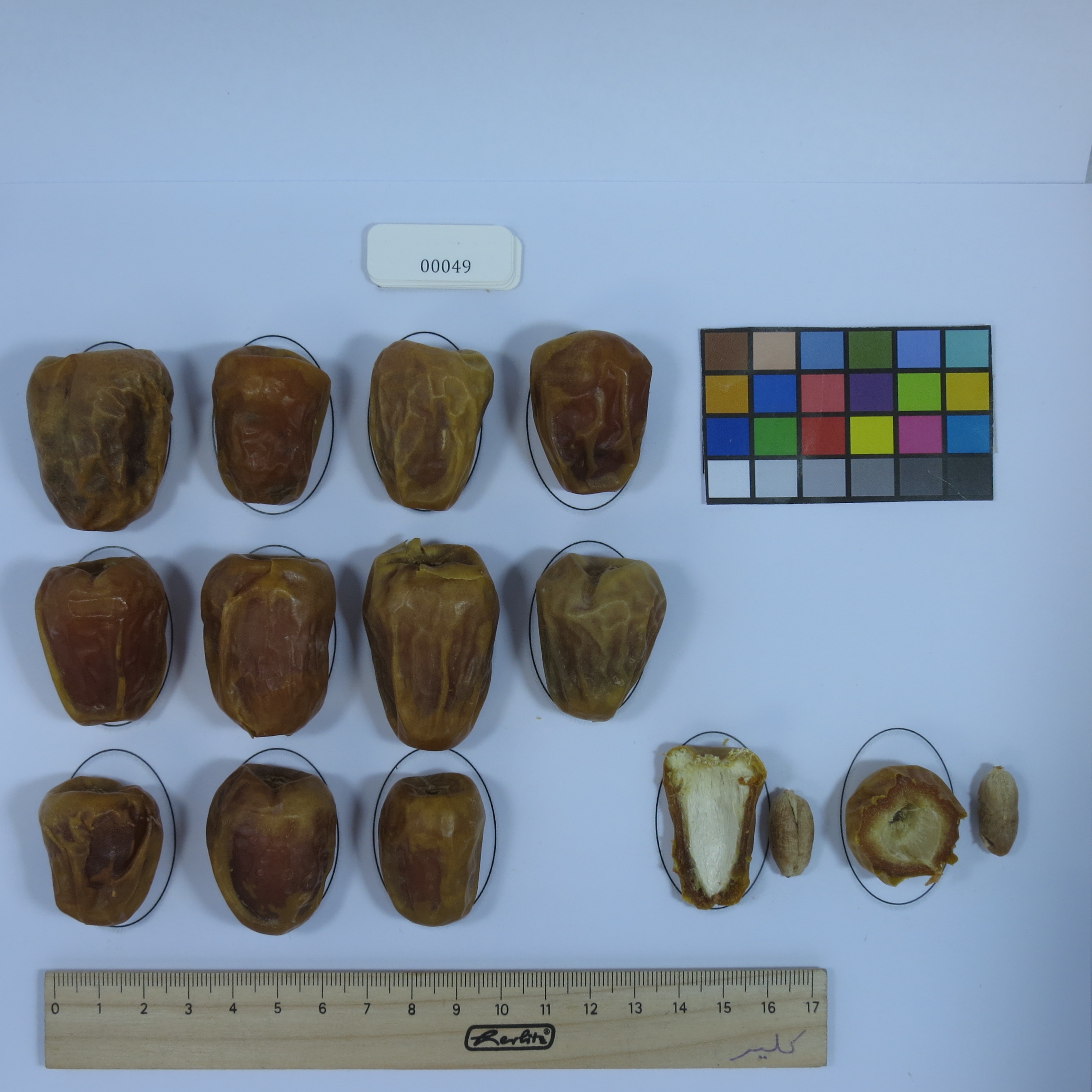

Supplement: Supplementary file 5 — Supplementary material [file mmc5.zip › dates images/00049.JPG]

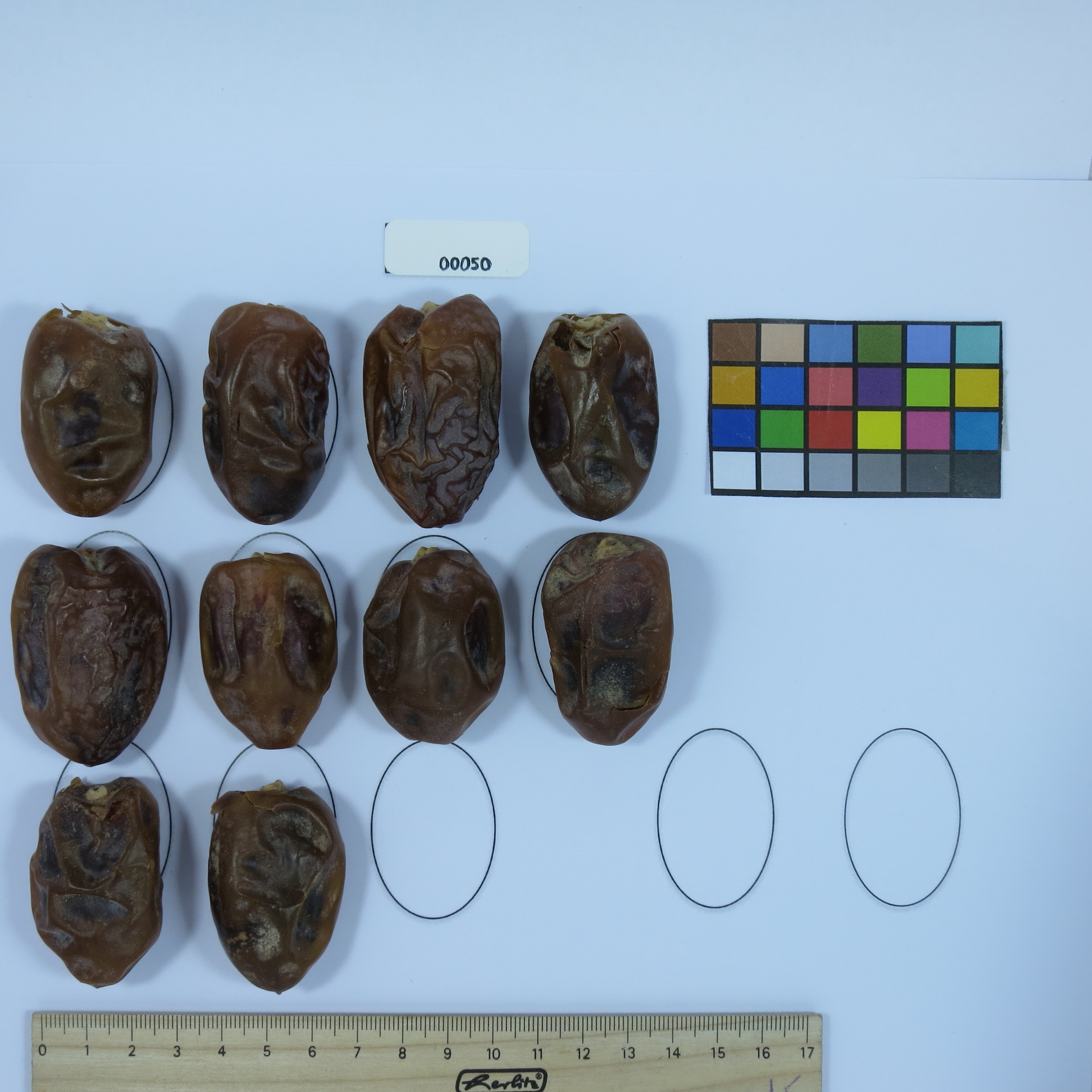

Supplement: Supplementary file 5 — Supplementary material [file mmc5.zip › dates images/00050.JPG]

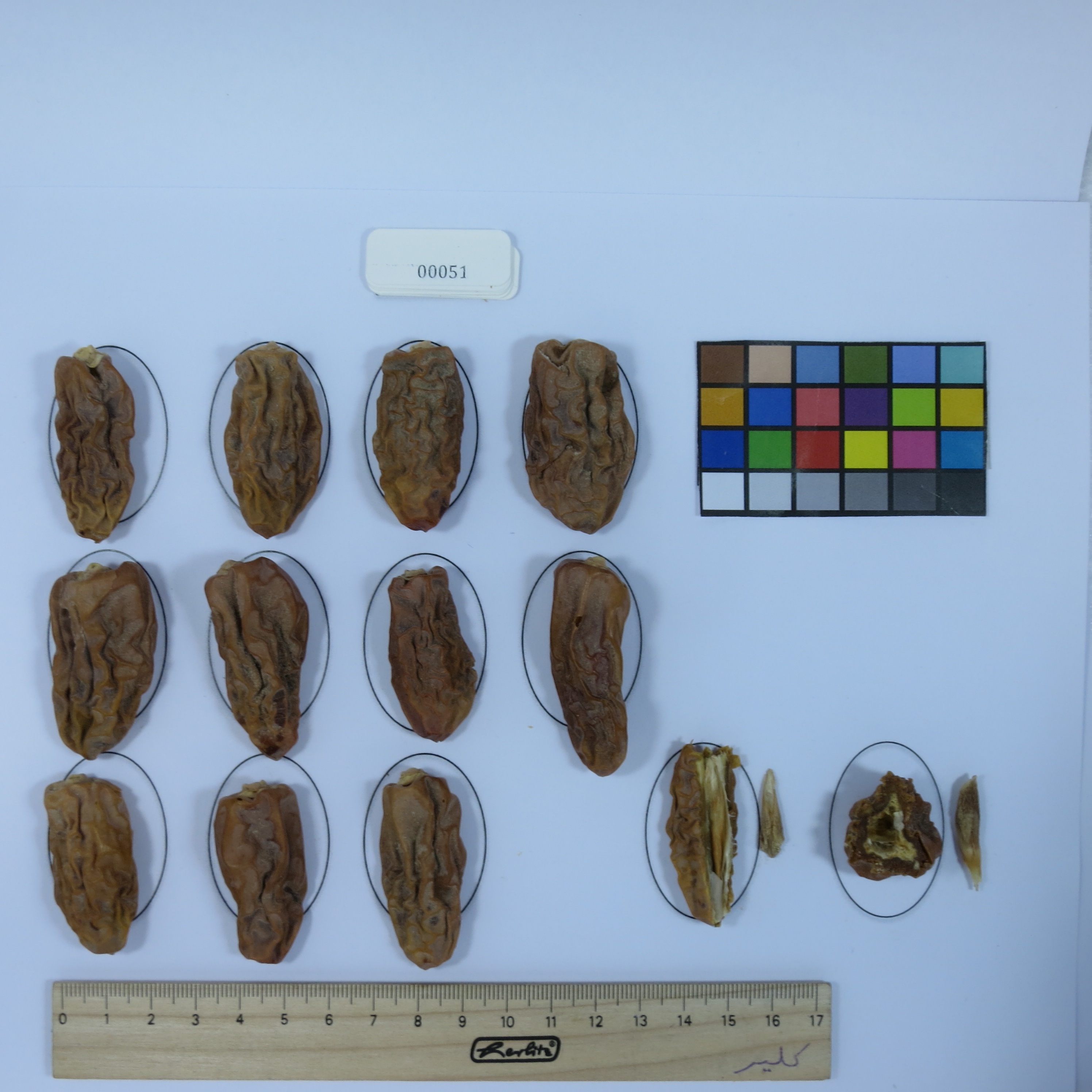

Supplement: Supplementary file 5 — Supplementary material [file mmc5.zip › dates images/00051.JPG]

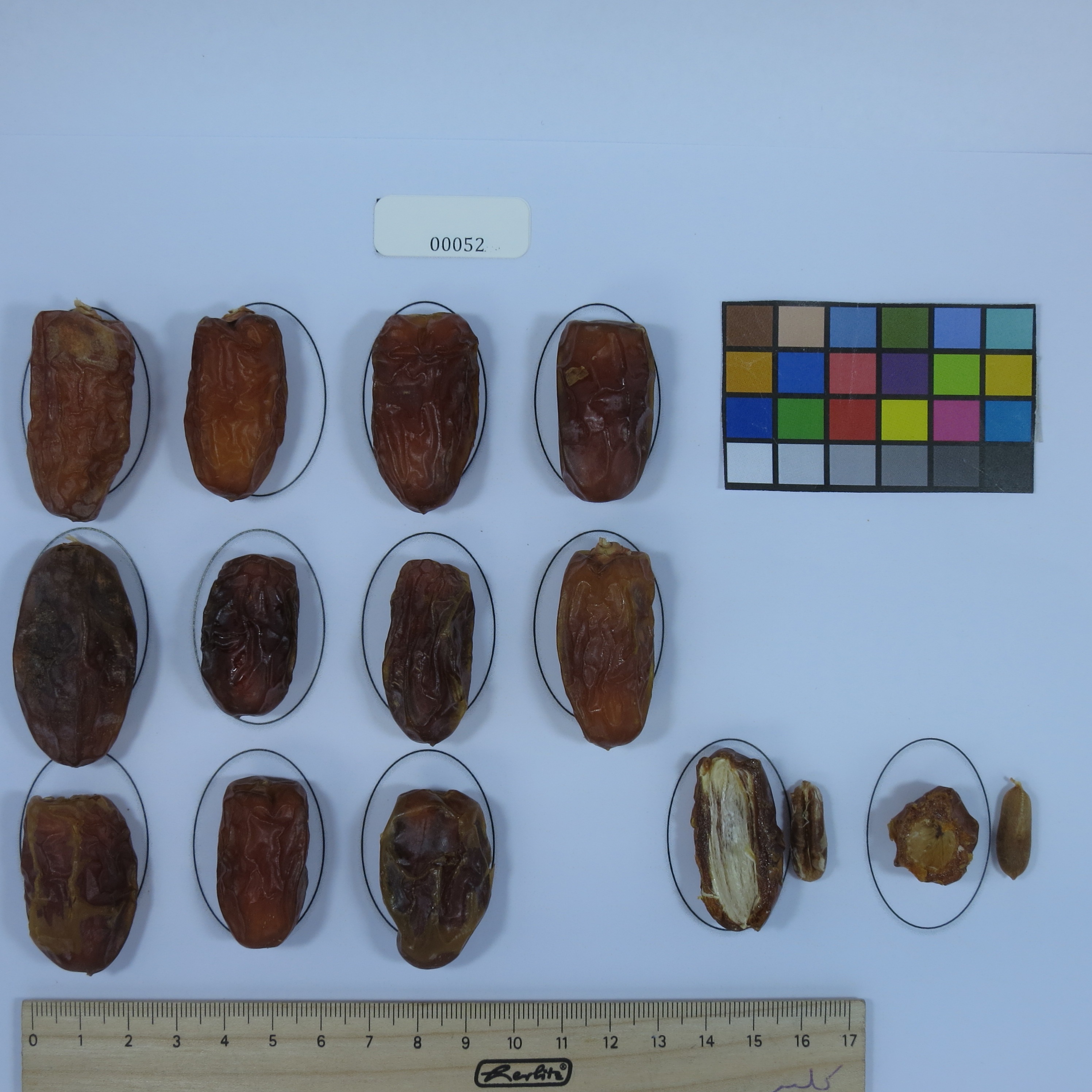

Supplement: Supplementary file 5 — Supplementary material [file mmc5.zip › dates images/00052.JPG]

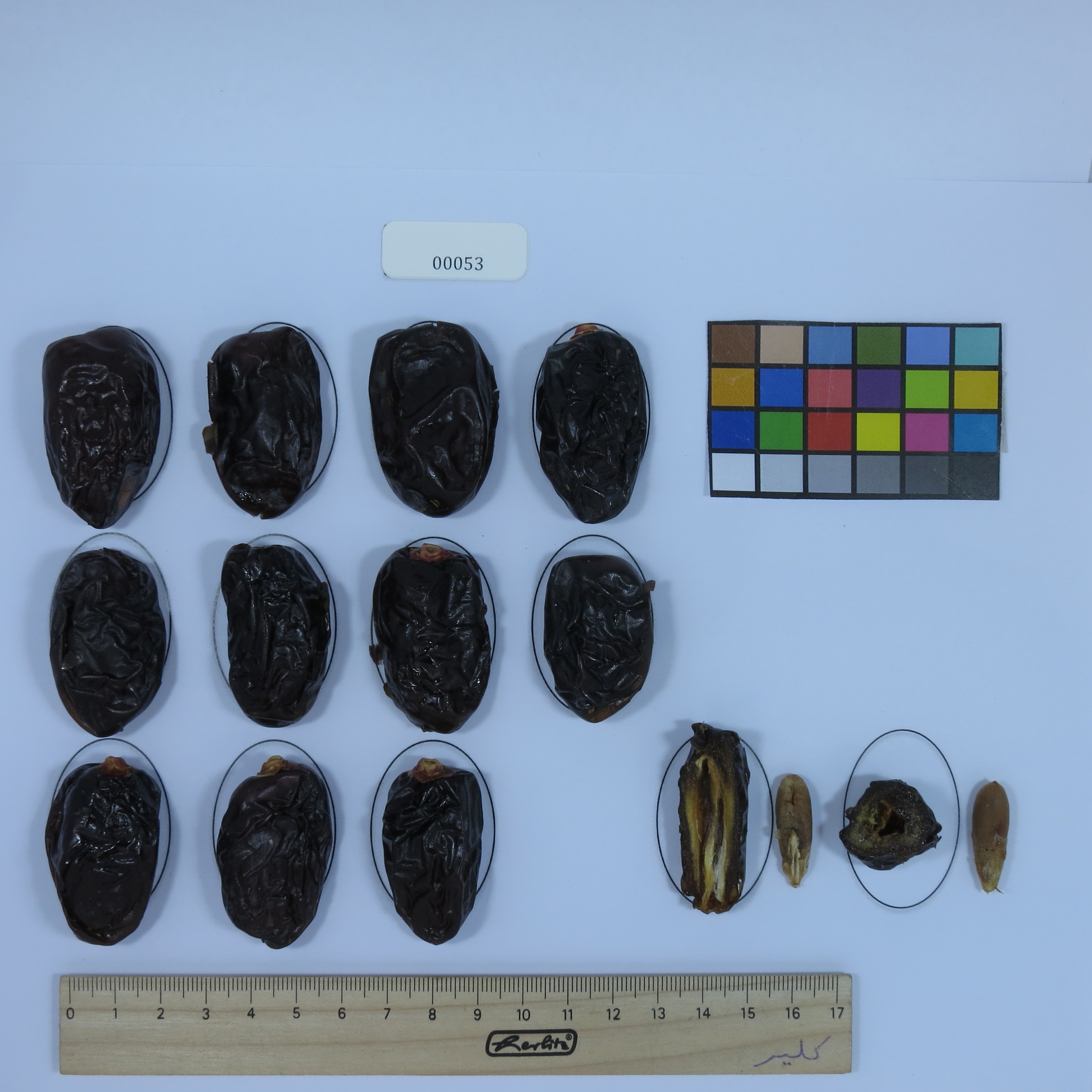

Supplement: Supplementary file 5 — Supplementary material [file mmc5.zip › dates images/00053.JPG]

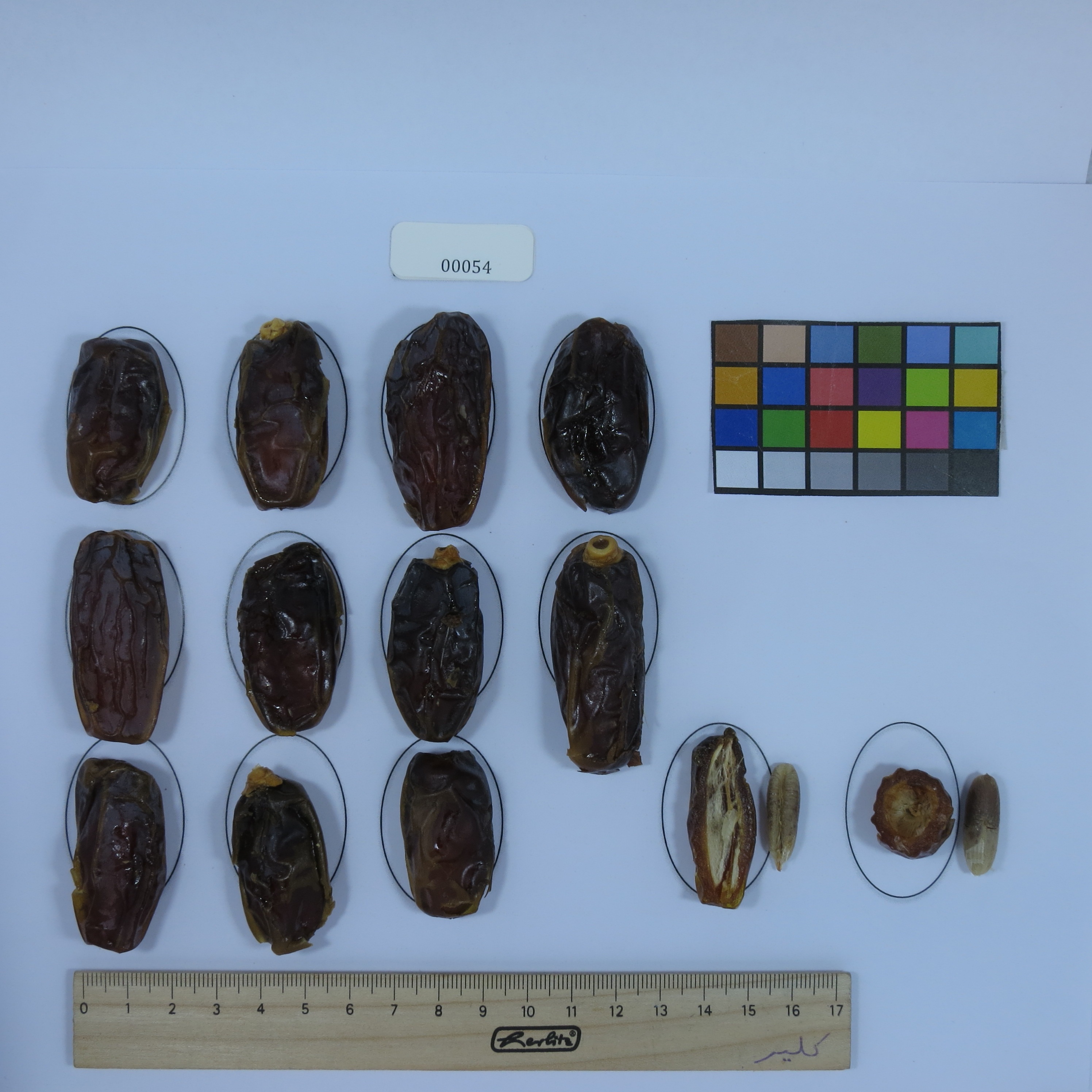

Supplement: Supplementary file 5 — Supplementary material [file mmc5.zip › dates images/00054.JPG]

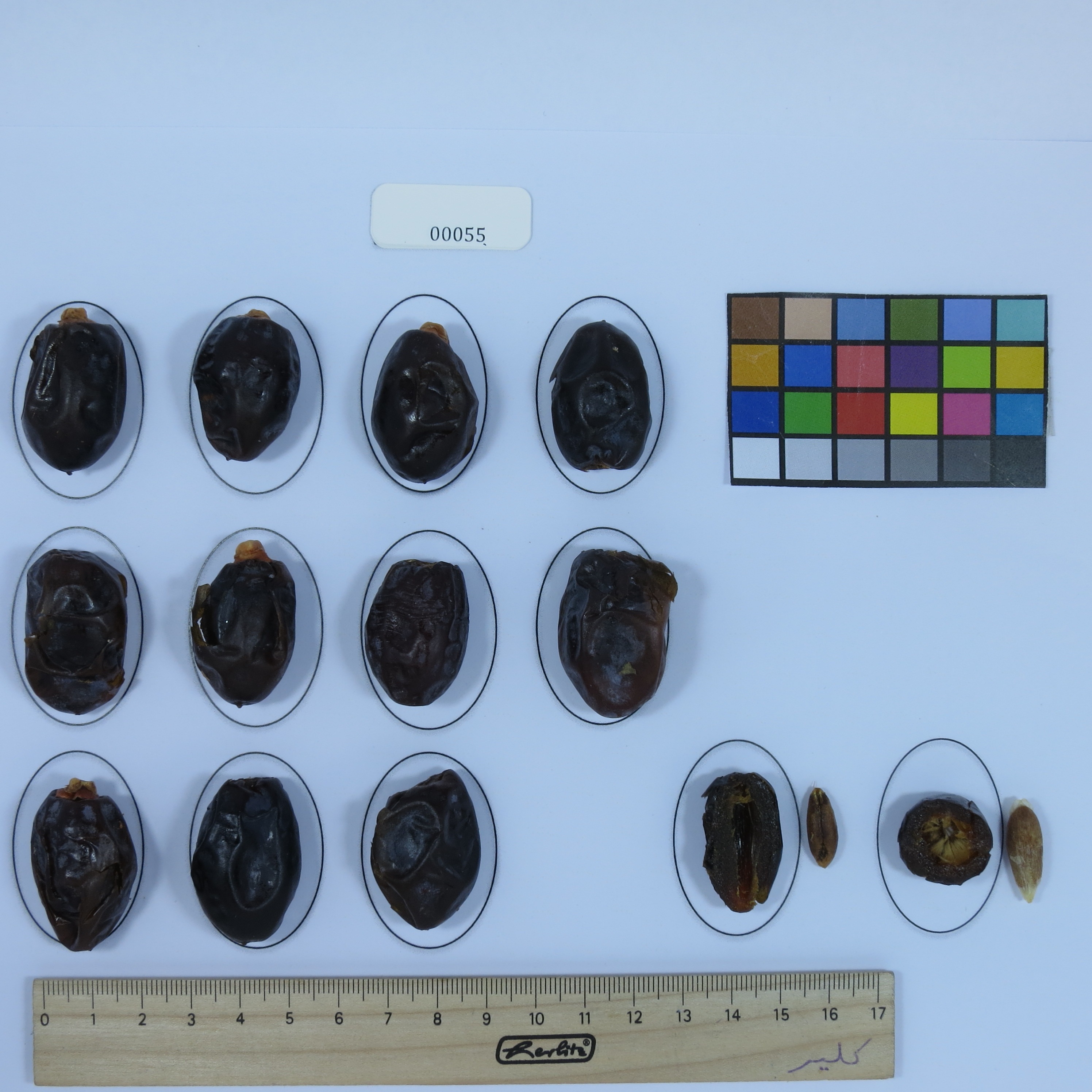

Supplement: Supplementary file 5 — Supplementary material [file mmc5.zip › dates images/00055.JPG]

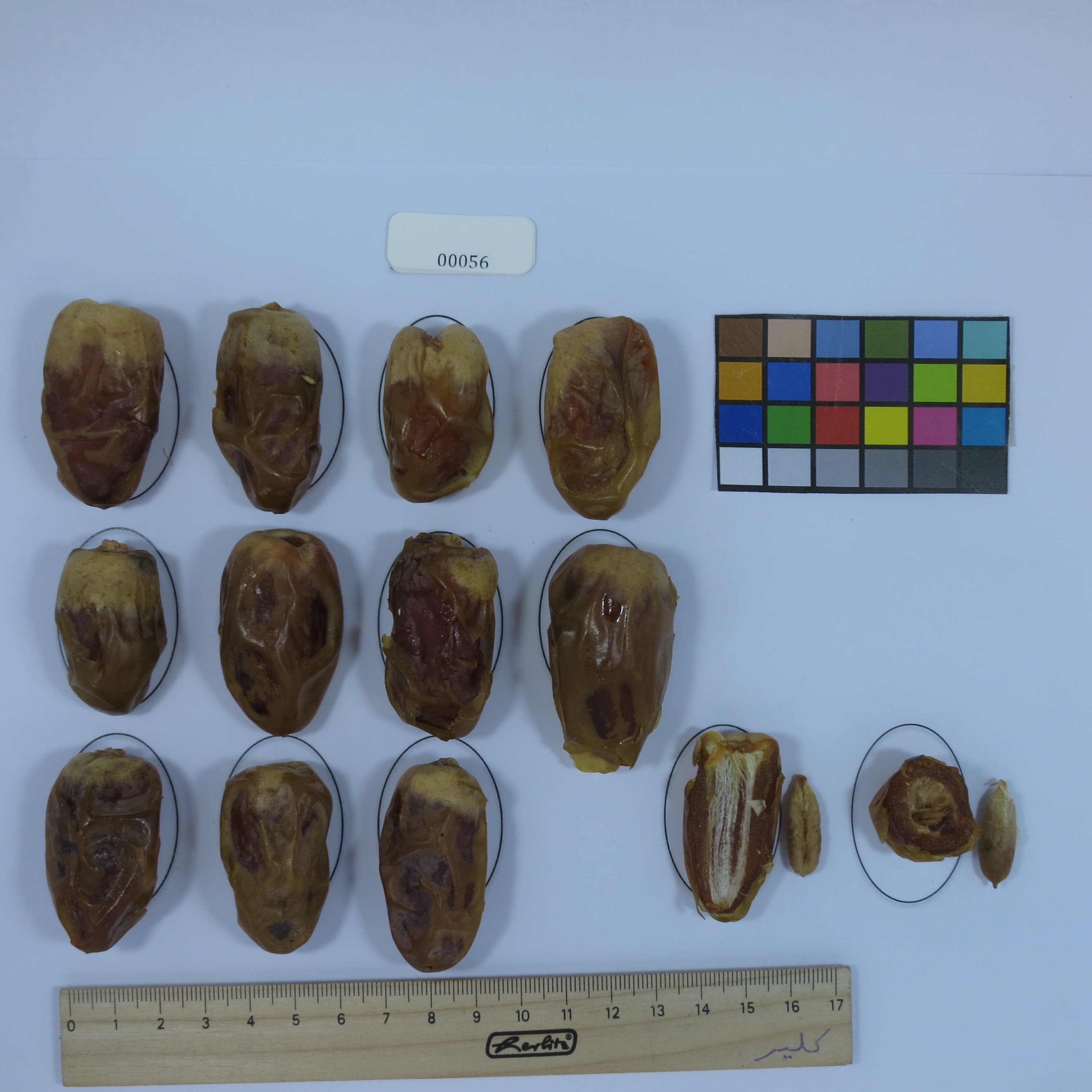

Supplement: Supplementary file 5 — Supplementary material [file mmc5.zip › dates images/00056.JPG]

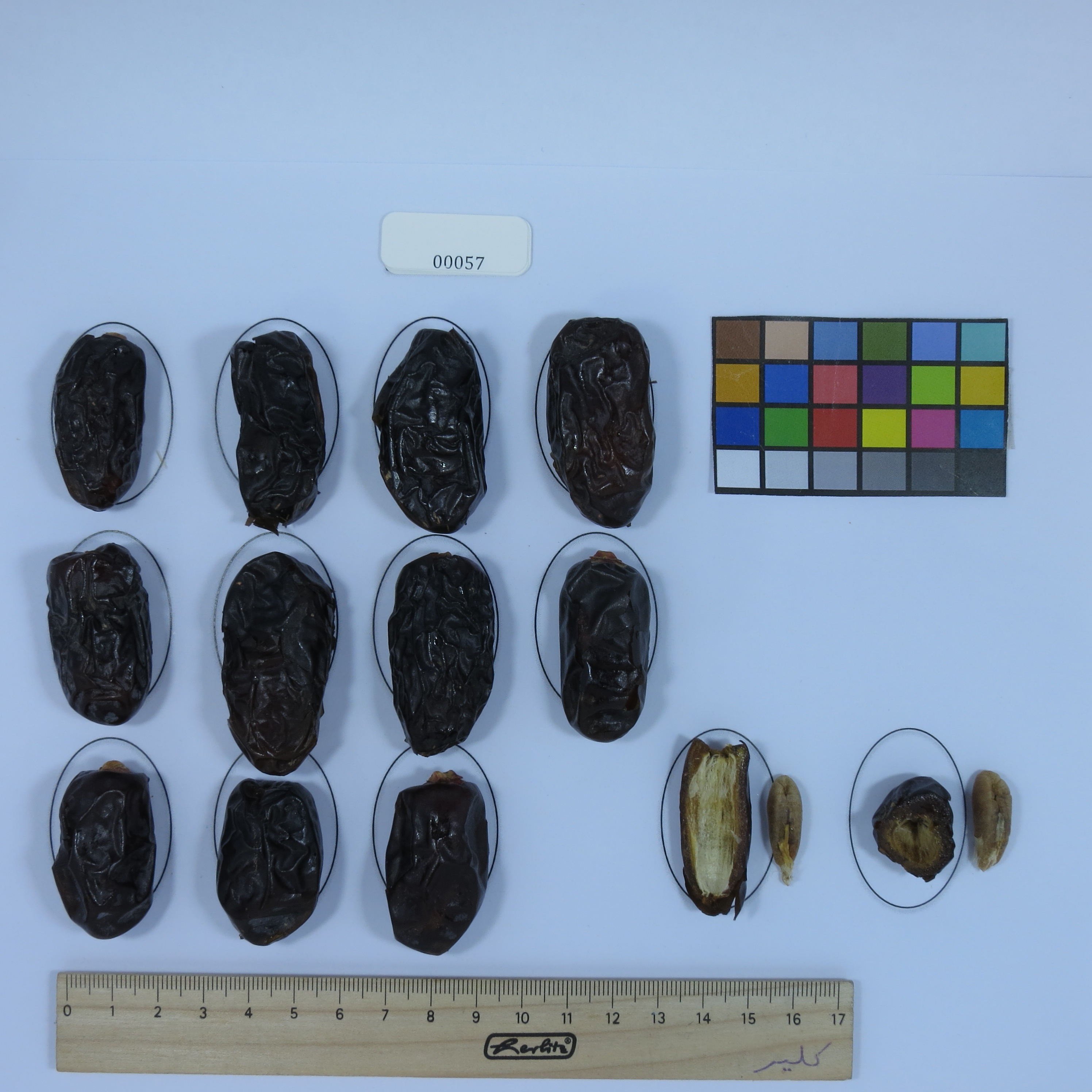

Supplement: Supplementary file 5 — Supplementary material [file mmc5.zip › dates images/00057.JPG]

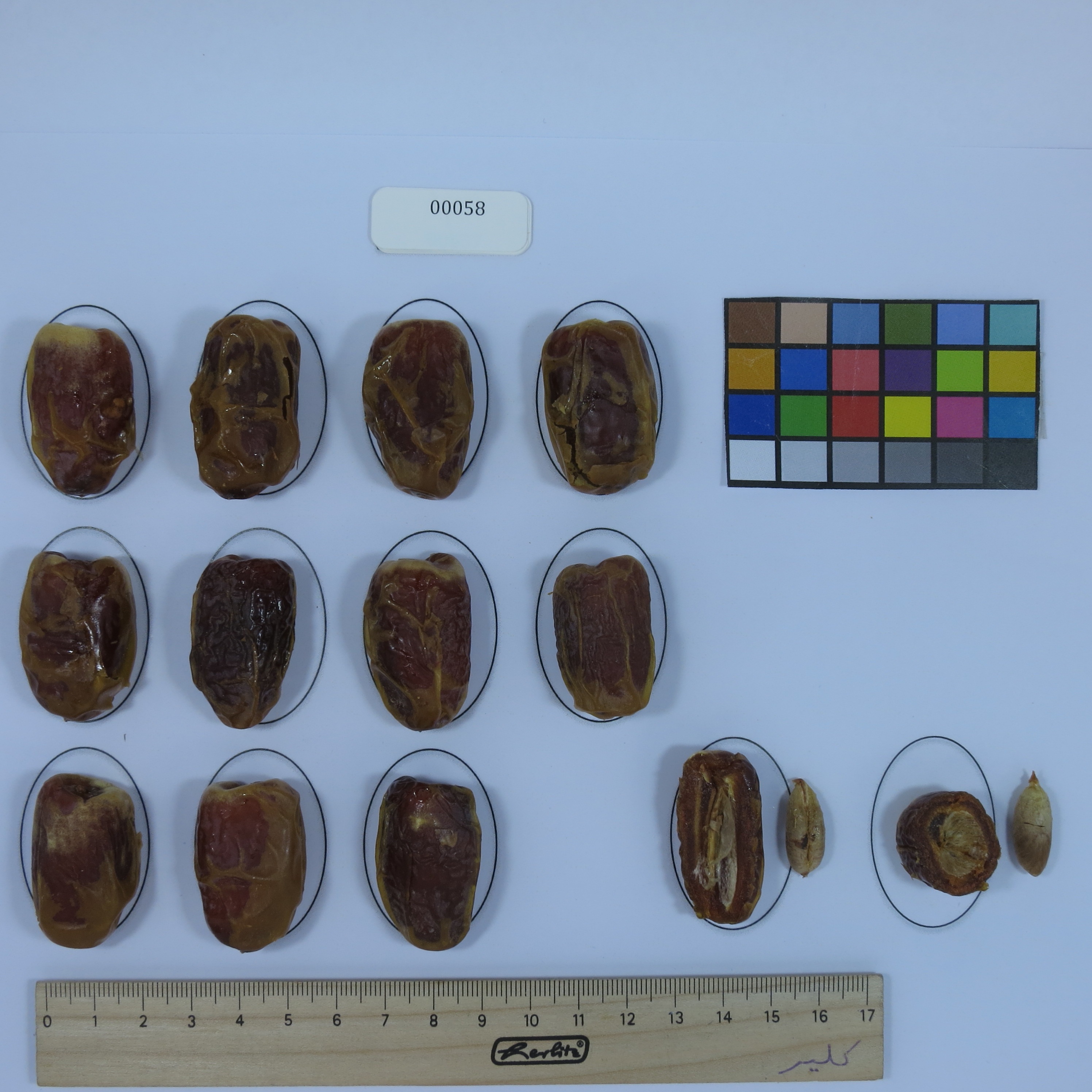

Supplement: Supplementary file 5 — Supplementary material [file mmc5.zip › dates images/00058.JPG]

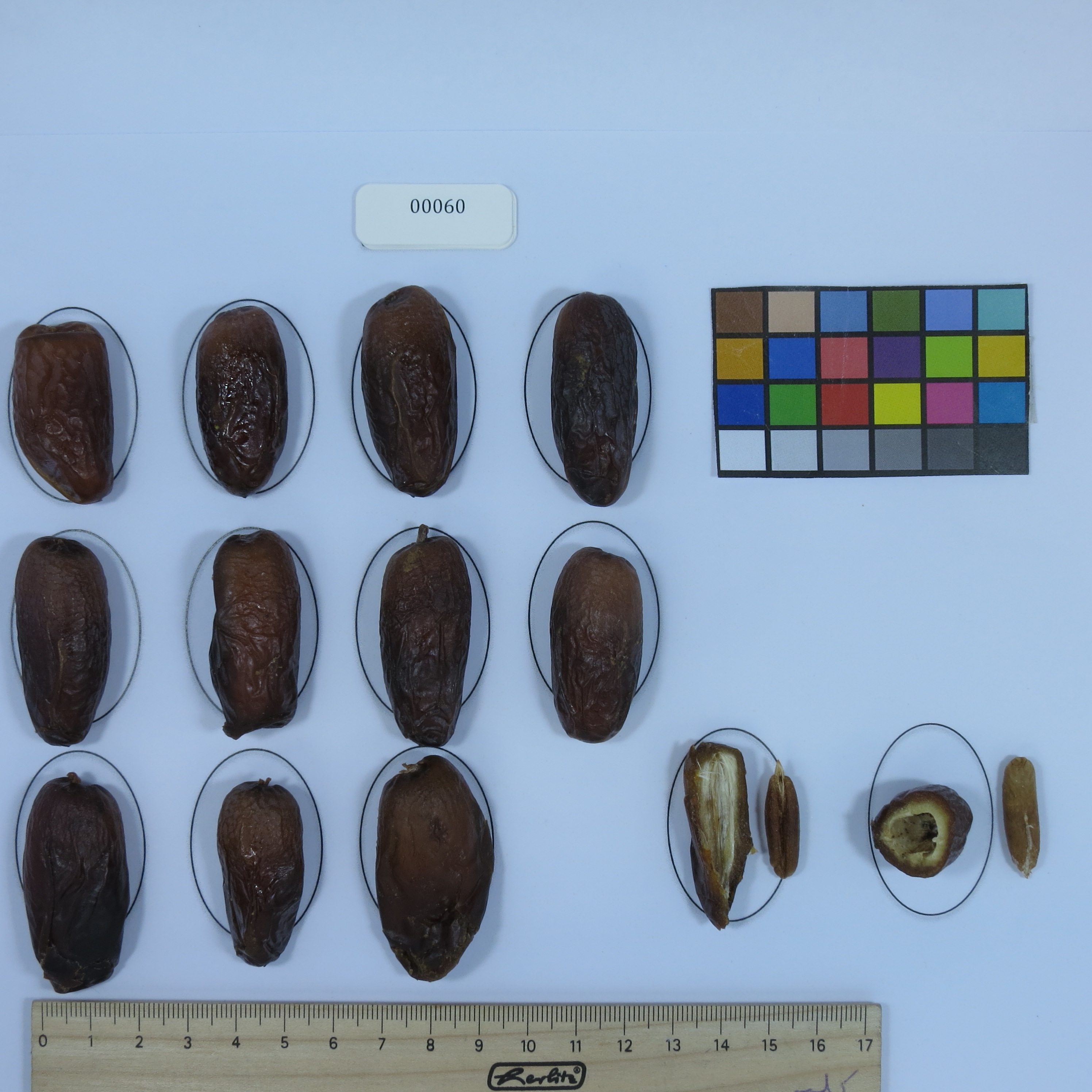

Supplement: Supplementary file 5 — Supplementary material [file mmc5.zip › dates images/00060.JPG]

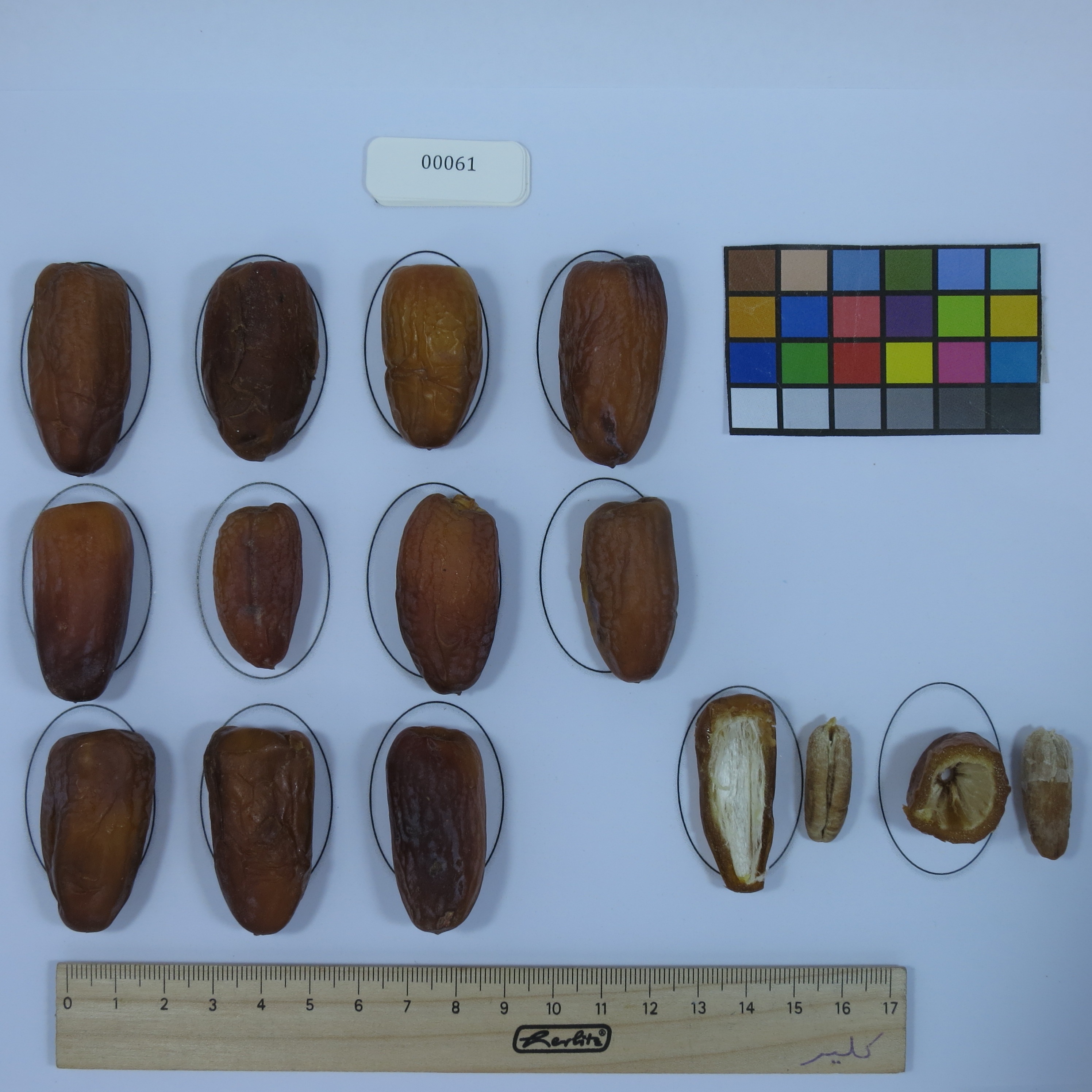

Supplement: Supplementary file 5 — Supplementary material [file mmc5.zip › dates images/00061.JPG]

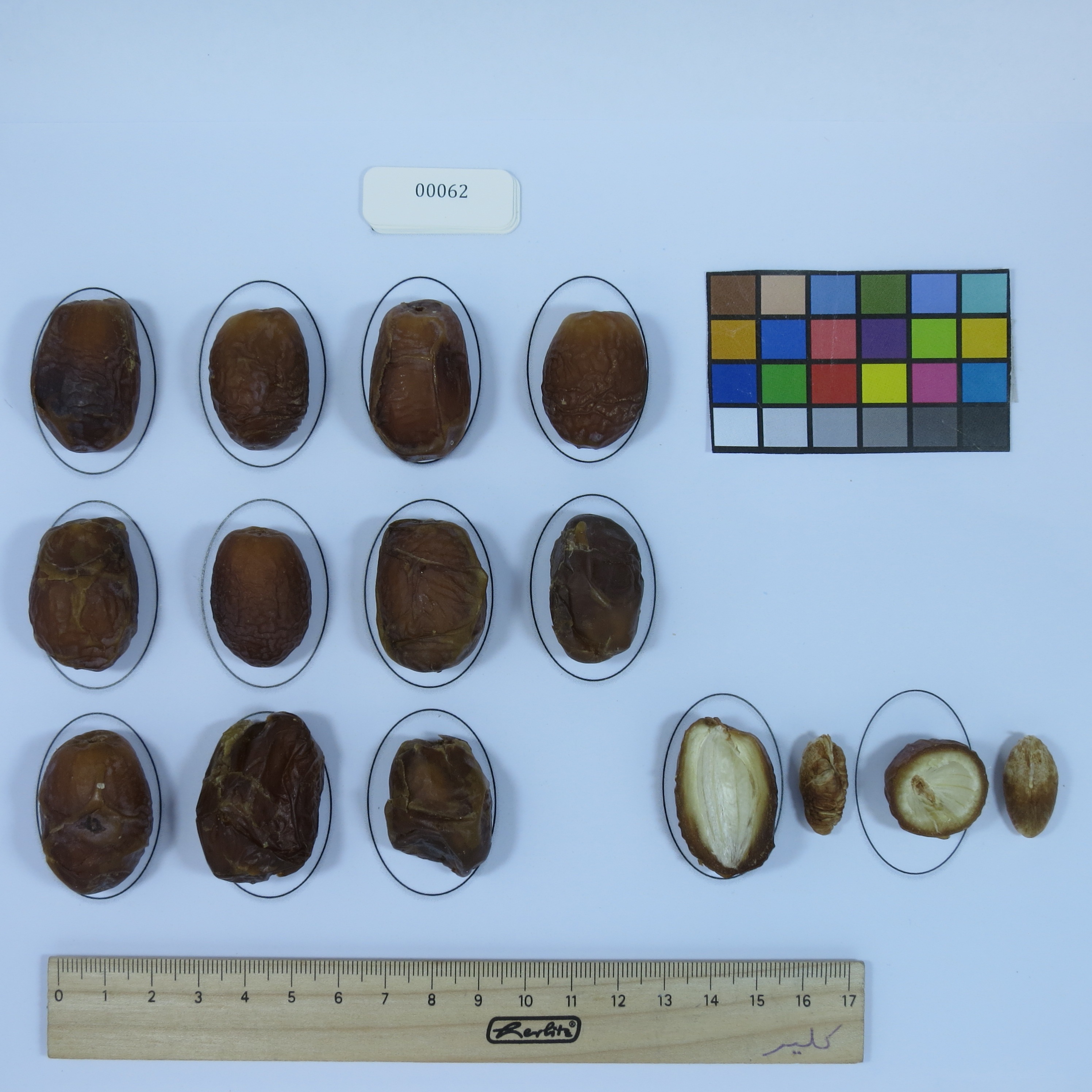

Supplement: Supplementary file 5 — Supplementary material [file mmc5.zip › dates images/00062.JPG]

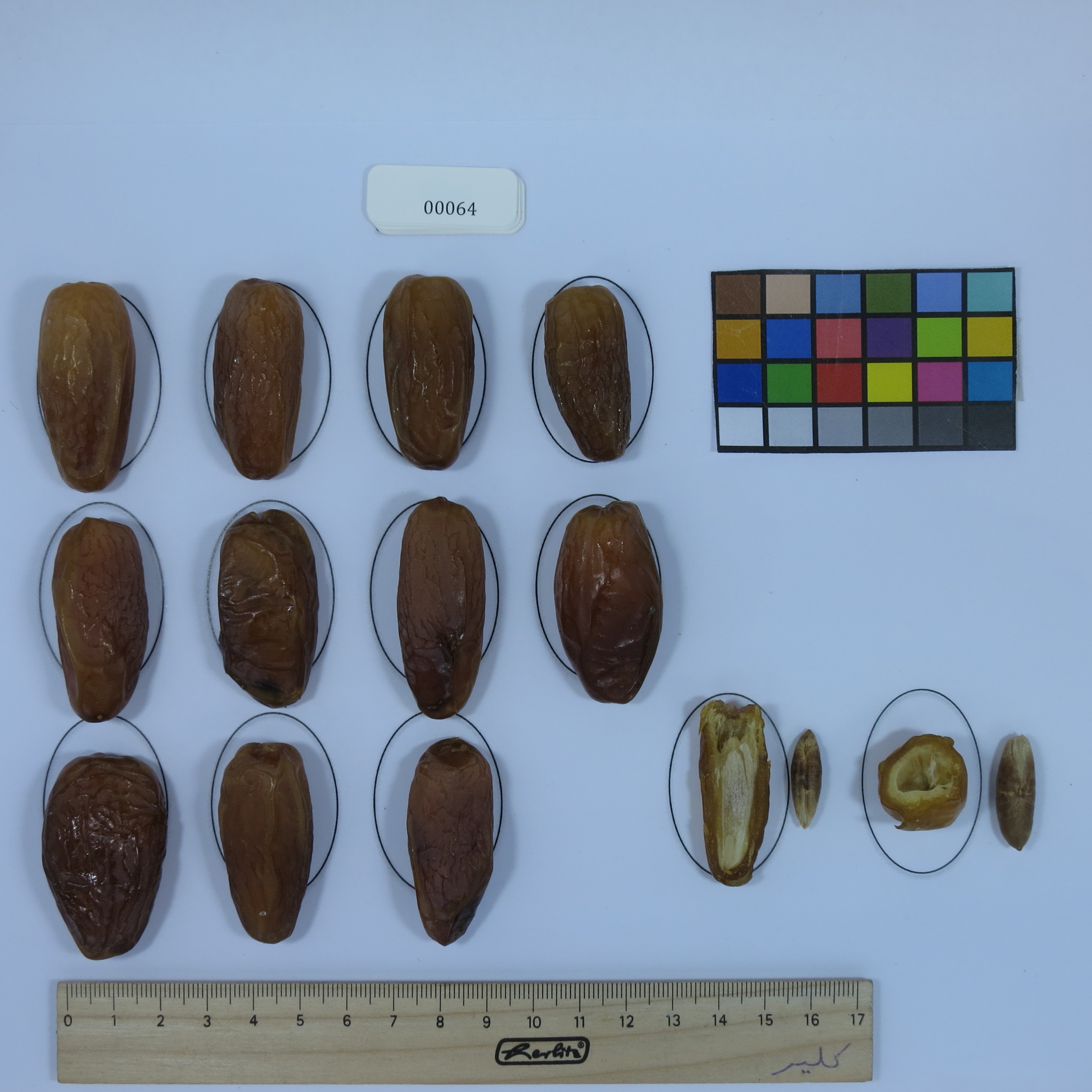

Supplement: Supplementary file 5 — Supplementary material [file mmc5.zip › dates images/00064.JPG]

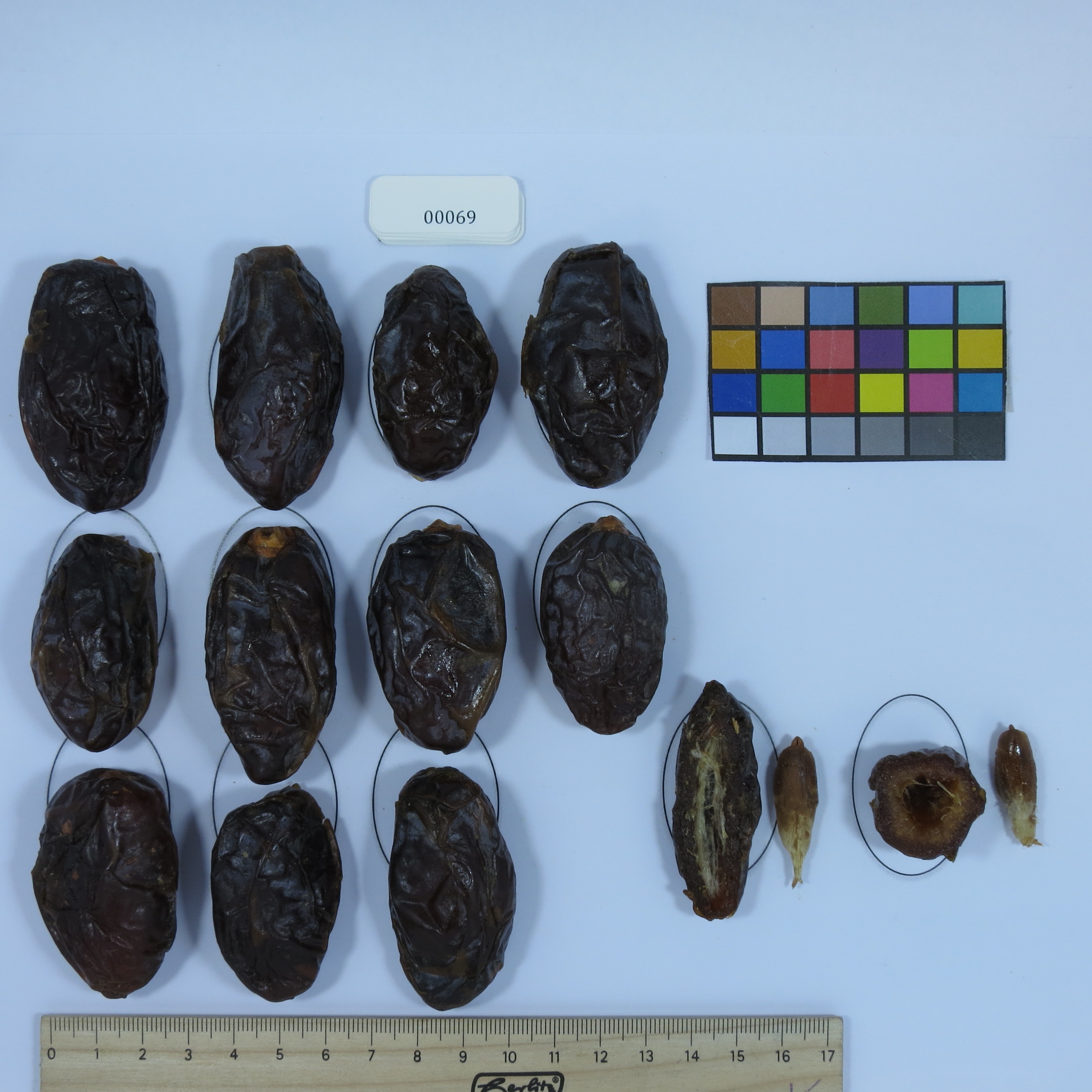

Supplement: Supplementary file 5 — Supplementary material [file mmc5.zip › dates images/00069.JPG]

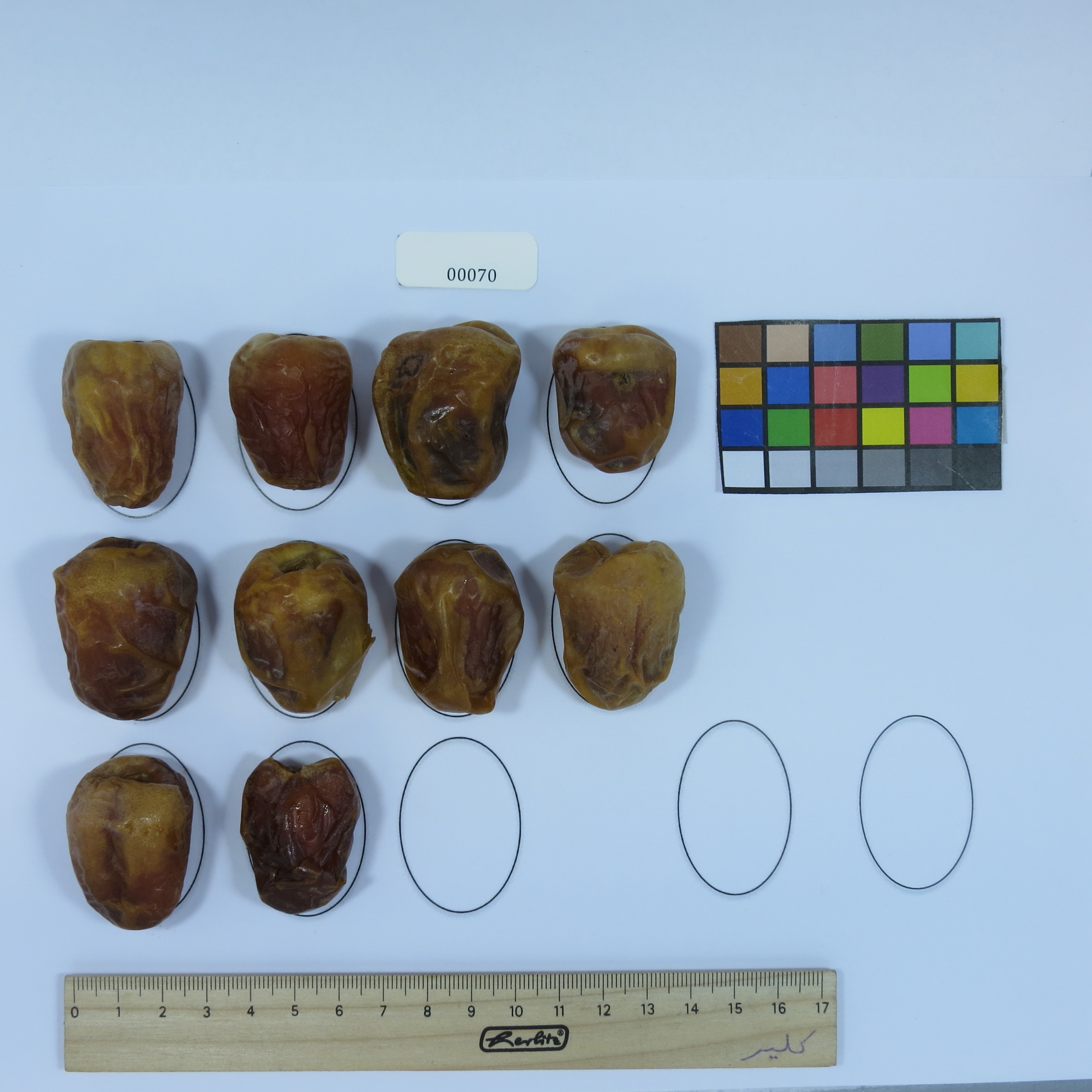

Supplement: Supplementary file 5 — Supplementary material [file mmc5.zip › dates images/00070.JPG]

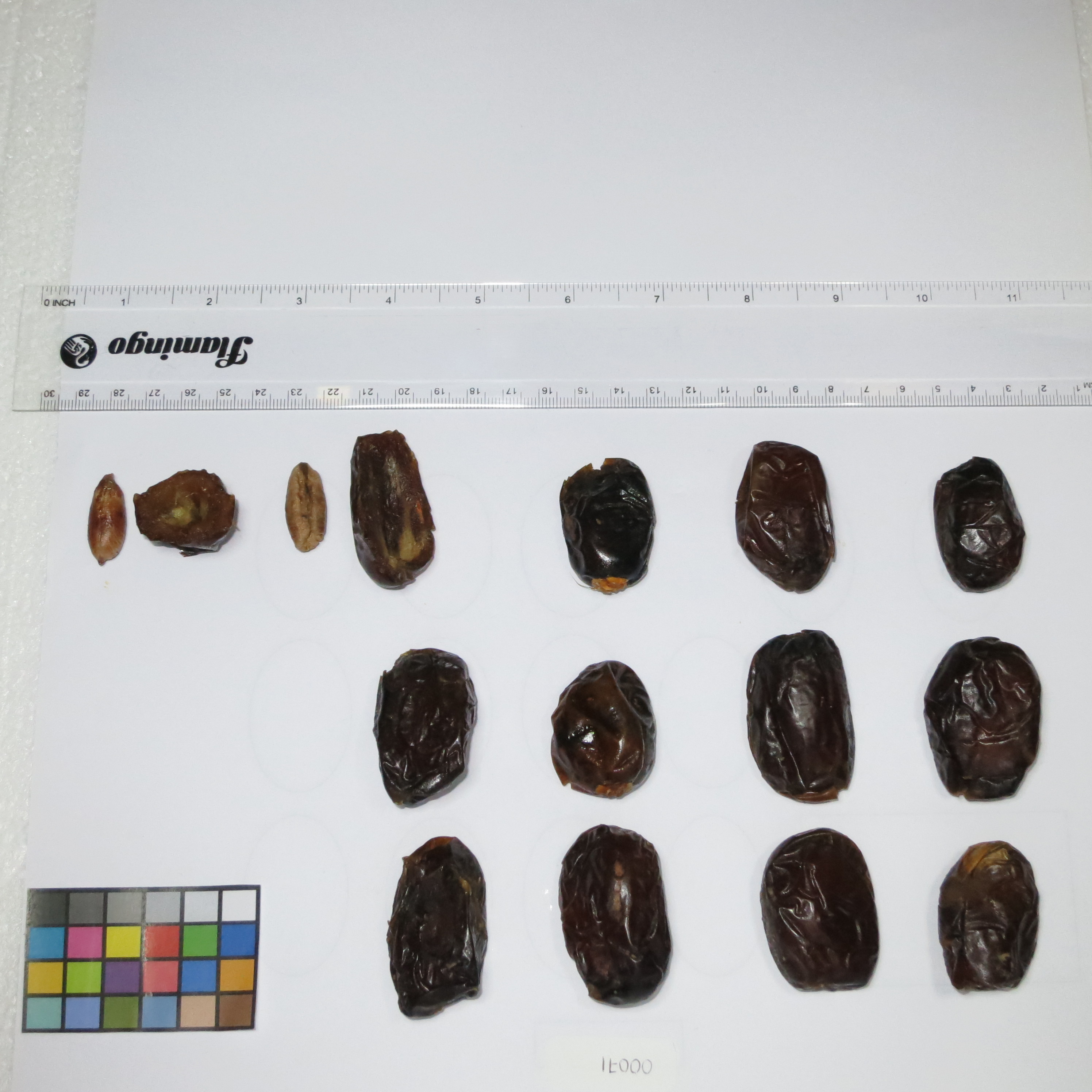

Supplement: Supplementary file 5 — Supplementary material [file mmc5.zip › dates images/00071.JPG]

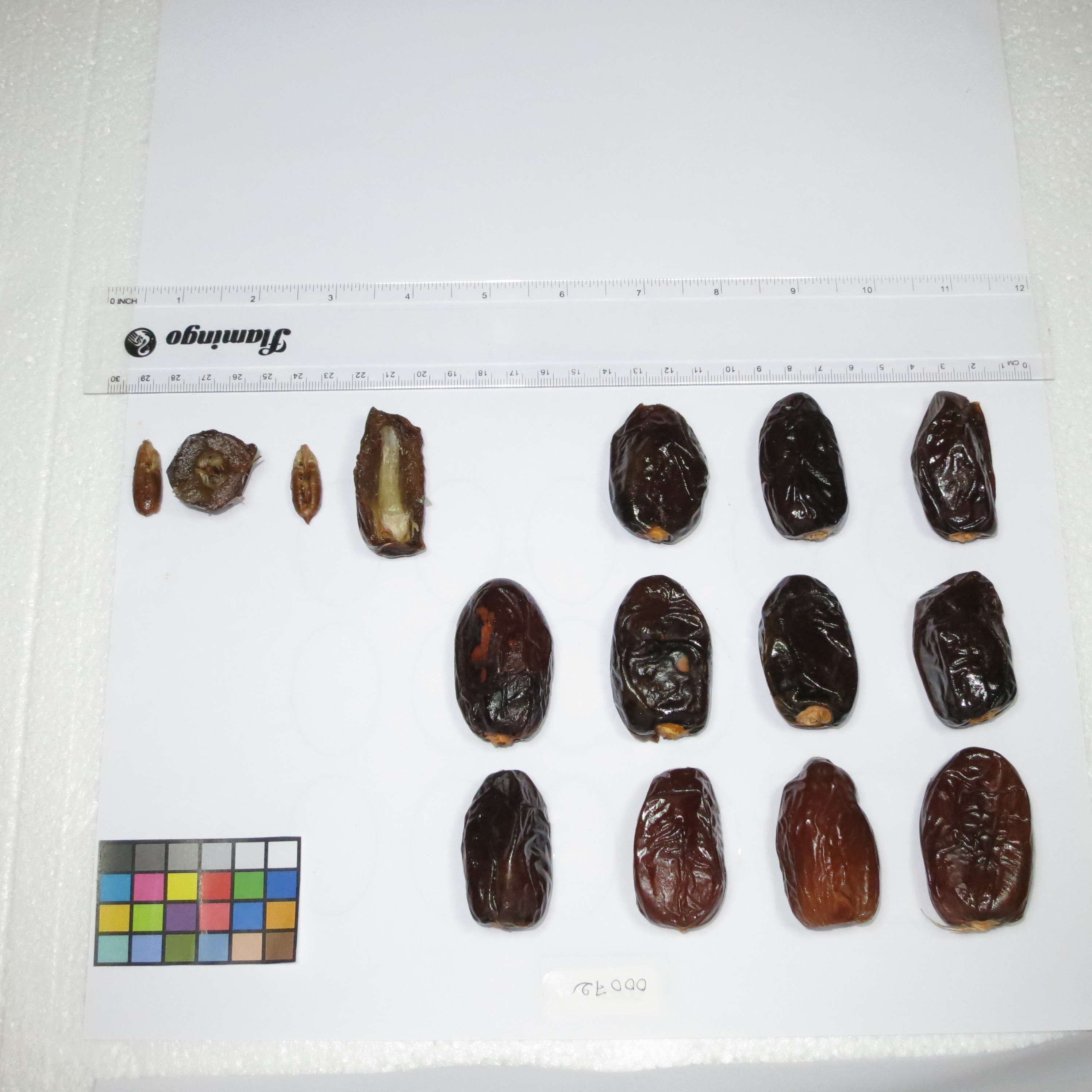

Supplement: Supplementary file 5 — Supplementary material [file mmc5.zip › dates images/00072.JPG]

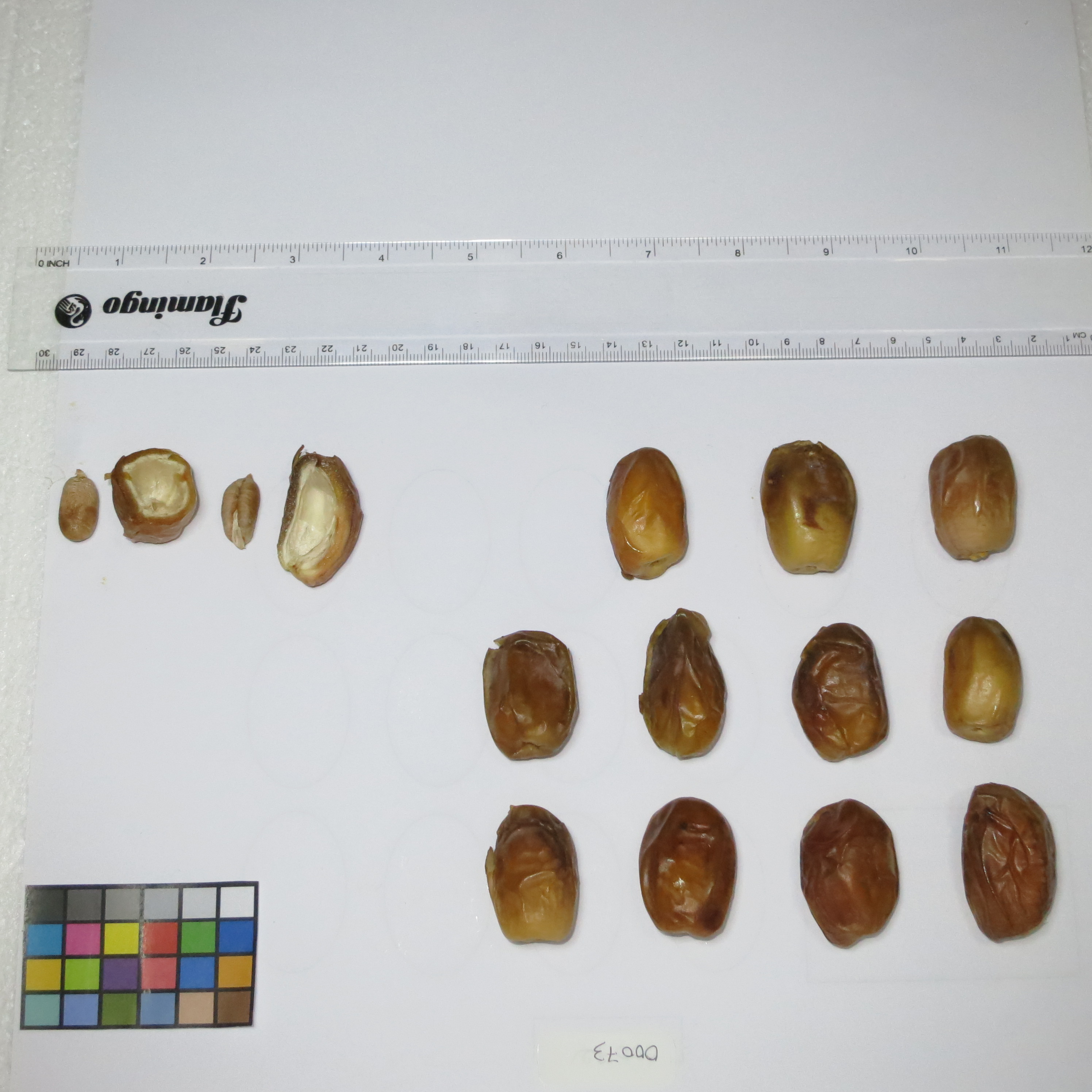

Supplement: Supplementary file 5 — Supplementary material [file mmc5.zip › dates images/00073.JPG]

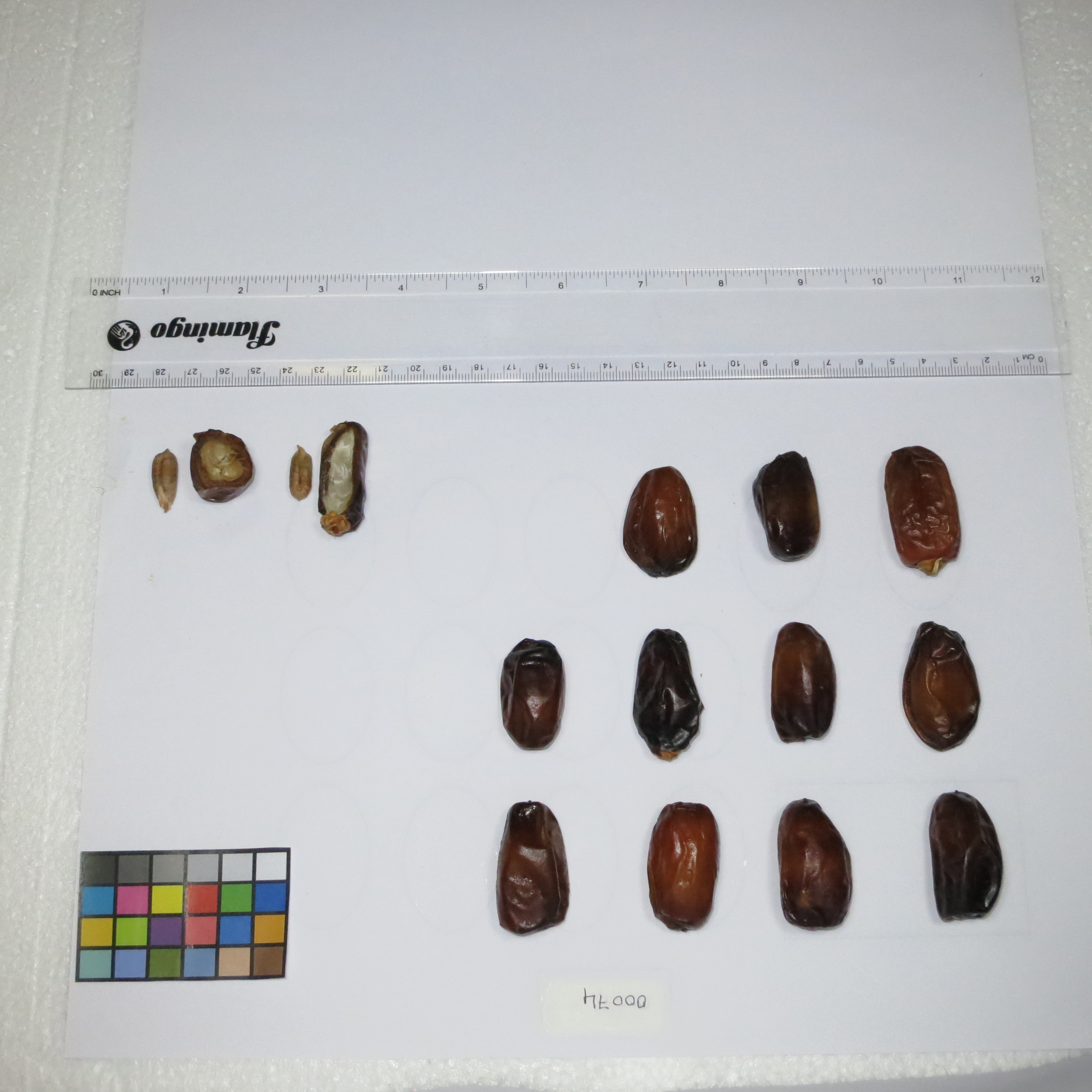

Supplement: Supplementary file 5 — Supplementary material [file mmc5.zip › dates images/00074.JPG]

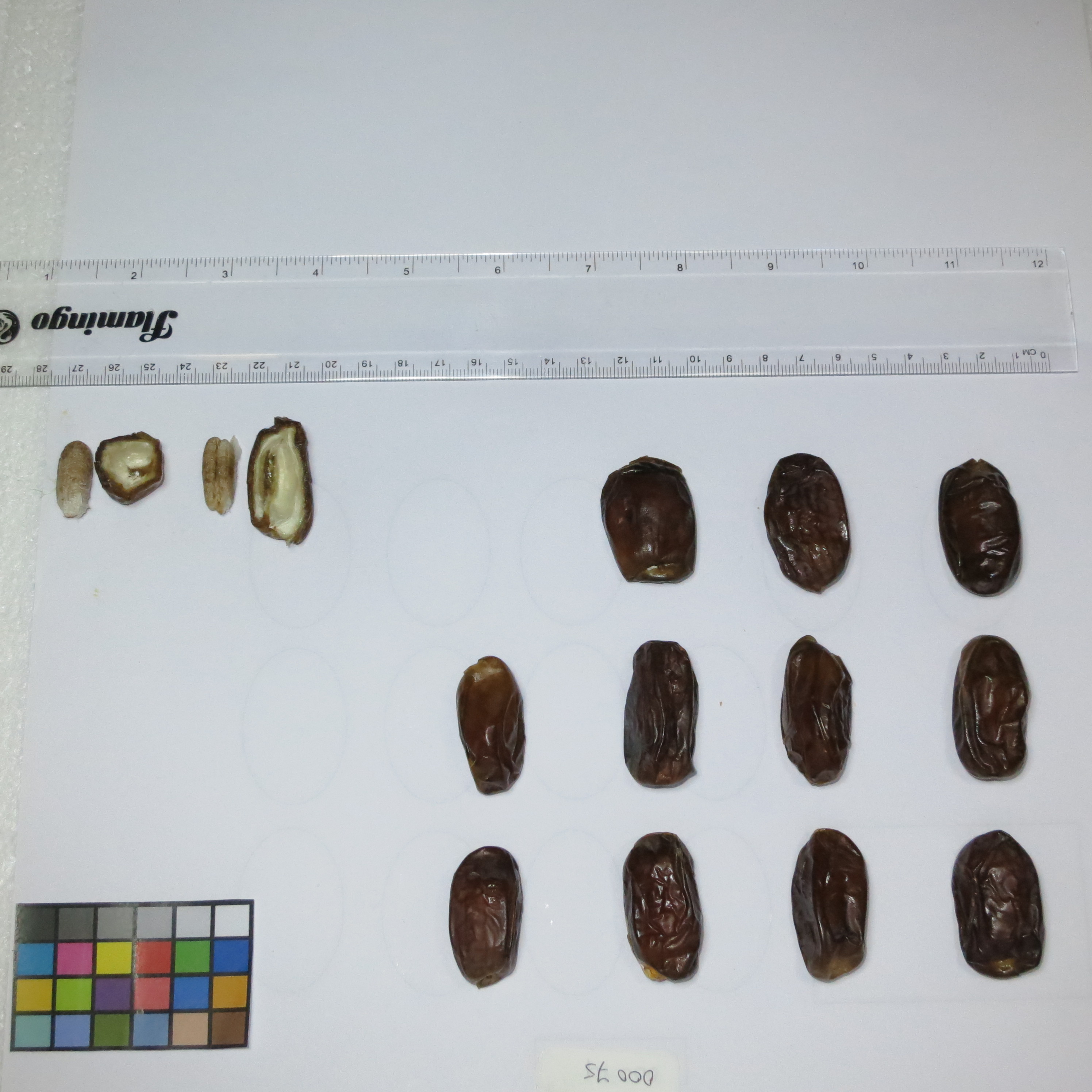

Supplement: Supplementary file 5 — Supplementary material [file mmc5.zip › dates images/00075.JPG]

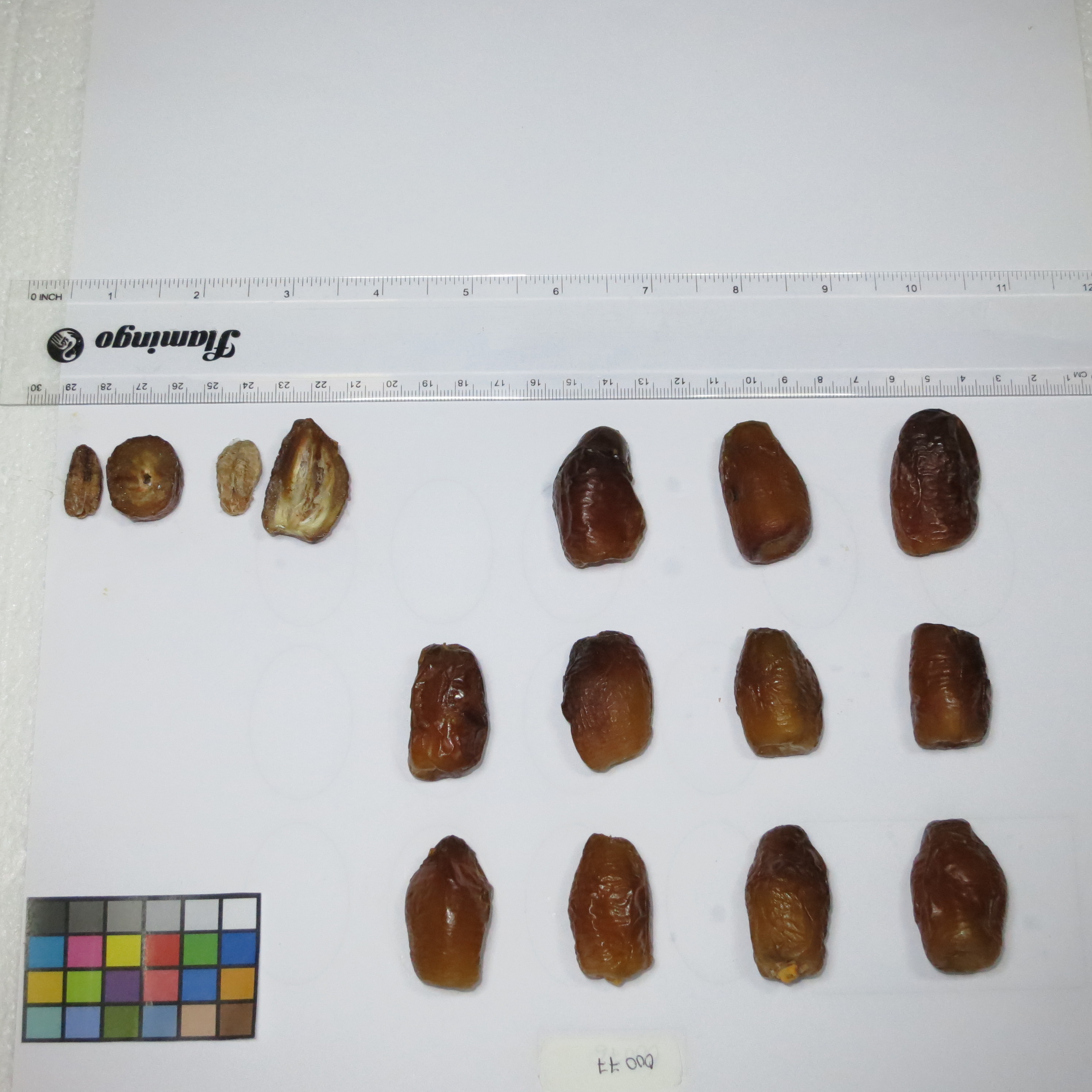

Supplement: Supplementary file 5 — Supplementary material [file mmc5.zip › dates images/00077.JPG]

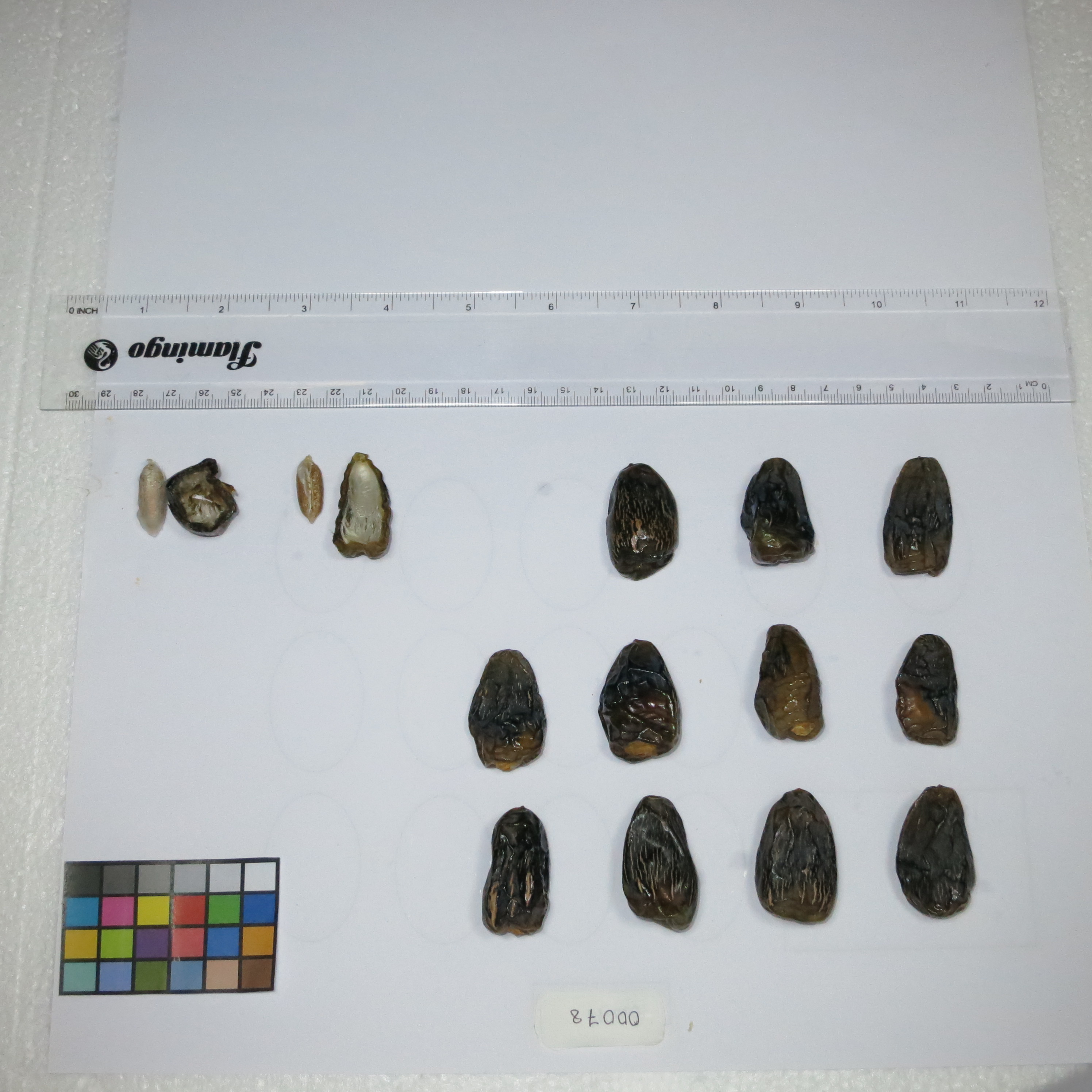

Supplement: Supplementary file 5 — Supplementary material [file mmc5.zip › dates images/00078.JPG]

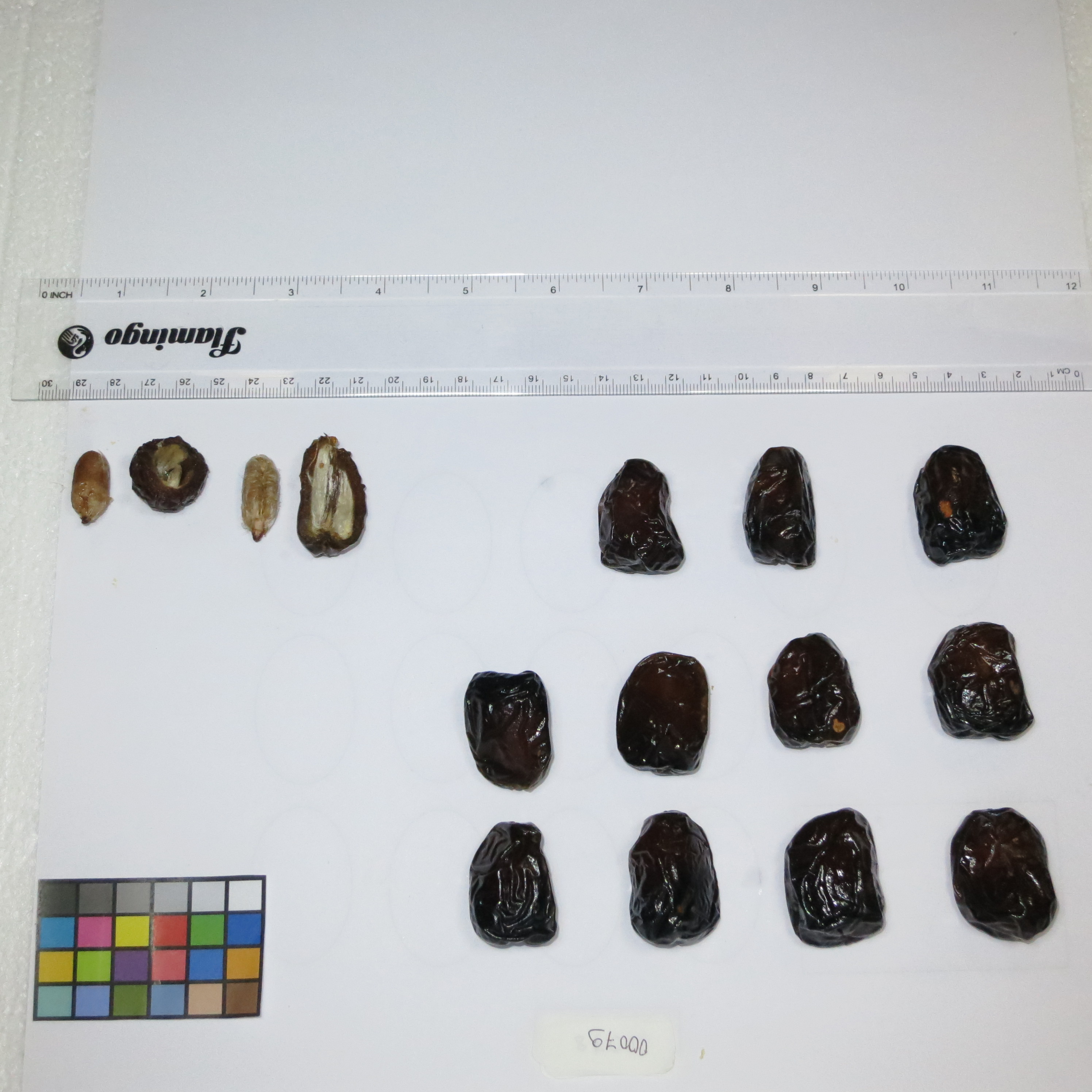

Supplement: Supplementary file 5 — Supplementary material [file mmc5.zip › dates images/00079.JPG]

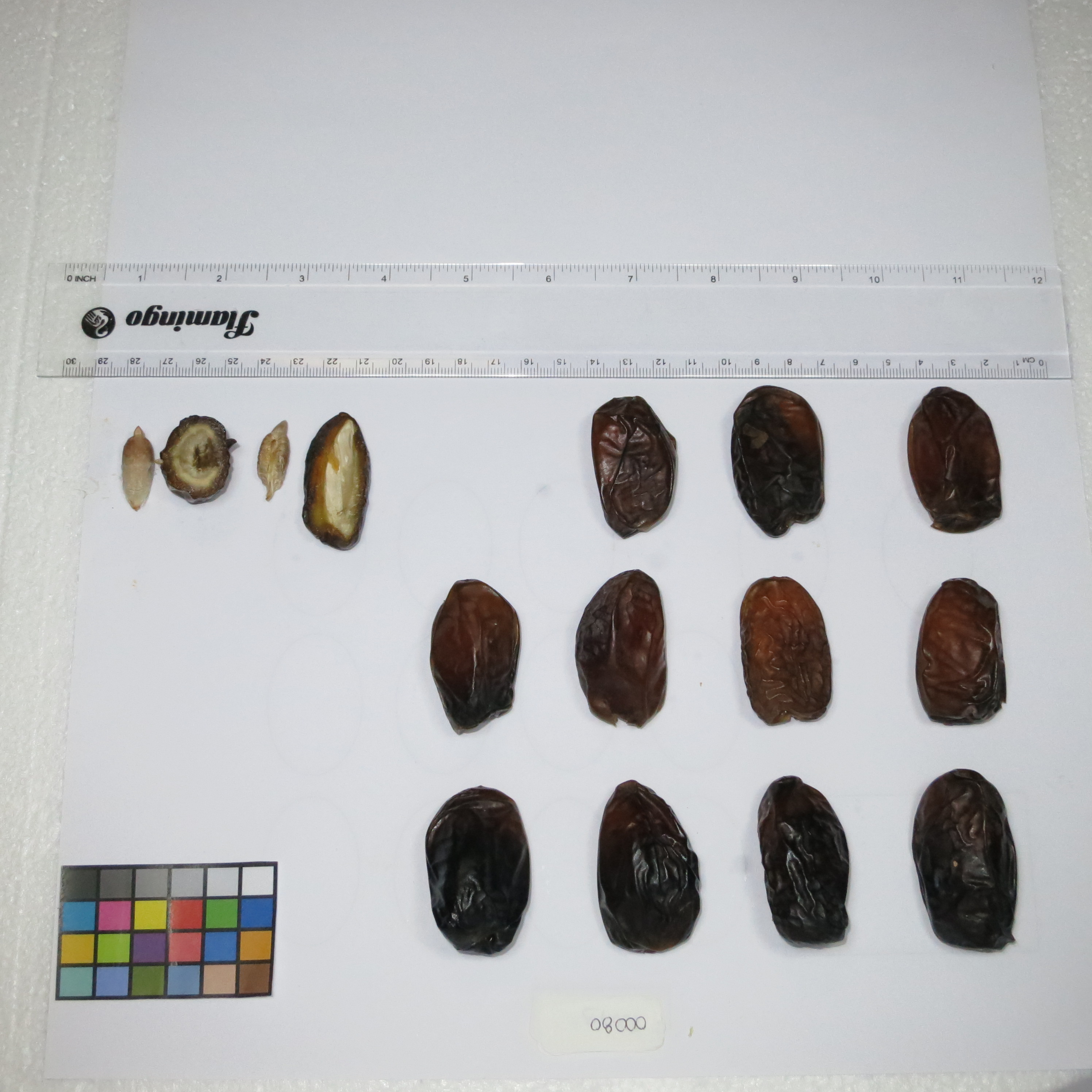

Supplement: Supplementary file 5 — Supplementary material [file mmc5.zip › dates images/00080.JPG]

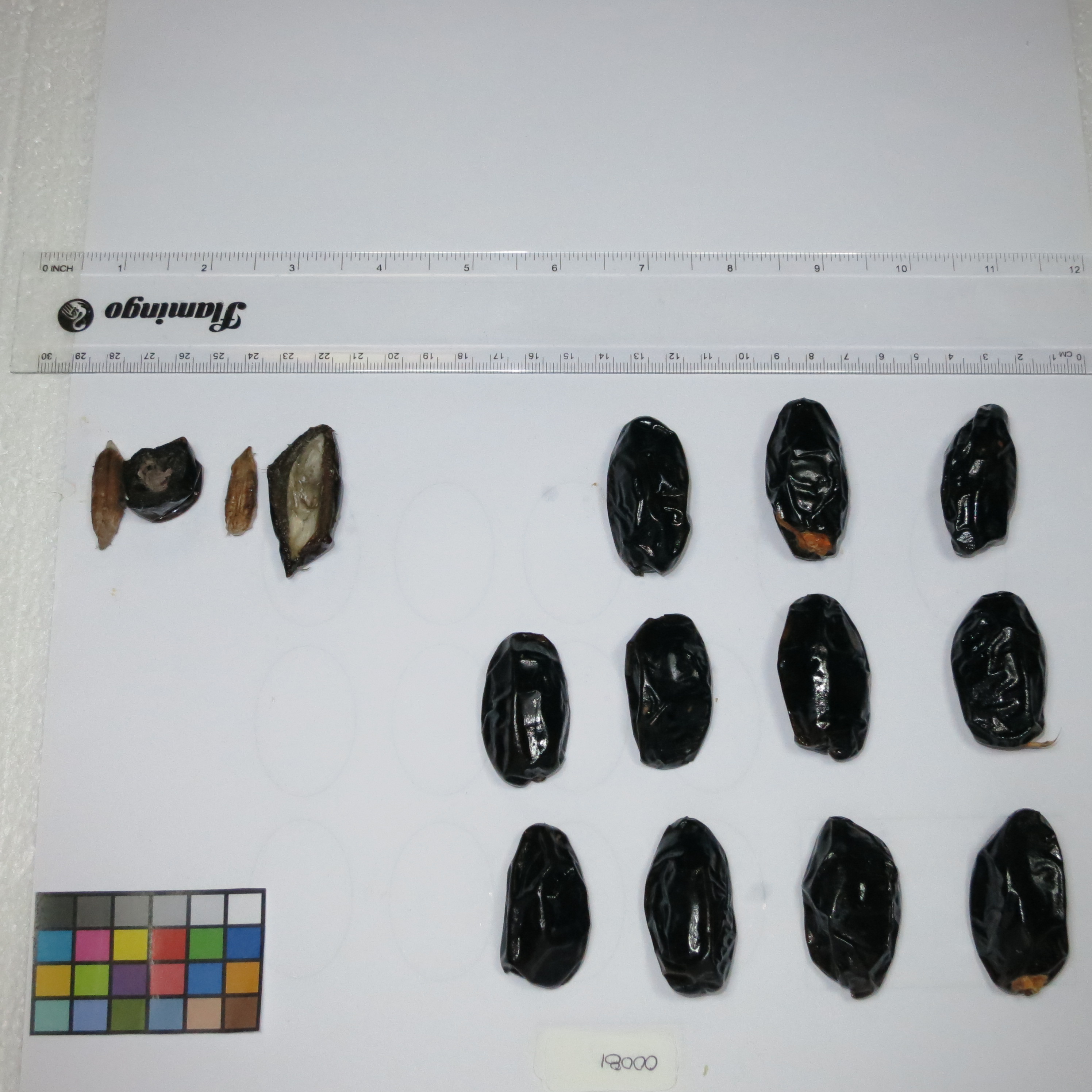

Supplement: Supplementary file 5 — Supplementary material [file mmc5.zip › dates images/00081.JPG]

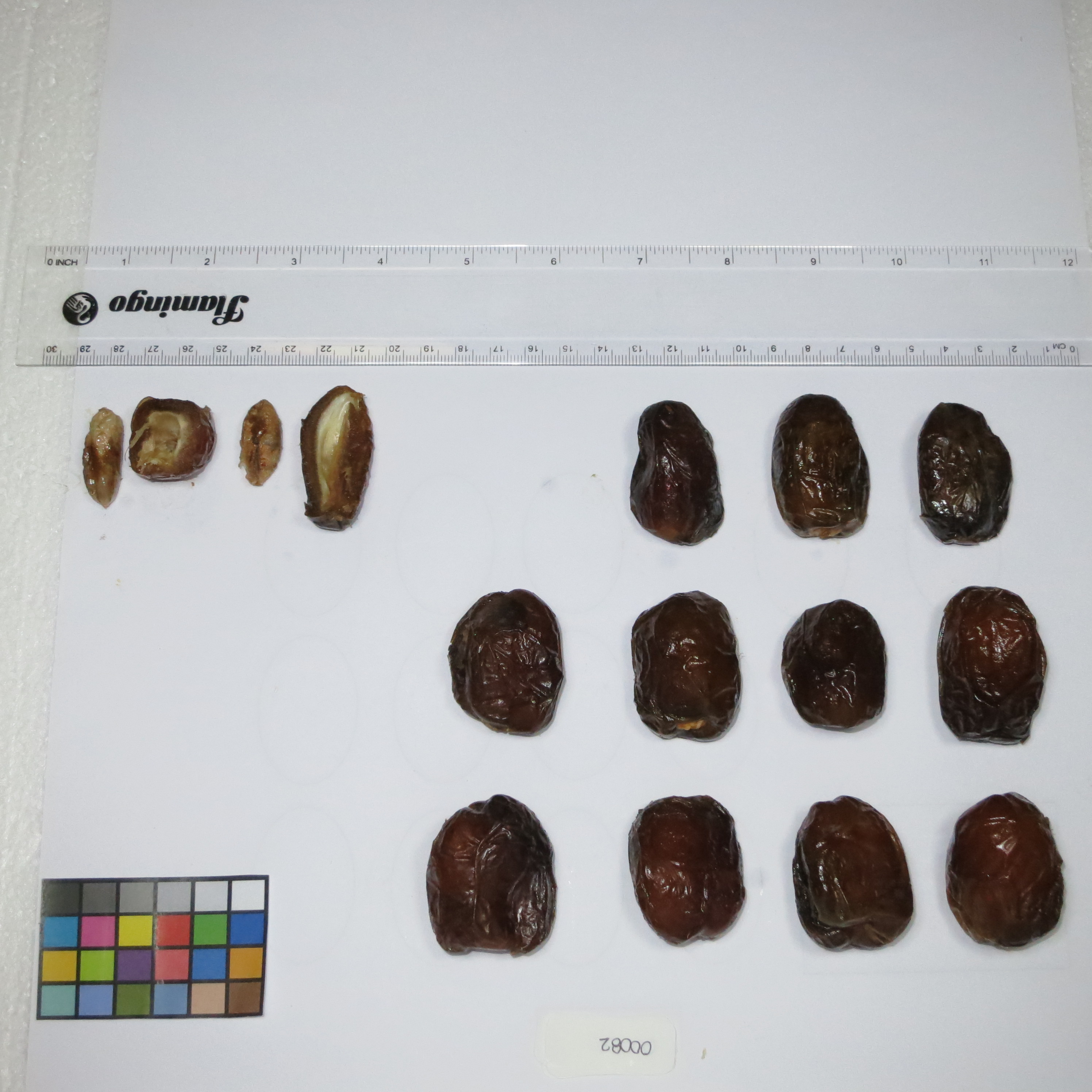

Supplement: Supplementary file 5 — Supplementary material [file mmc5.zip › dates images/00082.JPG]

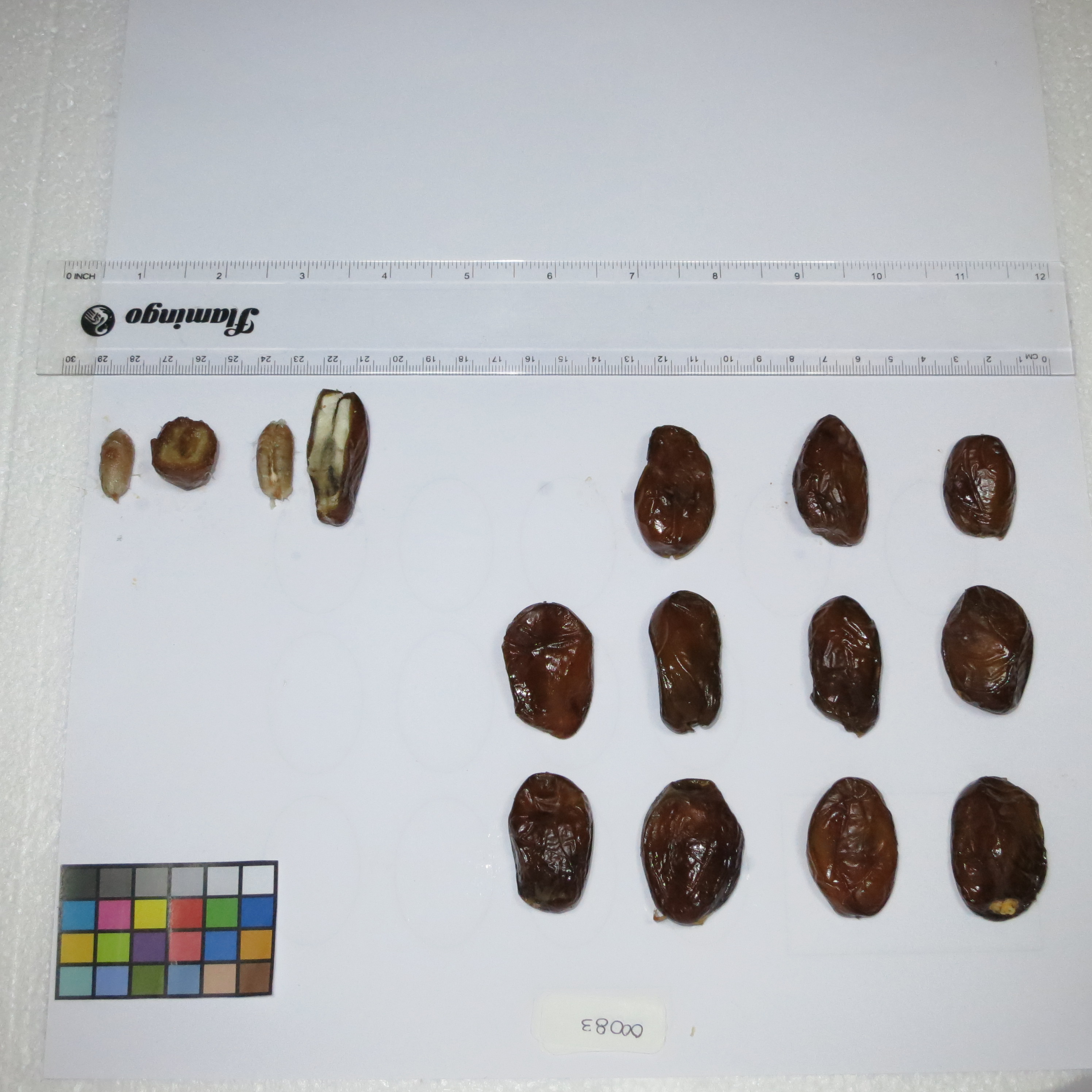

Supplement: Supplementary file 5 — Supplementary material [file mmc5.zip › dates images/00083.JPG]

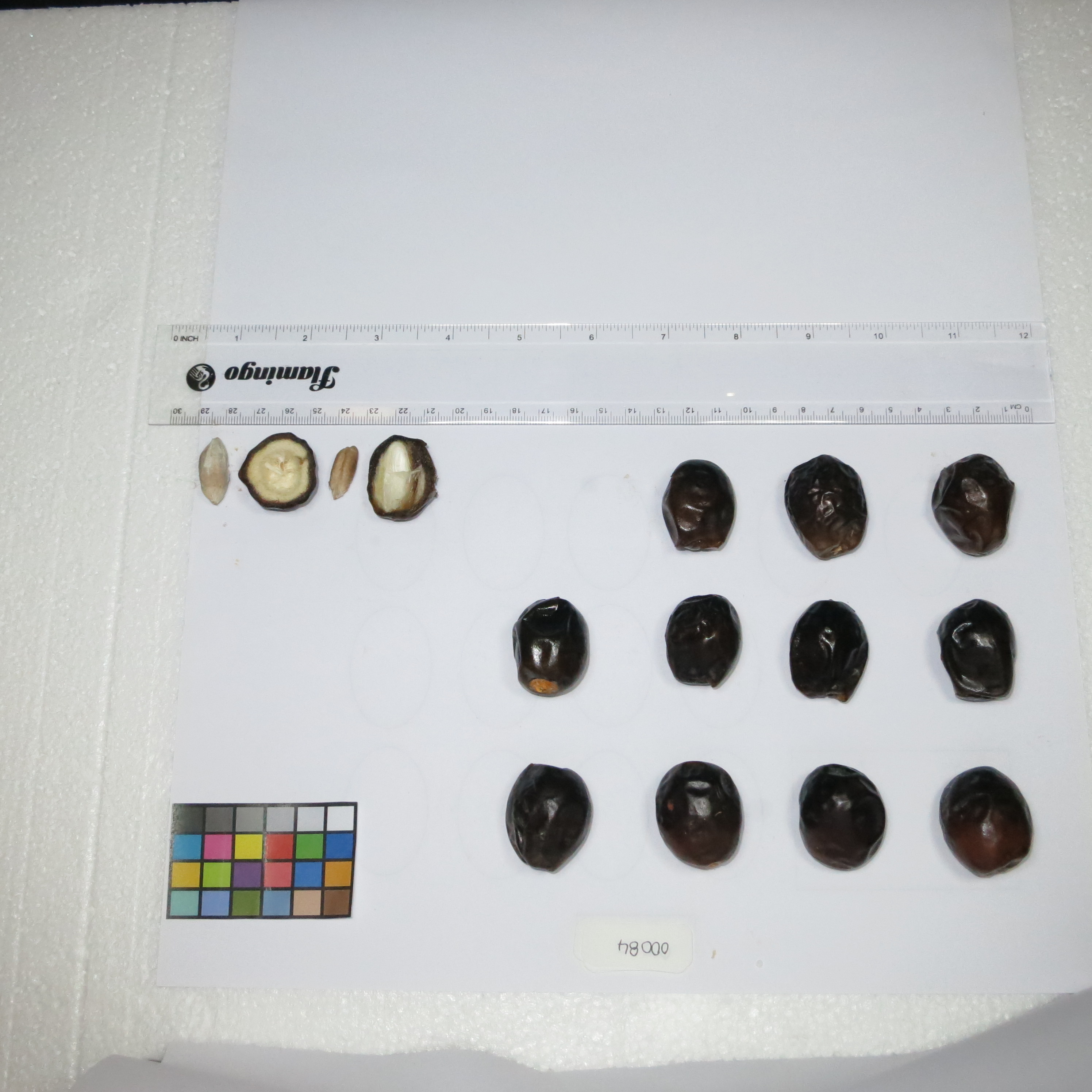

Supplement: Supplementary file 5 — Supplementary material [file mmc5.zip › dates images/00084.JPG]

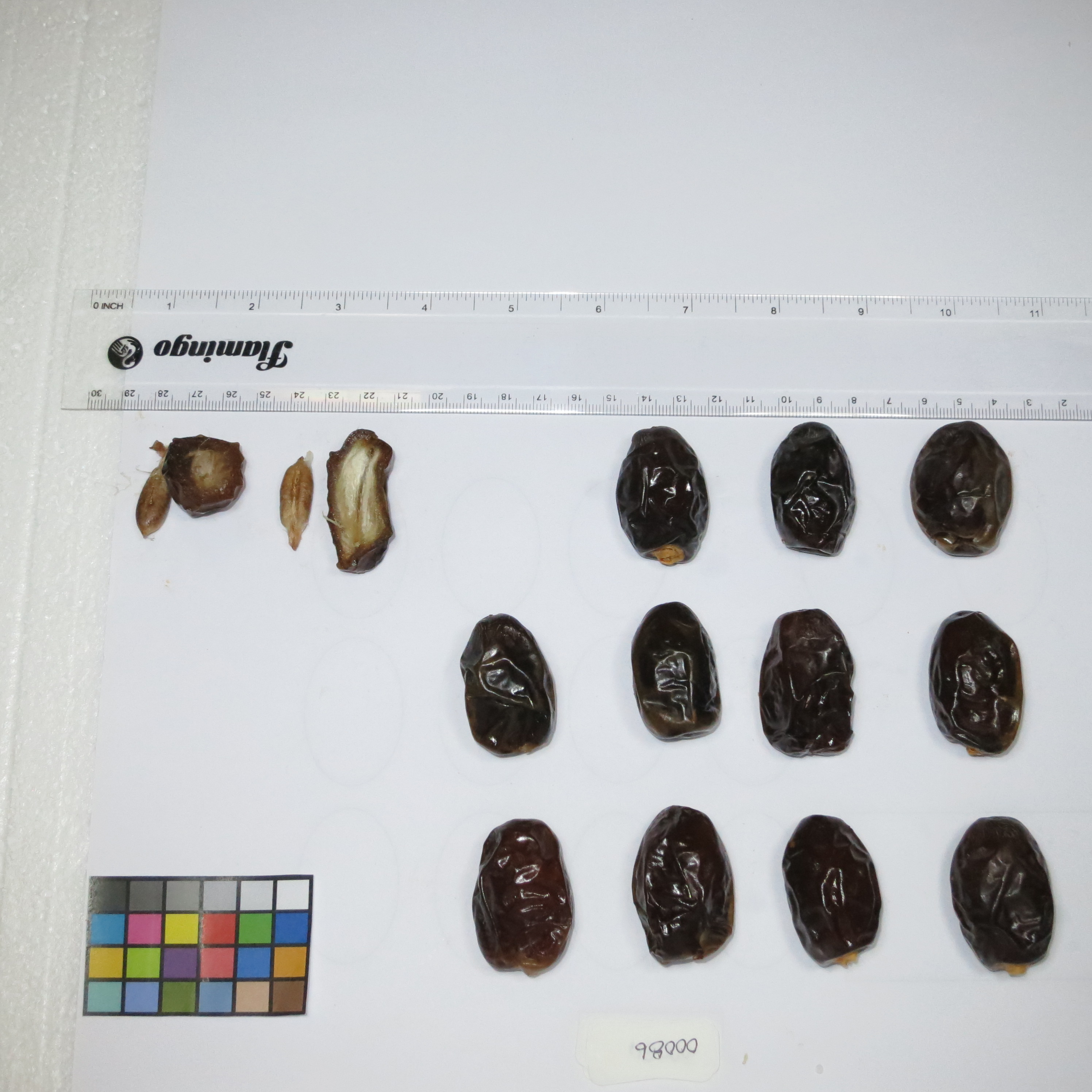

Supplement: Supplementary file 5 — Supplementary material [file mmc5.zip › dates images/00085.JPG]

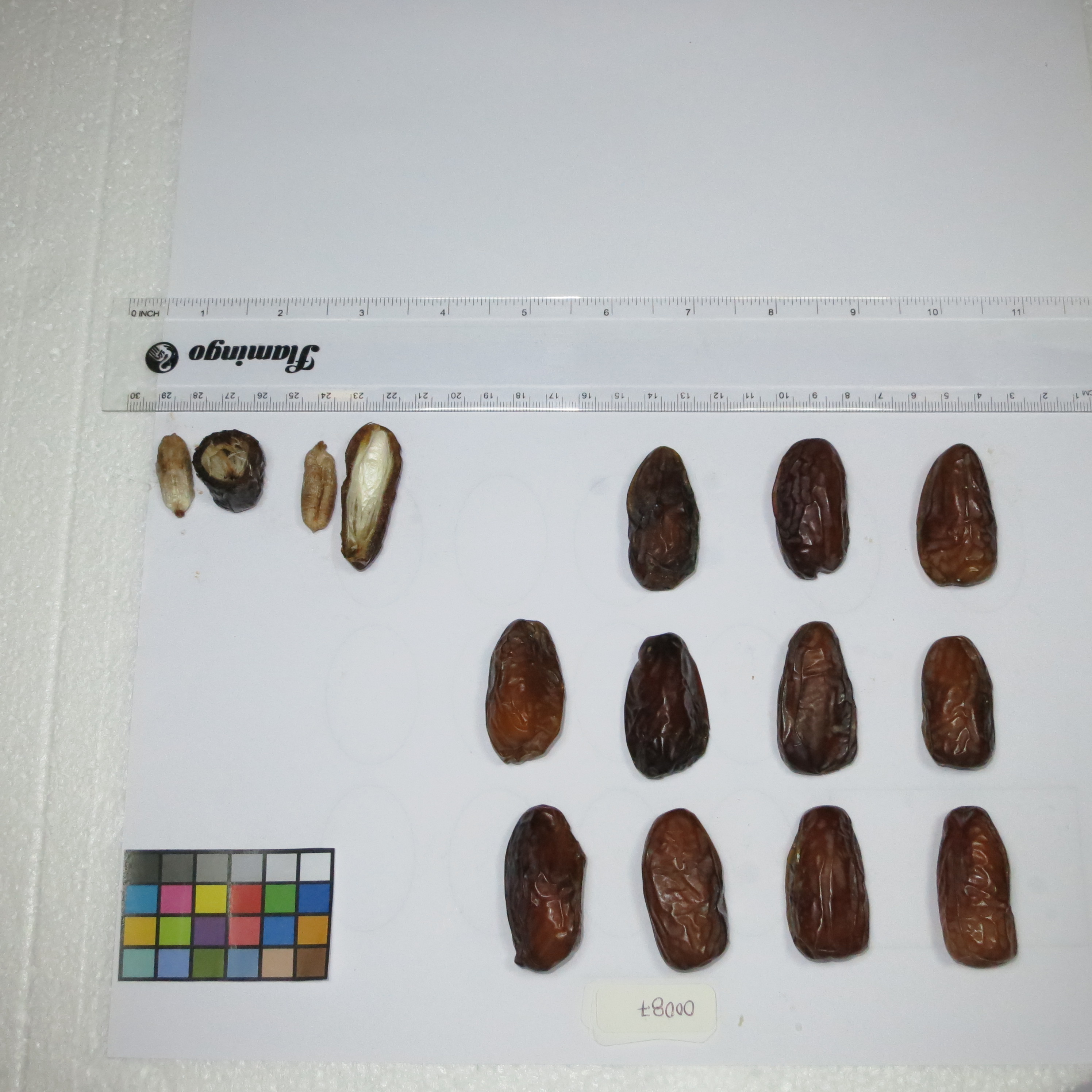

Supplement: Supplementary file 5 — Supplementary material [file mmc5.zip › dates images/00086.JPG]

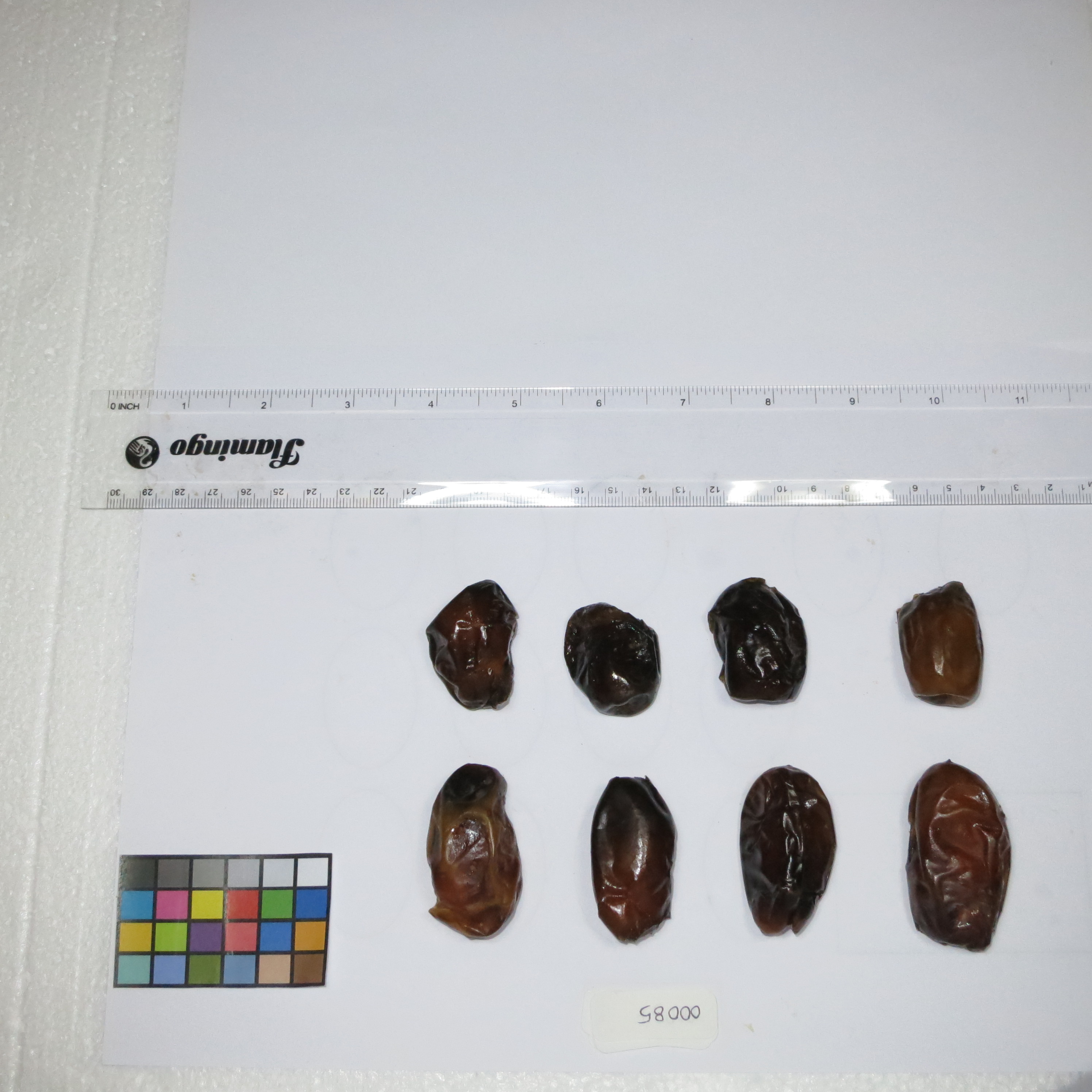

Supplement: Supplementary file 5 — Supplementary material [file mmc5.zip › dates images/00087.JPG]

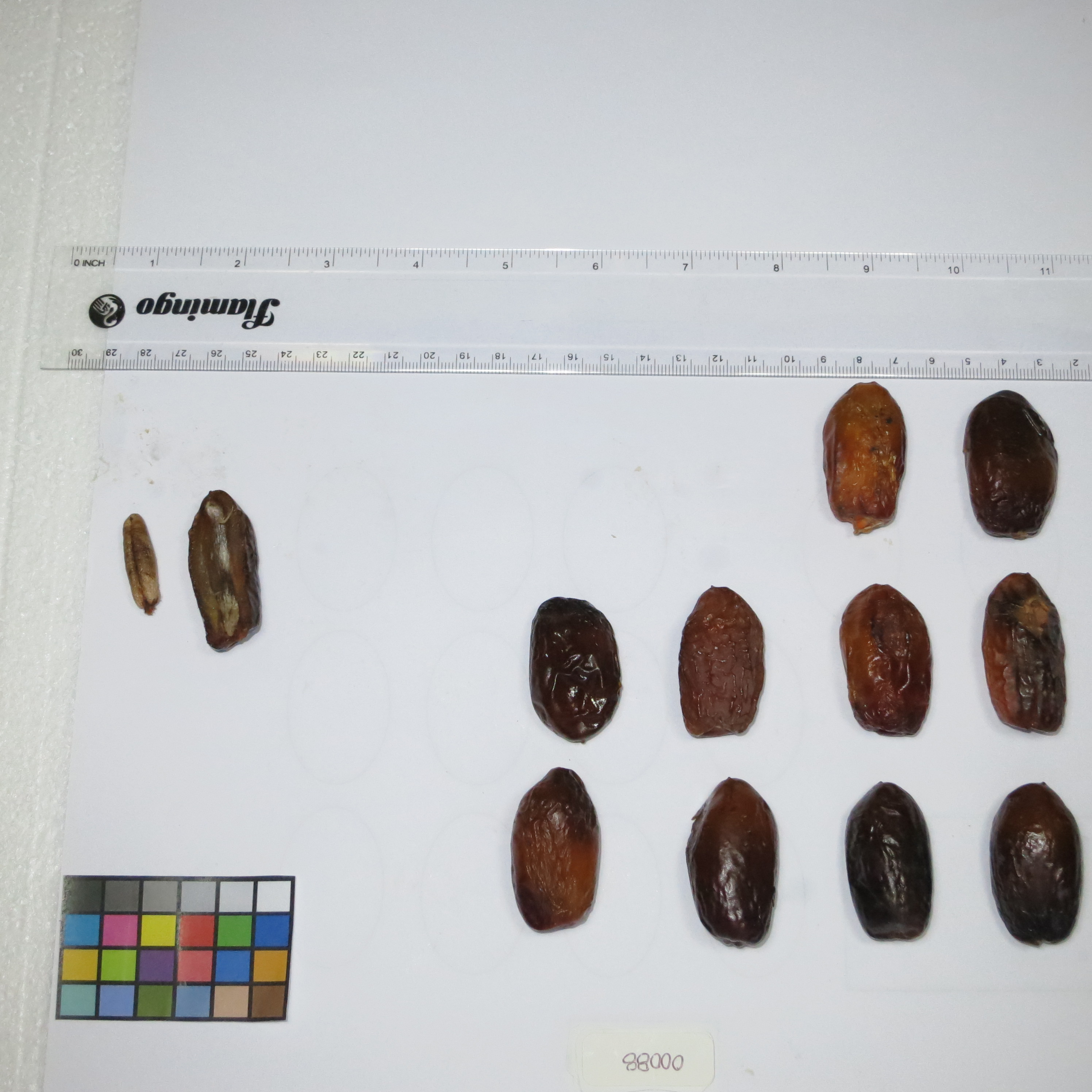

Supplement: Supplementary file 5 — Supplementary material [file mmc5.zip › dates images/00088.JPG]

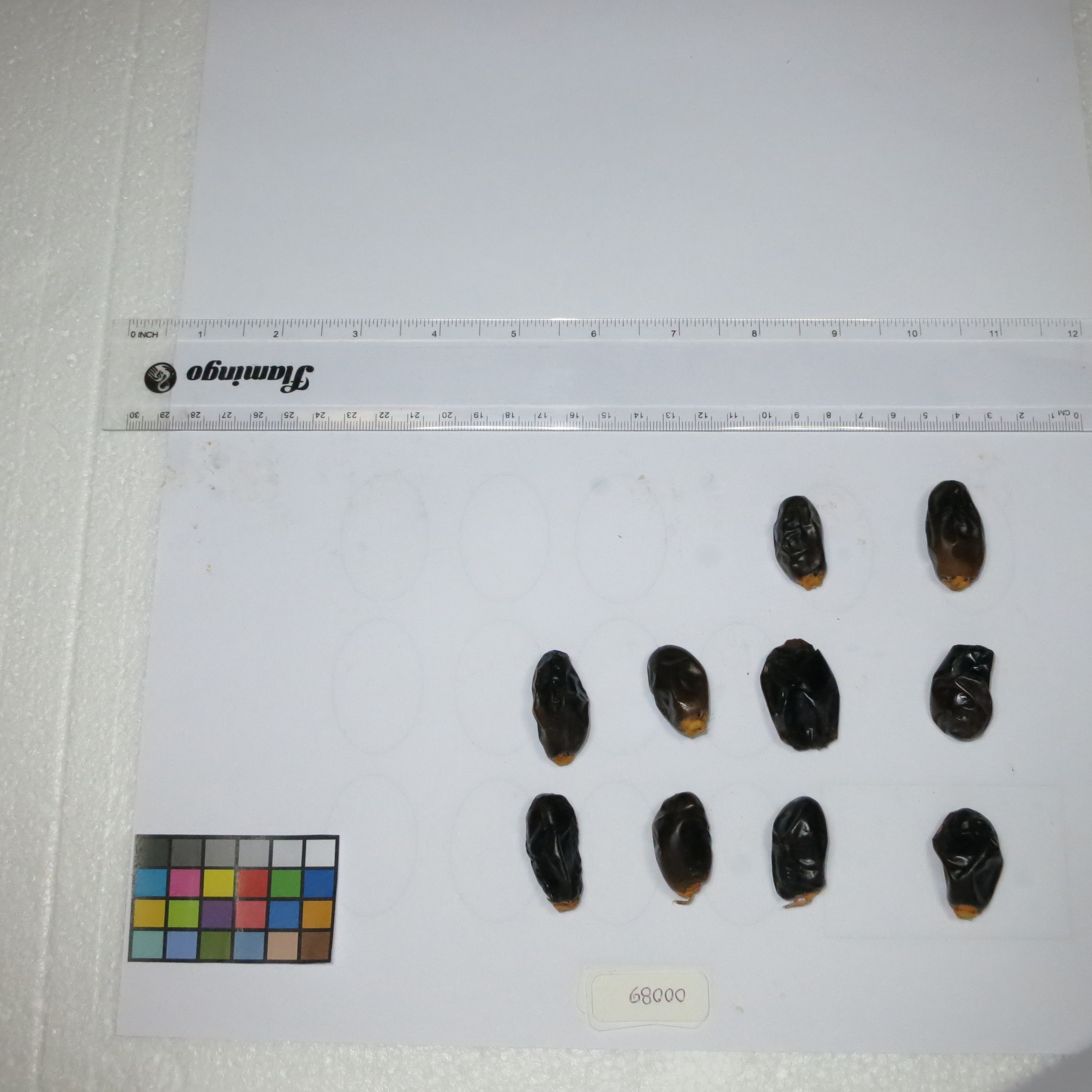

Supplement: Supplementary file 5 — Supplementary material [file mmc5.zip › dates images/00089.JPG]

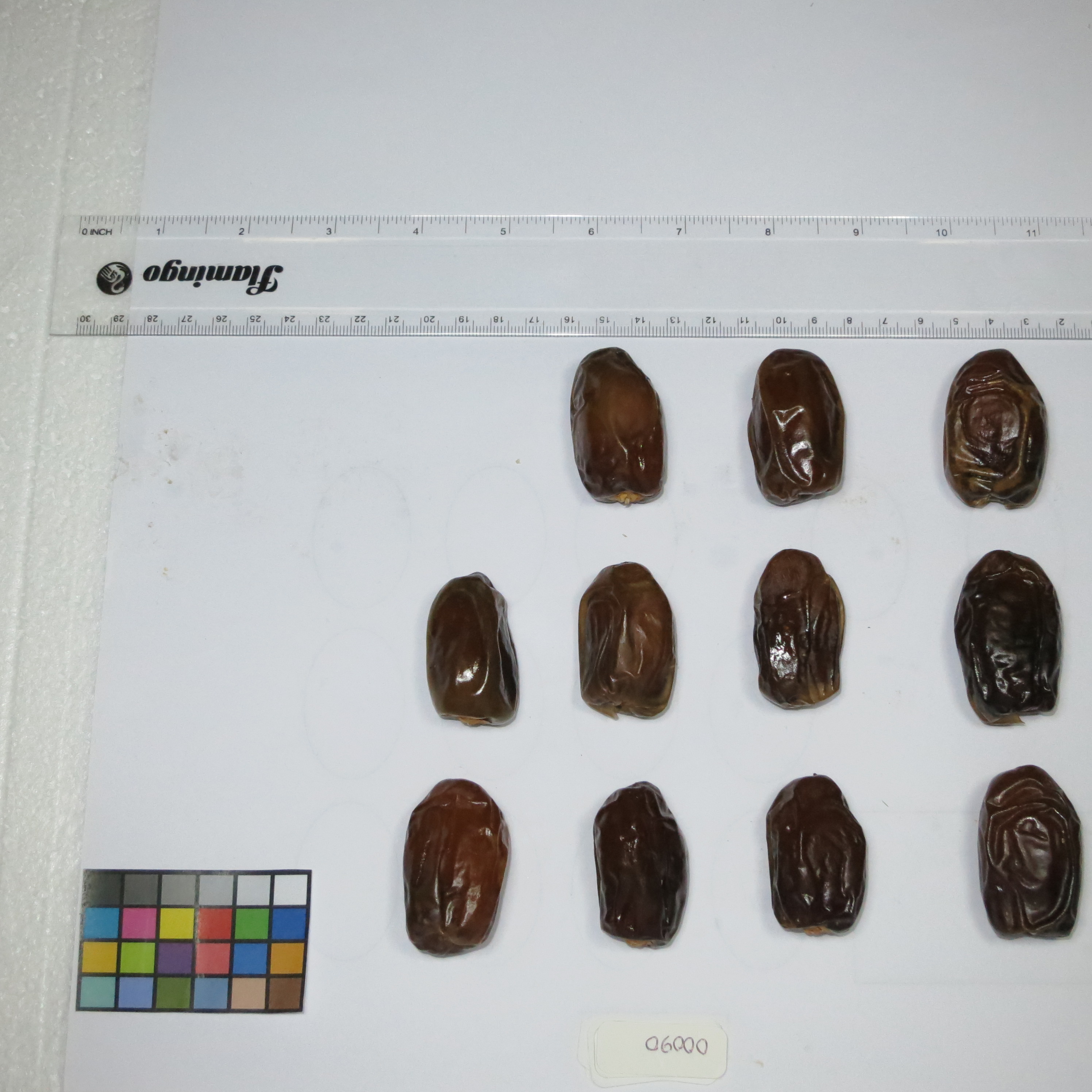

Supplement: Supplementary file 5 — Supplementary material [file mmc5.zip › dates images/00090.JPG]

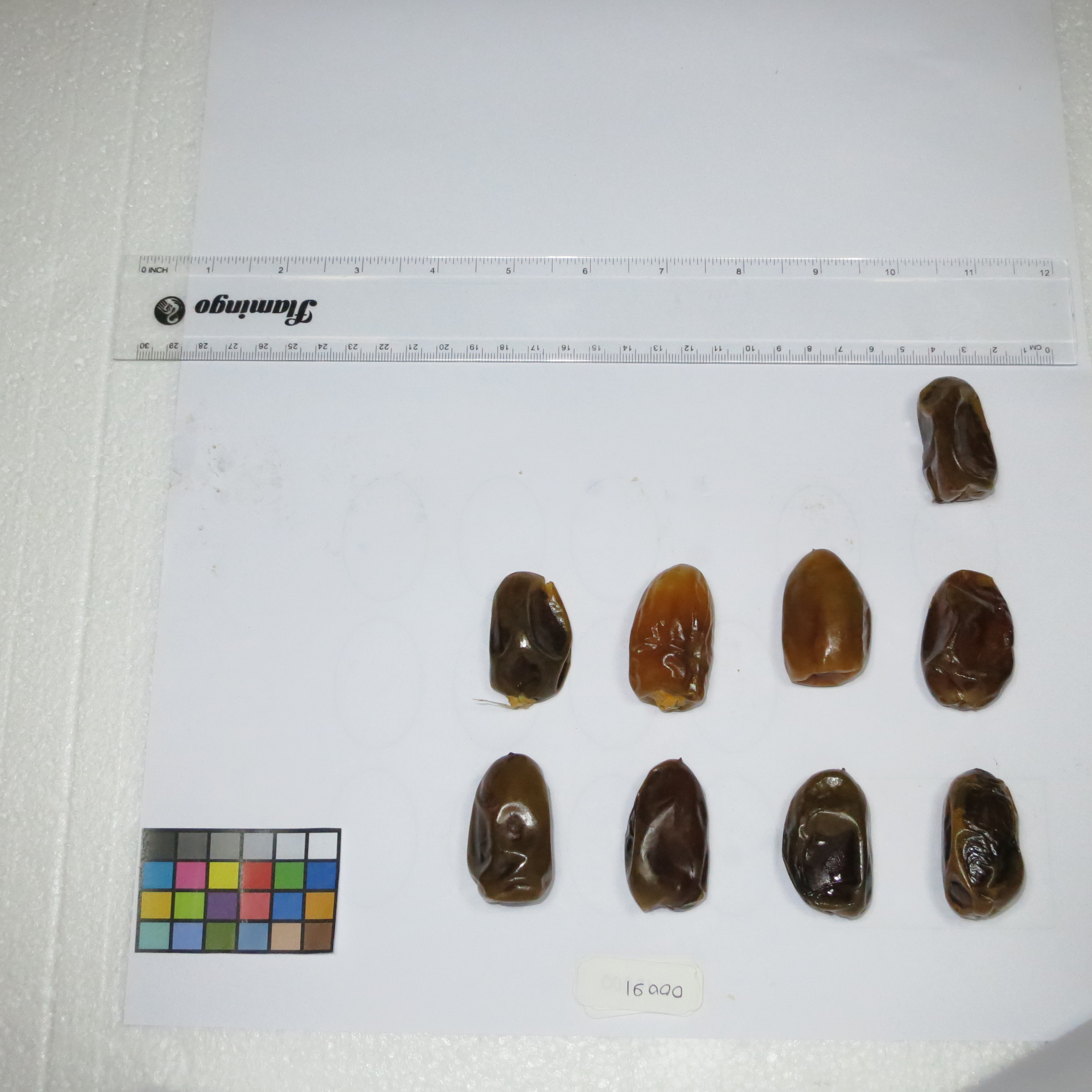

Supplement: Supplementary file 5 — Supplementary material [file mmc5.zip › dates images/00091.JPG]

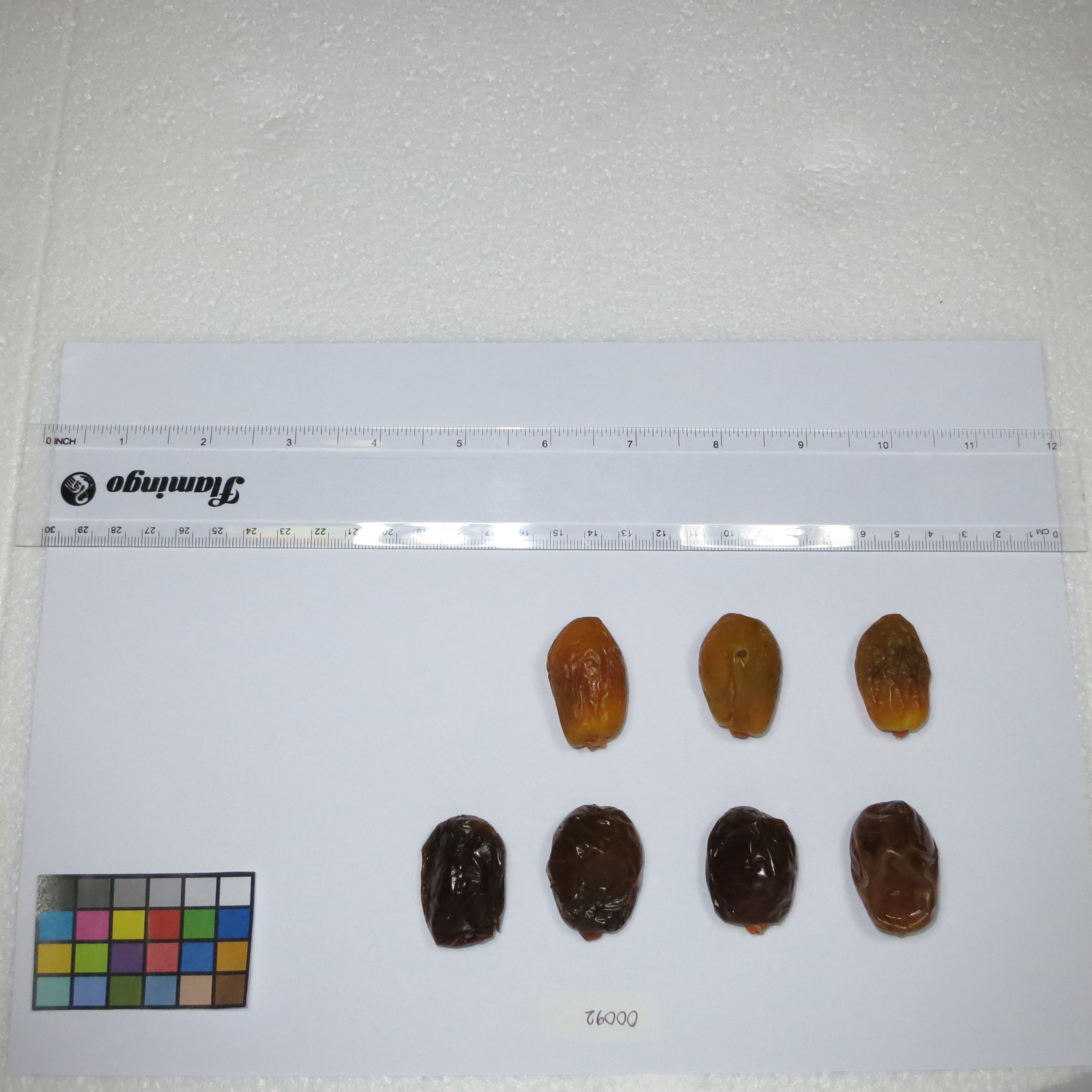

Supplement: Supplementary file 5 — Supplementary material [file mmc5.zip › dates images/00092.JPG]

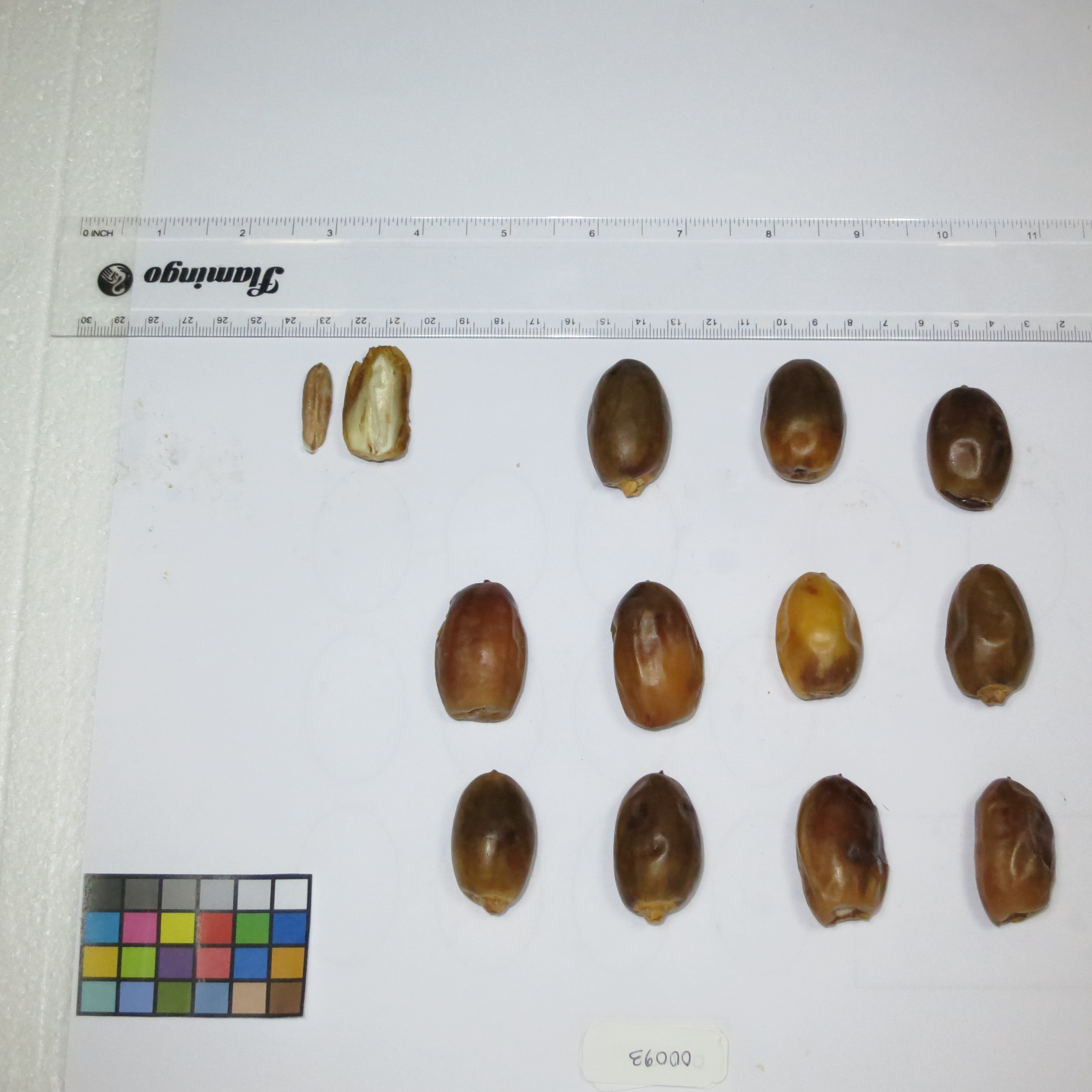

Supplement: Supplementary file 5 — Supplementary material [file mmc5.zip › dates images/00093.JPG]

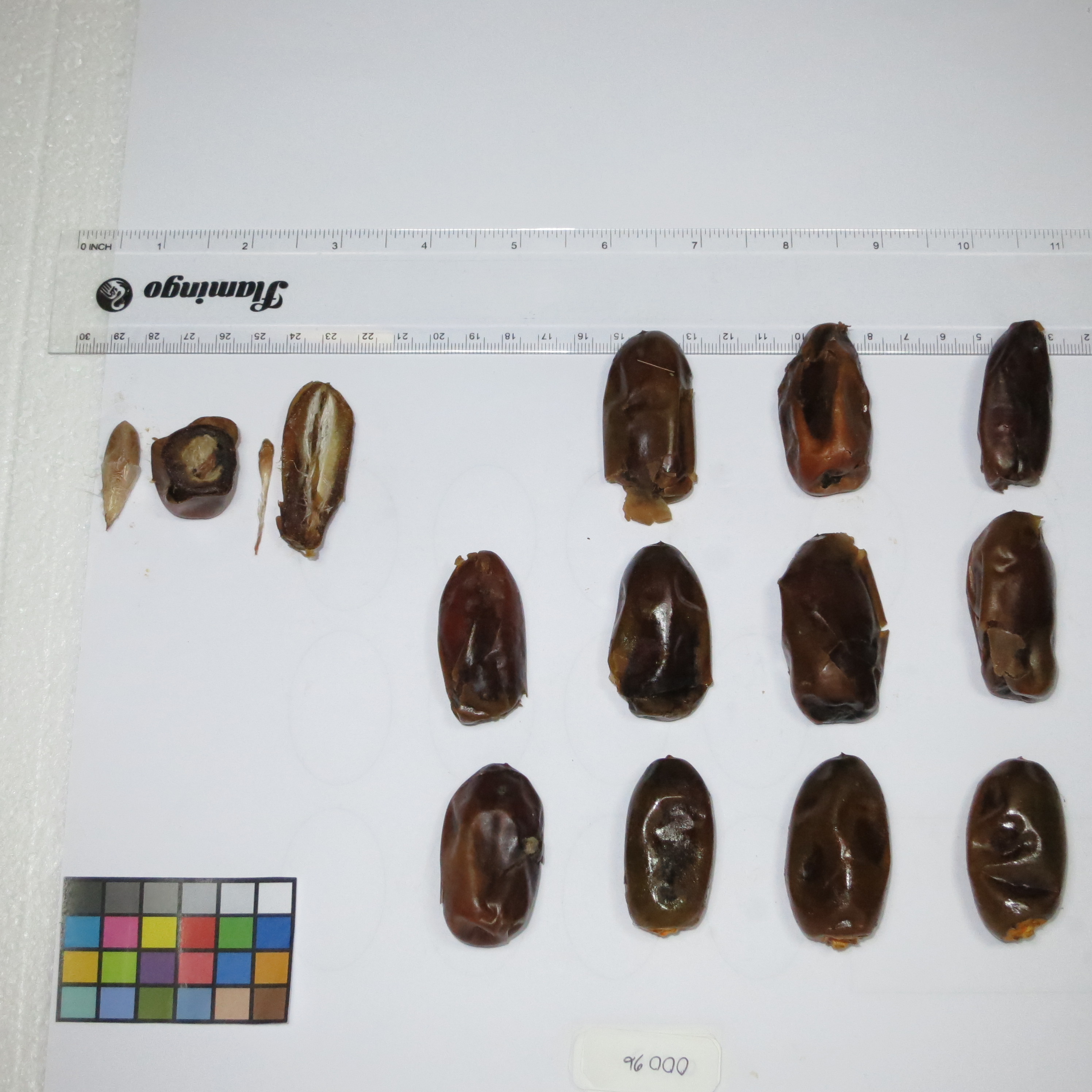

Supplement: Supplementary file 5 — Supplementary material [file mmc5.zip › dates images/00096.JPG]

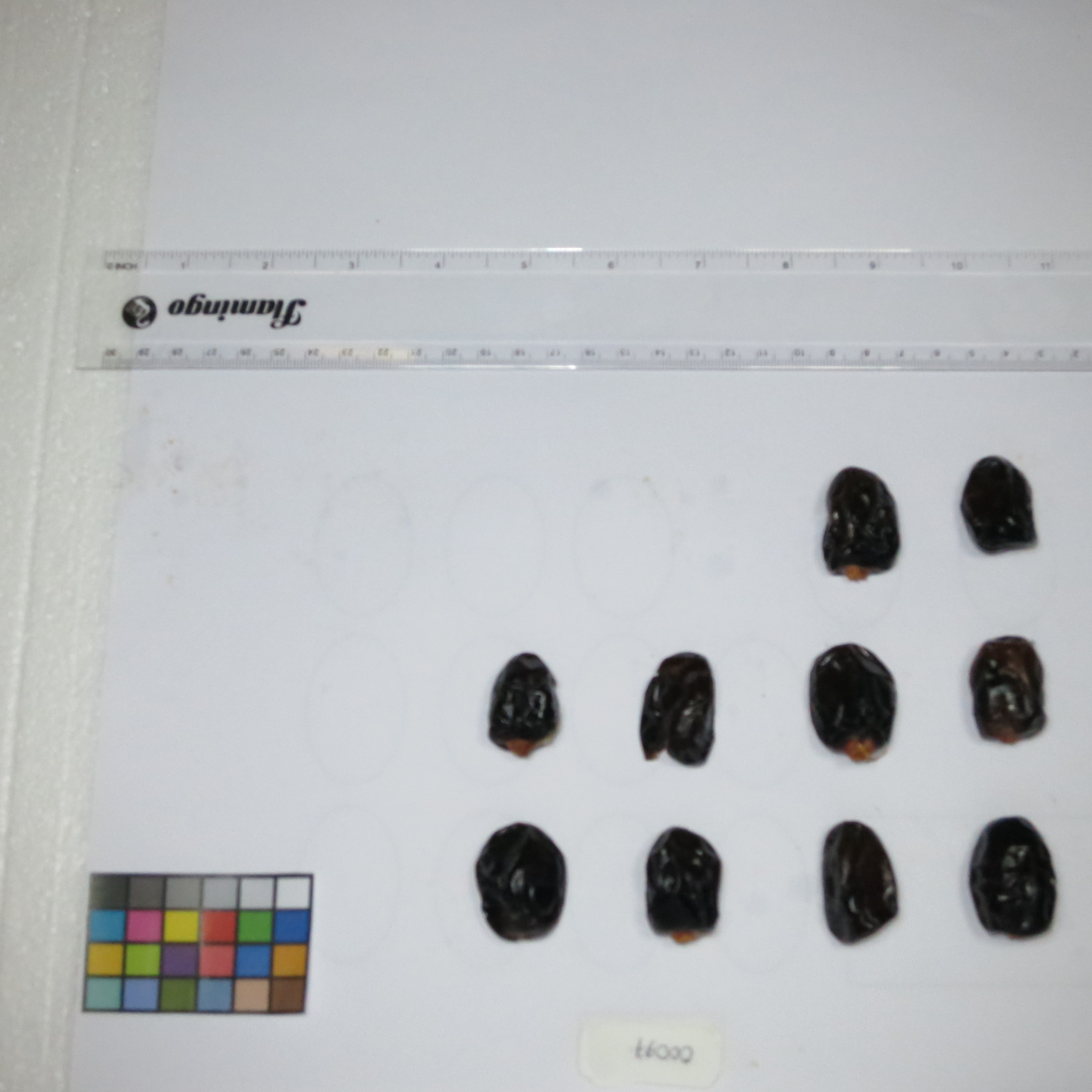

Supplement: Supplementary file 5 — Supplementary material [file mmc5.zip › dates images/00097.JPG]

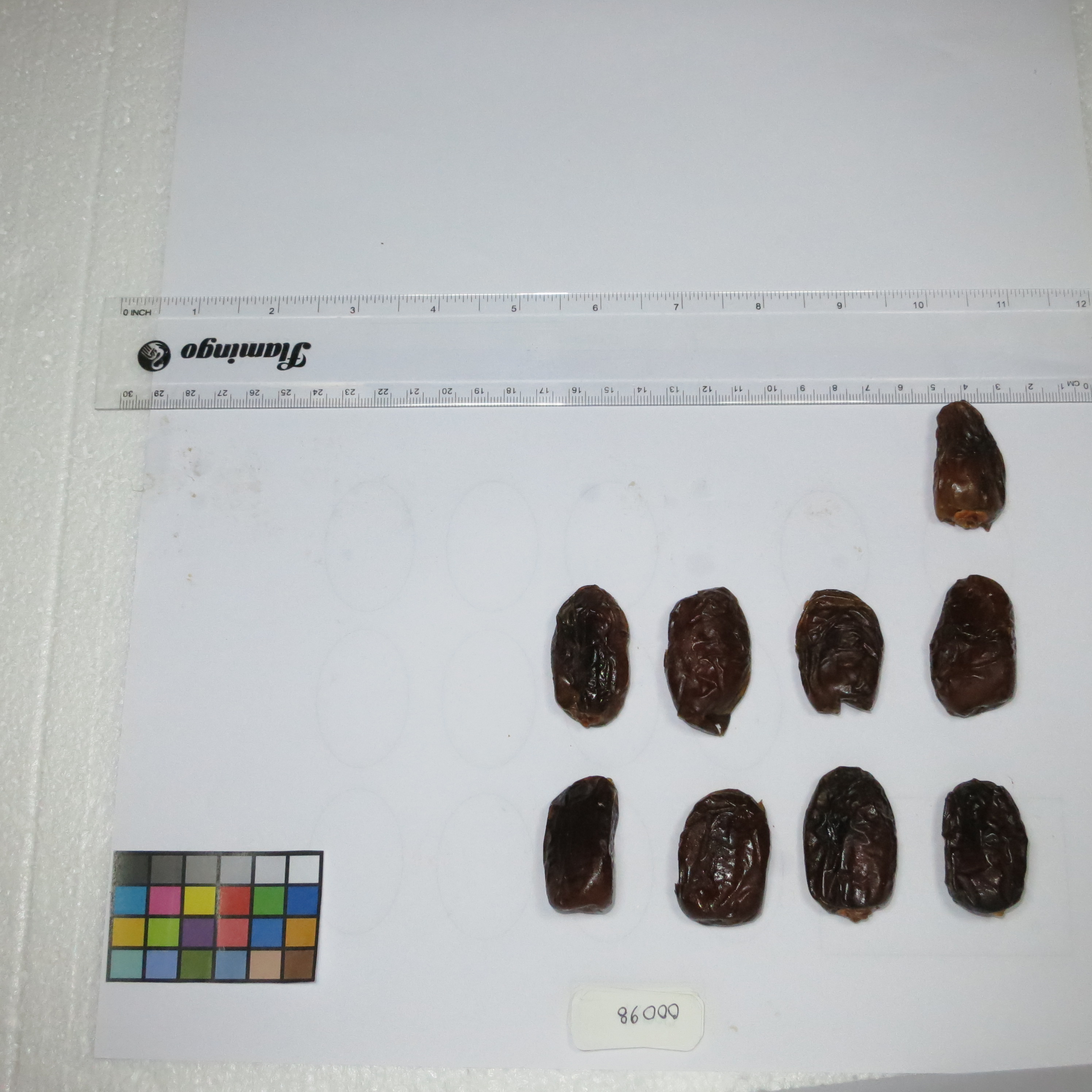

Supplement: Supplementary file 5 — Supplementary material [file mmc5.zip › dates images/00098.JPG]

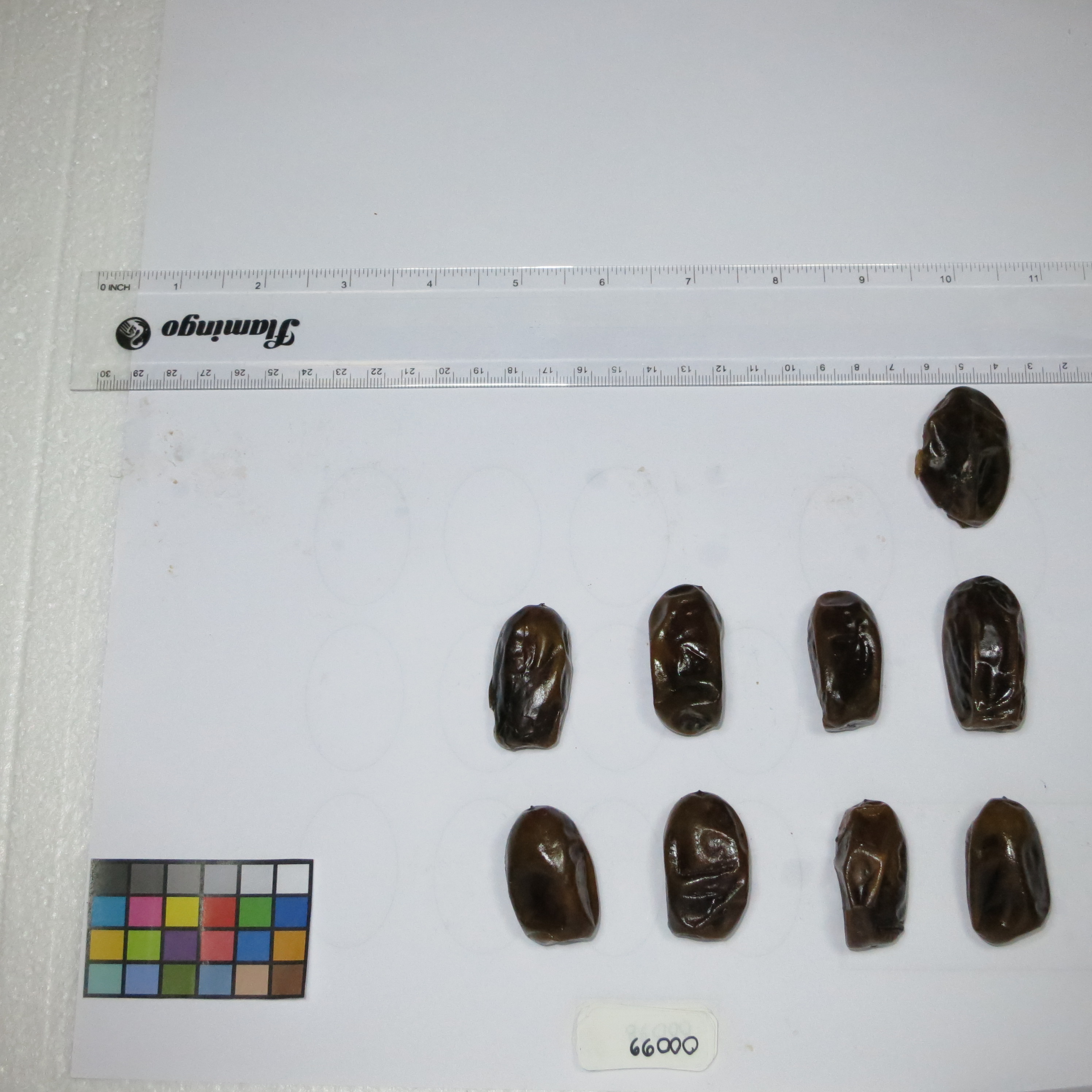

Supplement: Supplementary file 5 — Supplementary material [file mmc5.zip › dates images/00099.JPG]

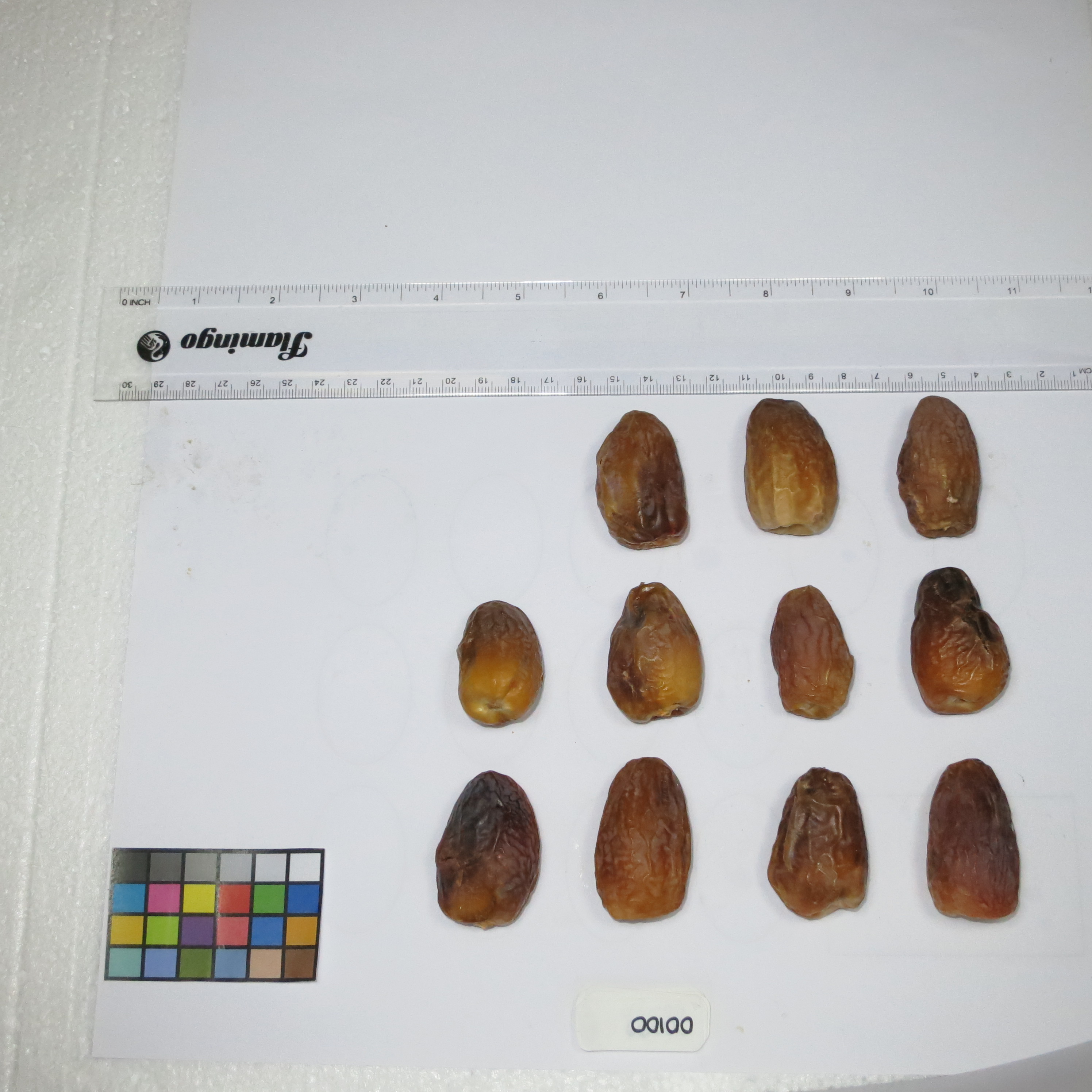

Supplement: Supplementary file 5 — Supplementary material [file mmc5.zip › dates images/00100.JPG]

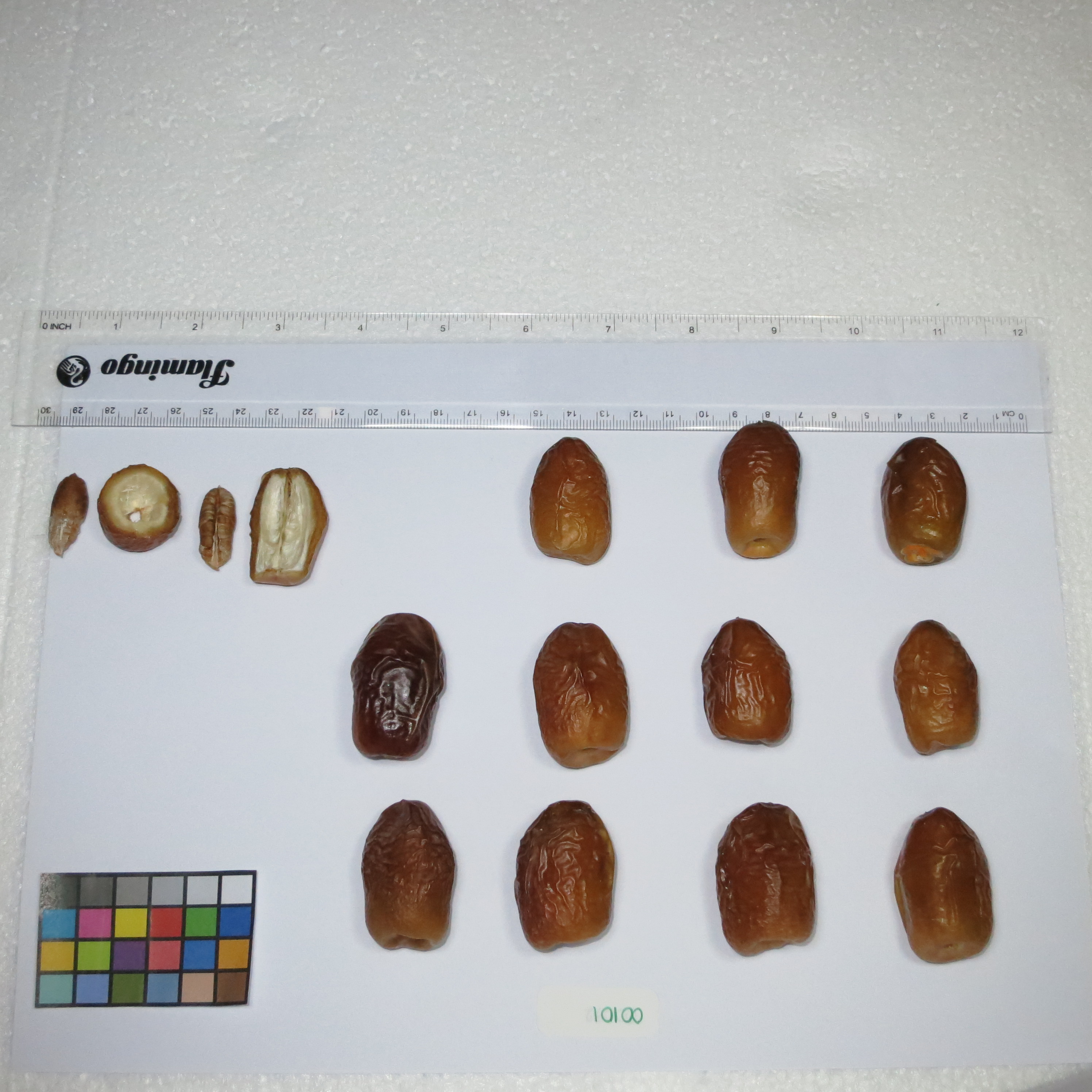

Supplement: Supplementary file 5 — Supplementary material [file mmc5.zip › dates images/00101.JPG]

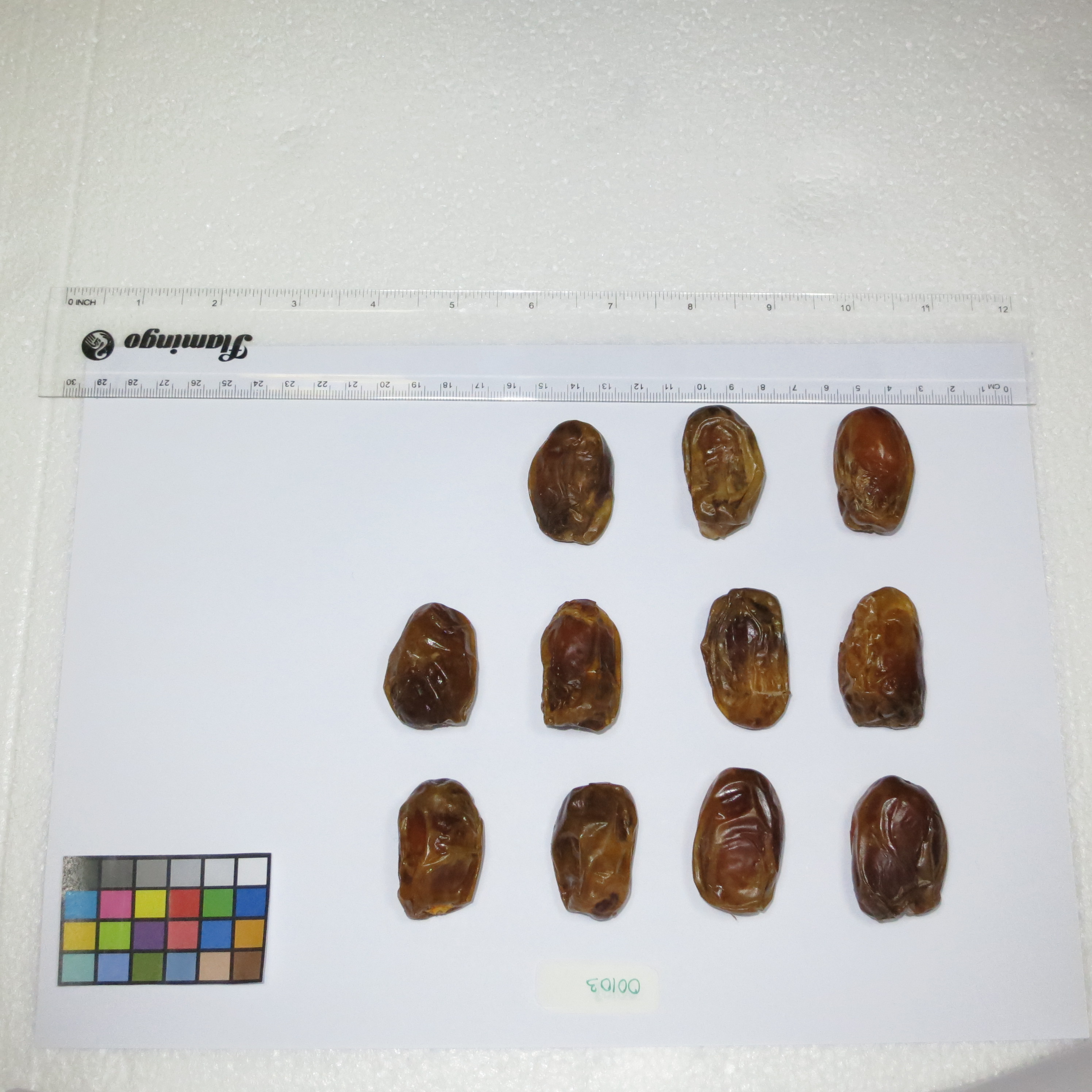

Supplement: Supplementary file 5 — Supplementary material [file mmc5.zip › dates images/00103.JPG]

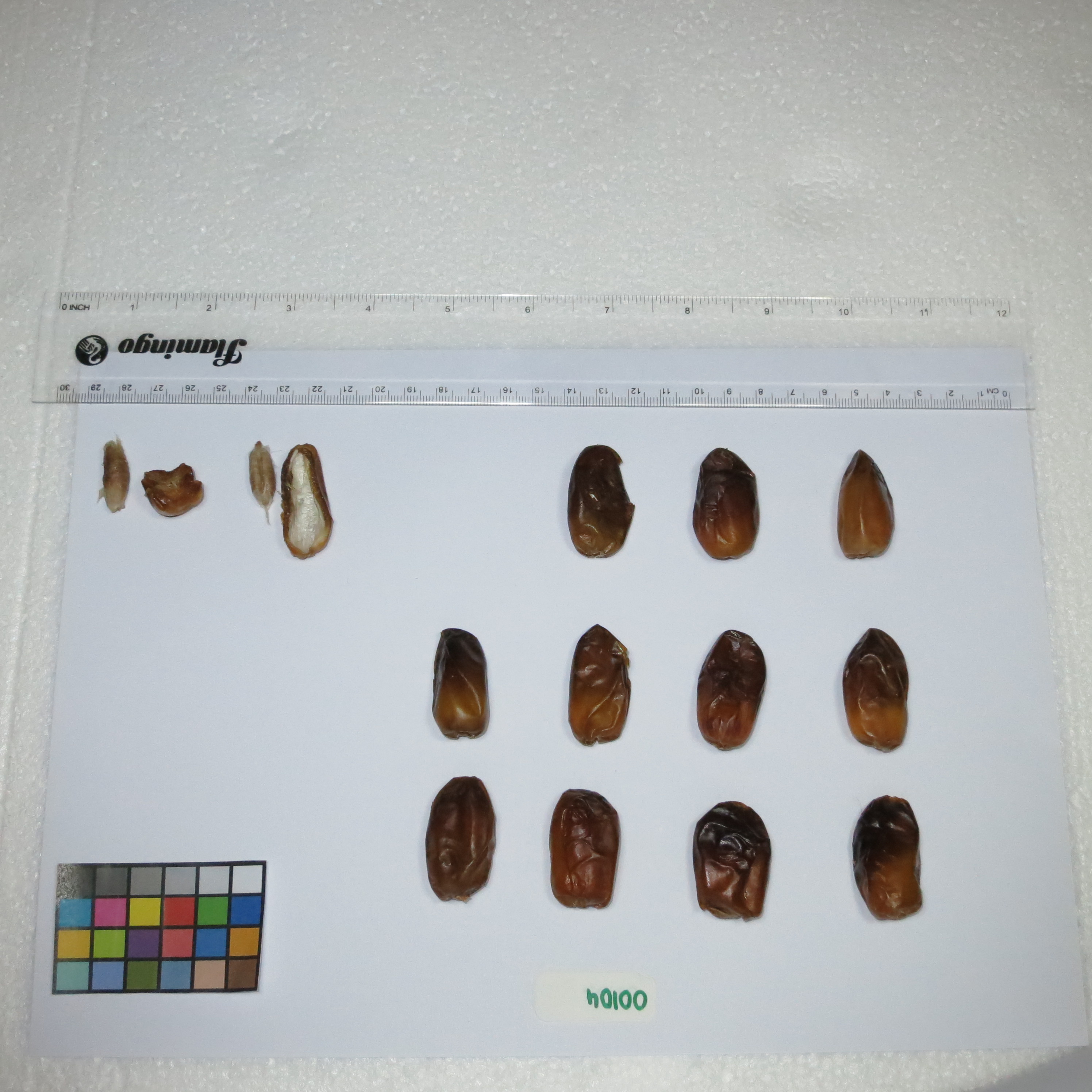

Supplement: Supplementary file 5 — Supplementary material [file mmc5.zip › dates images/00104.JPG]

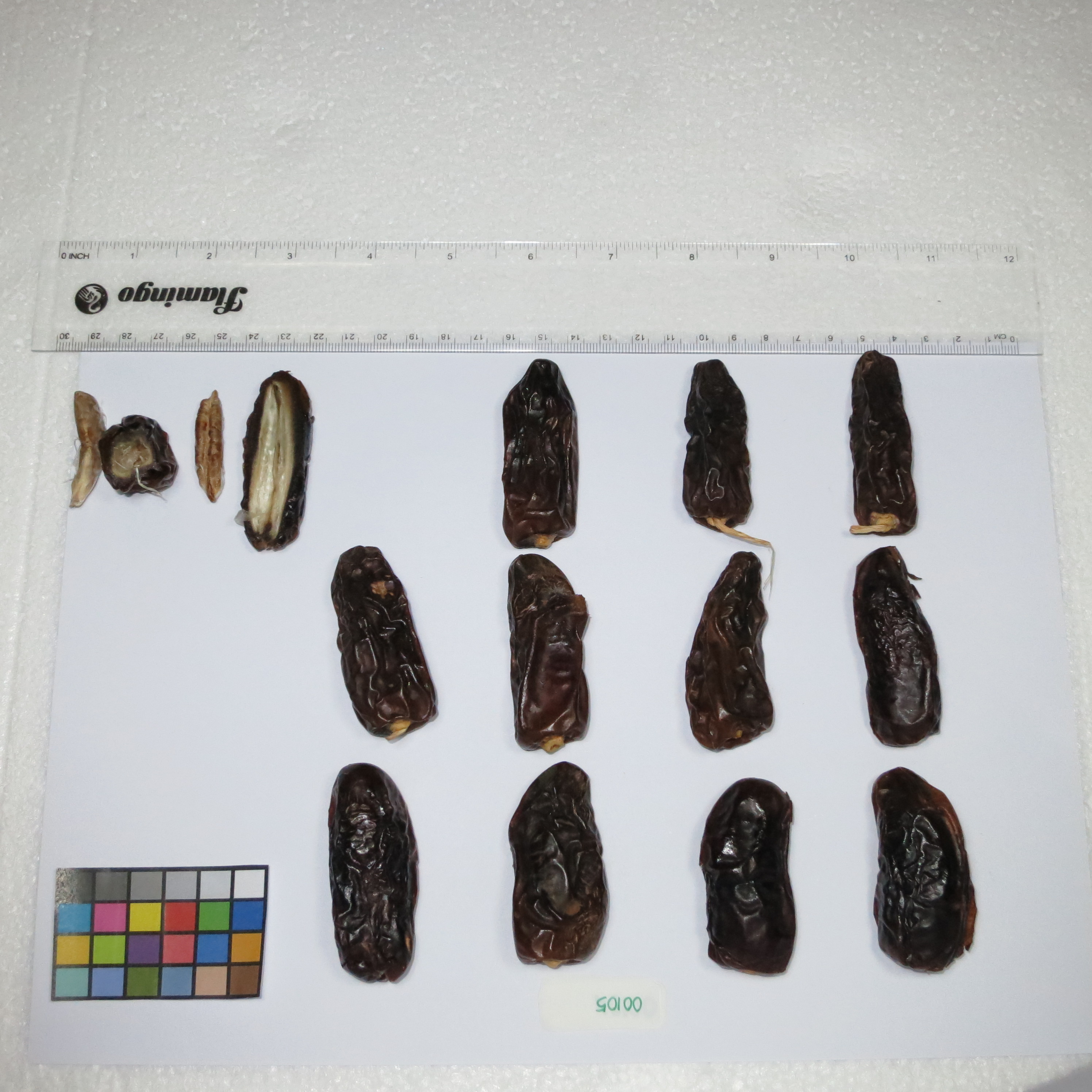

Supplement: Supplementary file 5 — Supplementary material [file mmc5.zip › dates images/00105.JPG]

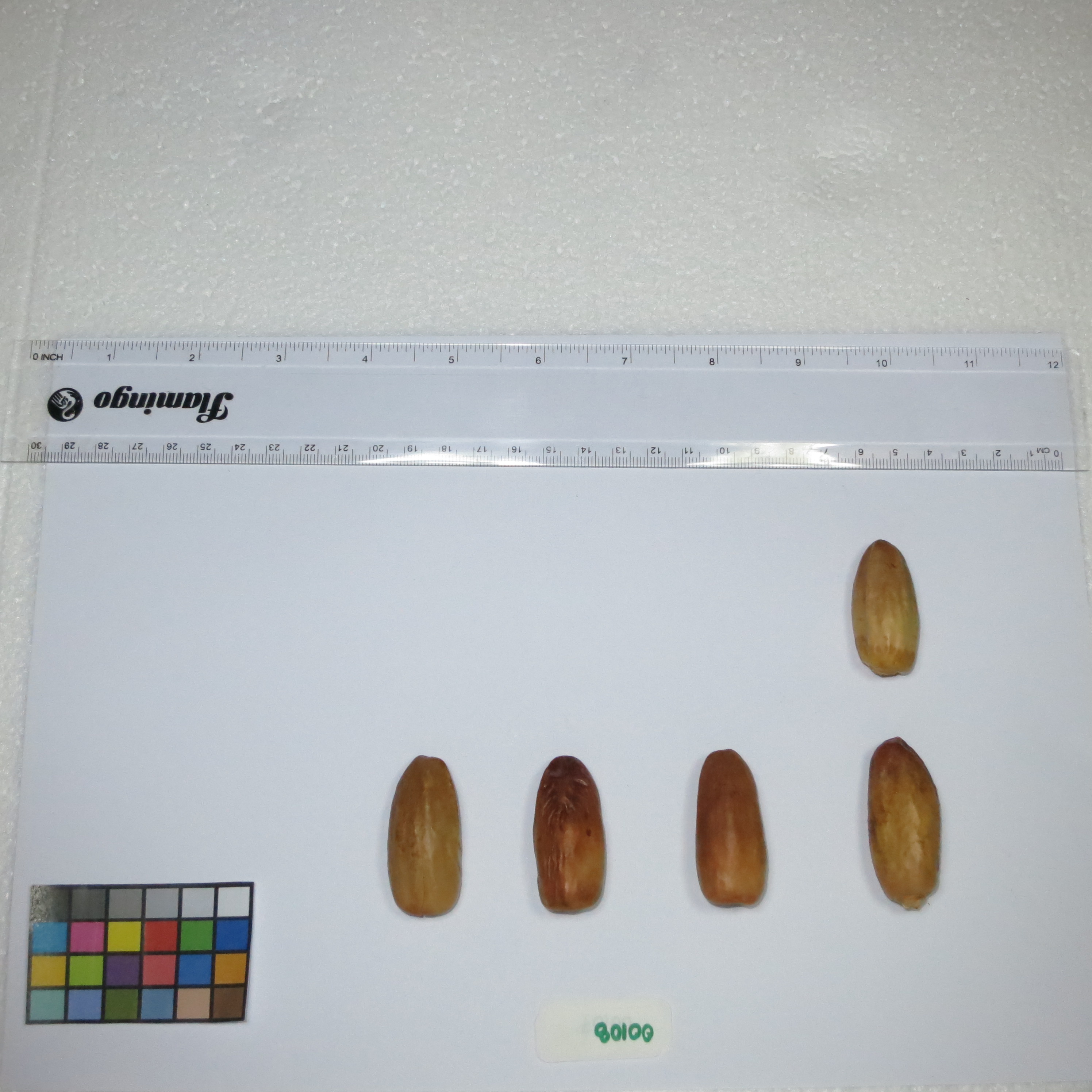

Supplement: Supplementary file 5 — Supplementary material [file mmc5.zip › dates images/00108.JPG]

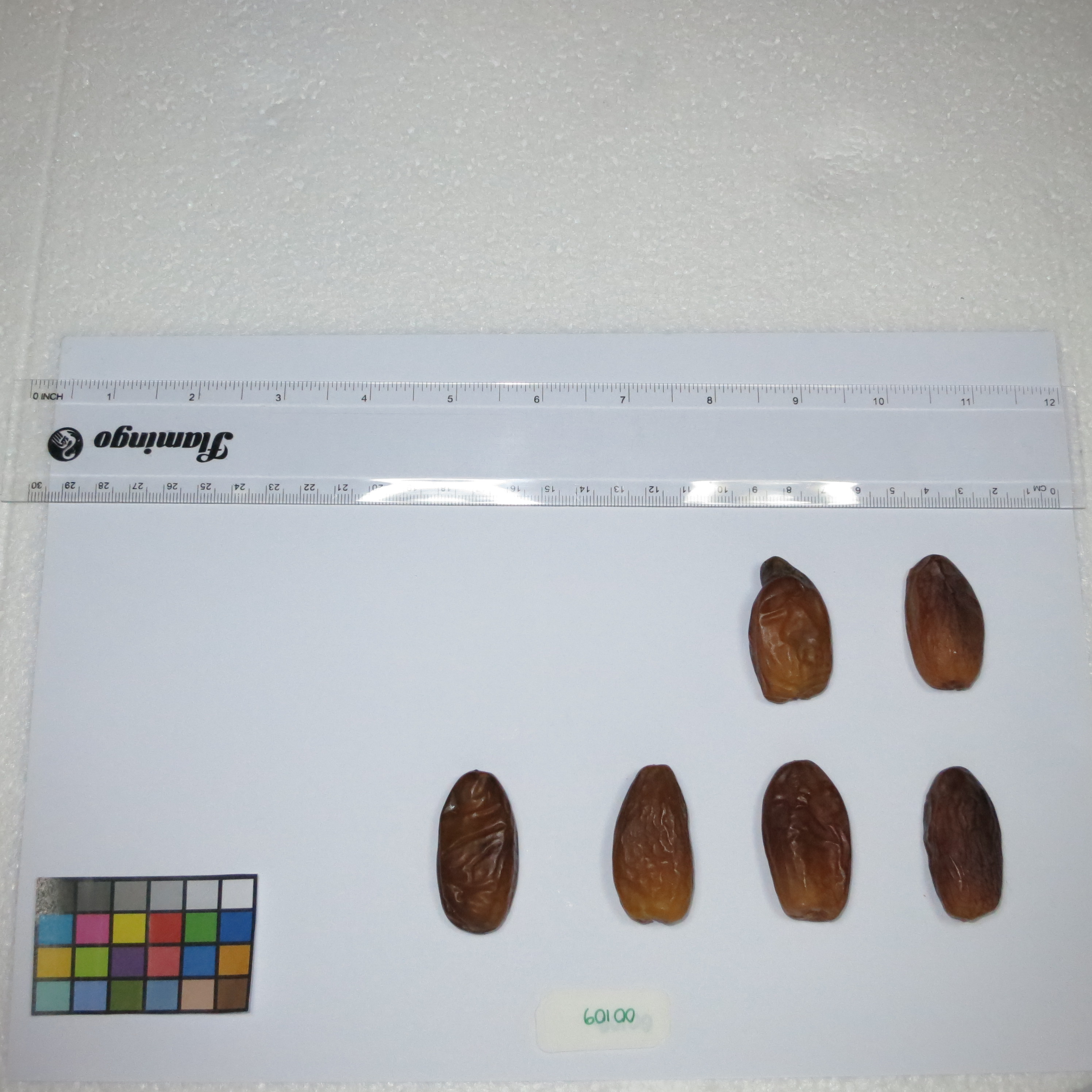

Supplement: Supplementary file 5 — Supplementary material [file mmc5.zip › dates images/00109.JPG]

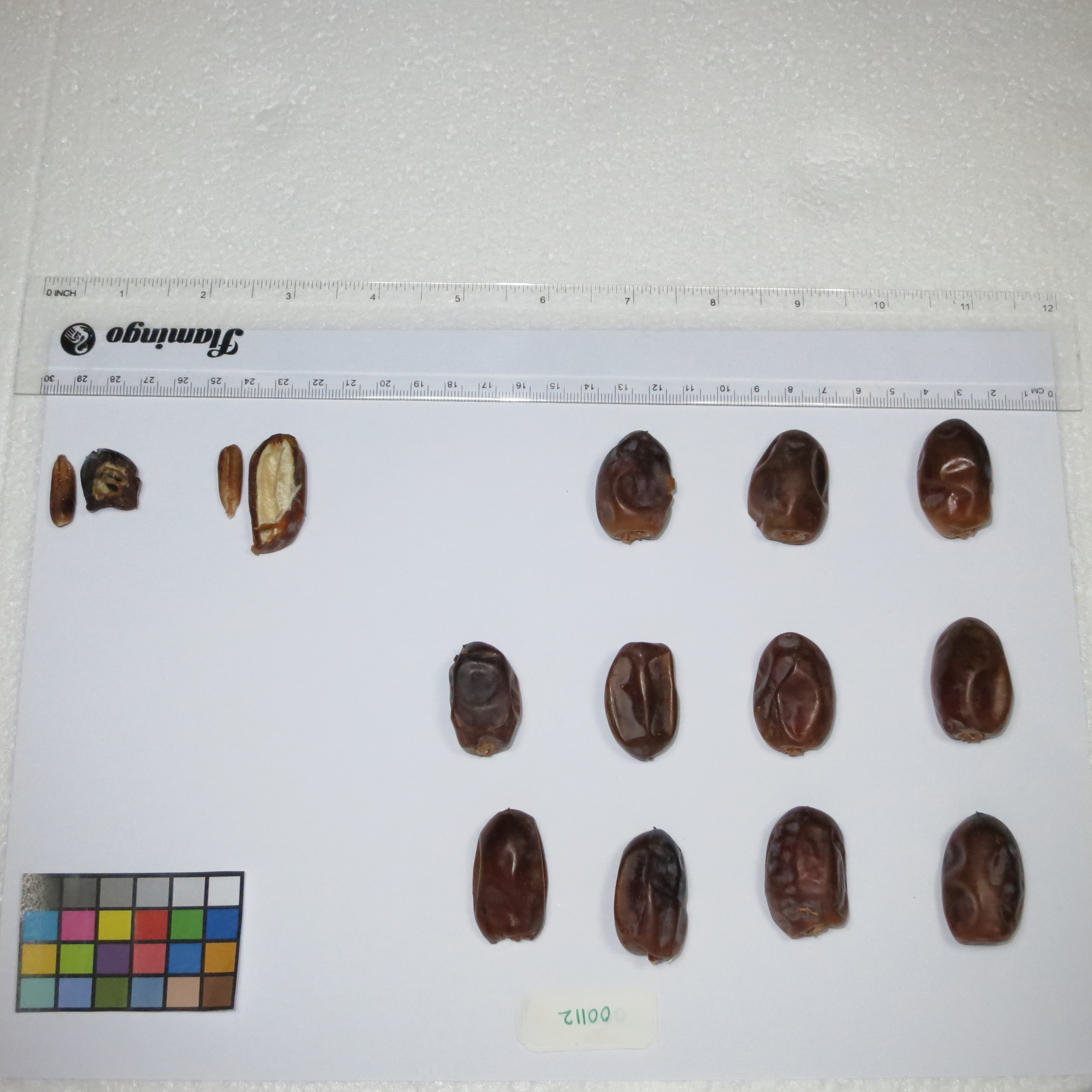

Supplement: Supplementary file 5 — Supplementary material [file mmc5.zip › dates images/00112.JPG]

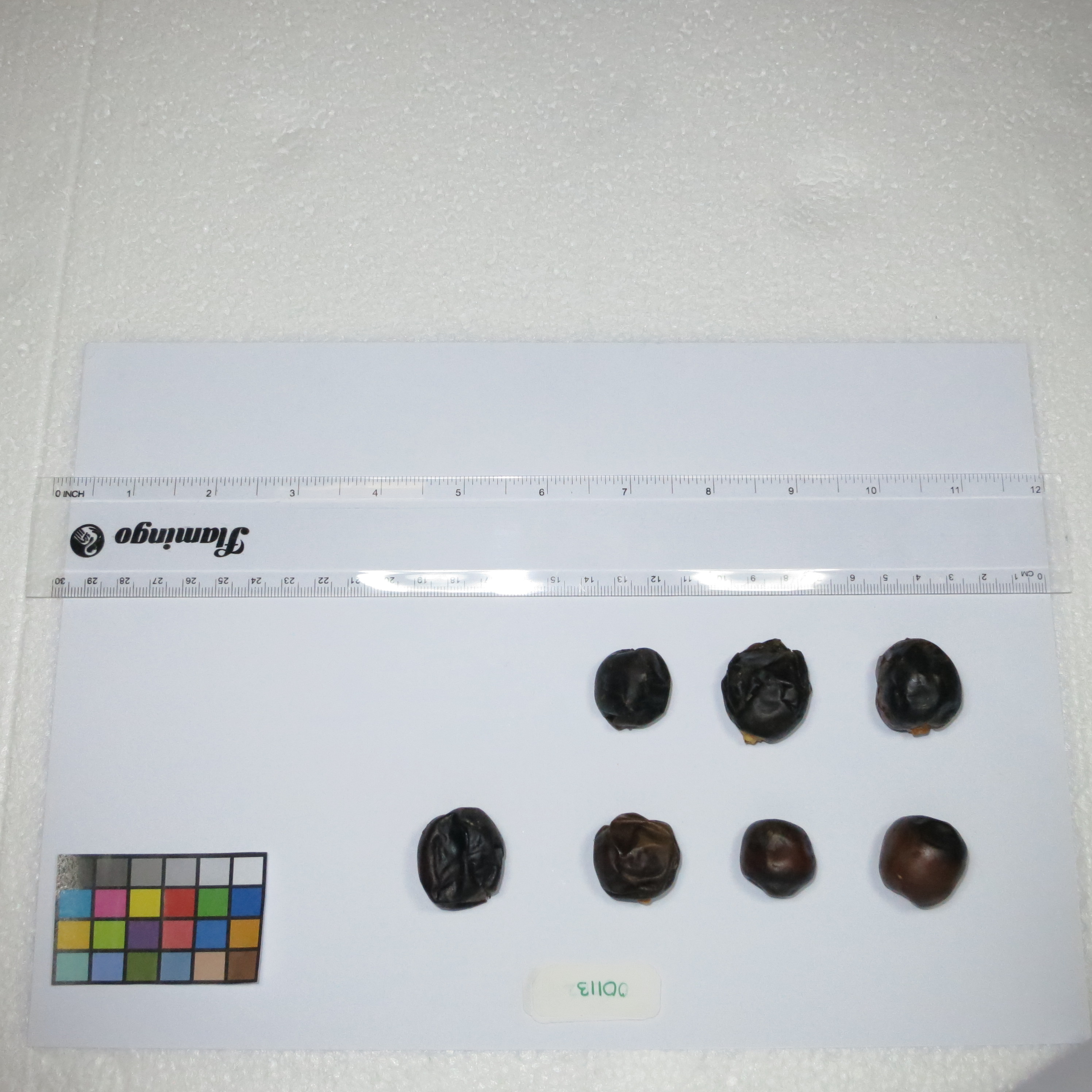

Supplement: Supplementary file 5 — Supplementary material [file mmc5.zip › dates images/00113.JPG]

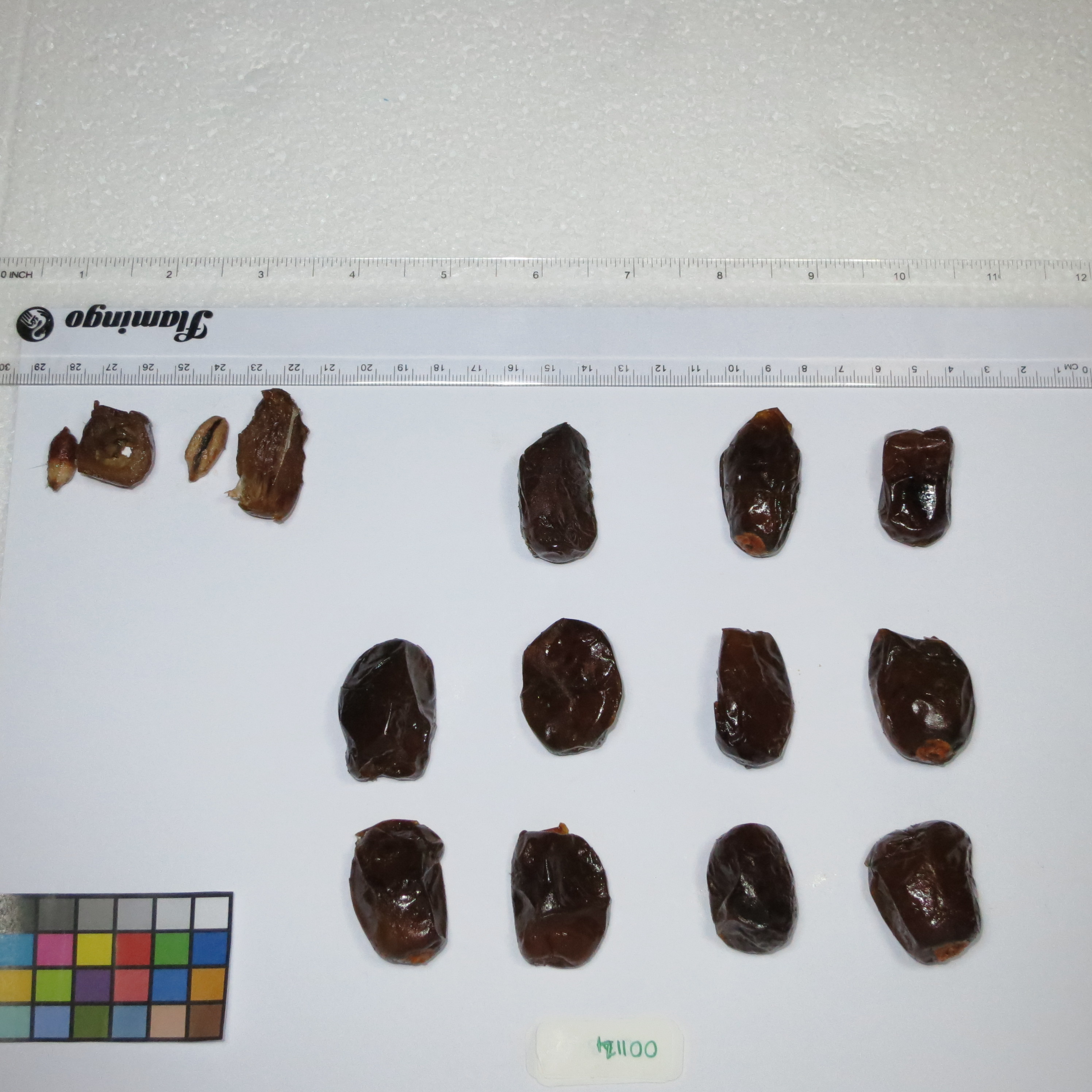

Supplement: Supplementary file 5 — Supplementary material [file mmc5.zip › dates images/00114.JPG]

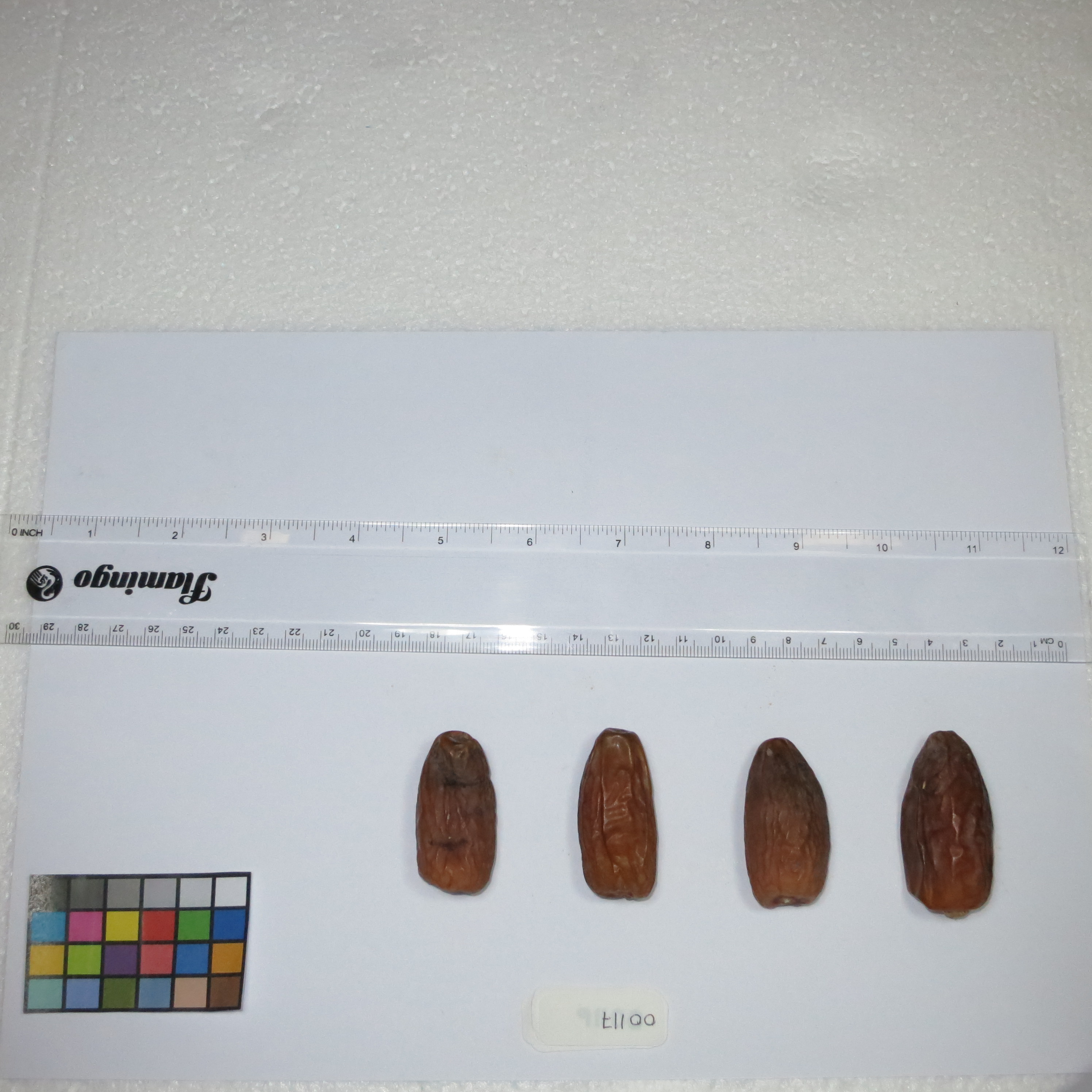

Supplement: Supplementary file 5 — Supplementary material [file mmc5.zip › dates images/00117.JPG]

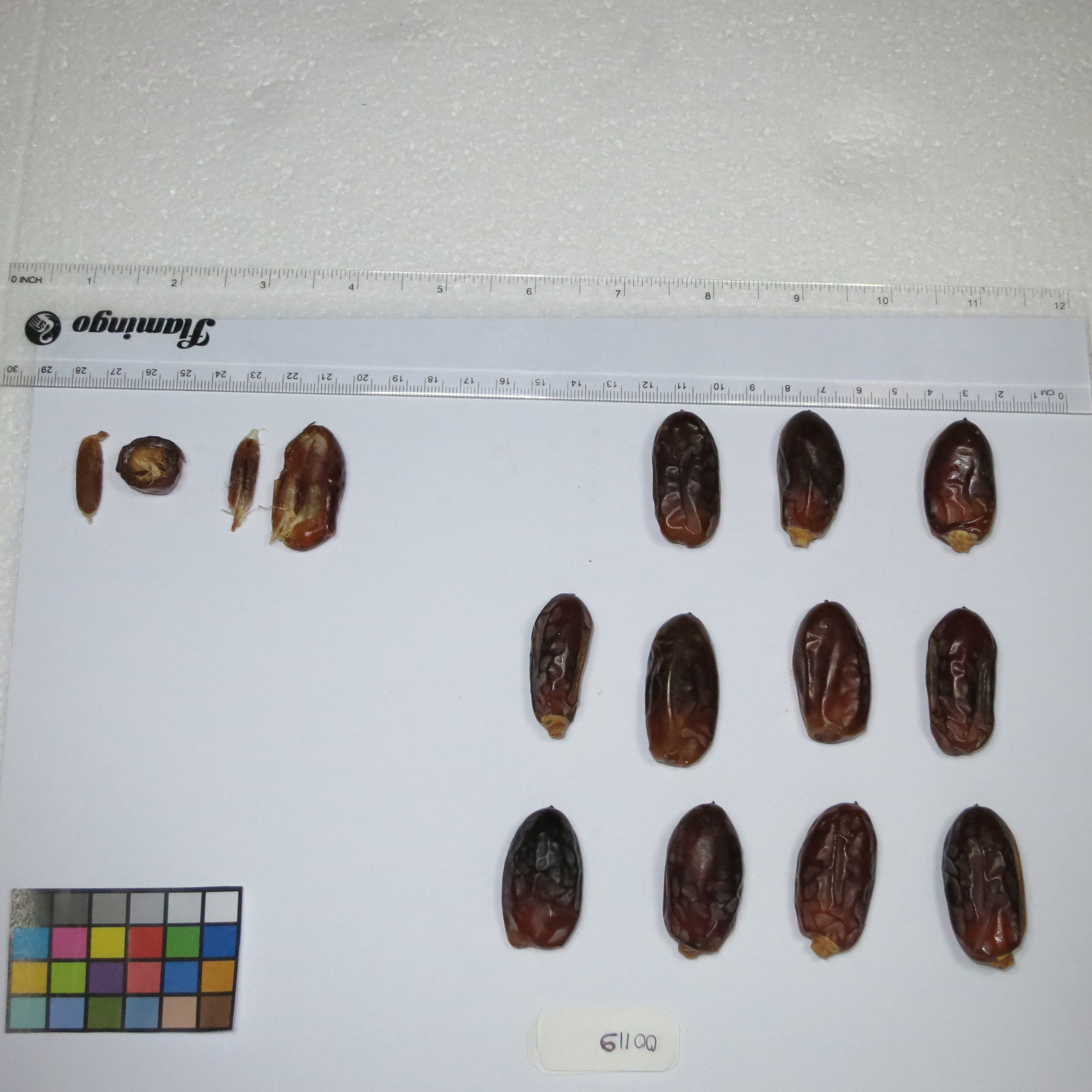

Supplement: Supplementary file 5 — Supplementary material [file mmc5.zip › dates images/00119.JPG]
